# Exploration of New Biomass‐Derived Solvents: Application to Carboxylation Reactions

**DOI:** 10.1002/cssc.201903224

**Published:** 2020-02-11

**Authors:** Ashot Gevorgyan, Kathrin H. Hopmann, Annette Bayer

**Affiliations:** ^1^ Department of Chemistry UiT The Arctic University of Norway 9037 Tromsø Norway; ^2^ Hylleraas Centre for Quantum Molecular Sciences Department of Chemistry UiT The Arctic University of Norway 9037 Tromsø Norway

**Keywords:** biomass valorization, carbon dioxide, carboxylation, organic synthesis, solvents

## Abstract

A range of hitherto unexplored biomass‐derived chemicals have been evaluated as new sustainable solvents for a large variety of CO_2_‐based carboxylation reactions. Known biomass‐derived solvents (biosolvents) are also included in the study and the results are compared with commonly used solvents for the reactions. Biosolvents can be efficiently applied in a variety of carboxylation reactions, such as Cu‐catalyzed carboxylation of organoboranes and organoboronates, metal‐catalyzed hydrocarboxylation, borocarboxylation, and other related reactions. For many of these reactions, the use of biosolvents provides comparable or better yields than the commonly used solvents. The best biosolvents identified are the so far unexplored candidates isosorbide dimethyl ether, acetaldehyde diethyl acetal, rose oxide, and eucalyptol, alongside the known biosolvent 2‐methyltetrahydrofuran. This strategy was used for the synthesis of the commercial drugs Fenoprofen and Flurbiprofen.

## Graphical abstract

**Sustainable solvents**: The number of biomass‐derived solvents can be expanded. Hitherto unexplored as well as known biomass‐derived solvents can be used for numerous carboxylation reactions with carbon dioxide. Biomass‐derived solvents, such as isosorbide dimethyl ether and acetaldehyde diethyl acetal, are found to be not only as good as but sometimes even better than common organic solvents.

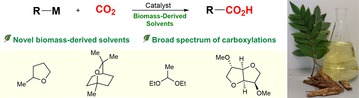

## Introduction

The vast majority of known organic transformations require use of a solvent. Solvents are essential not only for running a reaction, but also for the separation and purification of target products. As a result, solvents usually constitute over 80 % of all materials needed for the successful accomplishment of a typical synthetic transformation.^1^ However, most commonly used organic solvents are derived from fossil resources, are not renewable, and have high toxicities. This can cause serious environmental and economic issues for large‐scale chemical processes. One of the key directions of modern green chemistry is the minimization, elimination, or replacement of these solvents.^2^ In this regard, so‐called “solvent‐free′′ reactions have significant potential.^3^ However, most of them are not really solvent‐free and require large amounts of organic solvents for the workup and purification. In most cases, the research tasks cannot be achieved without solvents. Nevertheless, undesirable solvents can be replaced by sustainable/renewable alternatives. For instance, liquids or chemicals with low melting points, available from renewable resources, can fill the gap.^4^ Particularly, many chemicals derived from biomass share common properties with organic solvents derived from fossil resources. Importantly, most biomass‐derived chemicals fulfill many of the criteria for green solvents as proposed by Gu and Jerome, such as availability, renewability, low toxicity, biodegradability, and reasonable prices.^4^

**Figure 1:**
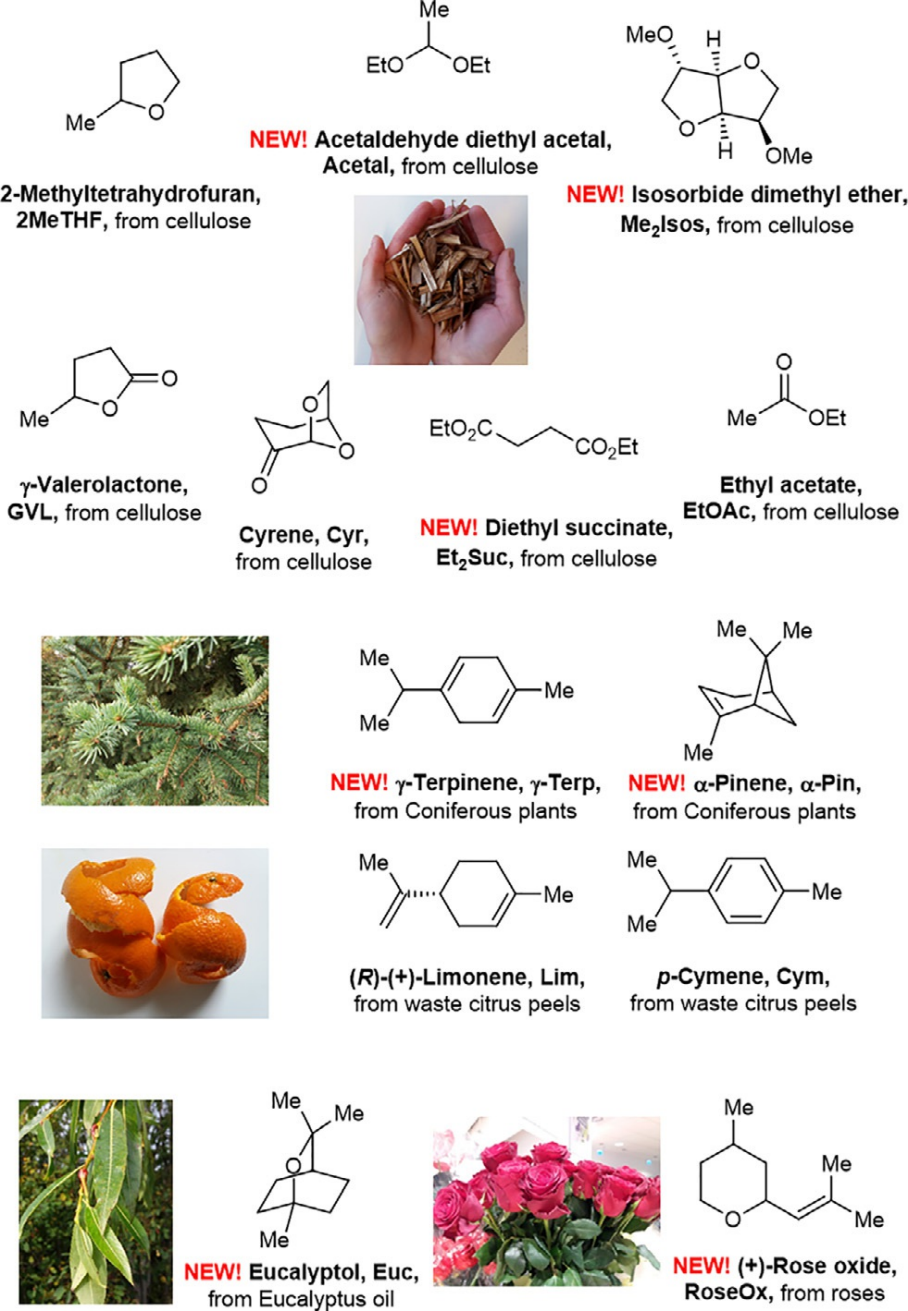
Overview of solvents used in this work (pictures taken by AG).

The main biomass‐derived solvents used in organic synthesis today are glycerol and its acetals, several low‐melting mixtures of carbohydrates, esters of lactic acid and gluconic acid, 2‐methyltetrahydrofuran (2MeTHF), cyrene (Cyr), limonene (Lim), *p*‐cymene (Cym), and γ‐valerolactone (GVL; Figure 1).^2^, ^4^, ^5^ The polar protic solvents like glycerol, carbohydrates as well as esters of lactic acid and gluconic acid are mainly used in condensation reactions and have found limited application in transformations involving organometallics.^4^ The polar aprotic (2MeTHF, GVL, Cyr) and nonpolar aprotic (Lim, Cym) solvents are far more popular and have been used for many classical transformations including transition metal (TM)‐catalyzed cross‐couplings^2^, ^4^, 5c and several C−H activations.5c–5e, 6 We should also highlight ethyl acetate (EtOAc), which is readily available from biomass and is often overlooked in the context of biomass‐derived solvents.

In addition, some biomass‐derived chemicals have been proposed as green solvents, such as isosorbide dimethyl ether (Me_2_Isos) and diethyl succinate (Et_2_Suc), both derived from cellulose but, to our knowledge, have not been examined for this purpose.7g, 8 Further new and yet‐unexplored candidates for biomass‐derived solvents in organic synthesis may include acetaldehyde diethyl acetal (Acetal), which is readily available from ethanol, γ‐terpinene (γ‐Terp) and α‐pinene (α‐Pin) available from various coniferous plants, eucalyptol (Euc) from eucalyptus oil, and rose oxide (RoseOx) from rose oil (Figure 1). The availability of these solvents can be judged based on their prices, which are comparable with those of common organic solvents and can decrease with further development of technologies in biorefinery (see the Supporting Information, Scheme S2). It should be emphasized that most of the solvents considered in the work here are quite safe and are used in large quantities in the food industry as flavor and fragrance ingredients.^9^ Low toxicity and biodegradability is particularly inherent to Acetal, Me_2_Isos, Et_2_Suc, Euc, RoseOx, and GVL. Unfortunately, information on overall environmental impacts and full life‐cycle assessments (LCAs) of the solvents introduced here remain limited.^7^, ^10^

**Scheme 1:**
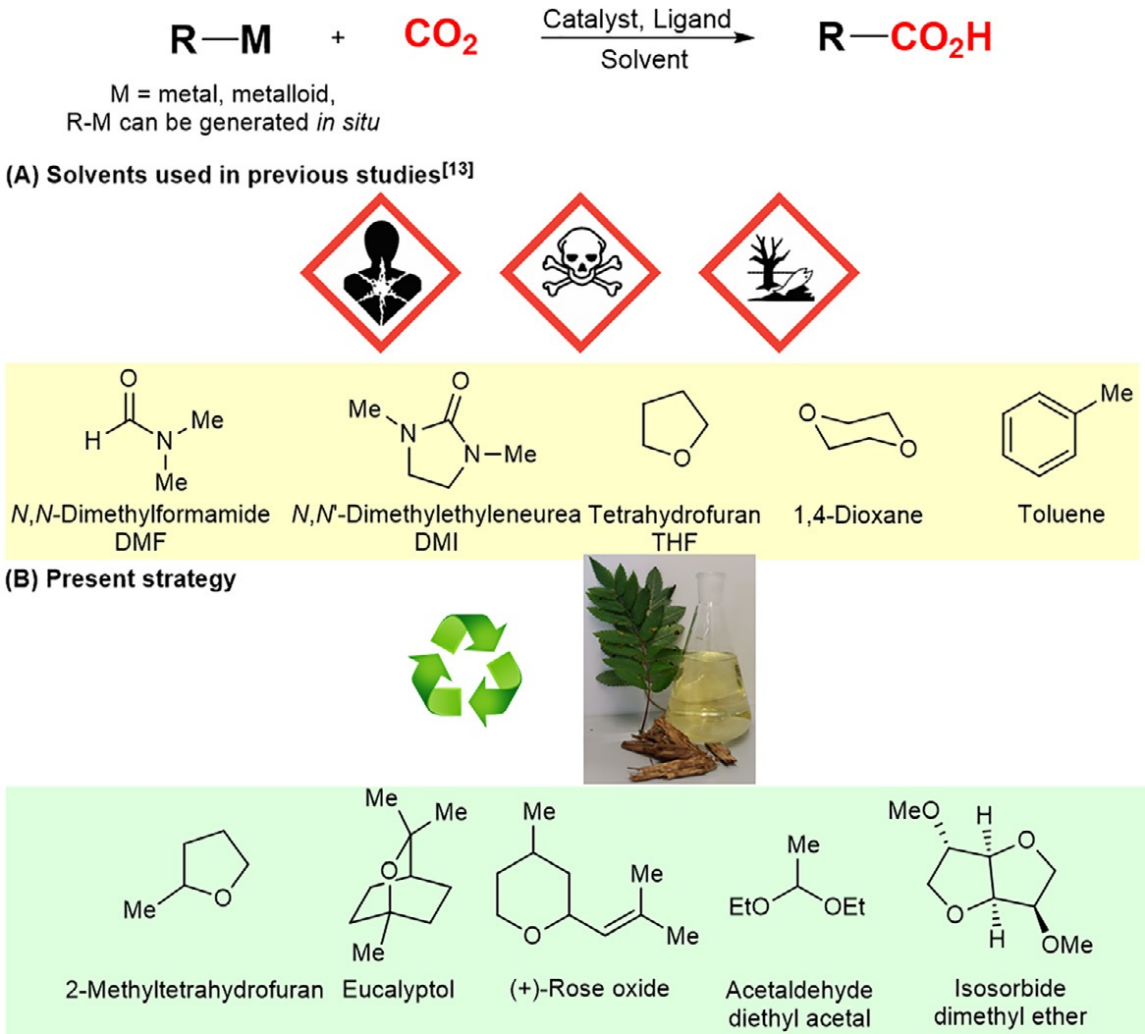
A) Solvents used in previous carboxylations;^13^ B) present strategy (picture taken by AG).

In the frame of our ongoing research program on C−C bond‐forming reactions involving CO_2_,^11^ we became particularly interested to examine the use of biomass‐derived solvents in a variety of carboxylative transformations (Scheme 1 B). Utilization of CO_2_,^12^ and particularly development of C−C bond‐forming reactions involving CO_2_,^13^ is a highly promising field of research that potentially can solve many global issues, such as replacement of depleting natural resources.^14^ Previously reported carboxylations were typically performed in DMF, dioxane, toluene, and other related solvents,^12^, ^13^ which are highly undesirable from the perspective of industry and green chemistry (Scheme 1 A). To our knowledge, biomass‐derived solvents have not been applied for any transformation involving CO_2_.

The main goal of the present study was to examine the suitability of a wide range of biomass‐derived chemicals as solvents for carboxylation reactions, including the known solvents 2MeTHF, GVL, Cyr, EtOAc, Lim, and Cym, and the unexplored solvents Acetal, Me_2_Isos, Et_2_Suc, γ‐Terp, α‐Pin, Euc, and RoseOx. We started out by screening the above listed biosolvents in two model reactions—carboxylations of in situ‐generated organoboranes and organoboronates. The carboxylative transformation of organoboronates to carboxylic acids in biosolvents was proven to be useful in the preparation of two commercial drugs. Finally, some of the best solvents were evaluated in a wide range of carboxylation reactions using CO_2_. Among others, these reactions included hydrocarboxylations, borocarboxylation, and carbocarboxylation. The biomass‐derived solvents were also successfully applied as extraction media during product isolation. Overall, biomass‐derived solvents, and in particular some of the solvents tested for the first time in this study, have a high potential to replace common organic solvents in the near future.

## Results and Discussion

**Figure 2:**
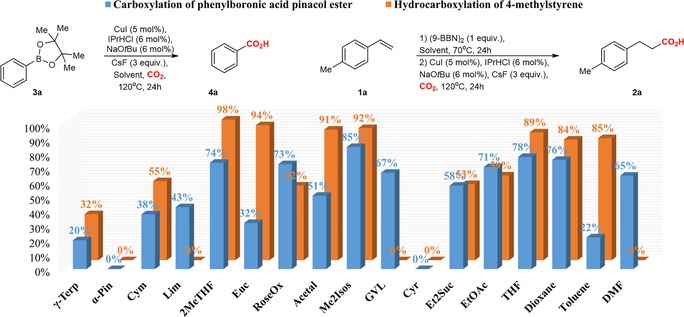
Screening of solvents for Cu‐catalyzed carboxylation of phenylboronic acid pinacol ester **3** **a** (blue) and hydrocarboxylation of 4‐methylstyrene **1** **a** (orange).

As a starting point, we have examined the carboxylation of organoboranes, which can be easily generated in situ by hydroboration of the corresponding olefins with 9‐BBN (9‐borabicyclo[3.3.1]nonane dimer).^15^ Analysis of the influence of various parameters on the outcome of the reaction were conducted on 4‐methylstyrene **1 a** using 2MeTHF as a solvent, which already proved to be a suitable media for reactions involving organometallics (Table 1).^2^, ^4^, ^5^ Careful examination of various parameters showed that the best yields of hydrocarboxylation can be achieved by using salts of copper combined with carbene ligands like IPrHCl, I*t*BuHBF_4_, and IAdHCl (Table 1, entries 1–6). Thus, **2 a** was isolated in quantitative yield by using in situ‐generated IPrCuI (5 mol %) catalyst precursor and CsF (3 equiv) as base when running the reaction at 120 °C for 24 h (Table 1, entry 1). Other catalysts and bases in general were less effective (see Tables S1–S4). Overall, our findings were in good agreement with previous reports performed in common solvents.^15^ The optimal conditions for hydrocarboxylation of **1 a** were also tested for a model organoboronate, the phenylboronic acid pinacol ester **3 a** (Figure 2 and Table S5).^16^ Gratifyingly, these conditions performed well for **3 a**, providing benzoic acid **4 a** isolated in 74 % yield. Having these promising results in 2MeTHF in hand, we focused on the screening of other biosolvents (Figure 2).

**Table 1:** Overview of the best conditions developed for hydrocarboxylation of **1 a** (see also Tables S1–S4).^[a]^ 
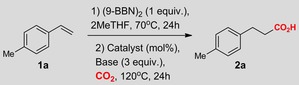

Entry	Catalyst (mol %)	Base	Yield [%]^[b]^
1	IPrHCl/CuI/NaO*t*Bu (5)	CsF	98
2	IPrHCl/CuBr/NaO*t*Bu (5)	CsF	93
3	IPrHCl/CuCl/NaO*t*Bu (5)	CsF	82
4	IPrHCl/Cu(OAc)_2_/NaO*t*Bu (5)	CsF	77
5	I*t*BuHBF_4_/CuI/NaO*t*Bu (5)	CsF	68
6	IAdHCl/CuI/NaO*t*Bu (5)	CsF	84
7	IPrHCl/CuI/NaO*t*Bu (3)	CsF	84
8	IPrHCl/CuI/NaO*t*Bu (5)	KO*t*Bu^[c]^	75
9	IPrHCl/CuI/NaO*t*Bu (5)	Cs_2_CO_3_	95

[a] Reaction conditions: 1) **1 a** (0.846 mmol), (9‐BBN)_2_ (1 equiv), 2MeTHF (3 mL), 70 °C, 24 h. 2) Catalyst (3–5 mol %), 2MeTHF (1 mL), base (3 equiv), CO_2_ (120 mL), 120 °C, 24 h. [b] Isolated product. [c] The reaction mixture was stirred at 20 °C for 30 min before addition of CO_2_. IPrHCl=1,3‐bis(2,6‐diisopropylphenyl)imidazolium chloride; I*t*BuHBF_4_=1,3‐di‐*tert*‐butylimidazolium tetrafluoroborate; IAdHCl=1,3‐bis(1‐adamantyl)imidazolium chloride.

Among the solvents analyzed for the hydrocarboxylation of **1 a**, the highest yields of **2 a** were obtained in the biomass‐derived ethers 2MeTHF (98 %), Euc (94 %), Me_2_Isos (92 %), and Acetal (91 %; Figure 2). In comparison, the best traditional solvents were THF (89 %), dioxane (84 %), and toluene (85 %). Biomass‐derived solvents possessing double bonds or other functional groups that could be reduced such as α‐Pin, Lim, GVL, and Cyr were not good media for hydrocarboxylation of **1 a**. The reason could be that the functional groups present in the solvent interact with 9‐BBN. However, comparable solvents such as RoseOx, γ‐Terp, Et_2_Suc, and EtOAc gave moderate yields of **2 a** (52, 32, 53, and 59 %, respectively). In addition, we observed moderate yields of **2 a** in Cym (55 %), whereas toluene gave good yields for the hydrocarboxylation of **1 a** (85 %).

Similarly, screening of biomass‐derived solvents for the carboxylation of **3 a** showed that the best yields of **4 a** can be achieved in biomass‐derived ethers Me_2_Isos (85 %), 2MeTHF (74 %), and RoseOx (73 %; Figure 2). In addition, the reaction worked well in the ester EtOAc (71 %). The traditional solvents THF (78 %) and dioxane (76 %) provided comparable yields. Moderate yields were obtained for several of the remaining solvents, such as Lim (43 %), Acetal (51 %), GVL (67 %), and Et_2_Suc (58 %). In comparison, Acetal was one of the best solvents for the hydrocarboxylation of **1 a**, whereas the hydrocarboxylation did not work in Lim and GVL. Eventually, both carboxylation and hydrocarboxylation did not work in α‐Pin and Cyr. In the case of Cyr, we observed a massive polymerization of the solvent for both reactions.

Our analysis shows that biomass‐derived solvents can provide excellent yields for the tested hydrocarboxylation and carboxylation reactions. Previously unexplored Me_2_Isos and Acetal are among the best solvents. The reactions worked particularly well in biomass‐derived ethers, outperforming traditional organic solvents.

**Scheme 2:**
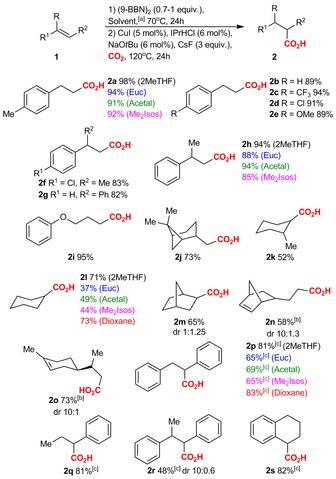
Scope of Cu‐catalyzed and Cu‐free hydrocarboxylation of olefins. [a] Unless otherwise mentioned, the reaction was performed in 2MeTHF. [b] Conducted using 0.7 equivalents of 9‐BBN. [c] The reactions were performed without Cu catalyst.

With these promising results in hand, we examined the substrate dependence of hydrocarboxylation of olefins in biomass‐derived solvents (Scheme 2; for an overview of used starting materials, see Scheme S3). The solvents that showed the best performance for the hydrocarboxylation (2MeTHF, Euc, Acetal, and Me_2_Isos) were examined for the disubstituted styrene **1 h**, cyclohexene **1 l**, and stilbene **1 p**. In the case of **1 h**, Acetal was as efficient as 2MeTHF, providing 94 % yield of **2 h** in both solvents, whereas Euc (88 %) and Me_2_Isos (85 %) gave slightly lower yields (Scheme 2). For cyclohexene **1 l**, the yields of **2 l** varied from 37–71 % with best results in 2MeTHF (71 %). However, when running the reaction in dioxane, the yield of **2 l** was slightly improved (73 %). For *trans*‐stilbene **1 p**, involving a benzylic organoboron intermediate, hydrocarboxylation in biomass‐derived solvents was investigated by using our previously reported cesium‐mediated conditions without Cu catalyst.11d The best biosolvent was again 2MeTHF (81 %) and reaction in dioxane gave comparable yields (83 %; see also Table S6).

Having established that 2MeTHF is a suitable solvent for hydrocarboxylations, we continued to investigate the scope of this reaction (Scheme 2). Styrenes **1 a**–**h** and primary olefins **1 i** and **1 j** consistently provided moderate to excellent yields of the corresponding acids (**2 a**–**j**, 73–98 %). For these systems, the hydroboration step proceeded as an anti‐Markovnikov addition, eventually leading to terminal carboxylic acids with excellent regioselectivity.^15^, ^17^ Other regioisomers were not observed. Further studies showed that internal alkenes **1 k**–**m** are far less reactive than terminal olefins (**2 k**–**m**, 52–71 %). However, for these substrates the reactions proceeded with excellent regioselectivities owing to the steric control of the hydroboration step.^15^, ^17^ The reduced reactivity of internal alkenes allowed us to conduct regioselective hydrocarboxylation on nonconjugated dienes possessing one internal and one terminal double bond, **1 n** and **1 o**. In this case, we used 0.7 equivalents of 9‐BBN, which allowed us to prepare only the hydrocarboxylation product of the terminal double bond (**2 n**, 58 %; **2 o**, 73 %). These observations may explain why the hydrocarboxylation of **1 a** worked in RoseOx and γ‐Terp, which have internal double bonds (Figure 2). The Cu‐free hydrocarboxylation of stilbenes and β‐substituted styrenes **1 p**–**s** also worked well in 2MeTHF and the hydrocarboxylation products **2 p**–**s** were observed in good yields (48–82 %) and excellent regioselectivity.

**Scheme 3:**
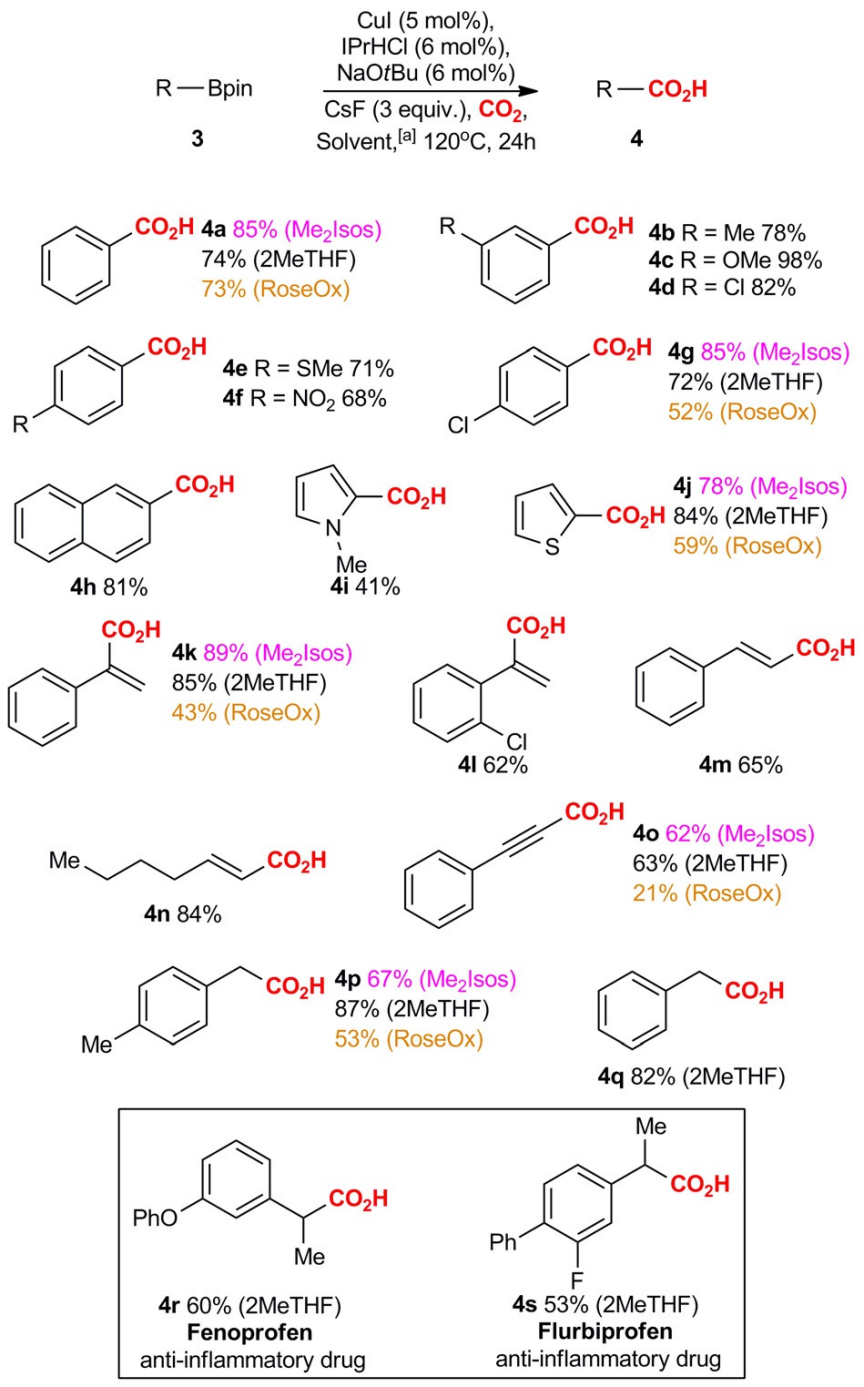
Scope of Cu‐catalyzed carboxylation of boronic acid pinacol esters. [a] Unless otherwise mentioned, the reaction was performed in Me_2_Isos.

Next, we examined the substrate dependence of carboxylations of organoboronates in biomass‐derived solvents (Scheme 3). The best solvents identified for organoboronates (Figure 2) were screened on several substrates. These studies indicated that depending on the substrate, the efficiency of the used solvent differs. For most aromatic systems, the best solvent was Me_2_Isos. However, for thiophene **4 j**, 2MeTHF performed slightly better (78 % vs. 84 %). For benzylboronic acid pinacol esters, 2MeTHF proved to be the best solvent, outperforming Me_2_Isos by 20 % (**4 p**, Scheme 3).

A range of differently substituted arylboronic acid pinacol esters **3 a**–**j** were then carboxylated in Me_2_Isos as the solvent. We found that the reaction works well for systems possessing electron‐rich (**4 c**, 98 %; **4 e**, 71 %) or ‐deficient (**4 d**, 82 %; **4 f**, 68 %; **4 g**, 85 %) aryl groups. Fused systems such as naphthalene **4 h** (81 %) and heterocycles **4 i** (41 %) and **4 j** (78 %) were also successfully carboxylated. Analysis of other organoboronates revealed that the reaction has a general character and works equally well for alkenyl‐ (**4 k**, 89 %; **4 l**, 62 %; **4 m**, 65 %; **4 n**, 84 %) and alkynylboronic acid pinacol esters (**4 o**, 62 %). The scope of the carboxylation of benzylboronic acid pinacol esters was evaluated in 2MeTHF as the solvent and a range of benzylic boronates were successfully transformed into the corresponding acids in good yields (**4 p**, 87 %; **4 q**, 82 %; **4 r**, 60 %; **4 s**, 53 %).

The developed methodology for benzylboronic acid pinacol esters could be further applied for the synthesis of the commercial nonsteroidal anti‐inflammatory drugs Fenoprofen (**4 r**, 60 %) and Flurbiprofen (**4 s**, 53 %). Notably, the starting materials of these drugs were prepared in two steps from the corresponding commercially available aldehydes. These steps involved sequential Wittig olefination and Cu‐catalyzed hydroboration reactions, which were conducted in biomass‐derived solvents (2MeTHF and Cym, respectively; see the Supporting Information).

**Scheme 4:**
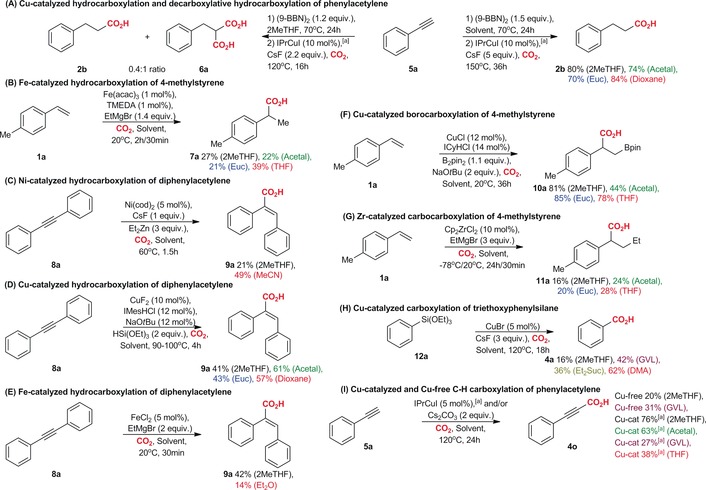
Screening of other carboxylation reactions in biosolvents. [a] The catalyst was generated in situ.

The observed excellent performance of various biomass‐derived solvents for Cu‐catalyzed carboxylations of in situ‐generated organoboranes and organoboronates prompted us to test these solvents on a range of other C−C bond‐forming reactions involving CO_2_ (Scheme 4 and Scheme S4). We began with the examination of Cu‐catalyzed hydroboration/carboxylation of phenylacetylene **5 a**,15c which was here performed in biomass‐derived ethers (Scheme 4 A). Using the conditions developed by Skrydstrup and co‐workers, but applying them in 2MeTHF instead of dioxane, we observed a mixture of benzylmalonic acid **6 a** with decarboxylative hydrocarboxylation product **2 b** in a 1:0.4 ratio. Further analysis of the reaction showed that the decarboxylation of **6 a** can be complete when the reaction is performed at 150 °C for 36 h. This improvement allowed us to obtain **2 b** as a major product in 80 % yield by using 2MeTHF. Acetal and Euc also gave good yields of **2 b** but were less effective than 2MeTHF; whereas, the yield of the reaction in dioxane (84 %) was comparable with the yield observed in 2MeTHF. Overall, decarboxylative hydrocarboxylation was not described earlier.

This was followed by examination of the hydrocarboxylation of styrenes in biomass‐derived solvents. Among others, these studies involved Fe‐catalyzed hydrocarboxylation of 4‐methylstyrene **1 a** by using EtMgBr as a stoichiometric reductant (Scheme 4 B). Notably, similar hydrocarboxylations were already reported in ether and THF.^18^ Our studies showed that for Fe‐catalyzed hydrocarboxylation of **1 a**, it is possible to apply biomass‐derived solvents. The best results were obtained by using the Fe(acac)_3_/TMEDA (tetramethylethylenediamine) system as the catalyst in 2MeTHF. In this case, the yield of **7 a** was 27 %, application of other biomass‐derived ethers did not improve the outcome of the reaction; whereas the use of THF slightly enhanced the yield of hydrocarboxylation (27 % 2MeTHF vs. 39 % THF).18c It should be emphasized that EtMgBr is now available as a 3.4 m solution in 2MeTHF, and this type of experiment can be conducted by applying exclusively biomass‐derived solvents. The hydrocarboxylation of styrenes was also examined by using different Ni‐based catalysts, which unfortunately were not successful (Scheme S4 C,D).

Further, we have explored the hydrocarboxylation of acetylenes in biomass‐derived solvents (Scheme 4 C–E).^19^ We employed diphenylacetylene **8 a** to test different catalytic systems based on Ni, Cu, and Fe. Promising results were observed when using the CuF_2_/IMesHCl/NaO*t*Bu catalytic system with triethoxysilane as a reducing agent (Scheme 4 D). The best solvent proved to be Acetal (61 %), whereas the yields of the hydrocarboxylation product **9 a** were slightly lower in 2MeTHF (41 %) and Euc (43 %). The reaction performed in dioxane (solvent used in the original work) gave **9 a** in 57 % yield.19a Hydrocarboxylation of **8 a** was also possible with the Ni(cod)_2_/CsF catalytic system and using Et_2_Zn as the reductant. In this case, the yield of **9 a** was only 21 % in 2MeTHF, whereas in MeCN (solvent used in the original work) the product was obtained in 49 % yield (Scheme 4 C).19b Among Fe‐based catalytic systems, moderate yields of **9 a** were observed with FeCl_2_ used in combination with 3.4 m EtMgBr in 2MeTHF (42 % in 2MeTHF, Scheme 4 E). Similar conditions were tested in Et_2_O (solvent used in the original work) where **9 a** was obtained in 14 % yield.19e

Next, we examined other carboxylative transformations. Excellent results were observed for the Cu‐catalyzed borocarboxylation of styrenes (Scheme 4 F).^20^ Particularly, we found that the catalytic system based on CuCl and ICyHCl (1,3‐dicyclohexylimidazolium chloride), originally developed by Popp and co‐workers,20b operates well in biomass‐derived ethers, initiating efficient borocarboxylation of **1 a**. In this case, the best solvent was Euc (85 %), but good yields of borocarboxylation product **10 a** were also observed in 2MeTHF (81 %), whereas Acetal (44 %) was far less effective. For comparison, the borocarboxylation of **1 a** performed in THF (solvent used in the original work) gave **10 a** in 78 % yield.20b

We also explored the carbocarboxylation of olefins, which is known to proceed under the influence of a wide range of catalysts based on both early and late transition metals.^21^ Screening of several catalysts derived from Zr and Ti as well as reducing agents showed that carbocarboxylation of 4‐methylstyrene **1 a** can be performed in biomass‐derived solvents (Scheme 4 G). The best results were observed when using Cp_2_ZrCl_2_ (zirconocene dichloride) as catalyst precursor combined with EtMgBr in Acetal (24 %). Application of other ethers as solvents did not improve the yield of **11 a**. Using THF under otherwise identical conditions gave **11 a** in comparable 28 % yield.21c

Similar to organoboronates, the carboxylation of organosilicon reagents can be performed in biomass‐derived solvents (Scheme 4 H).^22^ The best results were observed with triethoxyphenylsilane **12 a** when using Cu‐based catalysts. Particularly, we found that biomass‐derived ethers are not the best solvents for this reaction (2MeTHF 16 %, Acetal 0 %). The best yields of benzoic acid **4 a** were observed when using the esters GVL (42 %) and Et_2_Suc (36 %) as solvents, CuBr as catalyst precursor, and CsF as a base. In this case, the yield of **4 a** could be notably improved when running the reaction in DMA (62 %). It should be noted that the reaction does not work without the Cu catalyst.

Finally, we examined TM‐catalyzed direct C−H carboxylations.^23^, ^24^ To date, direct C−H carboxylations have been performed on azoles possessing an acidic C−H bond, arenes with appropriate directing groups,^23^ and terminal acetylenes.^24^ Our studies on phenylacetylene **5 a** indicated that Cs_2_CO_3_ alone can initiate direct C−H carboxylation in 2MeTHF, albeit with only 20 % yield of the isolated product (Scheme 4 I). The yield was improved to 31 % by switching to GVL. Further improvements were achieved by using the catalytic system developed for carboxylation of organoboranes and organoboronates. The best yields of **4 o** were observed in 2MeTHF and Acetal (76 and 63 %, respectively), whereas GVL turned out to be far less effective with the Cu catalyst (27 %). The best conditions were also tested with THF where **4 o** was obtained in 38 % yield. Unfortunately, all attempts to accomplish C−H carboxylation of azoles in biomass‐derived solvents failed (Scheme S4 A,B).

For isolation and purification of the obtained carboxylic acids, we mainly used acid–base extraction techniques. Analysis of different renewable solvents for extraction showed that Et_2_O, which is readily available from ethanol, but is not popular in industry owing to its volatility and flammability, can be replaced by renewable 2MeTHF, Acetal, diethoxymethane, and dimethoxymethane without any noticeable drop in yields. Column chromatography, with mixtures of heptane/EtOAc or Et_2_O/pentane/HCO_2_H as eluent, was only necessary for the purification of the products of decarboxylative hydrocarboxylation of phenylacetylene (Scheme 4 A).

## Conclusions

We have shown that a variety of CO_2_‐based carboxylations can be performed in biomass‐derived solvents, including a range of previously unknown solvents. The studied media included polar aprotic biomass‐derived ethers (2MeTHF, Acetal, Me_2_Isos, Cyr, Euc, RoseOx) and esters (GVL, Et_2_Suc, EtOAc), as well as nonpolar aprotic unsaturated terpenes and their derivatives (γ‐Terp, α‐Pin, Lim, Cym). Initial studies on Cu‐catalyzed carboxylation of in situ‐generated organoboranes and ‐boronates revealed that most of the biosolvents are suitable for carboxylative transformations, with biomass‐derived ethers showing the best efficiency. Our methodology was successfully applied to organoboranes generated from styrenes and internal alkenes, as well as for carboxylation of aryl‐, alkenyl‐, alkynyl‐, and benzylboronic acid pinacol esters. On the basis of the latter, we have synthesized the commercial drugs Fenoprofen and Flurbiprofen.

Biomass‐derived solvents were further applied for the hydrocarboxylation of acetylenes and styrenes, using catalysts based on Cu, Ni, or Fe. We observed moderate to good yields and excellent regioselectivities. Very good results were obtained for the Cu‐catalyzed borocarboxylation of styrenes and C−H carboxylation of phenylacetylene. Biomass‐derived ethers can also be used for the Cu‐catalyzed carboxylation of triethoxyphenylsilane and the Zr‐catalyzed carbocarboxylation of styrenes. Most of the reactions were examined in traditional organic solvents as a comparison. These studies revealed that there is no advantage in using traditional solvents for the reactions described herein. In most cases, the yields obtained in traditional solvents were comparable with those in biosolvents, whereas in some cases, biomass‐derived solvents performed even better. Biomass‐derived ethers showed the best performance, with 2MeTHF generally being superior. However, it is not a universal solvent. In several cases, excellent results were instead observed when using Me_2_Isos, Acetal, RoseOx, or Euc solvents. We believe that the biomass‐derived solvents introduced herein will find broad applications in many processes currently based on traditional organic solvents.

## Experimental Section

### General experimental procedure for Cu‐catalyzed hydroboration/carboxylation of olefins (Scheme 2)

Inside of a glovebox, a 45 mL pressure tube was charged with the appropriate olefin (1.5 mmol), (9‐BBN)_2_ (1 equiv or 0.7 equiv in the case of dienes), and the corresponding dry solvent (4 mL). The flask was closed with a suitable cap, removed from the glovebox, and heated to 70 °C for 24 h. Afterwards, the pressure tube was transferred back to the glovebox. To the reaction mixture at 20 °C was added CsF (3 equiv) and a previously prepared solution of catalyst (mixture of CuI (5 mol %), IPrHCl (6 mol %), and NaO*t*Bu (6 mol %) in appropriate dry solvent (2 mL) stirred at 20 °C for 30 min) was added. The pressure tube was closed with the cap and removed from the glovebox. Afterwards, CO_2_ (120 mL) was added via a syringe, which was followed by stirring of the reaction mixture at 120 °C for 24 h. Next, the reaction mixture was diluted with Et_2_O (30 mL) and transferred into a 500 mL separating funnel. The resulting mixture was extracted with saturated NaHCO_3_ solution (3×30 mL). The resulting basic solution was washed with Et_2_O (15 mL), acidified (50–55 mL 6 m HCl), and extracted with Et_2_O (3×30 mL). The resulting solution of Et_2_O was distilled to dryness to give the corresponding acid.

In the cases of Me_2_Isos, GVL, and Et_2_Suc, the basic solution was washed with either CH_2_Cl_2_ or Et_2_O (3×15 mL), and the final Et_2_O solution was washed with distilled water (3×15 mL) before evaporation. Other renewable solvents such as 2MeTHF, Acetal, diethoxymethane, or dimethoxymethane can replace Et_2_O without any noticeable difference (the difference was in the range ±3 %). Similarly, the saturated solution of NaHCO_3_ can be replaced by a 2 m solution of KOH.

### General experimental procedure for Cu‐catalyzed carboxylation of organoboronates (Scheme 3)

Inside of a glovebox, a 45 mL pressure tube was charged with the appropriate organoboronate (0.8 mmol), CsF (3 equiv), and corresponding dry solvent (2 mL). This was followed by addition of a previously prepared solution of the catalyst (mixture of CuI (5 mol %), IPrHCl (6 mol %), and NaO*t*Bu (6 mol %) in an appropriate dry solvent (2 mL) was stirred at 20 °C for 30 min). The pressure tube was closed with the cap and removed from the glovebox. Afterwards, CO_2_ (120 mL) was added via a syringe, which was followed by stirring of the reaction mixture at 120 °C for 24 h. Next, the reaction mixture was diluted with Et_2_O (30 mL) and transferred into a 500 mL separating funnel. The resulting mixture was extracted with saturated NaHCO_3_ solution (3×30 mL). The resulting basic solution was washed with Et_2_O (15 mL), acidified (50–55 mL 6 m HCl), and extracted with Et_2_O (3×30 mL). The resulting solution of Et_2_O was distilled to dryness to give the corresponding acid.

In the cases of Me_2_Isos, the basic solution was washed with either CH_2_Cl_2_ or Et_2_O (3×15 mL), and the final Et_2_O solution was washed with distilled water (3×10 mL) before evaporation. Other renewable solvents such as 2MeTHF, Acetal, diethoxymethane, or dimethoxymethane can replace Et_2_O without any noticeable difference (the difference was in the range ±3 %). Similarly, the saturated solution of NaHCO_3_ can be replaced by a 2 m solution of KOH.

### Abbreviations

2MeTHF=2‐methyltetrahydrofuran; 9‐BBN=9‐borabicyclo[3.3.1]nonane; Bpin=boronic acid pinacol ester; Acetal=acetaldehyde diethyl acetal; Cyr=cyrene; Cym=*p*‐cymene; DMF=dimethylformamide; DMA=dimethylacetamide; Euc=eucalyptol; Et_2_Suc=diethyl succinate; EtOAc=ethyl acetate; GVL=γ‐valerolactone; IPrHCl=1,3‐bis(2,6‐diisopropylphenyl)imidazolium chloride; I*t*BuHBF_4_=1,3‐di‐*tert*‐butylimidazolium tetrafluoroborate; IAdHCl=1,3‐bis(1‐adamantyl)imidazolium chloride; IMesHCl=1,3‐bis(2,4,6‐trimethylphenyl)imidazolium chloride; ICyHCl=1,3‐dicyclohexylimidazolium chloride; Lim=(*R*)‐(+)‐limonene; LCA=life‐cycle assessment; Me_2_Isos=isosorbide dimethyl ether; α‐Pin=α‐pinene; RoseOx=(+)‐rose oxide; γ‐Terp=γ‐terpinene; THF=tetrahydrofuran; TMEDA=tetramethylethylenediamine.

## Conflict of interest

*The authors declare no conflict of interest*.

## Supporting information

As a service to our authors and readers, this journal provides supporting information supplied by the authors. Such materials are peer reviewed and may be re‐organized for online delivery, but are not copy‐edited or typeset. Technical support issues arising from supporting information (other than missing files) should be addressed to the authors.

Supplementary
