# Supplementary material for: Exploration of New Biomass‐Derived Solvents: Application to Carboxylation Reactions
Source: ChemSusChem. 2020 Feb 11;13(8):2080–8. doi: 10.1002/cssc.201903224 (PMC7217053; doi:10.1002/cssc.201903224)

ChemSusChem

Supporting Information

Exploration of New Biomass-Derived Solvents: Application to Carboxylation Reactions

Ashot Gevorgyan,*[a] Kathrin H. Hopmann,[b] and Annette Bayer*[a]

Supplementary Information
Exploration of New Biomass-Derived Solvents: Application to Carboxylation Reactions

Ashot Gevorgyan,*[,a] Kathrin H. Hopmann,[b] and Annette Bayer*[,a]

[a] Department of Chemistry, UiT The Arctic University of Norway, 9037 Tromsø, Norway.
[b] Hylleraas Centre for Quantum Molecular Sciences, Department of Chemistry, UiT The Arctic University of Norway, 9037 Tromsø, Norway.
E-mail: ashot.gevorgyan@uit.no; annette.bayer@uit.no

Table of content:

General considerations 2
Optimization of reaction. 3
Table S1. Screening of catalysts 5
Table S2. Screening of duration and temperature 6
Table S3. Screening of bases 7
Table S4. Screening of solvents 8
Table S5. Screening of solvents for phenylboronic acid pinacol ester 9
Table S6. Optimization of Cu-free hydrocarboxylation of *trans*-stilbene 10
Prices of biomass-derived solvents *vs* common organic solvents 11
Starting materials used in the study 12
Unsuccessful experiments 13
Setup of the reaction 14
General procedures 18
Characterization of products 28
Copies of spectra 40

General considerations

Commercially available starting materials, reagents, catalysts and anhydrous and degassed solvents were used without further purification. Flash column chromatography was performed with Merck silica gel 60 (230-400 mesh). The solvents for column chromatography were distilled before use (in case of technical solvents). Thin layer chromatography was carried out using Merck TLC Silica gel 60 F_{254} and visualized by short-wavelength ultraviolet light or by treatment with potassium permanganate ($KmnO_4$) stain. ^1H, ^{13}C and ^{19}F NMR spectra were recorded on a Bruker Avance 400 MHz at 20ºC. All ^1H NMR spectra are reported in parts per million (ppm) downfield of TMS and were measured relative to the signals for $CHCl_3$ (7.26 ppm), methanol (4.87 ppm, 3.31 ppm) and DMSO (2.50 ppm). All ^{13}C NMR spectra were reported in ppm relative to residual $CDCl_3$ (77.20 ppm), methanol (49.1 ppm) or DMSO (39.70 ppm) and were obtained with ^1H decoupling. Coupling constants, *J*, are reported in Hertz (Hz). High-resolution mass spectra (HRMS) were recorded from methanol solutions on an LTQ Orbitrap XL (Thermo Scientific) either in negative or in positive electrospray ionization (ESI) mode. Melting points were measured using Stuart SMP50 automatic melting point detector.

Solvents used in the work are commercially available. 2MeTHF (anhydrous, ≥99%, inhibitor-free, Sigma-Aldrich 673277-1L) was bought as anhydrous solvent equipped with a septa. Other biomass-derived solvents were reagent grade; they were degassed and kept over activated molecular sieves (4 Å) at least for a week before use. The purity of the solvents used in the work were as follows: Acetal (99%, inhibitor-free, Sigma-Aldrich A902-500ML); Me_2Isos (98%, inhibitor-free, Sigma-Aldrich 247898-100G); GVL (99%, Sigma-Aldrich V403-500G); Cyr (not specified, inhibitor-free, Sigma-Aldrich 807796-100ML); Et_2Suc (natural, ≥99%, Sigma-Aldrich W237712-100G-K); EtOAc (≥99.5%, VWR 23882.321); γ-Terp (97%, Sigma-Aldrich 223190-100ML); α-Pin (98%, Sigma-Aldrich 147524-250ML); Lim (97%, Sigma-Aldrich 183164-100ML); Cym (99%, Sigma-Aldrich C121452-1L); Euc (99%, inhibitor-free, Sigma-Aldrich C80601-500ML); RoseOx (97%, inhibitor-free, Chemtronica/TCI M2363-25G).

All solvents were degassed and stored under Ar atmosphere. 2MeTHF, Acetal, Me_2Isos, Cyr, Euc and RoseOx are ethers and may form peroxides when stored under air; however, peroxide tests (Test strips for peroxide, MQuant®, Supelco®, VWR/Merck 1.10081.0001) of freshly bought solvents did not show any noticeable levels of peroxides. Acetal can be hydrolyzed in the presence of strong acids when heated. Under basic conditions, which are frequently used for the reactions involving organometallics, acetal is stable. GVL, Et_2Suc and EtOAc can be hydrolyzed in the presence of strongly basic water solutions; under anhydrous conditions, they are stable. Except Cyr, other solvents proved to be quite stable under various conditions used in the work.

Warning! Most of the reactions were performed in specialized glassware under pressure. The glassware should always be examined for damages before any manipulation. All laboratory safety procedures must be followed strictly and the work with pressure tubes must be conducted behind a shield.

Optimization of reaction.

General experimental procedure for hydrocarboxylation of 4-methylstyrene. For general setup, see Figure S1-S4.

Inside of glove box 45 mL pressure tube was charged with 4-methylstyrene (0.846 mmol, 100 mg), $(9\text{-BBN})_2$ (1 equiv.) and corresponding dry solvent (3 mL). The flask was closed with suitable cap, removed from the glove box and heated to 70°C for 24h. Afterwards, the pressure tube was transferred back to the glove box. To the reaction mixture at 20°C was added the base (0-3 equiv.) and (in case of Cu-catalyzed experiments) previously prepared solution of catalyst (the mixture of transition metal (5 mol%), ligand (6 mol%) and NaO*t*Bu (0-6 mol%) in appropriate dry solvent (1 mL) was stirred at 20°C for 30 min). The pressure tube was closed with the cap and removed from the glove box. Afterwards CO_2 (120 mL) was added *via* a syringe, which was followed by stirring of reaction mixture at 80-120°C for 6-28h. Next, the reaction mixture was diluted with 15 mL Et_2O and transferred into 250 mL separating funnel. The resulting mixture was extracted with 15 mL saturated $NaHCO_3$ solution (3 times). The resulting basic solution was washed with 10 mL Et_2O (once), acidified (25-30 mL 6M HCl) and extracted with 15 mL Et_2O (3 times). The resulting solution of Et_2O was distilled to dryness to give corresponding acid.

In cases of Me_2Isos, GVL and Et_2Suc the basic solution was washed with either DCM or Et_2O (15 mL, 3 times), and the final Et_2O solution was washed with 10 mL distilled water (3 times) before evaporation.

Other renewable solvents like 2MeTHF, Acetal, diethoxymethane or dimethoxymethane without any noticeable difference can replace Et_2O (the difference was in the range ±3%). Similarly, saturated solution of $NaHCO_3$ can be replaced by 2M solution of KOH.

General experimental procedure for Cu-catalyzed carboxylation of phenylboronic acid pinacol ester (Table S5). For general setup, see Figure S1-S4.

Inside of glove box 45 mL pressure tube was charged with phenylboronic acid pinacol ester (0.735 mmol, 150 mg), CsF (3 equiv.) and corresponding dry solvent (2 mL). This was followed by addition of previously prepared solution of the catalyst (the mixture of CuI (5 mol%), IprHCl (6 mol%) and NaO*t*Bu (6 mol%) in appropriate dry solvent (2 mL) was stirred at 20°C for 30 min). The pressure tube was closed with the cap and removed from the glove box. Afterwards CO_2 (120 mL) was added *via* a syringe, which was followed by stirring of reaction mixture at 120°C for 24h. Next, the reaction mixture was diluted with 15 mL Et_2O and transferred into 250 mL separating funnel. The resulting mixture was extracted with 15 mL saturated $NaHCO_3$ solution (3 times). The resulting basic solution was washed with 10 mL Et_2O (once), acidified (25-30 mL 6M HCl) and extracted with 15 mL Et_2O (3 times). The resulting solution of Et_2O was distilled to dryness to give corresponding acid.

In cases of Me_2Isos, GVL and Et_2Suc the basic solution was washed with either DCM or Et_2O (15 mL, 3 times), and the final Et_2O solution was washed with 10 mL distilled water (3 times) before evaporation.

Other renewable solvents like 2MeTHF, Acetal, diethoxymethane or dimethoxymethane without any noticeable difference can replace Et_2O (the difference was in the range ±3%). Similarly, saturated solution of $NaHCO_3$ can be replaced by 2M solution of KOH.

General experimental procedure for Cu-catalyzed/Cu-free hydrocarboxylation of *trans*-stilbene (Table S6). For general setup, see Figure S1-S4.

Inside of glove box 45 mL pressure tube was charged with *trans*-stilbene (0.554 mmol, 100 mg), $(9\text{-BBN})_2$ (1 equiv.) and corresponding dry solvent (3 mL (Cu-catalyzed), 4 mL (Cu-free)). The flask was closed with suitable cap, removed from the glove box and heated to 70°C for 24h. Afterwards, the pressure tube was transferred back to the glove box. To the reaction mixture at 20°C was added the base (0-3 equiv.) and (in case of Cu-catalyzed experiments) previously prepared solution of catalyst (the mixture of CuI (5 mol%), IprHCl (6 mol%) and NaO*t*Bu (6 mol%) in appropriate dry solvent (1 mL) was stirred at 20°C for 30 min). The pressure tube was closed with the cap and removed from the glove box. Afterwards CO_2 (120 mL) was added *via* a syringe, which was followed by stirring of reaction mixture at 80-120°C for 6-24h. Next, the reaction mixture was diluted with 15 mL Et_2O and transferred into 250 mL separating funnel. The resulting mixture was extracted with 15 mL saturated $NaHCO_3$ solution (3 times). The resulting basic solution was washed with 10 mL Et_2O (once), acidified (25-30 mL 6M HCl) and extracted with 15 mL Et_2O (3 times). The resulting solution of Et_2O was distilled to dryness to give corresponding acid.

In cases of Me_2Isos the basic solution was washed with either DCM or Et_2O (15 mL, 3 times), and the final Et_2O solution was washed with 10 mL distilled water (3 times) before evaporation.

Other renewable solvents like 2MeTHF, Acetal, diethoxymethane or dimethoxymethane without any noticeable difference can replace Et_2O (the difference was in the range ±3%). Similarly, saturated solution of $NaHCO_3$ can be replaced by 2M solution of KOH.

Table S1. Screening of catalysts

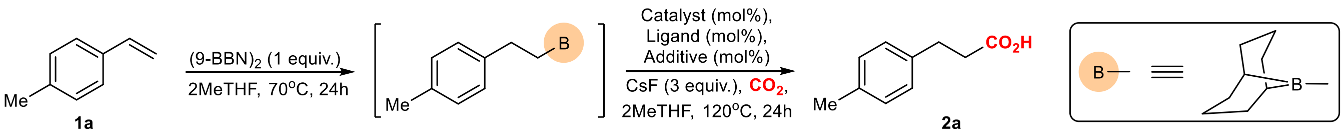

Entry	Catalyst (mol%)	Ligand (mol%)	Additive (mol%)	Yield of 2a %[a,b]
1	-	-	-	0
**2**	**CuI (5)**	**IPrHCl (6)**[c]	**NaO*t*Bu (6)**	**98**
3	CuBr (5)	IPrHCl (6)	NaO*t*Bu (6)	93
4	CuCl (5)	IPrHCl (6)	NaO*t*Bu (6)	82
5	$Cu(OAc)_2$ (5)	IPrHCl (6)	NaO*t*Bu (6)	77
6	Ag_2CO_3 (5)	IPrHCl (6)	NaO*t*Bu (6)	7
7	CuI (5)	I*t*BuHBF_4 (6)[d]	NaO*t*Bu (6)	68
8	CuI (5)	IAdHCl (6)[e]	NaO*t*Bu (6)	84
9	CuI (5)	IMesHCl (6)[f]	NaO*t*Bu (6)	26
10	CuI (5)	1,10-phen (6)[g]	NaO*t*Bu (6)	0
11	CuI (3)	IPrHCl (4)[c]	NaO*t*Bu (4)	84

[a] Reaction conditions: 1) **1a** (0.846 mmol), $(9\text{-BBN})_2$ (1 equiv.), 2MeTHF (3 mL), 70°C, 24h. 2) Transition metal (0-5 mol%), ligand (0-6 mol%), NaO*t*Bu (0-6 mol%), 2MeTHF (1 mL), CsF (3 equiv.), CO_2 (120 mL), 120°C, 24h. [b] Isolated yields. [c] IPrHCl = 1,3-bis(2,6-diisopropylphenyl)imidazolium chloride. [d] I*t*BuHBF_4 = 1,3-di-*tert*-butylimidazolium tetrafluoroborate. [e] IAdHCl = 1,3-bis(1-adamantyl)imidazolium chloride. [f] IMesHCl = 1,3-bis(2,4,6-trimethylphenyl)imidazolium chloride. [g] 1,10-phen = 1,10-phenanthroline.

Table S2. Screening of duration and temperature

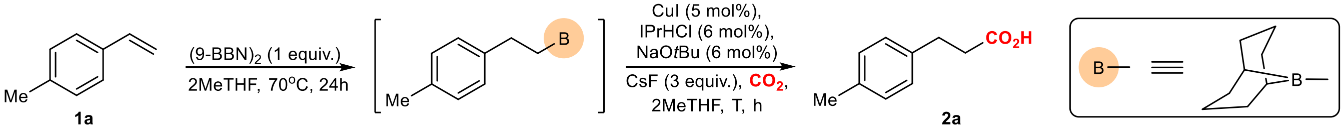

Entry	T, ºC	h	Yield of 2a %[a,b]
**1**	**120**	**24**	**98**
2	80	24	80
3	120	6	66
4	120	16	96
5	120	28	98

[a] Reaction conditions: 1) **1a** (0.846 mmol), (9-BBN)$_2$ (1 equiv.), 2MeTHF (3 mL), 70ºC, 24h. 2) CuI (5 mol%), IPrHCl (6 mol%), NaO*t*Bu (6 mol%), 2MeTHF (1 mL), CsF (3 equiv.), CO_2 (120 mL), 80-120ºC, 6-28h. [b] Isolated yields.

Table S3. Screening of bases

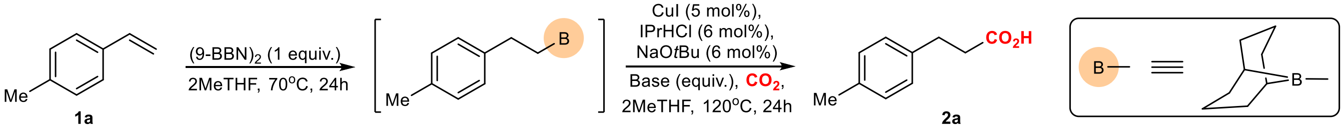

**Entry**	**Base (equiv.)**	**Yield of 2a %**[a,b]
**1**	**CsF (3)**	**98**
2	CsF (2)	73
3	KF (3)	61
4	NaF (3)	0
5	KO*t*Bu (3)[c]	75
**6**	**Cs_2CO_3 (3)**	**95**
7	Rb_2CO_3 (3)	34
8	K_2CO_3 (3)	37
9	CsOAc (3)	20
10	K_3PO_4 (3)	0
11	-	0

[a] Reaction conditions: 1) **1a** (0.846 mmol), $(9\text{-BBN})_2$ (1 equiv.), 2MeTHF (3 mL), 70°C, 24h. 2) CuI (5 mol%), IPrHCl (6 mol%), NaO*t*Bu (6 mol%), 2MeTHF (1 mL), base (0-3 equiv.), CO_2 (120 mL), 120°C, 24h. [b] Isolated yields. [c] Before addition of CO_2 the reaction mixture, containing KO*t*Bu, was mixed at 20°C for 30 min.

Table S4. Screening of solvents

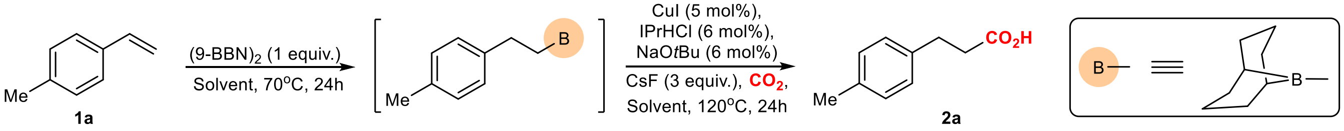

Entry	Solvent	Yield of 2a %[a,b]
**1**	**2MeTHF**[c]	**98**
2	EtOAc[c]	59
3	GVL[c]	0
4	γ-Terp[c]	32
5	Cym[c]	55
6	Lim[c]	0
7	α-Pin[c]	0
8	Cyr[c]	0
**9**	**Euc**[c]	**94**
10	RoseOx[c]	52
**11**	**Acetal**[c]	**91**
12	Et_2Suc[c]	53
**13**	**Me_2Isos**[c]	**92**
14	THF	89
15	Dioxane	84
16	Toluene	85
17	DMF	0

[a] Reaction conditions: 1) **1a** (0.846 mmol), $(9\text{-BBN})_2$ (1 equiv.), Solvent (3 mL), 70°C, 24h. 2) CuI (5 mol%), IPrHCl (6 mol%), NaO*t*Bu (6 mol%), Solvent (1 mL), CsF (3 equiv.), CO_2 (120 mL), 120°C, 24h. [b] Isolated yields. [c] For full names and structure see Scheme S1.

Table S5. Screening of solvents for phenylboronic acid pinacol ester

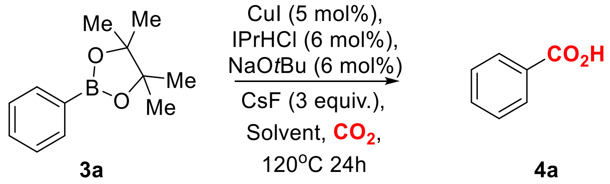

Entry	Solvent	Yield of 4a %[a,b]
1	γ-Terp[c]	20
2	Cym[c]	38
3	Lim[c]	43
4	α-Pin[c]	0
**5**	**2MeTHF[c]**	**74**
6	Euc[c]	32
**7**	**RoseOx[c]**	**73**
8	Acetal[c]	51
**9**	**Me_2Isos[c]**	**85**
10	GVL[c]	67
11	Cyr[c]	0
12	Et_2Suc[c]	58
13	EtOAc[c]	71
14	THF	78
15	Dioxane	76
16	Toluene	22
17	DMF	65

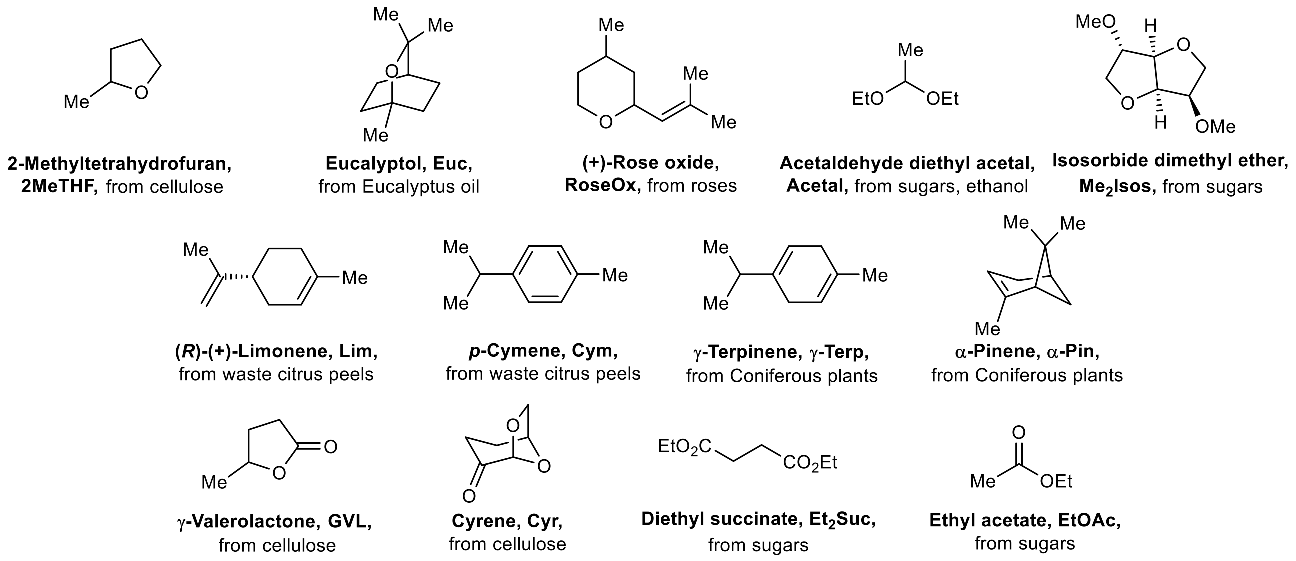

Scheme S1. Structure of solvents with suitable abbreviations.

[a] Reaction conditions: **3a** (0.735 mmol), Solvent (2 mL), CsF (3 equiv.), CuI (5 mol%), IPrHCl (6 mol%), NaO*t*Bu (6 mol%), Solvent (2 mL), CO_2 (120 mL), 120°C, 24h. [b] Isolated yields. [c] For full names and structure see Scheme S1.

Table S6. Optimization of Cu-free hydrocarboxylation of *trans*-stilbene

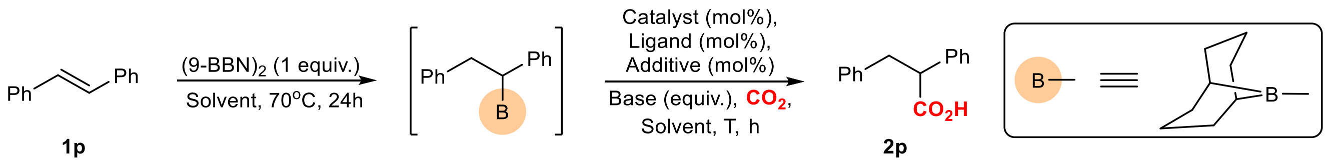

Entry	Catalyst (mol%)	Ligand (mol%)	Additive (mol%)	Base (equiv.)	Solvent	T, °C	h	Yield of 2p %[a,b]
**1**	**CuI (5)**	**IPrHCl (6)[c]**	**NaO*t*Bu (6)**	**CsF (3)**	**2MeTHF[e]**	**120**	**24**	**83**
2	CuI (5)	IPrHCl (6)	NaO*t*Bu (6)	KF (3)	2MeTHF[e]	120	24	59
3	CuI (5)	IPrHCl (6)	NaO*t*Bu (6)	CsF (3)	2MeTHF[e]	80	24	44
4	CuI (5)	IPrHCl (6)	NaO*t*Bu (6)	CsF (3)	2MeTHF[e]	120	6	47
**5**	**-**	**-**	**-**	**CsF (3)**	**2MeTHF[e]**	**120**	**24**	**81**
6	-	-	-	CsF (3)	Euc[e]	120	24	65
7	-	-	-	CsF (3)	Acetal[e]	120	24	69
8	-	-	-	CsF (3)	Me_2Isos[e]	120	24	65
9	-	-	-	CsF (3)	THF	120	24	61
10	-	-	-	CsF (3)	Dioxane	120	24	83
11	-	-	-	KF (3)	2MeTHF[e]	120	24	29
12	-	-	-	NaF (3)	2MeTHF[e]	120	24	0
13	-	-	-	KO*t*Bu (3)[d]	2MeTHF[e]	120	24	49
14	-	-	-	Cs_2CO_3 (3)	2MeTHF[e]	120	24	71
15	-	-	-	-	2MeTHF[e]	120	24	0

[a] Reaction conditions: 1) **1p** (0.554 mmol), $(9\text{-BBN})_2$ (1 equiv.), Solvent (3-4 mL), 70°C, 24h. 2) (CuI (5 mol%), IPrHCl (6 mol%), NaO*t*Bu (6 mol%), Solvent (1 mL)), base (0-3 equiv.), CO_2 120 mL, 80-120°C, 6-24h. [b] Isolated yields. [c] IPrHCl = 1,3-bis(2,6-diisopropylphenyl)imidazolium chloride. [d] Before addition of CO_2 the reaction mixture, containing KO*t*Bu, was mixed at 20°C for 30 min. [e] For full names and structure see Scheme S1.

Prices of biomass-derived solvents *vs* common organic solvents

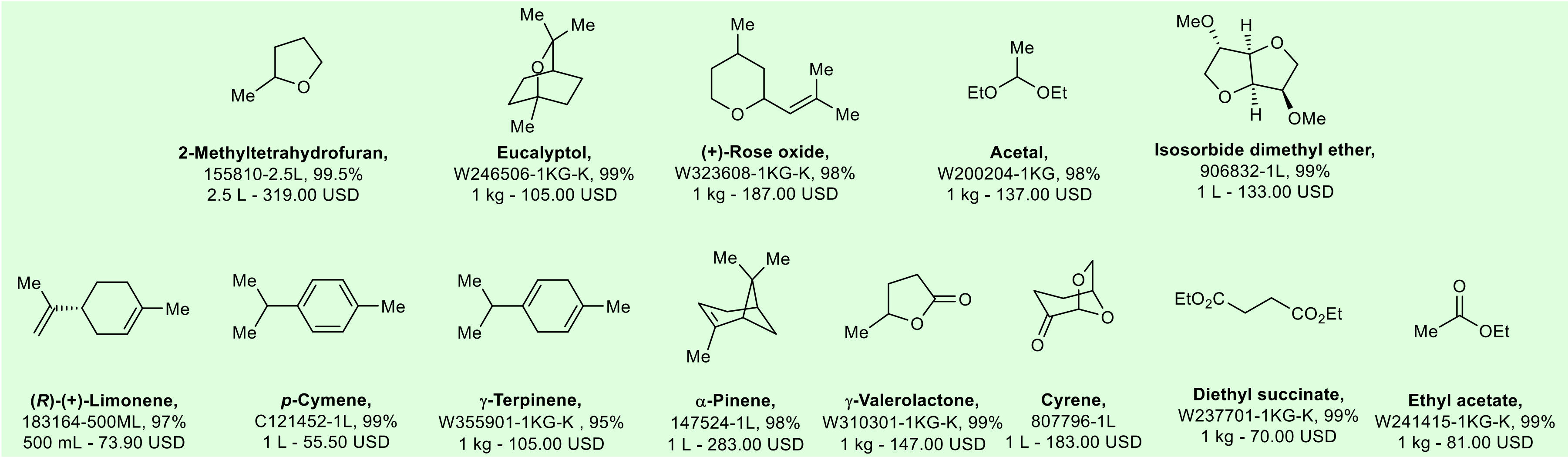

2-Methyltetrahydrofuran, 155810-2.5L, 99.5% 2.5 L - 319.00 USD

Eucalyptol, W246506-1KG-K, 99% 1 kg - 105.00 USD

(+)-Rose oxide, W323608-1KG-K, 98% 1 kg - 187.00 USD

Acetal, W200204-1KG, 98% 1 kg - 137.00 USD

Isosorbide dimethyl ether, 906832-1L, 99% 1 L - 133.00 USD

(*R*)-(+)-Limonene, 183164-500ML, 97% 500 mL - 73.90 USD

***p*-Cymene,** C121452-1L, 99% 1 L - 55.50 USD

γ-Terpinene, W355901-1KG-K , 95% 1 kg - 105.00 USD

α-Pinene, 147524-1L, 98% 1 L - 283.00 USD

γ-Valerolactone, W310301-1KG-K, 99% 1 kg - 147.00 USD

Cyrene, 807796-1L 1 L - 183.00 USD

Diethyl succinate, W237701-1KG-K, 99% 1 kg - 70.00 USD

Ethyl acetate, W241415-1KG-K, 99% 1 kg - 81.00 USD

Toluene 179418-1L, 99.5 % 1 L - 65.10 USD

Hexane 32293-1L, 99% 1 L - 127.00 USD

Tetrahydrofuran 360589-1L, 99% 1 L - 114.00 USD

1,4-Dioxane 360481-1L, 99% 1 L - 115.00 USD

Acetonitrile 360457-1L, 99.5% 1 L - 127.00 USD

***N,N*-Dimethylformamide** 319937-1L, 99.8% 1 L - 124.00 USD

***N,N'*-Dimethylethyleneurea** 40727-1L, 99% 1 L - 284.00 USD

Scheme S2. Prices of biomass-derived solvents *vs* common organic solvents. The prices were obtained from official website of Sigma Aldrich for USA at 04.09.2019.

Starting materials used in the study

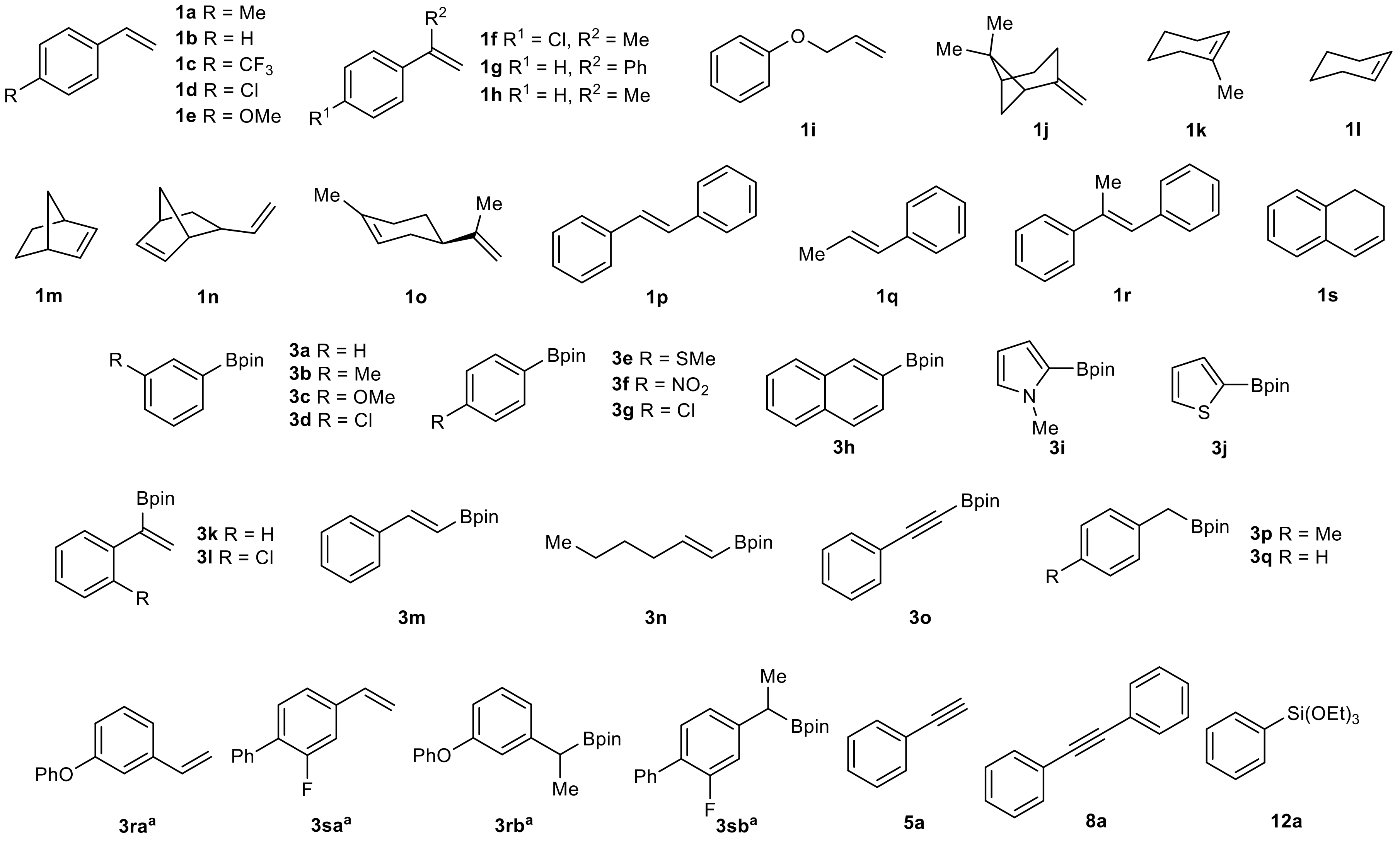

Scheme S3. List of starting materials used in the work. [a] These compounds were synthesized, while others are commercially available.

Unsuccessful experiments

(**A**) Hu et al. *Org. Lett.* **2010**, *12*, 3567-3569. Solvent-DMF

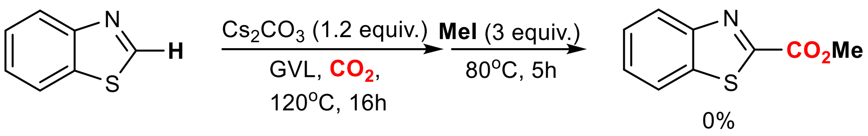

(**B**) Hou et al. *Angew. Chem. Int. Ed.* **2010**, *49*, 8670-8673. Solvent-THF

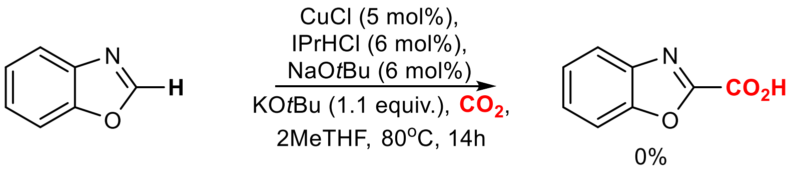

(**C**) Rovis et al. *J. Am. Chem. Soc.* **2008**, *130*, 14936-14937. Solvent-THF

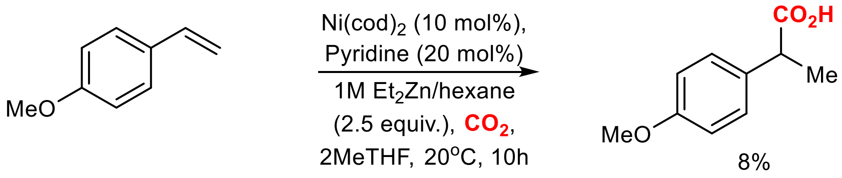

(**D**) Rovis et al. *J. Am. Chem. Soc.* **2008**, *130*, 14936-14937. Solvent-THF

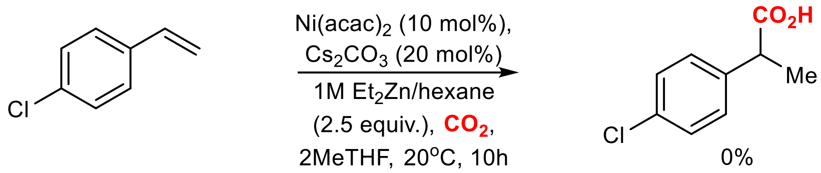

(**E**) Martin et al. *J. Am. Chrm. Soc.* **2009**, *131*, 15974-15975. Solvent-DMA/Hexane

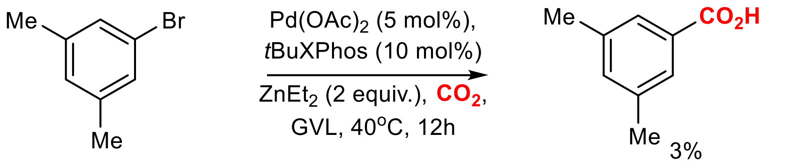

(**F**) Yu, He et al. *ChemCatChem* **2015**, *7*, 3972-3977. Solvent-DMF

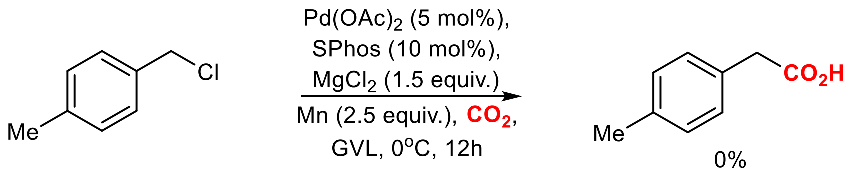

(**G**) Martin et al. *J. Am. Chem. Soc.* **2013**, *135*, 1221-1224. Solvent-DMF

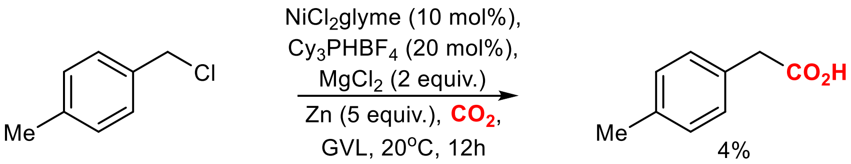

(**H**) Schomaker et al. *Chem. Eur. J.* **2012**, *18*, 9391-9396. Solvent-THF

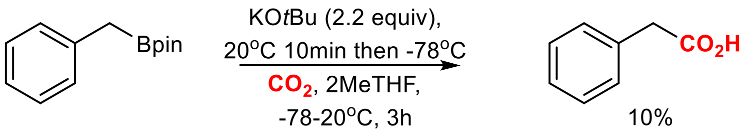

Scheme S4. Unsuccessful experiments.

Setup of the reaction

Figure S1. 45mL pressure tube with suitable stabilizer and septa.

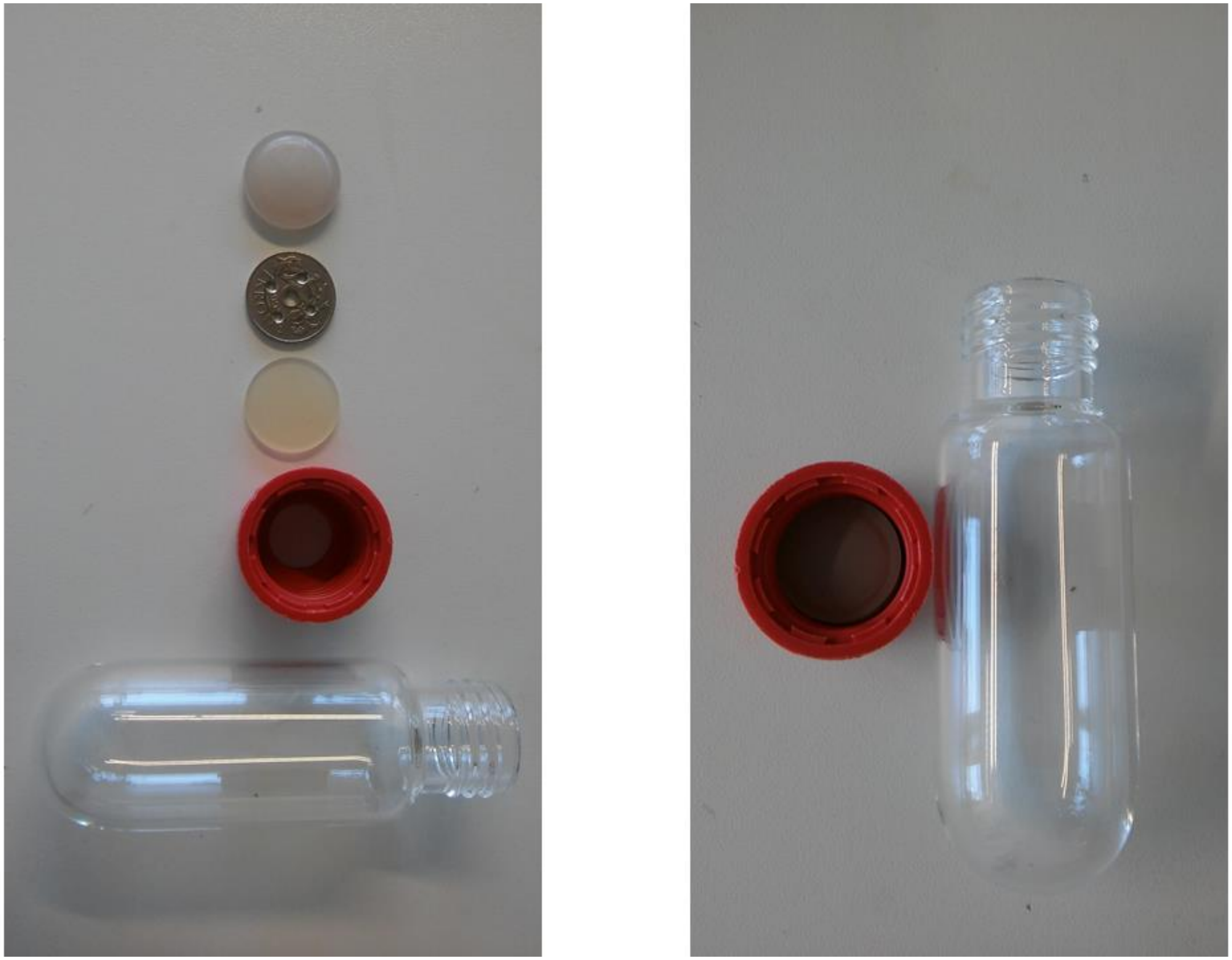

Figure S2. 45mL pressure tube with suitable stabilizer and septa.

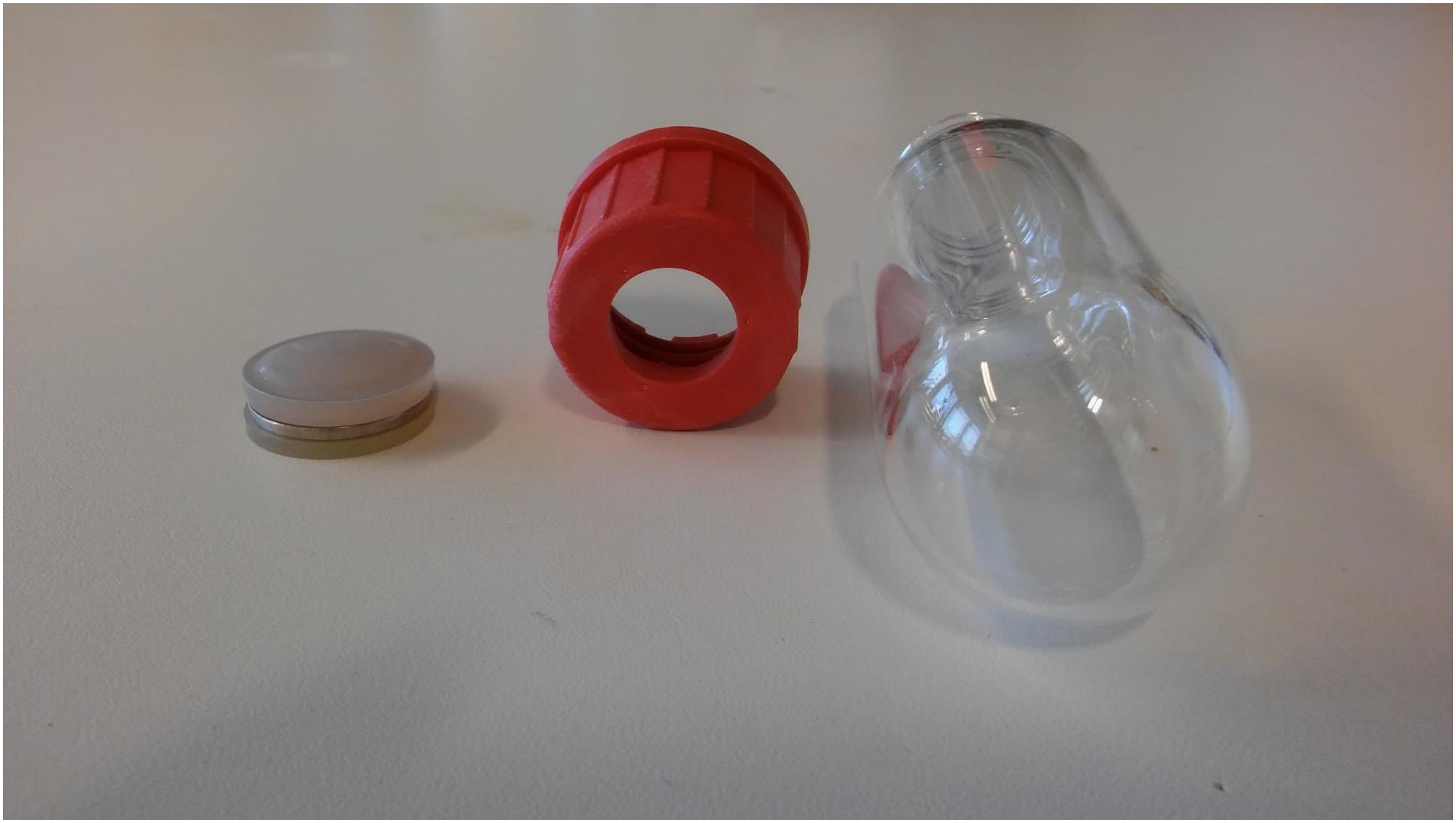

Figure S3. Syringe with CO_2 ready for injection.

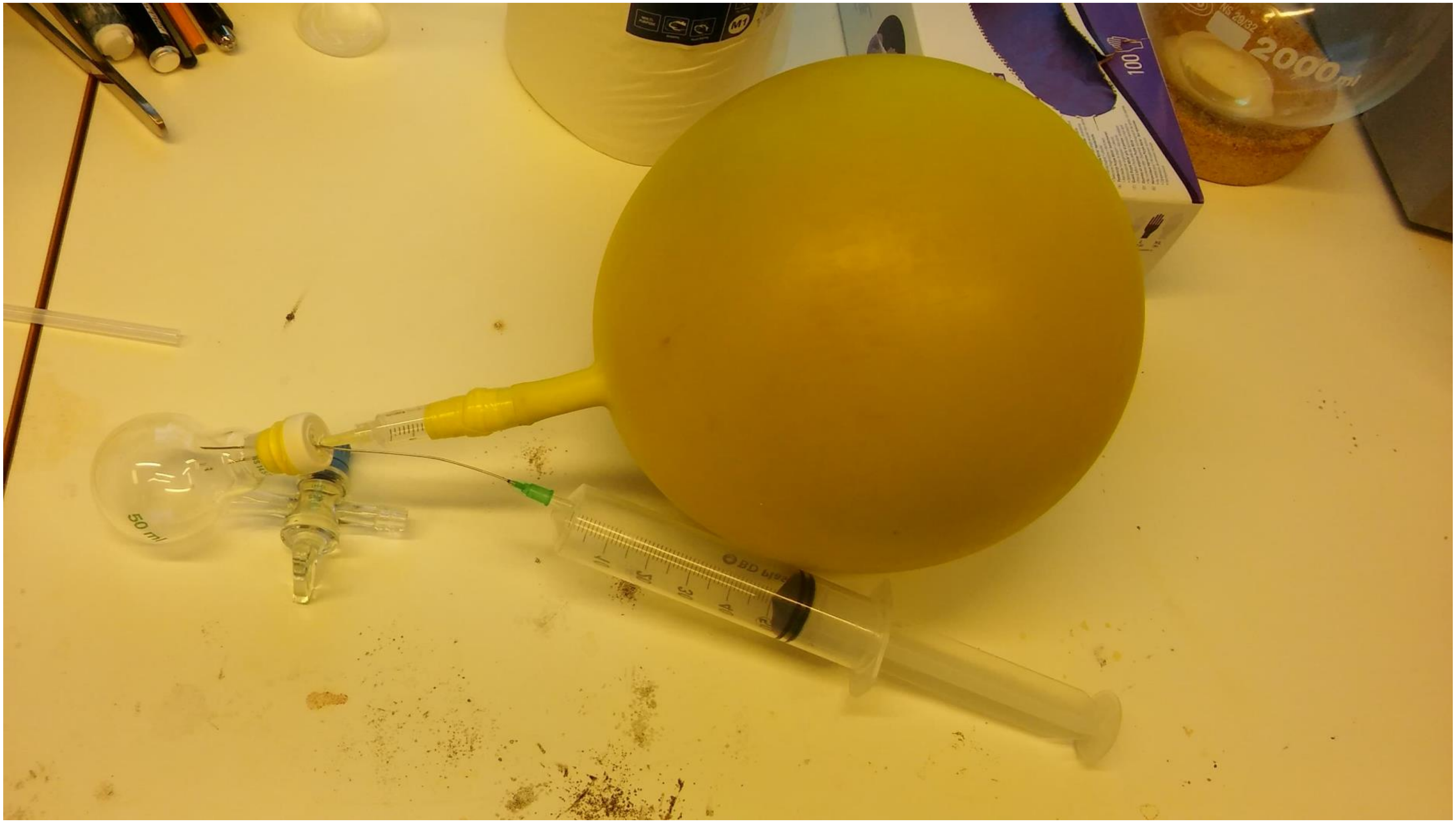

Figure S4. First injection of CO_2.

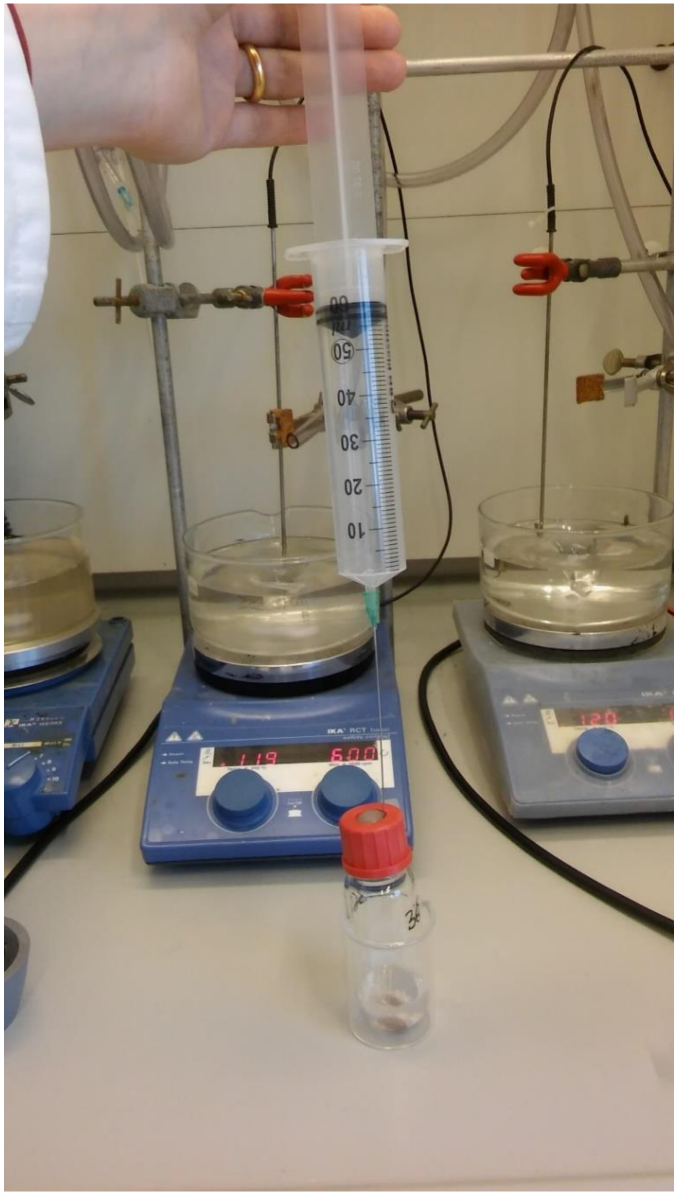

General procedures

Preparation of starting materials **3ra** and **3sa** by Wittig reaction.

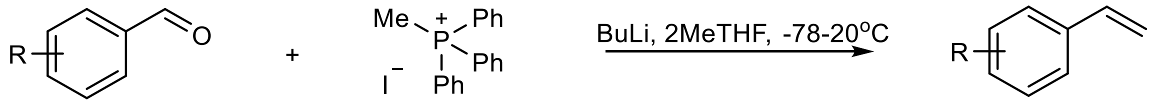

50mL round bottom flask was charged with phosphonium salt (1.2 equiv.), sealed with a rubber septa, evacuated and back filled with Ar. Afterwards, an Ar balloon was added to the system followed by addition of dry 2MeTHF (15 mL). The reaction mixture was transferred into isopropanol bath (-78°C), which was followed by addition of BuLi (2.5M in hexanes, 1.2 equiv., dropped). The reaction mixture was allowed to reach 20°C. Next, it was transferred back to the isopropanol bath (-78°C), which was followed by addition of carbonyl compound (8 mmol, solid carbonyl compounds were dissolved in 5 mL dry 2MeTHF before addition). Further, the reaction mixture was allowed to reach 20°C where it was stirred for 12h. Formed mixture was evaporated to dryness and purified by column chromatography.

Preparation of starting materials **3rb** and **3sb** by Cu-catalyzed hydroboration.

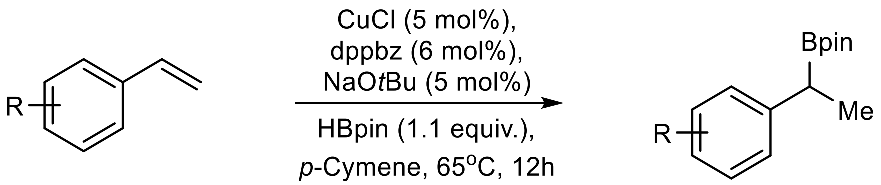

Inside of glove box 25 mL tube was charged with CuCl (5 mol%), dppbz (6 mol%) and NaO*t*Bu (5 mol%). Then it was sealed with a rubber septa, removed from the glove box and equipped with an Ar balloon. Next, dry *p*-cymene (5 mL) was added *via* a syringe, which was followed by stirring of the reaction mixture at 20°C for 10 min. This was followed by addition of HBpin (1.1 equiv.) and stirring of the reaction mixture at 20°C for another 10 min. Eventually, corresponding styrene (2.520 mmol, dissolved in 2 mL dry *p*-cymene) was added *via* a syringe and the reaction mixture was stirred at 65°C for 12h. The product was purified by column chromatography.

General experimental procedure for Cu-catalyzed hydrocarboxylation of olefins. For general setup, see Figure S1-S4.

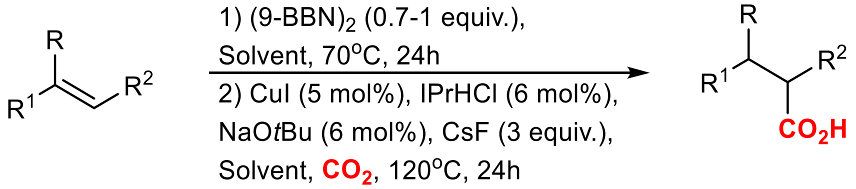

Inside of glove box 45 mL pressure tube was charged with appropriate olefin (1.5 mmol), $(9\text{-BBN})_2$ (1 equiv. (0.7 equiv in case of dienes)) and corresponding dry solvent (4 mL). The flask was closed with suitable cap, removed from the glove box and heated to 70°C for 24h. Afterwards, the pressure tube was transferred back to the glove box. To the reaction mixture at 20°C was added CsF (3 equiv.) and previously prepared solution of catalyst (the mixture of CuI (5 mol%), IPrHCl (6 mol%) and NaO*t*Bu (6 mol%) in appropriate dry solvent (2 mL) was stirred at 20°C for 30 min). The pressure tube was closed with the cap and removed from the glove box. Afterwards CO_2 (120 mL) was added *via* a syringe, which was followed by stirring of reaction mixture at 120°C for 24h. Next, the reaction mixture was diluted with 30 mL Et_2O and transferred into 500 mL separating funnel. The resulting mixture was extracted with 30 mL saturated $NaHCO_3$ solution (3 times). The resulting basic solution was washed with 15 mL Et_2O (once), acidified (50-55 mL 6M HCl) and extracted with 30 mL Et_2O (3 times). The resulting solution of Et_2O was distilled to dryness to give corresponding acid.

In cases of Me_2Isos, GVL and Et_2Suc the basic solution was washed with either DCM or Et_2O (15 mL, 3 times), and the final Et_2O solution was washed with 15 mL distilled water (3 times) before evaporation.

Other renewable solvents like 2MeTHF, Acetal, diethoxymethane or dimethoxymethane without any noticeable difference can replace Et_2O (the difference was in the range ±3%). Similarly, saturated solution of $NaHCO_3$ can be replaced by 2M solution of KOH.

General experimental procedure for Cu-free hydrocarboxylation of stilbenes and beta-substituted styrenes. For general setup, see Figure S1-S4.

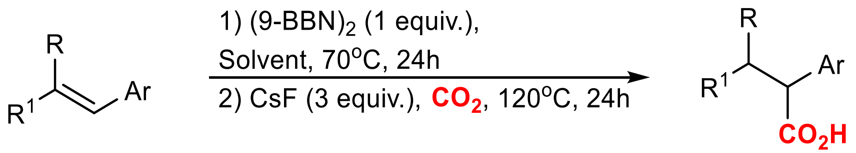

Inside of glove box 45 mL pressure tube was charged with appropriate stilbene or beta-substituted styrene (1.5 mmol), $(9\text{-BBN})_2$ (1 equiv.) and corresponding dry solvent (6 mL). The flask was closed with suitable cap, removed from the glove box and heated to 70°C for 24h. Afterwards, the pressure tube was transferred back to the glove box. To the reaction mixture at 20°C was added CsF (3 equiv.). The pressure tube was closed with the cap and removed from the glove box. Afterwards CO_2 (120 mL) was added *via* a syringe, which was followed by stirring of reaction mixture at 120°C for 24h. Next, the reaction mixture was diluted with 30 mL Et_2O and transferred into 500 mL separating funnel. The resulting mixture was extracted with 30 mL saturated $NaHCO_3$ solution (3 times). The resulting basic solution was washed with 15 mL Et_2O (once), acidified (50-55 mL 6M HCl) and extracted with 30 mL Et_2O (3 times). The resulting solution of Et_2O was distilled to dryness to give corresponding acid.

In cases of Me_2Isos the basic solution was washed with either DCM or Et_2O (15 mL, 3 times), and the final Et_2O solution was washed with 15 mL distilled water (3 times) before evaporation.

Other renewable solvents like 2MeTHF, Acetal, diethoxymethane or dimethoxymethane without any noticeable difference can replace Et_2O (the difference was in the range ±3%). Similarly, saturated solution of $NaHCO_3$ can be replaced by 2M solution of KOH.

General experimental procedure for Cu-catalyzed carboxylation of organoboronates. For general setup, see Figure S1-S4.

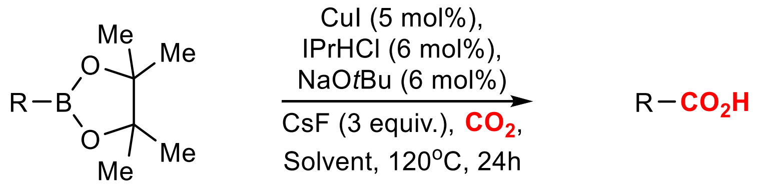

Inside of glove box 45 mL pressure tube was charged with appropriate organoboronate (0.8 mmol), CsF (3 equiv.) and corresponding dry solvent (2 mL). This was followed by addition of previously prepared solution of the catalyst (the mixture of CuI (5 mol%), IPrHCl (6 mol%) and NaO*t*Bu (6 mol%) in appropriate dry solvent (2 mL) was stirred at 20°C for 30 min). The pressure tube was closed with the cap and removed from the glove box. Afterwards CO_2 (120 mL) was added *via* a syringe, which was followed by stirring of reaction mixture at 120°C for 24h. Next, the reaction mixture was diluted with 30 mL Et_2O and transferred into 500 mL separating funnel. The resulting mixture was extracted with 30 mL saturated $NaHCO_3$ solution (3 times). The resulting basic solution was washed with 15 mL Et_2O (once), acidified (50-55 mL 6M HCl) and extracted with 30 mL Et_2O (3 times). The resulting solution of Et_2O was distilled to dryness to give corresponding acid.
In cases of Me_2Isos the basic solution was washed with either DCM or Et_2O (15 mL, 3 times), and the final Et_2O solution was washed with 10 mL distilled water (3 times) before evaporation.
Other renewable solvents like 2MeTHF, Acetal, diethoxymethane or dimethoxymethane without any noticeable difference can replace Et_2O (the difference was in the range ±3%). Similarly, saturated solution of $NaHCO_3$ can be replaced by 2M solution of KOH.

General experimental procedure for Cu-catalyzed decarboxylative hydrocarboxylation of phenylacetylene. For general setup, see Figure S1-S4.

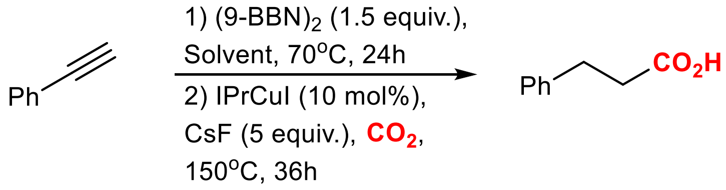

Inside of glove box 45 mL pressure tube was charged with phenylacetylene (0.979 mmol), $(9\text{-BBN})_2$ (1.5 equiv.) and corresponding dry solvent (4 mL). The flask was closed with suitable cap, removed from the glove box and heated to 70°C for 24h. Afterwards, the pressure tube was transferred back to the glove box. To the reaction mixture at 20°C was added CsF (5 equiv.) and previously prepared solution of catalyst (the

mixture of CuI (10 mol%), IPrHCl (12 mol%) and NaO*t*Bu (12 mol%) in appropriate dry solvent (3 mL) was stirred at 20°C for 30 min). The pressure tube was closed with the cap and removed from the glove box. Afterwards CO_2 (120 mL) was added *via* a syringe, which was followed by stirring of reaction mixture at 150°C for 36h. Next, the reaction mixture was diluted with 30 mL Et_2O and transferred into 500 mL separating funnel. The resulting mixture was extracted with 30 mL saturated $NaHCO_3$ solution (3 times). The resulting basic solution was washed with 15 mL Et_2O (once), acidified (50-55 mL 6M HCl) and extracted with 30 mL Et_2O (3 times). The resulting solution of Et_2O was distilled to dryness and if necessary was further purified by column chromatography.
Other renewable solvents like 2MeTHF, Acetal, diethoxymethane or dimethoxymethane without any noticeable difference can replace Et_2O (the difference was in the range ±3%). Similarly, saturated solution of $NaHCO_3$ can be replaced by 2M solution of KOH.

General experimental procedure for Cu-catalyzed hydrocarboxylation of phenylacetylene. For general setup, see Figure S1-S4.

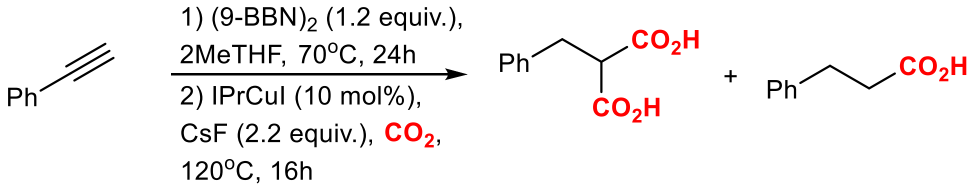

Inside of glove box 45 mL pressure tube was charged with phenylacetylene (1.470 mmol), $(9\text{-}BBN)_2$ (1.2 equiv.) and dry 2MeTHF (4 mL). The flask was closed with suitable cap, removed from the glove box and heated to 70°C for 24h. Afterwards, the pressure tube was transferred back to the glove box. To the reaction mixture at 20°C was added CsF (2.2 equiv.) and previously prepared solution of catalyst (the mixture of CuI (10 mol%), IPrHCl (12 mol%) and NaO*t*Bu (12 mol%) in dry 2MeTHF (3 mL) was stirred at 20°C for 30 min). The pressure tube was closed with the cap and removed from the glove box. Afterwards CO_2 (120 mL) was added *via* a syringe, which was followed by stirring of reaction mixture at 120°C for 16h. Next, the reaction mixture was diluted with 30 mL Et_2O and transferred into 500 mL separating funnel. The resulting mixture was extracted with 30 mL saturated $NaHCO_3$ solution (3 times). The resulting basic solution was washed with 15 mL Et_2O (once), acidified (50-55 mL 6M HCl) and extracted with 30 mL Et_2O (3 times). The resulting solution of Et_2O was distilled to dryness to give the mixture of acids.
Other renewable solvents like 2MeTHF, Acetal, diethoxymethane or dimethoxymethane without any noticeable difference can replace Et_2O (the difference was in the range ±3%). Similarly, saturated solution of $NaHCO_3$ can be replaced by 2M solution of KOH.

General experimental procedure for Fe-catalyzed hydrocarboxylation of 4-methylstyrene.

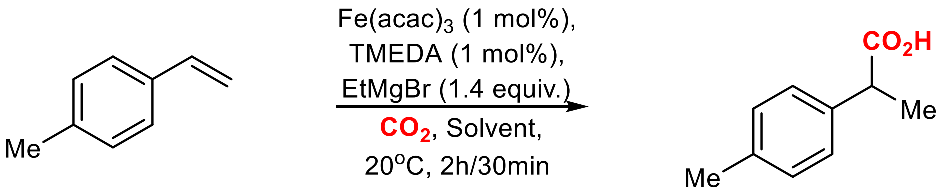

Inside of glove box 50 mL Schlenk flask was charged with $Fe(acac)_3$ (1 mol%). The flask was sealed with a rubber septa, removed from the glove box and equipped with an Ar balloon. Next, to the reaction mixture at 20°C was added previously prepared solution of 4-methylstyrene (5.080 mmol) and TMEDA (1 mol%) in corresponding dry solvent (8 mL THF, 14 mL biosolvent). The resulting mixture was stirred at 20°C for 10min, which was followed by dropwise addition of 3.4M EtMgBr solution in 2MeTHF (1.4 equiv.) (in case of the reaction in THF 1M EtMgBr solution in THF (1.4 equiv.)). The resulting mixture was stirred at 20°C for 2h. Further, the reaction mixture was transferred into an ice bath where it was bubbled with CO_2 for 10min. Then the ice bat was removed and bubbling was continued for another 20min at 20°C. The resulting mixture, equipped with a CO_2 balloon, was stirred at 20°C for 12h. Next, the reaction mixture was carefully treated with 6M HCl (3 mL), diluted with 30 mL Et_2O and transferred into 500 mL separating funnel. This was followed by careful addition of $NaHCO_3$ (1 g). The resulting mixture was extracted with 30 mL saturated $NaHCO_3$ solution (3 times). The resulting basic solution was washed with 15 mL Et_2O or DCM (once), acidified (70 mL 6M HCl) and extracted with 30 mL Et_2O (3 times). The resulting solution of Et_2O was distilled to dryness to give corresponding acid.

Other renewable solvents like 2MeTHF, Acetal, diethoxymethane or dimethoxymethane without any noticeable difference can replace Et_2O (the difference was in the range ±3%).

General experimental procedure for Ni-catalyzed hydrocarboxylation of diphenylacetylene.

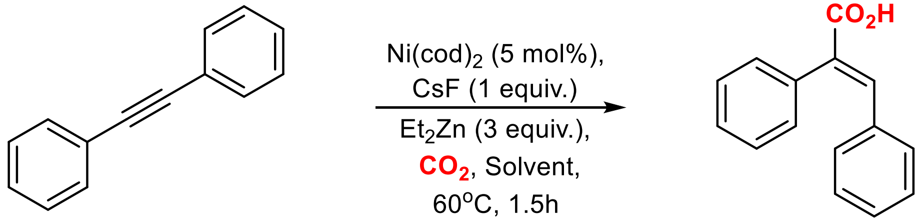

Inside of glove box, 25 mL Schlenk flask was charged with $Ni(cod)_2$ (5 mol%), CsF (1 equiv.), diphenylacetylene (1.120 mmol) and sealed with rubber septa. Next, the flask was evacuated and fitted with a CO_2 balloon. This was followed by addition of corresponding dry solvent (8 mL). Finally, Et_2Zn (1 M solution in hexane, 3 equiv.) was carefully dropped to the reaction mixture, which was followed by stirring of the reaction mixture at 60°C for 1.5h. Next, the reaction mixture was carefully treated with 3 mL 6M HCl solution, diluted with 30 mL Et_2O and

transferred into 500 mL separating funnel. This was followed by careful addition of $NaHCO_3$ (1 g). The resulting mixture was extracted with 30 mL saturated $NaHCO_3$ solution (3 times). The resulting basic solution was washed with 15 mL Et_2O or DCM (once), acidified (70 mL 6M HCl) and extracted with 30 mL Et_2O (3 times). The resulting solution of Et_2O was distilled to dryness to give corresponding acid.
Other renewable solvents like 2MeTHF, Acetal, diethoxymethane or dimethoxymethane without any noticeable difference can replace Et_2O (the difference was in the range ±3%).

General experimental procedure for Cu-catalyzed hydrocarboxylation of diphenylacetylene.

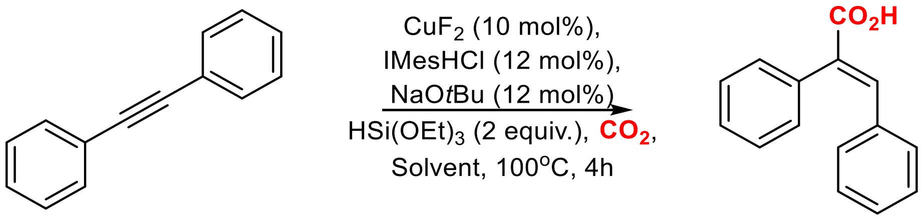

25mL Schlenk flask was charged with diphenylacetylene (1.120 mmol), sealed with a rubber septa, evacuated and fitted with a CO_2 balloon. This was followed by addition of corresponding dry solvent (2 mL). Next, inside of glove box a 10 mL round bottom flask was charged with CuF_2 (10 mol%), IMesHCl (12 mol%), NaO*t*Bu (12 mol%) and sealed with a rubber septa. The flask was removed from the glove box and equipped with an Ar balloon, which was followed by addition of corresponding dry solvent (3 mL). The resulting mixture was stirred at 20°C for 30 min. Further, the catalyst was transferred into the Schlenk flask containing diphenylacetylene. The Schlenk flask was covered by Al-folia, which was followed by addition of triethoxysilane (2 equiv.). The resulting mixture was stirred at 20°C for 1 min and transferred into the pre-heated oil bath where it was stirred at 100°C for 4h (in case of 2MeTHF the reaction was conducted at 90°C). This was followed by addition of 2M KOH (5 mL) and stirring of the resulting mixture at 20°C for 1h. Next, the reaction mixture was diluted with 30 mL Et_2O and transferred into 500 mL separating funnel. The resulting mixture was extracted with 30 mL saturated $NaHCO_3$ solution (3 times). The resulting basic solution was washed with 15 mL Et_2O (once), acidified (55 mL 6M HCl) and extracted with 30 mL Et_2O (3 times). The resulting solution of Et_2O was distilled to dryness to give corresponding acid.
Other renewable solvents like 2MeTHF, Acetal, diethoxymethane or dimethoxymethane without any noticeable difference can replace Et_2O (the difference was in the range ±3%).

General experimental procedure for Fe-catalyzed hydrocarboxylation of diphenylacetylene.

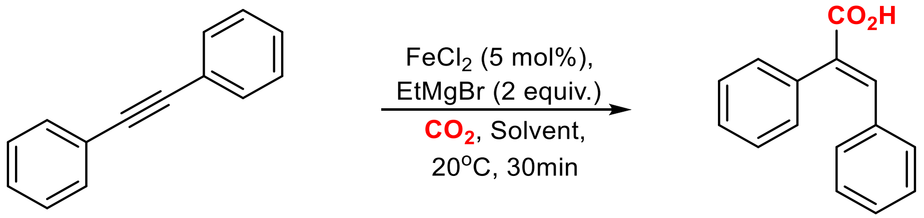

Inside of glove box, 25mL Schlenk flask was charged with $FeCl_2$ (5 mol%) and diphenylacetylene (1.120 mmol). The flask was sealed with a rubber septa, removed from the glove box and equipped with an Ar balloon. Next, to the reaction mixture was added corresponding dry solvent (6 mL) and 3.4M solution of EtMgBr in 2MeTHF (2 equiv.) (in case of the reaction in Et_2O 1M EtMgBr solution in THF (2 equiv.)). The resulting mixture was stirred at 20°C for 15min, then Ar balloon was replaced by CO_2 balloon and the reaction mixture was transferred into an ice bath where it was bubbled with CO_2 for 10min. Then the ice bat was removed and bubbling was continued for another 20min at 20°C. The resulting mixture, equipped with a CO_2 balloon, was stirred at 20°C for 12h. Next, the reaction mixture was carefully treated with 3 mL 6M HCl solution, diluted with 30 mL Et_2O and transferred into 500 mL separating funnel. This was followed by careful addition of $NaHCO_3$ (1 g). The resulting mixture was extracted with 30 mL saturated $NaHCO_3$ solution (3 times). The resulting basic solution was washed with 15 mL Et_2O or DCM (once), acidified (70 mL 6M HCl) and extracted with 30 mL Et_2O (3 times). The resulting solution of Et_2O was distilled to dryness to give corresponding acid.
Other renewable solvents like 2MeTHF, Acetal, diethoxymethane or dimethoxymethane without any noticeable difference can replace Et_2O (the difference was in the range ±3%).

General experimental procedure for Cu-catalyzed borocarboxylation of 4-methylstyrene.

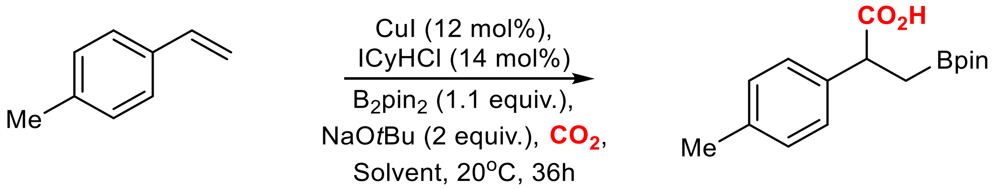

Inside of glove box, 50 mL Schlenk flask was charged with B_2pin_2 (1.1 equiv.) and sealed with rubber septa. Next, the flask was evacuated and fitted with a CO_2 balloon. This was followed by addition of 4-methylstyrene (1.690 mmol) and corresponding dry solvent (10 mL). Afterwards, under the flow of argon a 50 mL round bottom flask was charged with corresponding solvent (15 mL) and sealed with a robber septa. The flask was transferred into the glove box where it was charged with CuCl (12 mol%), ICyHCl (14 mol%) and NaO*t*Bu (2 equiv.). The flask was sealed, removed from the glove box and stirred at 20°C for 2h. Afterwards, the content of 50 mL round bottom flask (the catalyst/base) was transferred into the Schlenk flask filled with starting materials and CO_2, which was followed by stirring of the reaction

mixture at 20°C for 36h. Next, the reaction mixture was diluted with 30 mL Et_2O and transferred into 500 mL separating funnel. The resulting mixture was extracted with 30 mL saturated $NaHCO_3$ solution (3 times). The resulting basic solution was washed with 15 mL Et_2O (once), acidified (50-55 mL 6M HCl) and extracted with 30 mL Et_2O (3 times). The resulting solution of Et_2O was distilled to dryness to give corresponding acid.

Other renewable solvents like 2MeTHF, Acetal, diethoxymethane or dimethoxymethane without any noticeable difference can replace Et_2O (the difference was in the range ±3%).

General experimental procedure for Zr-catalyzed carbocarboxylation of 4-methylstyrene.

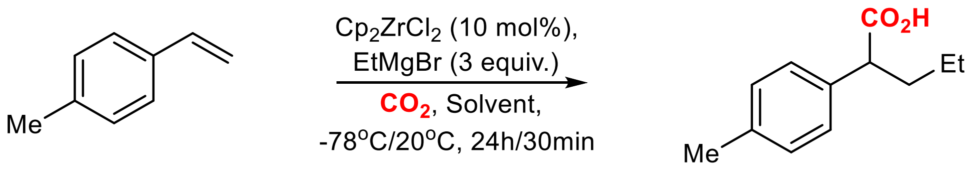

Inside of glove box 50 mL round bottom flask was charged with Cp_2ZrCl_2 (10 mol%). The flask was sealed with a rubber septa, removed from the glove box and equipped with an Ar balloon. This was followed by addition of corresponding dry solvent (6 mL THF, 13 mL biosolvent) and EtMgBr (3.4 M 2MeTHF solution, 3 equiv. in case of biosolvents, 1 M THF solution, 3 equiv. in case of THF) at -78°C. The resulting mixture was allowed to reach 20°C where it was stirred for 1h. Then it was transferred back to isopropanol bath (-78°C), which was followed by addition of 4-methylstyrene (3.380 mmol). The resulting mixture was stirred at -78°C for 1h and allowed to reach 20°C, were it was stirred for 24h. Further, the reaction mixture was transferred into an ice bath, the Ar balloon was replaced by CO_2 balloon and the reaction mixture was bubbled with CO_2 for 10 min. Next, the ice bath was removed and bubbling was continued for another 20 min at 20°C. The resulting mixture, equipped with a CO_2 balloon, was stirred at 20°C for 12h. Next, the reaction mixture was carefully treated with 3 mL 6M HCl solution, diluted with 30 mL Et_2O and transferred into 500 mL separating funnel. This was followed by careful addition of $NaHCO_3$ (1 g). The resulting mixture was extracted with 30 mL saturated $NaHCO_3$ solution (3 times). The resulting basic solution was washed with 15 mL Et_2O or DCM (once), acidified (70 mL 6M HCl) and extracted with 30 mL Et_2O (3 times). The resulting solution of Et_2O was distilled to dryness to give corresponding acid.

Other renewable solvents like 2MeTHF, Acetal, diethoxymethane or dimethoxymethane without any noticeable difference can replace Et_2O (the difference was in the range ±3%).

General experimental procedure for Cu-catalyzed carboxylation of triethoxyphenylsilane. For general setup, see Figure S1-S4.

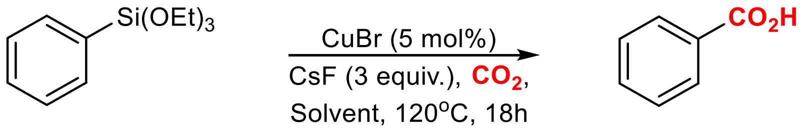

Inside of glove box 45 mL pressure tube was charged with triethoxyphenylsilane (0.832 mmol), CuBr (5 mol%), CsF (3 equiv.) and corresponding dry solvent (3 mL). The pressure tube was closed with the cap and removed from the glove box. Afterwards CO_2 (120 mL) was added *via* a syringe, which was followed by stirring of reaction mixture at 120°C for 18h. Next, the reaction mixture was diluted with 30 mL Et_2O and transferred into 500 mL separating funnel. The resulting mixture was extracted with 30 mL saturated $NaHCO_3$ solution (3 times). The resulting basic solution was washed with 15 mL Et_2O (once), acidified (50-55 mL 6M HCl) and extracted with 30 mL Et_2O (3 times). The resulting solution of Et_2O was distilled to dryness to give corresponding acid.

In cases of GVL and Et_2Suc the basic solution was washed with either DCM or Et_2O (15 mL, 3 times), and the final Et_2O solution was washed with 10 mL distilled water (3 times) before evaporation.

Other renewable solvents like 2MeTHF, Acetal, diethoxymethane or dimethoxymethane without any noticeable difference can replace Et_2O (the difference was in the range ±3%). Similarly, saturated solution of $NaHCO_3$ can be replaced by 2M solution of KOH.

General experimental procedure for C-H carboxylation of phenylacetylene. For general setup, see Figure S1-S4.

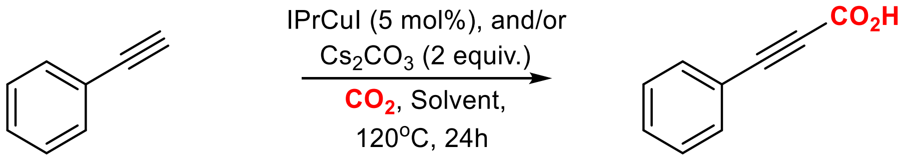

Inside of glove box 45 mL pressure tube was charged with phenylacetylene (1.960 mmol), Cs_2CO_3 (2 equiv.) and corresponding dry solvent (Cu-free GVL 7 mL, Cu-cat GVL 4 mL, Cu-free 2MeTHF 7 mL, Cu-cat 2MeTHF/Acetal/THF 4mL). In case of Cu-catalyzed reactions this was followed by addition of previously prepared solution of the catalyst (the mixture of CuI (5 mol%), IPrHCl (6 mol%) and NaO*t*Bu (6 mol%) in appropriate dry solvent (3 mL) was stirred at 20°C for 30 min). The pressure tube was closed with the cap and removed from the glove box. Afterwards CO_2 (120 mL) was added *via* a syringe, which was followed by stirring of reaction mixture at 120°C for 24h. Next, the reaction mixture was diluted with 30 mL Et_2O and transferred into 500 mL separating funnel. The resulting mixture was extracted with 30 mL saturated $NaHCO_3$ solution (3 times). The resulting basic solution was washed with 15 mL Et_2O (once), acidified (50-55 mL 6M HCl) and extracted with 30 mL Et_2O (3 times). The resulting solution of Et_2O was distilled to dryness to give corresponding acid.

In cases of GVL the basic solution was washed with either DCM or Et_2O (15 mL, 3 times), and the final Et_2O solution was washed with 10 mL distilled water (3 times) before evaporation.

Other renewable solvents like 2MeTHF, Acetal, diethoxymethane or dimethoxymethane without any noticeable difference can replace Et_2O (the difference was in the range ±3%). Similarly, saturated solution of $NaHCO_3$ can be replaced by 2M solution of KOH.

Characterization of products

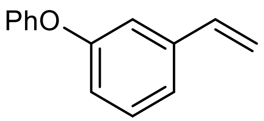

***1-Phenoxy-3-vinylbenzene*, 3ra.**[1] Starting from 7.567 mmol of corresponding aldehyde the product was obtained as a colourless liquid, yield 87% (1.285 g, 2MeTHF). **^{1}H NMR** (400 MHz, $CDCl_3$): δ = 5.26 (dd, *J* = 10.8, 0.9 Hz, 1H, olefin), 5.73 (dd, *J* = 17.6, 0.9 Hz, 1H, olefin), 6.68 (dd, *J* = 17.6, 10.9 Hz, 1H, olefin), 6.90-6.93 (m, 1H, Ar), 7.01-7.04 (m, 2H, Ar), 7.08-7.17 (m, 3H, Ar), 7.27-7.37 (m, 3H, Ar). **^{13}C NMR** (101 MHz, $CDCl_3$): δ = 114.8, 116.7, 118.5, 119.0, 121.5, 123.4, 129.9, 130.0, 136.5, 139.7, 157.4, 157.7.

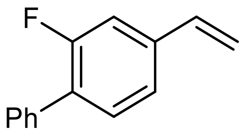

***2-Fluoro-4-vinyl-1,1'-biphenyl*, 3sa.**[2] Starting from 7.492 mmol of corresponding aldehyde the product was obtained as a colourless liquid, yield 88% (1.311 g, 2MeTHF). **^{1}H NMR** (400 MHz, $CDCl_3$): δ = 5.26 (d, *J* = 10.9 Hz, 1H, olefin), 5.73 (d, *J* = 17.5 Hz, 1H, olefin), 6.64 (dd, *J* = 17.5, 10.8 Hz, 1H, olefin), 7.12-7.19 (m, 2H, Ar), 7.28-7.40 (m, 4H, Ar), 7.48-7.51 (m, 2H, Ar). **^{13}C NMR** (101 MHz, $CDCl_3$): δ = 113.6 (d, *J* = 23.5 Hz), 115.4, 122.6 (d, *J* = 3.2 Hz), 127.9, 128.6, 129.1 (d, *J* = 3.0 Hz), 130.9 (d, *J* = 4.1 Hz), 135.7 (d, *J* = 2.3 Hz), 135.8, 139.1 (d, *J* = 8.0 Hz), 161.4.

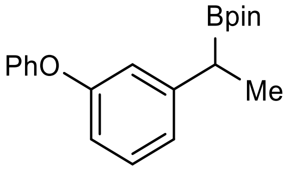

***4,4,5,5-Tetramethyl-2-(1-(3-phenoxyphenyl)ethyl)-1,3,2-dioxaborolane*, 3rb.** Starting from 2.550 mmol of corresponding styrene the product was obtained as a colourless liquid, yield 82% (0.674 g, Cym). **^{1}H NMR** (400 MHz, $CDCl_3$): δ = 1.19 (d, *J* = 2.7 Hz, 12H, Bpin), 1.31 (d, *J* = 7.5 Hz, 3H, Me), 2.42 (q, *J* = 7.5 Hz, 1H, CH), 6.79 (ddd, *J* = 8.1, 2.5, 1.0 Hz, 1H, Ar), 6.90 (t, *J* = 2.1 Hz, 1H, Ar), 6.96-7.02 (m, 3H, Ar), 7.05-7.09 (m, 1H, Ar), 7.22 (t, *J* = 7.9 Hz, 1H, Ar), 7.28-7.34 (m, 2H, Ar). **^{13}C NMR** (101 MHz, $CDCl_3$): δ = 17.1, 24.8, 24.8, 83.5, 116.0, 118.7, 118.9, 123.0, 123.1, 129.6, 129.8, 147.3, 157.2, 157.7.

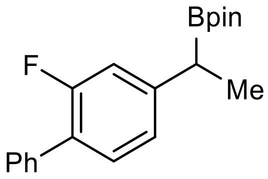

***2-(1-(2-Fluoro-[1,1'-biphenyl]-4-yl)ethyl)-4,4,5,5-tetramethyl-1,3,2-dioxaborolane*, 3sb.**[2] Starting from 2.520 mmol of corresponding styrene the product was obtained as a colourless liquid, yield 77% (0.633 g, Cym). **^{1}H NMR** (400 MHz, $CDCl_3$): δ = 1.24 (d, *J* = 3.7 Hz, 12H, Bpin), 1.36 (d, *J* = 7.5 Hz, 3H, Me), 2.48 (q, *J* = 7.5 Hz, 1H, CH), 7.01-7.07 (m, 2H, Ar), 7.31-7.36 (m, 2H, Ar), 7.42 (t, *J* = 7.6 Hz, 2H, Ar), 7.54-7.56 (m, 2H, Ar). **^{13}C NMR** (101 MHz, $CDCl_3$): δ = 17.0, 24.8, 24.8, 83.7, 115.4 (d, *J* = 23.0 Hz), 124.0, 127.4, 128.5, 129.1, 130.5.

[1] N. Hu, H. Jung, Y. Zheng, J. Lee, L. Zhang, Z. Ullah, X. Xie, K. Harms, M.-H. Baik and E. Meggers, *Angew. Chem. Int. Ed.*, 2018, **57**, 6242-6246.
[2] R. D. Grigg, J. W. Rigoli, R. V. Hoveln, S. Neale and J. M. Schomaker, *Chem. Eur. J.*, 2012, **18**, 9391-9396.

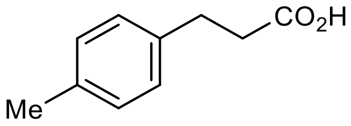

***3-(p-Tolyl)propanoic acid,* 2a.**[3] Starting from 0.846 mmol of corresponding styrene the product was obtained as a white solid, m.p. = 116-118°C, yield 98% (0.137 g, 2MeTHF), 94% (0.130 g, Euc), 91% (0.126 g, Acetal), 92% (0.128 g, Me_2Isos). **^{1}H NMR** (400 MHz, $CDCl_3$): δ = 2.33 (s, 3H, Me), 2.67 (dd, *J* = 8.4, 7.2 Hz, 2H, CH_2), 2.93 (t, *J* = 7.8 Hz, 2H, CH_2), 7.11 (s, 4H, Ar), 10.48 (br s, 1H, CO_2H). **^{13}C NMR** (101 MHz, $CDCl_3$): δ = 21.2, 30.4, 35.9, 128.2, 129.4, 136.1, 137.3, 179.2. **HRMS-EI** (m/z) $[M-H]^-$ calcd. for $C_{10}H_{11}O_2$ 163.0765 found 163.0765.

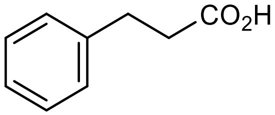

***3-Phenylpropanoic acid,* 2b.**[4] Starting from 1.920 mmol of corresponding styrene the product was obtained as a white solid, m.p. = 46-48°C, yield 89% (0.256 g, 2MeTHF). In case of decarboxylative hydrocarboxylation of phenylacetylene (0.979 mmol) the product was obtained as a white solid, m.p. = 45-48°C, yield 80% (0.117 g, 2MeTHF), 74% (0.108 g, Acetal), 70% (0.103 g, Euc), 84% (0.124 g, dioxane). **^{1}H NMR** (400 MHz, $CDCl_3$): δ = 2.80 (dd, *J* = 8.4, 7.2 Hz, 2H, CH_2), 3.08 (t, *J* = 7.8 Hz, 2H, CH_2), 7.32-7.36 (m, 3H, Ar), 7.40-7.47 (m, 2H, Ar), 12.02 (br s, 1H, CO_2H). **^{13}C NMR** (101 MHz, $CDCl_3$): δ = 30.6, 35.7, 126.5, 128.3, 128.6, 140.2, 179.8. **HRMS-EI** (m/z) $[M-H]^-$ calcd. for $C_9H_9O_2$ 149.0608 found 149.0610.

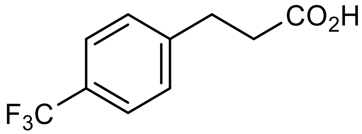

***3-(4-(Trifluoromethyl)phenyl)propanoic acid,* 2c.**[4] Starting from 1.160 mmol of corresponding styrene the product was obtained as a white solid, m.p. = 107-109°C, yield 94% (0.239 g, 2MeTHF). **^{1}H NMR** (400 MHz, $CDCl_3$): δ = 2.72 (t, *J* = 7.6 Hz, 2H, CH_2), 3.02 (t, *J* = 7.6 Hz, 2H, CH_2), 7.33 (d, *J* = 8.0 Hz, 2H, Ar), 7.56 (d, *J* = 8.0 Hz, 2H, Ar), 11.27 (br s, 1H, CO_2H). **^{13}C NMR** (101 MHz, $CDCl_3$): δ = 30.4, 35.3, 123.1, 125.70 (q, *J* = 3.9 Hz), 128.8, 129.2, 144.3, 179.0. **HRMS-EI** (m/z) $[M-H]^-$ calcd. for $C_{10}H_8F_3O_2$ 217.0482 found 217.0482.

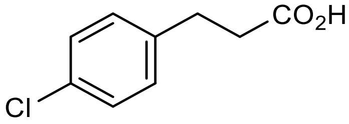

***3-(4-Chlorophenyl)propanoic acid,* 2d.**[4] Starting from 1.440 mmol of corresponding styrene the product was obtained as a white solid, m.p. = 129-131°C, yield 91% (0.242 g, 2MeTHF). **^{1}H NMR** (400 MHz, $CDCl_3$): δ = 2.63 (dd, *J* = 8.1, 7.2 Hz, 2H, CH_2), 2.89 (t, *J* = 7.7 Hz, 2H, CH_2), 7.08-7.12 (m, 2H, Ar), 7.21-7.24 (m, 2H, Ar), 10.59 (br s, 1H, CO_2H). **^{13}C NMR** (101 MHz, $CDCl_3$): δ = 30.1, 35.6, 128.9, 129.8, 132.4, 138.7, 179.0. **HRMS-EI** (m/z) $[M-H]^-$ calcd. for $C_9H_8ClO_2$ 183.0218 found 183.0216.

---

[3] A. Kisic, M. Stephan and B. Mohar, *Adv. Synth. Catal.*, 2015, **357**, 2540-2546.
[4] H. Chen, J. Wang, X. Hong, H.-B. Zhou and C. Dong, *Can. J. Chem.*, 2012, **90**, 758-761.

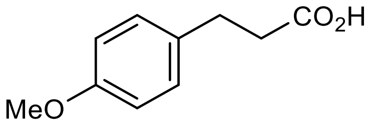

***3-(4-Methoxyphenyl)propanoic acid*, 2e.**[4] Starting from 1.490 mmol of corresponding styrene the product was obtained as a white solid, m.p. = 98-100ºC, yield 89% (0.239 g, 2MeTHF). **^{1}H NMR** (400 MHz, $CDCl_3$): δ = 2.66 (t, *J* = 7.8 Hz, 2H, CH_2), 2.91 (t, *J* = 7.7 Hz, 2H, CH_2), 3.79 (s, 3H, OMe), 6.84-6.86 (m, 2H, Ar), 7.12-7.15 (m, 2H, Ar), 11.05 (br s, 1H, CO_2H). **^{13}C NMR** (101 MHz, $CDCl_3$): δ = 29.9, 36.1, 55.4, 114.2, 129.4, 132.4, 158.3, 179.4. **HRMS-EI** (m/z) $[M-H]^-$ calcd. for $C_{10}H_{11}O_3$ 179.0714 found 179.0717.

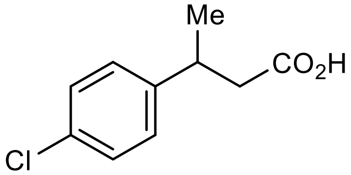

***3-(4-Chlorophenyl)butanoic acid*, 2f.**[5] Starting from 1.310 mmol of corresponding styrene the product was obtained as a white solid, m.p. = 89-91ºC, yield 83% (0.216 g, 2MeTHF). **^{1}H NMR** (400 MHz, $CDCl_3$): δ = 1.36 (d, *J* = 7.0 Hz, 3H, Me), 2.60-2.72 (m, 2H, CH_2), 3.31 (h, *J* = 7.2 Hz, 1H, CH), 7.20-7.23 (m, 2H, Ar), 7.31-7.35 (m, 2H, Ar), 9.96 (br s, 1H, CO_2H). **^{13}C NMR** (101 MHz, $CDCl_3$): δ = 22.0, 35.8, 42.6, 128.3, 128.9, 132.4, 144.0, 178.5. **HRMS-EI** (m/z) $[M-H]^-$ calcd. for $C_{10}H_{10}ClO_2$ 197.0375 found 197.0376.

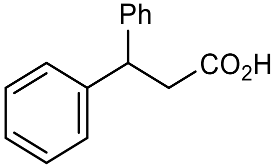

***3,3-Diphenylpropanoic acid*, 2g.**[6] Starting from 1.110 mmol of corresponding styrene the product was obtained as a white solid, m.p. = 152-154ºC, yield 82% (0.206 g, 2MeTHF). **^{1}H NMR** (400 MHz, $CDCl_3$): δ = 3.17 (d, *J* = 7.9 Hz, 2H, CH_2), 4.61 (t, *J* = 7.9 Hz, 1H, CH), 7.26-7.39 (m, 10H, Ar), 10.40 (br s, 1H, CO_2H). **^{13}C NMR** (101 MHz, $CDCl_3$): δ = 40.6, 46.8, 126.8, 127.8, 128.8, 143.4, 178.1. **HRMS-EI** (m/z) $[M-H]^-$ calcd. for $C_{15}H_{13}O_2$ 225.0921 found 225.0920.

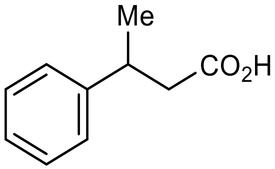

***3-Phenylbutanoic acid*, 2h.**[5] Starting from 1.690 mmol of corresponding styrene the product was obtained as a white solid, m.p. = 36-38ºC, yield 94% (0.260 g, 2MeTHF), 88% (0.243 g, Euc), 94% (0.258 g, Acetal), 85% (0.236 g, Me_2Isos). **^{1}H NMR** (400 MHz, $CDCl_3$): δ = 1.43 (d, *J* = 7.0 Hz, 3H, Me), 2.65-2.81 (m, 2H, CH_2), 3.39 (dt, *J* = 8.1, 6.8 Hz, 1H, CH), 7.29-7.34 (m, 3H, Ar), 7.39-7.43 (m, 2H, Ar), 11.94 (br s, 1H, CO_2H). **^{13}C NMR** (101 MHz, $CDCl_3$): δ = 21.9, 36.2, 42.7, 126.6, 126.8, 128.7, 145.5, 179.3. **HRMS-EI** (m/z) $[M-H]^-$ calcd. for $C_{10}H_{11}O_2$ 163.0765 found 163.0764.

[5] Y. Wang, W. Ren, J. Li, H. Wang and Y. Shi, *Org. Lett.,* 2014, **16**, 5960-5963.
[6] M. Juhl, S. L. R. Laursen, Y. Huang, D. U. Nielsen, K. Daasbjerg and T. Skrydstrup, *ACS Catal.,* 2017, **7**, 1392-1396.

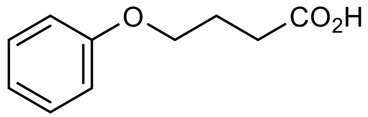

***4-Phenoxybutanoic acid*, 2i.**[7] Starting from 1.490 mmol of corresponding olefin the product was obtained as a white solid, m.p. = 63-65ºC, yield 95% (0.255 g, 2MeTHF). **^{1}H NMR** (400 MHz, $CDCl_3$): δ = 2.10-2.17 (m, 2H, CH_2), 2.61 (t, J = 7.3 Hz, 2H, CH_2), 4.03 (t, J = 6.1 Hz, 2H, CH_2), 6.89-6.92 (m, 2H, Ar), 6.94-6.98 (m, 1H, Ar), 7.27-7.31 (m, 2H, Ar), 11.45 (br s, 1H, CO_2H). **^{13}C NMR** (101 MHz, $CDCl_3$): δ = 24.6, 30.8, 66.6, 114.7, 121.0, 129.6, 158.9, 179.9. **HRMS-EI** (m/z) $[M-H]^-$ calcd. for $C_{10}H_{11}O_3$ 179.0714 found 179.0715.

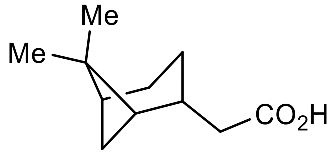

***2-(6,6-Dimethylbicyclo[3.1.1]heptan-2-yl)acetic acid*, 2j.**[6] Starting from 1.470 mmol of corresponding olefin the product was obtained as a colourless viscous oil, yield 73% (0.195 g, 2MeTHF). **^{1}H NMR** (400 MHz, $CDCl_3$): δ = 0.85 (s, 3H, Me), 1.20 (s, 3H, Me), 1.23-1.35 (m, 2H, CH_2), 1.70-1.82 (m, 4H, $2xCH_2$), 1.87 (qd, J = 5.2, 1.4 Hz, 1H, CH), 2.03-2.09 (m, 1H, CH), 2.19-2.29 (m, 2H, CH_2), 2.42-2.50 (m, 1H, CH), 11.43 (br s, 1H, CO_2H). **^{13}C NMR** (101 MHz, $CDCl_3$): δ = 20.2, 21.8, 23.4, 24.4, 26.9, 32.2, 39.7, 40.8, 41.0, 45.7, 180.1. **HRMS-EI** (m/z) $[M-H]^-$ calcd. for $C_{11}H_{17}O_2$ 181.1234 found 181.1234.

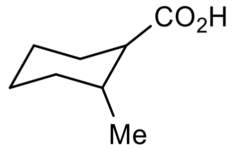

***2-Methylcyclohexane-1-carboxylic acid*, 2k.**[6] Starting from 1.870 mmol of corresponding olefin the product was obtained as a colourless viscous oil, yield 52% (0.137 g, 2MeTHF). **^{1}H NMR** (400 MHz, $CDCl_3$): δ = 0.93 (d, J = 6.5 Hz, 3H, Me), 1.16-1.35 (m, 2H, CH_2), 1.41-1.51 (m, 1H, CH), 1.57-1.80 (m, 5H, $2xCH_2$ + CH), 1.91-1.99 (m, 2H, CH_2), 11.05 (br s, 1H, CO_2H). **^{13}C NMR** (101 MHz, $CDCl_3$): δ = 20.8, 25.5, 25.9, 30.0, 30.4, 34.2, 34.3, 51.4, 183.1. **HRMS-EI** (m/z) $[M-H]^-$ calcd. for $C_8H_{13}O_2$ 141.0921 found 141.0924.

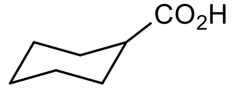

***Cyclohexanecarboxylic acid*, 2l.**[6] Starting from 2.435 mmol of corresponding olefin the product was obtained as a colourless liquid, yield 71% (0.220 g, 2MeTHF), 37% (0.114 g, Euc), 49% (0.153 g, Acetal), 44% (0.137 g, Me_2Isos) 73% (0.228 g, dioxane). **^{1}H NMR** (400 MHz, $CDCl_3$): δ = 1.20-1.33 (m, 3H, CH_2), 1.39-1.49 (m, 2H, CH_2), 1.62-1.65 (m, 1H, CH_2), 1.72-1.78 (m, 2H, CH_2), 1.90-1.95 (m, 2H, CH_2), 2.32 (tt, J = 11.2, 3.7 Hz, 1H, CH), 11.31 (br s, 1H, CO_2H). **^{13}C NMR** (101 MHz, $CDCl_3$): δ = 25.5, 25.8, 28.9, 43.1, 183.0. **HRMS-EI** (m/z) $[M-H]^-$ calcd. for $C_7H_{11}O_2$ 127.0765 found 127.0767.

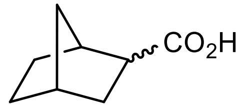

***Bicyclo[2.2.1]heptane-2-carboxylic acid*, mixture of diastereomers dr = 1:1.25 (NMR), 2m.**[6] Starting from 1.590 mmol of corresponding olefin the product was obtained as a colourless viscous oil, yield 65% (0.144 g, 2MeTHF). **^{1}H NMR** (400 MHz, $CDCl_3$): δ = 1.14-1.28 (m, 6.48H, CH_2), 1.42-1.54 (m, 10.11H, CH_2), 1.78-1.86 (m, 2.65H, 2xCH), 2.26-2.38 (m, 4.19H, CH_2 + 2xCH), 2.47-2.48 (m, 1.25H, CH), 2.54-2.55 (m, 1H, CH), 10.18 (br s, 1.39H, $2xCO_2H$). **^{13}C NMR** (101 MHz,

[7] Z.-z. Zhou, M. Liu, L. Lv and C.-J. Li, *Angew. Chem. Int. Ed.*, 2018, **57**, 2616-2620.

$CDCl_3$): δ = 28.7, 28.8, 29.6, 29.7, 30.5, 34.3, 34.4, 36.2, 36.6, 36.7, 41.0, 41.1, 46.5, 46.6, 51.9, 177.2, 182.3. **HRMS-EI** (m/z) $[M-H]^-$ calcd. for $C_8H_{11}O_2$ 139.0765 found 139.0766.

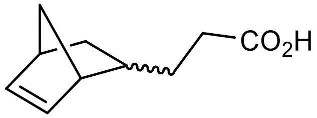

***3-Bicyclo[2.2.1]hept-5-en-2-yl)propanoic acid*, mixture of diastereomers dr = 10:1.3 (NMR), 2n.** Starting from 1.660 mmol of corresponding diene the product was obtained as a colourless viscous oil, yield 58% (0.160 g, 2MeTHF). **^{1}H NMR** (400 MHz, $CDCl_3$): δ = 0.46-0.50 (m, 1.31H, CH), 1.18-1.21 (m, 1.31H, CH), 1.35-1.41 (m, 4.30H, CH_2), 1.78-1.86 (m, 2.41H, CH_2), 1.92-2.02 (m, 1.49H, CH), 2.21-2.33 (m, 4.61H, CH_2), 2.72-2.75 (m, 2.15H, CH_2), 5.90 (dt, *J* = 5.5, 2.5 Hz, 1H, olefin, diastereomer A), 5.99 (dt, *J* = 5.6, 2.7 Hz, 0.10H, olefin, diastereomer B), 6.05 (dt, *J* = 5.6, 2.6 Hz, 0.13H, olefin, diastereomer B), 6.10 (dt, *J* = 5.9, 3.0 Hz, 0.94H, olefin, diastereomer A), 10.08 (br s, 1.20H, CO_2H). **^{13}C NMR** (101 MHz, $CDCl_3$): δ = 26.9, 29.8, 30.0, 31.7, 32.3, 32.9, 33.3, 33.4, 33.6, 38.1, 38.3, 38.4, 38.6, 38.7, 41.6, 42.0, 42.6, 45.2, 45.3, 46.2, 46.8, 49.7, 49.8, 51.6, 51.7, 132.1, 132.2, 137.4, 137.5, 174.7, 180.0. **HRMS-EI** (m/z) $[M-H]^-$ calcd. for $C_{10}H_{13}O_2$ 165.0921 found 165.0921.

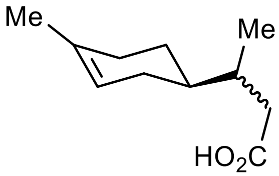

***3-(4-Methylcyclohex-3-en-1-yl)butanoic acid*, mixture of diastereomers dr = 10:1 (NMR), 2o.**[6] Starting from 1.470 mmol of corresponding diene the product was obtained as a colourless viscous oil, yield 73% (0.196 g, 2MeTHF). **^{1}H NMR** (400 MHz, $CDCl_3$): δ = 0.91-0.96 (m, 4.8H, Me), 1.19-1.32 (m, 2.41H, CH_2), 1.38-1.49 (m, 2.25H, CH_2), 1.62 (d, *J* = 2.2 Hz, 3.62H, Me), 1.66-1.78 (m, 2.98H, CH_2), 1.88-2.00 (m, 4.76H, CH_2), 2.09-2.16 (m, 1.57H, CH), 2.45 (dd, *J* = 15.0, 5.1 Hz, 1.30H, CH), 4.68 (d, *J* = 1.5 Hz, 0.1H, olefin, diastereomer B), 5.34-5.36 (m, 1H, olefin, diastereomer A), 11.09 (br s, 1.57H, CO_2H). **^{13}C NMR** (101 MHz, $CDCl_3$): δ = 16.4, 16.8, 20.3, 20.4, 20.8, 20.9, 22.0, 23.5, 24.7, 25.7, 27.0, 27.9, 29.3, 30.5, 30.7, 30.8, 30.9, 31.3, 33.5, 34.1, 34.3, 34.5, 34.6, 34.8, 34.9, 38.3, 38.5, 39.1, 39.4, 40.2, 44.4, 51.4, 120.6, 120.7, 134.1, 134.2, 180.4, 180.5. **HRMS-EI** (m/z) $[M-H]^-$ calcd. for $C_{11}H_{17}O_2$ 181.1234 found 181.1234.

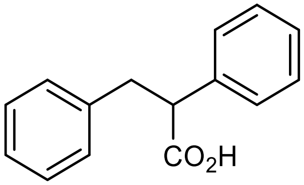

***2,3-Diphenylpropanoic acid*, 2p.**[6] Starting from 0.555 mmol of corresponding olefin the product was obtained as a white solid, m.p. = 78-80°C, yield 81% (0.102 g, 2MeTHF), 65% (0.082 g, Euc), 69% (0.086 g, Acetal), 65% (0.082 g, Me_2Isos), 83% (0.104 g, dioxane). **^{1}H NMR** (400 MHz, $CDCl_3$): δ = 3.04 (dd, *J* = 13.8, 7.0 Hz, 1H, CH_2), 3.41 (dd, *J* = 13.8, 8.4 Hz, 1H, CH_2), 3.86 (dd, *J* = 8.4, 7.0 Hz, 1H, CH), 7.09-7.11 (m, 2H, Ar), 7.15-7.24 (m, 3H, Ar), 7.27-7.31 (m, 5H, Ar), 10.77 (br s, 1H, CO_2H). **^{13}C NMR** (101 MHz, $CDCl_3$): δ = 39.4, 53.6, 126.6, 127.8, 128.3, 128.5, 128.9, 129.1, 138.1, 138.8, 179.7. **HRMS-EI** (m/z) $[M-H]^-$ calcd. for $C_{15}H_{13}O_2$ 225.0921 found 225.0919.

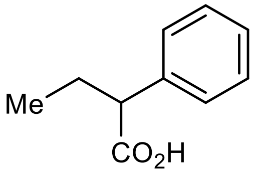

***2-Phenylbutanoic acid,* 2q.**[6] Starting from 1.692 mmol of corresponding olefin the product was obtained as a white solid, m.p. = 39-41°C, yield 81% (0.225 g, 2MeTHF). **^{1}H NMR** (400 MHz, $CDCl_3$): δ = 0.92 (t, *J* = 7.4 Hz, 3H, Me), 1.78-1.88 (m, 1H, CH_2), 2.12 (dt, *J* = 13.7, 7.4 Hz, 1H, CH_2), 3.47 (t, *J* = 7.7 Hz, 1H, CH), 7.27-7.36 (m, 5H, Ar), 11.27 (br s, 1H, CO_2H). **^{13}C NMR** (101 MHz, $CDCl_3$): δ = 12.3, 26.5, 53.5, 127.6, 128.1, 128.3, 128.8, 138.5, 180.6. **HRMS-EI** (m/z) $[M-H]^-$ calcd. for $C_{10}H_{11}O_2$ 163.0765 found 163. 0766.

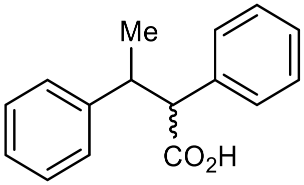

***2,3-Diphenylbutanoic acid*, mixture of diastereomers dr = 100:6 (NMR), 2r.**[8] Starting from 1.029 mmol of corresponding olefin the product was obtained as a white solid, m.p. = 128-130°C, yield 48% (0.118 g, 2MeTHF). **^{1}H NMR** (400 MHz, $CDCl_3$): δ = 1.06 (d, *J* = 7.0 Hz, 0.17H, Me, diastereomer B), 1.48 (d, *J* = 6.9 Hz, 3H, Me, diastereomer A), 3.50 (dq, *J* = 11.2, 6.8 Hz, 1H, CH, diastereomer A), 3.76 (d, *J* = 11.1 Hz, 1H, CH, diastereomer A), 7.01-7.21 (m, 9.46H, Ar, diastereomer A & B), 7.30-7.51 (m, 0.60H, Ar, diastereomer B), 10.61 (br s, 1.06H, CO_2H, diastereomer A & B). **^{13}C NMR** (101 MHz, $CDCl_3$): δ = 21.3, 43.2, 43.6, 59.4, 126.4, 126.9, 127.4, 127.5, 127.7, 128.3, 128.4, 128.7, 128.8, 128.9, 129.0, 137.1, 143.4, 180.0. **HRMS-EI** (m/z) $[M-H]^-$ calcd. for $C_{16}H_{15}O_2$ 239.1078 found 239.1075.

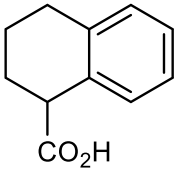

***1,2,3,4-Tetrahydronaphthalene-1-carboxylic acid,* 2s.**[8] Starting from 1.540 mmol of corresponding olefin the product was obtained as a white solid, m.p. = 77-79°C, yield 82% (0.223 g, 2MeTHF). **^{1}H NMR** (400 MHz, $CDCl_3$): δ = 1.80-1.91 (m, 1H, CH_2), 1.99-2.13 (m, 2H, CH_2), 2.21-2.30 (m, 1H, CH_2), 2.78-2.94 (m, 2H, CH_2), 3.91 (t, *J* = 5.7 Hz, 1H, CH), 7.16-7.31 (m, 4H, Ar), 10.92 (br s, 1H, CO_2H). **^{13}C NMR** (101 MHz, $CDCl_3$): δ = 20.6, 26.6, 29.2, 44.6, 126.0, 127.0, 127.3, 129.6, 129.8, 132.7, 137.5, 181.7. **HRMS-EI** (m/z) $[M-H]^-$ calcd. for $C_{11}H_{11}O_2$ 175.0765 found 175.0764.

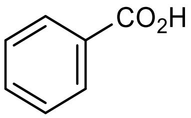

***Benzoic acid,* 4a.**[9] Starting from 0.735 mmol of corresponding boronic acid pinacol ester the product was obtained as a white solid, m.p. = 123-125°C, yield 85% (0.076 g, Me_2Isos), 74% (0.0667 g, 2MeTHF), 73% (0.0656 g, RoseOx). In case of carboxylation of triethoxyphenylsilane (0.832 mmol) the product was obtained as a white solid, m.p. = 123-125°C, yield 16% (0.016 g, 2MeTHF), 42% (0.043 g, GVL), 36% (0.037 g, Et_2Suc), 62% (0.063 g, DMA). **^{1}H NMR** (400 MHz, $CDCl_3$): δ = 7.49 (t, *J* = 7.8 Hz, 2H, Ar), 7.60-7.65 (m, 1H, Ar), 8.13-8.15 (m, 2H, Ar), 11.74 (br s, 1H, CO_2H). **^{13}C NMR** (101 MHz, $CDCl_3$): δ = 128.7, 129.5, 130.4, 134.0, 172.6. **HRMS-EI** (m/z) $[M-H]^-$ calcd. for $C_7H_5O_2$ 121.0295 found 121.0298.

---

[8] A. Gevorgyan, M. F. Obst, Y. Guttormsen, F. Maseras, K. H. Hopmann and A. Bayer, *Chem. Sci.,* 2019, **10**, 10072-10078.
[9] J. Takaya, S. Tadami, K. Ukai and N. Iwasawa, *Org. Lett.,* 2008, **10**, 2697-2700.

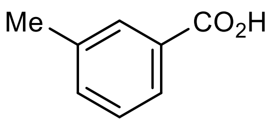

***3-Methylbenzoic acid*, 4b.**[10] Starting from 0.917 mmol of corresponding boronic acid pinacol ester the product was obtained as a white solid, m.p. = 108-110°C, yield 78% (0.097 g, Me_2Isos). **^{1}H NMR** (400 MHz, $CDCl_3$): δ = 2.43 (s, 3H, Me), 7.35-7.39 (m, 1H, Ar), 7.43-7.45 (m, 1H, Ar), 7.94-7.96 (m, 2H, Ar), 12.39 (br s, 1H, CO_2H). **^{13}C NMR** (101 MHz, $CDCl_3$): δ = 21.4, 127.6, 128.6, 129.5, 130.9, 134.8, 138.5, 173.0. **HRMS-EI** (m/z) $[M-H]^-$ calcd. for $C_8H_7O_2$ 135.0452 found 135.0455.

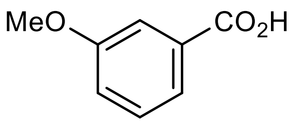

***3-Methoxybenzoic acid*, 4c.**[11] Starting from 0.854 mmol of corresponding boronic acid pinacol ester the product was obtained as a white solid, m.p. = 105-107°C, yield 98% (0.127 g, Me_2Isos). **^{1}H NMR** (400 MHz, $CDCl_3$): δ = 3.87 (s, 3H, OMe), 7.15-7.18 (m, 1H, Ar), 7.38 (t, J = 7.9 Hz, 1H, Ar), 7.63-7.64 (m, 1H, Ar), 7.72-7.74 (m, 1H, Ar), 11.80 (br s, 1H, CO_2H). **^{13}C NMR** (101 MHz, $CDCl_3$): δ = 55.6, 114.6, 120.6, 122.9, 129.7, 130.8, 159.8, 172.4. **HRMS-EI** (m/z) $[M-H]^-$ calcd. for $C_8H_7O_3$ 151.0401 found 151.0404.

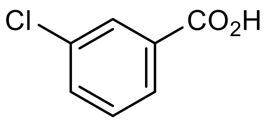

***3-Chlorobenzoic acid*, 4d.**[10] Starting from 0.839 mmol of corresponding boronic acid pinacol ester the product was obtained as a white solid, m.p. = 153-155°C, yield 82% (0.107 g, Me_2Isos). **^{1}H NMR** (400 MHz, MeOH-d4): δ = 7.45 (t, J = 7.9 Hz, 1H, Ar), 7.58 (ddd, J = 8.0, 2.2, 1.1 Hz, 1H, Ar), 7.92-7.97 (m, 2H, Ar). **^{13}C NMR** (101 MHz, MeOH-d4): δ = 129.1, 130.6, 131.2, 134.0, 134.1, 135.6, 168.4. **HRMS-EI** (m/z) $[M-H]^-$ calcd. for $C_7H_4ClO_2$ 154.9905 found 154.9907.

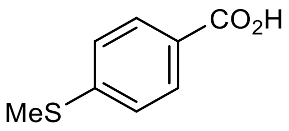

***4-(Methylthio)benzoic acid*, 4e.**[12] Starting from 0.799 mmol of corresponding boronic acid pinacol ester the product was obtained as a white solid, m.p. = 192-194°C, yield 71% (0.095 g, Me_2Isos). **^{1}H NMR** (400 MHz, MeOH-d4): δ = 2.52 (s, 3H, SMe), 7.30 (d, J = 8.6 Hz, 2H, Ar), 7.91 (d, J = 8.6 Hz, 2H, Ar). **^{13}C NMR** (101 MHz, MeOH-d4): δ = 14.8, 126.1, 127.9, 131.2, 147.4, 169.7. **HRMS-EI** (m/z) $[M-H]^-$ calcd. for $C_8H_7O_2S$ 167.0172 found 167.0172.

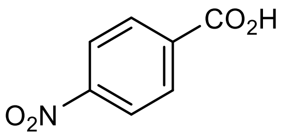

***4-Nitrobenzoic acid*, 4f.**[9] Starting from 0.803 mmol of corresponding boronic acid pinacol ester the product was obtained as a white solid, m.p. = above 200°C, yield 68% (0.091 g, Me_2Isos). **^{1}H NMR** (400 MHz, MeOH-d4): δ = 8.21-8.24 (m, 2H, Ar), 8.30-8.33 (m, 2H, Ar). **^{13}C NMR** (101 MHz, MeOH-d4): δ = 124.6, 132.0, 152.1, 167.7. **HRMS-EI** (m/z) $[M-H]^-$ calcd. for $C_7H_4NO_4$ 166.0146 found 166.0146.

---

[10] T. Osako, R. Kaiser, K. Torii and Y. Uozumi, *Synlett,* 2019, **30**, 961-966.
[11] Y. Makida, E. Marelli, A. M. Z. Slawin and S. P. Nolan, *Chem. Commun.,* 2014, **50**, 8010-8013.
[12] S. D. Friis, T. L. Andersen and T. Skrydstrup, *Org. Lett.,* 2013, **15**, 1378-1381.

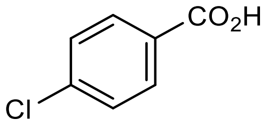

***4-Chlorobenzoic acid*, 4g.**[10] Starting from 0.839 mmol of corresponding boronic acid pinacol ester the product was obtained as a white solid, m.p. = above 200°C, yield 85% (0.111 g, Me_2Isos), 72% (0.094 g, 2MeTHF), 52% (0.068 g, RoseOx). **^{1}H NMR** (400 MHz, MeOH-d4): δ = 7.46-7.49 (m, 2H, Ar), 7.97-8.01 (m, 2H, Ar). **^{13}C NMR** (400 MHz, MeOH-d4): δ = 129.8, 132.4, 140.4, 168.8. **HRMS-EI** (m/z) $[M-H]^-$ calcd. for $C_7H_4ClO_2$ 154.9905 found 154.9908.

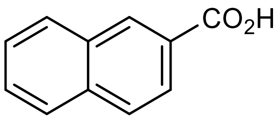

***2-Naphthoic acid*, 4h.**[10] Starting from 0.787 mmol of corresponding boronic acid pinacol ester the product was obtained as a white solid, m.p. = 185-187°C, yield 81% (0.110 g, Me_2Isos). **^{1}H NMR** (400 MHz, $CDCl_3$): δ = 7.61 (dddd, J = 22.1, 8.1, 6.9, 1.4 Hz, 2H, Ar), 7.90-7.94 (m, 2H, Ar), 7.99-8.01 (m, 1H, Ar), 8.14 (dd, J = 8.6, 1.7 Hz, 1H, Ar), 8.74 (m, 1H, Ar). **^{13}C NMR** (400 MHz, $CDCl_3$): δ = 125.6, 126.7, 127.0, 128.0, 128.5, 128.9, 129.8, 132.4, 132.7, 136.2, 172.5. **HRMS-EI** (m/z) $[M-H]^-$ calcd. for $C_{11}H_7O_2$ 171.0452 found 171.0453.

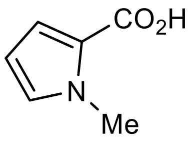

***1-Methyl-1H-pyrrole-2-carboxylic acid*, 4i.**[13] Starting from 0.966 mmol of corresponding boronic acid pinacol ester the product was obtained as a white solid, m.p. = 136-138°C, yield 41% (0.049 g, Me_2Isos). **^{1}H NMR** (400 MHz, $CDCl_3$): δ = 3.93 (s, 3H, Me), 6.15 (dd, J = 4.0, 2.5 Hz, 1H, pyrrole), 6.84 (t, J = 2.2 Hz, 1H, pyrrole), 7.10 (dd, J = 4.0, 1.8 Hz, 1H, pyrrole). **^{13}C NMR** (400 MHz, $CDCl_3$): δ = 37.2, 108.5, 120.1, 121.9, 130.9, 166.3. **HRMS-EI** (m/z) $[M-H]^-$ calcd. for $C_6H_6NO_2$ 124.0404 found 124.0408.

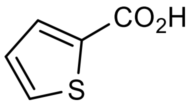

***Thiophene-2-carboxylic acid*, 4j.**[12] Starting from 0.952 mmol of corresponding boronic acid pinacol ester the product was obtained as a white solid, m.p. = 125-127°C, yield 78% (0.095 g, Me_2Isos), 84% (0.103 g, 2MeTHF), 59% (0.072 g, RoseOx). **^{1}H NMR** (400 MHz, $CDCl_3$): δ = 7.15 (dd, J = 5.0, 3.7 Hz, 1H, thiophene), 7.65 (dd, J = 4.9, 1.3 Hz, 1H, thiophene), 7.91 (dd, J = 3.8, 1.3 Hz, 1H, thiophene), 10.98 (br s, 1H, CO_2H). **^{13}C NMR** (101 MHz, $CDCl_3$): δ = 128.3, 133.1, 134.2, 135.2, 168.0. **HRMS-EI** (m/z) $[M-H]^-$ calcd. for $C_5H_3O_2S$ 126.9859 found 126.9863.

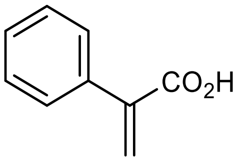

***2-Phenylacrylic acid*, 4k.**[11] Starting from 0.869 mmol of corresponding boronic acid pinacol ester the product was obtained as a white solid, m.p. = 104-106°C, yield 89% (0.114 g, Me_2Isos), 85% (0.109 g, 2MeTHF), 43% (0.055 g, RoseOx). **^{1}H NMR** (400 MHz, $CDCl_3$): δ = 6.04 (d, J = 1.2 Hz, 1H, acrylic acid), 6.56 (d, J = 1.2 Hz, 1H, acrylic acid), 7.36-7.41 (m, 3H, Ar), 7.44-7.48 (m, 2H, Ar), 9.89 (br s, 1H, CO_2H). **^{13}C NMR** (101 MHz, $CDCl_3$): δ = 128.3, 128.5, 128.6, 129.6, 136.3, 140.8, 172.3. **HRMS-EI** (m/z) $[M-H]^-$ calcd. for $C_9H_7O_2$ 147.0452 found 147.0456.

---

[13] S. Korsager, R. H. Taaning and T. Skrydstrup, *J. Am. Chem. Soc.*, 2013, **135**, 2891-2894.

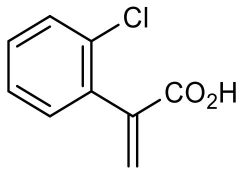

***2-(2-Chlorophenyl)acrylic acid*, 4l.**[14] Starting from 0.756 mmol of corresponding boronic acid pinacol ester the product was obtained as a yellowish viscous oil, yield 62% (0.086 g, Me_2Isos). **^{1}H NMR** (400 MHz, $CDCl_3$): δ = 6.20 (d, *J* = 1.2 Hz, 1H, acrylic acid), 6.94 (d, *J* = 1.2 Hz, 1H, acrylic acid), 7.54-7.60 (m, 3H, Ar), 7.67-7.69 (m, 1H, Ar). **^{13}C NMR** (101 MHz, $CDCl_3$): δ = 126.9, 129.5, 129.9, 131.1, 131.5, 133.6, 136.1, 139.7, 171.1. **HRMS-EI** (m/z) $[M-H]^-$ calcd. for $C_9H_6ClO_2$ 181.0062 found 181.0064.

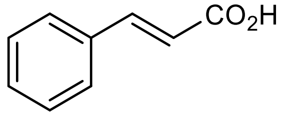

***Cinnamic acid*, 4m.**[9] Starting from 0.869 mmol of corresponding boronic acid pinacol ester the product was obtained as a white solid, m.p. = 132-134°C, yield 65% (0.084 g, Me_2Isos). **^{1}H NMR** (400 MHz, $CDCl_3$): δ = 6.47 (d, *J* = 16.0 Hz, 1H, acrylic acid), 7.38-7.44 (m, 3H, Ar), 7.54-7.59 (m, 2H, Ar), 7.81 (d, *J* = 16.0 Hz, 1H, acrylic acid), 10.13 (br s, 1H, CO_2H). **^{13}C NMR** (101 MHz, $CDCl_3$): δ = 117.5, 128.6, 129.1, 130.9, 134.2, 147.3, 172.7. **HRMS-EI** (m/z) $[M-H]^-$ calcd. for $C_9H_7O_2$ 147.0452 found 147.0454.

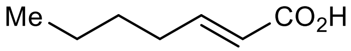

***(E)-Hept-2-enoic acid*, 4n.**[15] Starting from 0.952 mmol of corresponding boronic acid pinacol ester the product was obtained as a colourless viscous oil, yield 84% (0.102 g, Me_2Isos). **^{1}H NMR** (400 MHz, $CDCl_3$): δ = 0.90 (t, *J* = 7.3 Hz, 3H, Me), 1.29-1.38 (m, 2H, CH_2), 1.40-1.48 (m, 2H, CH_2), 2.22 (qd, *J* = 7.1, 1.6 Hz, 2H, CH_2), 5.81 (dt, *J* = 15.6, 1.6 Hz, 1H, acrylic acid), 7.07 (dt, *J* = 15.6, 7.0 Hz, 1H, acrylic acid), 9.19 (br s, 1H, CO_2H). **^{13}C NMR** (101 MHz, $CDCl_3$): δ = 13.9, 22.4, 30.1, 32.1, 120.8, 152.5, 172.2. **HRMS-EI** (m/z) $[M-H]^-$ calcd. for $C_7H_{11}O_2$ 127.0765 found 127.0768.

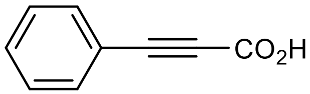

***3-Phenylpropiolic acid*, 4o.**[16] Starting from 0.877 mmol of corresponding boronic acid pinacol ester the product was obtained as a white solid, m.p. = 135-137°C, yield 62% (0.079 g, Me_2Isos), 63% (0.081 g, 2MeTHF), 21% (0.027 g, RoseOx). In case of direct C-H carboxylation of phenylacetylene (1.960 mmol) the product was obtained as a white solid, m.p. = 135-137°C, yield 20% (0.056 g, Cu-free, 2MeTHF), 31% (0.090 g, Cu-free, GVL), 76% (0.217 g, Cu-catalyzed, 2MeTHF), 63% (0.181 g, Cu-catalyzed, Acetal), 27% (0.077 g, Cu-catalyzed, GVL), 38% (0.108 g, Cu-catalyzed, THF). **^{1}H NMR** (400 MHz, $CDCl_3$): δ = 7.38-7.42 (m, 2H, Ar), 7.46-7.51 (m, 1H, Ar), 7.61-7.64 (m, 2H, Ar), 9.17 (br s, 1H, CO_2H). **^{13}C NMR** (101 MHz, $CDCl_3$): δ = 89.1, 119.3, 128.9, 131.3, 133.5, 158.4. **HRMS-EI** (m/z) $[M-H]^-$ calcd. for $C_9H_5O_2$ 145.0295 found 145.0315.

---

[14] Y. Fukata, K. Yao, R. Miyaji, K. Asano and S. Matsubara, *J. Org. Chem.,* 2017, **82**, 12655-12668.
[15] G. Wang, Y. Tang, Y. Zhang, X. Liu, L. Lin and X. Feng, *Chem. Eur. J.,* 2017, **23**, 554-557.
[16] Z. Wu, L. Sun, Q. Liu, X. Yang, X. Ye, Y. Hu and Y. Huang, *Green Chem.,* 2017, **19**, 2080-2085.

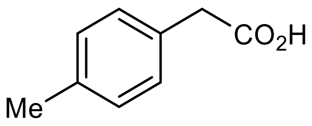

***2-(p-Tolyl)acetic acid*, 4p.**[17] Starting from 0.862 mmol of corresponding boronic acid pinacol ester the product was obtained as a white solid, m.p. = 88-90ºC, yield 67% (0.086 g, Me_2Isos), 87% (0.112 g, 2MeTHF), 53% (0.069 g, RoseOx). **^{1}H NMR** (400 MHz, $CDCl_3$): δ = 2.35 (s, 3H, Me), 3.62 (s, 2H, CH_2), 7.14-7.19 (m, 4H, Ar). **^{13}C NMR** (101 MHz, $CDCl_3$): δ = 21.3, 40.8, 129.4, 129.5, 130.4, 137.2, 178.4. **HRMS-EI** (m/z) $[M-H]^-$ calcd. for $C_9H_9O_2$ 149.0608 found 149.0611.

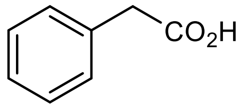

***2-Phenylacetic acid*, 4q.**[13] Starting from 0.917 mmol of corresponding boronic acid pinacol ester the product was obtained as a white solid, m.p. = 76-78ºC, yield 82% (0.102 g, 2MeTHF). **^{1}H NMR** (400 MHz, $CDCl_3$): δ = 3.66 (s, 2H, CH_2), 7.28-7.38 (m, 5H, Ar), 10.54 (br s, 1H, CO_2H). **^{13}C NMR** (101 MHz, $CDCl_3$): δ = 41.3, 127.5, 128.8, 129.5, 133.4, 178.2. **HRMS-EI** (m/z) $[M-H]^-$ calcd. for $C_8H_7O_2$ 135.0452 found 135.0455.

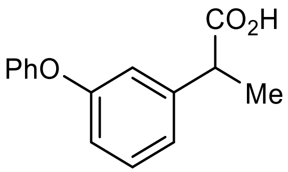

***2-(3-Phenoxyphenyl)propanoic acid, Fenoprofen*, 4r.**[18] Starting from 0.925 mmol of corresponding boronic acid pinacol ester the product was obtained as a colorless viscous oil, yield 60% (0.134 g, 2MeTHF). **^{1}H NMR** (400 MHz, MeOH-d4): δ = 1.42 (d, *J* = 7.2 Hz, 3H, Me), 3.68 (q, *J* = 7.1 Hz, 1H, CH), 6.82-6.85 (m, 1H, Ar), 6.95-9.98 (m, 3H, Ar), 7.04-7.11 (m, 2H, Ar), 7.24-7.35 (m, 3H, Ar). **^{13}C NMR** (101 MHz, MeOH-d4): δ = 25.1, 46.5, 118.3, 118.4, 119.1, 119.8, 120.0, 120.1, 123.4, 123.6, 124.5, 124.6, 130.9, 131.0, 131.1, 144.6, 158.6, 159.0, 177.9. **HRMS-EI** (m/z) $[M-H]^-$ calcd. for $C_{15}H_{13}O_3$ 241.0870 found 241.0870.

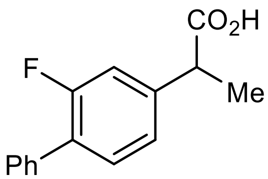

***2-(2-Fluoro-[1,1'-biphenyl]-4-yl)propanoic acid, Flurbiprofen*, 4s.**[2] Starting from 0.920 mmol of corresponding boronic acid pinacol ester the product was obtained as a white solid, m.p. = 110-112ºC, yield 53% (0.118 g, 2MeTHF). **^{1}H NMR** (400 MHz, MeOH-d4): δ = 1.48 (d, *J* = 7.1 Hz, 3H, Me), 3.76 (q, *J* = 7.3 Hz, 1H, CH), 7.13-7.20 (m, 2H, Ar), 7.31-7.35 (m, 1H, Ar), 7.37-7.43 (m, 3H, Ar), 7.48-7.51 (m, 2H, Ar). **^{13}C NMR** (101 MHz, MeOH-d4): δ = 25.1, 46.2, 116.2 (d, *J* = 23.9 Hz), 116.8, 124.9 (d, *J* = 3.3 Hz), 128.8, 128.9 (d, *J* = 2.6 Hz), 129.0, 129.5, 129.6, 130.0, 131.3, 131.9 (d, *J* = 3.9 Hz), 137.0, 144.2 (d, *J* = 7.7 Hz), 161.0 (d, *J* = 246.8 Hz), 177.7. **HRMS-EI** (m/z) $[M-H]^-$ calcd. for $C_{15}H_{12}FO_2$ 243.0827 found 243.0829.

[17] S. Kodumuri, S. Peraka, N. Mameda, D. Chevella, R. Banothu and N. Nama, *RSC Adv.,* 2016, **6**, 6719-6723.
[18] M. Gaydou, T. Moragas, F. Julia-Hernandez and R. Martin, *J. Am. Chem. Soc.,* 2017, **139**, 12161-12164.

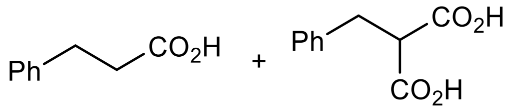

***3-Phenylpropanoic acid* 2b : *2-benzylmalonic acid* 6a, 0.4:1 ratio (NMR).**[6] Starting from 1.470 mmol of phenylacetylene the product was obtained as a a colourless viscous oil, 0.194 g (2MeTHF). **^{1}H NMR** (400 MHz, $CDCl_3$): δ = 2.70-2.81 (m, 0.90H, CH_2, **2b**), 3.01-3.05 (m, 0.80H, CH_2, **2b**), 3.33 (d, J = 7.6 Hz, 2H, CH_2, **6a**), 3.83 (t, J = 7.6 Hz, 1H, CH, **6a**), 7.27-7.39 (m, 7.46H, Ar, **2b**/**6a**), 11.35 (br s, 2.06H, CO_2H, **2b**/**6a**). **^{13}C NMR** (101 MHz, $CDCl_3$): δ = 30.7, 31.1, 34.6, 34.8, 35.7, 35.9, 52.0, 53.0, 53.7, 126.5, 126.6, 127.1, 127.2, 128.4, 128.7, 128.8, 128.9, 137.2, 137.5, 140.2, 140.5, 169.3, 174.4, 174.9, 179.8. **HRMS-EI** (m/z) $[M-H]^-$ calcd. for $C_9H_9O_2$ 149.0608, $C_{10}H_9O_4$ 193.0506 found 149.0611, 193.0508.

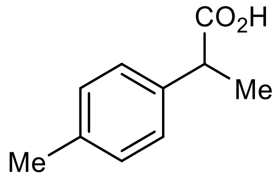

***2-(p-Tolyl)propanoic acid*, 7a.**[19] Starting from 5.080 mmol of 4-methylstyrene the product was obtained as a yellowish viscous oil, 27% (0.228 g, 2MeTHF), 22% (0.186 g, Acetal), 21% (0.174 g, Euc), 39% (0.325 g, THF). **^{1}H NMR** (400 MHz, $CDCl_3$): δ = 1.49 (d, J = 7.1 Hz, 3H, Me), 2.33 (s, 3H, Me), 3.70 (q, J = 7.2 Hz, 1H, CH), 7.14 (d, J = 7.9 Hz, 2H, Ar), 7.20-7.22 (m, 2H, Ar). **^{13}C NMR** (101 MHz, $CDCl_3$): δ = 18.3, 21.2, 45.1, 127.6, 129.5, 137.1, 137.2, 180.4. **HRMS-EI** (m/z) $[M-H]^-$ calcd. for $C_{10}H_{11}O_2$ 163.0765 found 163.0767.

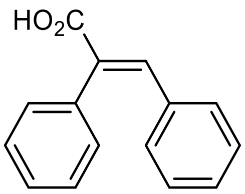

***(E)-2,3-Diphenylacrylic acid*, 9a.**[20] Starting from 1.120 mmol of diphenylacetylene the product was obtained as a white solid, m.p. = 172-174°C, Ni-catalyzed hydrocarboxylation - yield 21% (0.053 g, 2MeTHF), 49% (0.122 g, MeCN); Cu-catalyzed hydrocarboxylation - yield 41% (0.103 g, 2MeTHF), 61% (0.154 g, Acetal), 43% (0.107 g, Euc), 57% (0.144 g, dioxane); Fe-catalyzed hydrocarboxylation - yield 42% (0.106 g, 2MeTHF), 14% (0.036 g, Et_2O). **^{1}H NMR** (400 MHz, MeOH-d4): δ = 7.02-7.05 (m, 2H, Ar), 7.11-7.21 (m, 5H, Ar), 7.33-7.37 (m, 3H, Ar), 7.86 (s, 1H, acrylate). **^{13}C NMR** (101 MHz, MeOH-d4): δ = 128.8, 129.3, 129.7, 130.2, 131.0, 131.7, 136.2, 137.8, 141.5, 171.1. **HRMS-EI** (m/z) $[M-H]^-$ calcd. for $C_{15}H_{11}O_2$ 223.0765 found 223.0766.

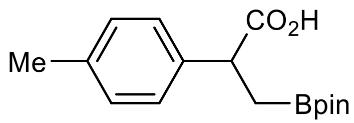

***3-(4,4,5,5-Tetramethyl-1,3,2-dioxaborolan-2-yl)-2-(p-tolyl)propanoic acid*, 10a.**[21] Starting from 1.690 mmol of 4-methylstyrene the product was obtained as a colourless viscous oil, yield 81% (0.397 g, 2MeTHF), 44% (0.218 g, Acetal), 85% (0.417 g, Euc), 78% (0.381 g, THF). **^{1}H NMR** (400 MHz, $CDCl_3$): δ = 1.15 (d, J = 6.6 Hz, 12H, pin), 1.22-1.28 (m, 1H, CH_2), 1.54 (dd, J = 16.0, 9.6 Hz, 1H, CH_2), 2.31 (s, 3H, Me), 3.83 (dd, J =

[19] Z. Huang, Y. Cheng, X. Chen, H.-F. Wang, C.-X. Dud and Y. Li, *Chem. Commun.*, 2018, **54**, 3967-3970.
[20] S. Li, W. Yuan and S. Ma, *Angew. Chem. Int. Ed.*, 2011, **50**, 2578-2582.
[21] T. W. Butcher, E. J. McClain, T. G. Hamilton, T. M. Perrone, K. M. Kroner, G. C. Donohoe, N. G. Akhmedov, J. L. Petersen and B. V. Popp, *Org. Lett.*, 2016, **18**, 6428-6431.

9.5, 7.0 Hz, 1H, CH), 7.10 (d, *J* = 7.9 Hz, 2H, Ar), 7.18-7.20 (m, 2H, Ar). **^{13}C NMR** (101 MHz, $CDCl_3$): δ = 21.2, 24.7, 24.8, 46.6, 50.8, 83.5, 127.8, 129.3, 136.9, 137.6, 180.9. **HRMS-EI** (m/z) $[M-H]^-$ calcd. for $C_{16}H_{22}BO_4$ 289.1617 found 289.1616.

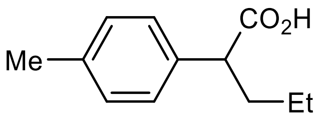

***2-(p-Tolyl)pentanoic acid*, 11a.**[22] Starting from 3.380 mmol of 4-methylstyrene the product was obtained as a yellowish viscous oil, yield 16% (0.105 g, 2MeTHF), 24% (0.155 g, Acetal), 20% (0.127 g, Euc), 28% (0.184 g, THF). **^{1}H NMR** (400 MHz, $CDCl_3$): δ = 0.82 (t, *J* = 7.3 Hz, 3H, Me), 1.57-1.68 (m, 1H, CH_2), 1.70-1.80 (m, 1H, CH_2), 2.35 (s, 3H, Me), 2.60-2.72 (m, 2H, CH_2), 2.96-3.03 (m, 1H, CH), 7.12 (q, *J* = 8.0 Hz, 4H, Ar), 10.37 (br s, 1H, CO_2H). **^{13}C NMR** (101 MHz, $CDCl_3$): δ = 12.0, 21.1, 29.3, 41.4, 43.2, 126.1, 127.5, 128.1, 129.3, 136.1, 140.7, 179.2. **HRMS-EI** (m/z) $[M-H]^-$ calcd. for $C_{12}H_{15}O_2$ 191.1078 found 191.1080.

[22] P. Shao, S. Wang, C. Chen and C. Xi, *Chem. Commun.*, 2015, **51**, 6640-6642.

Copies of spectra

Compound 3ra

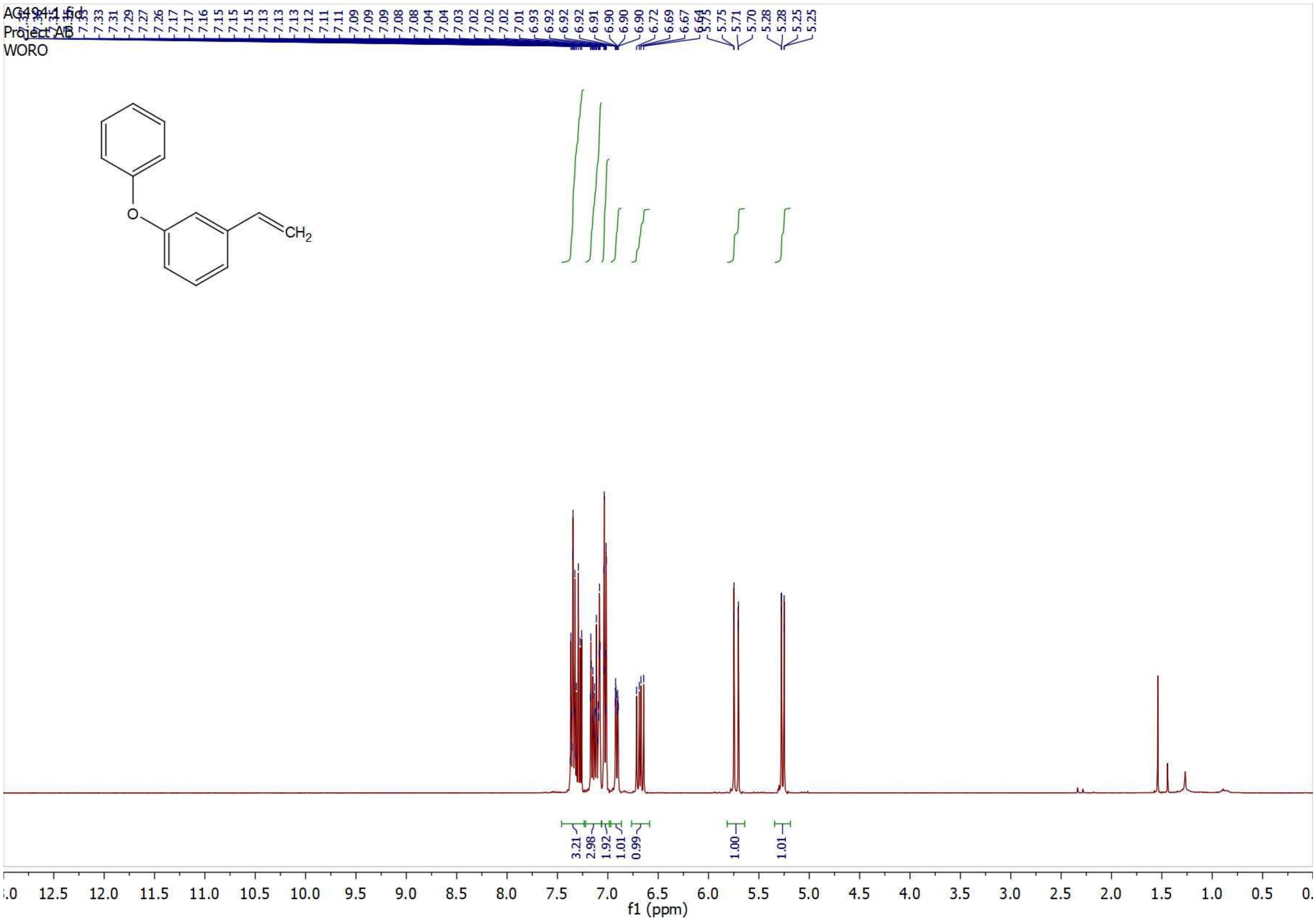

Compound 3ra

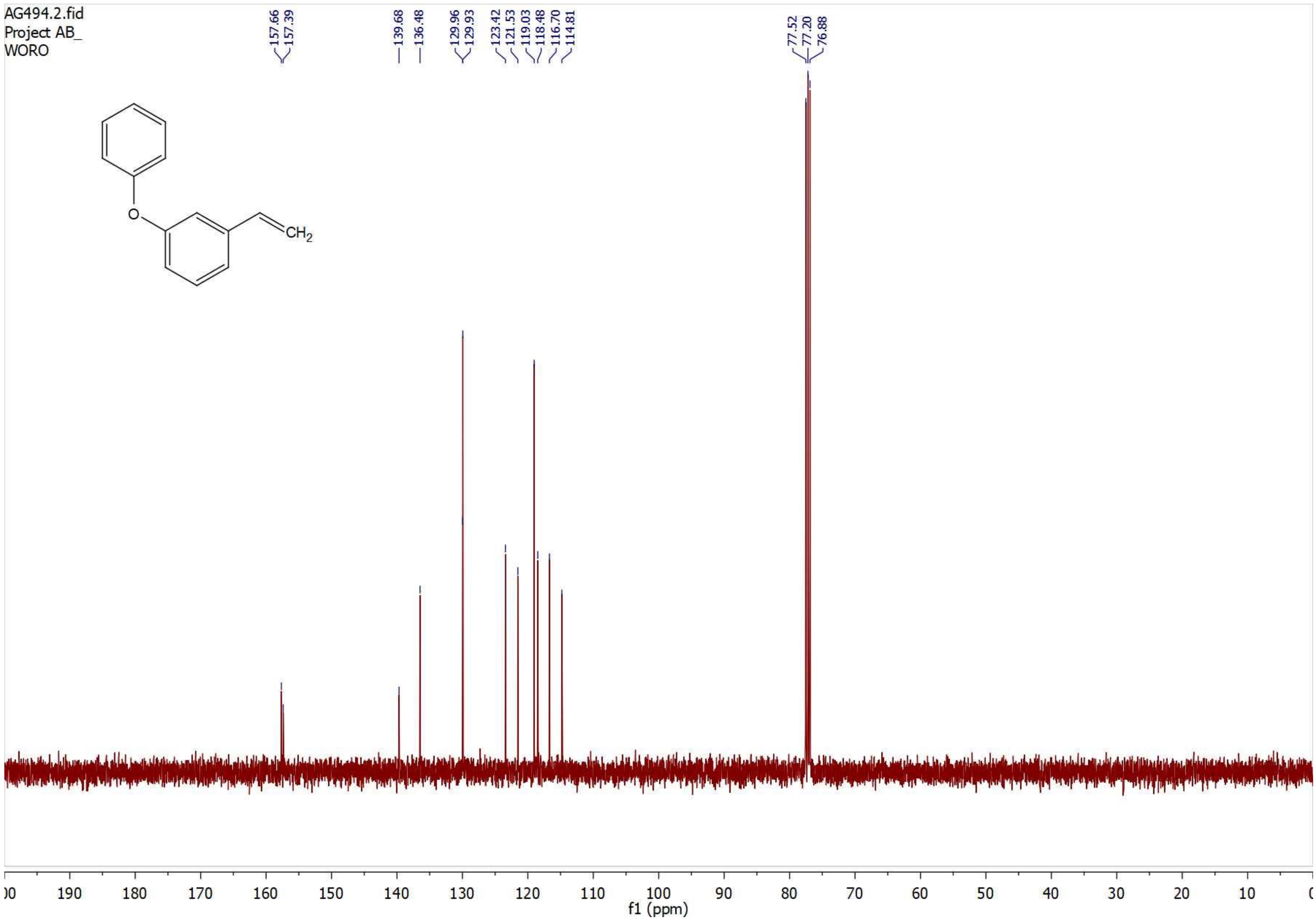

Compound 3sa

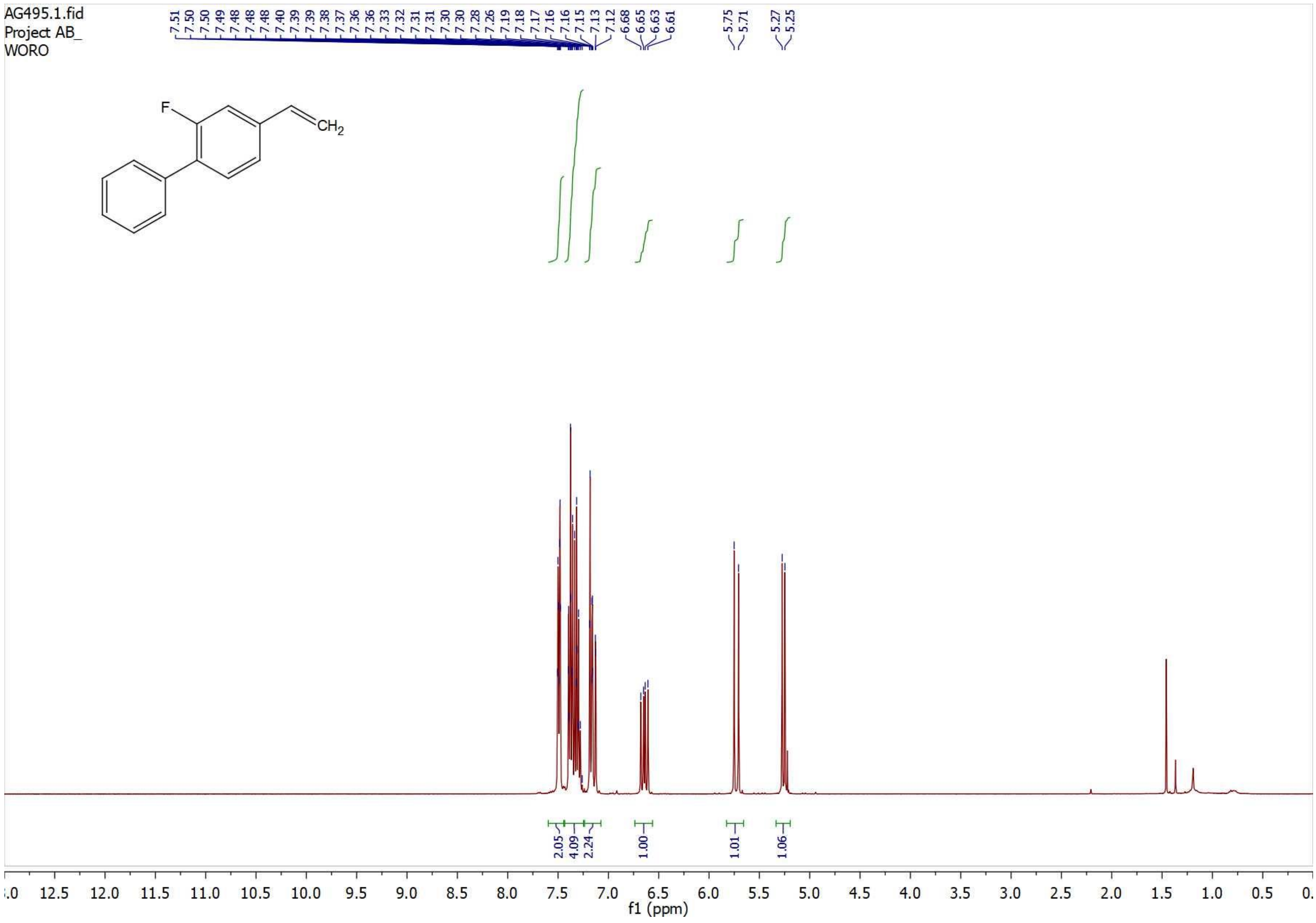

Compound 3sa

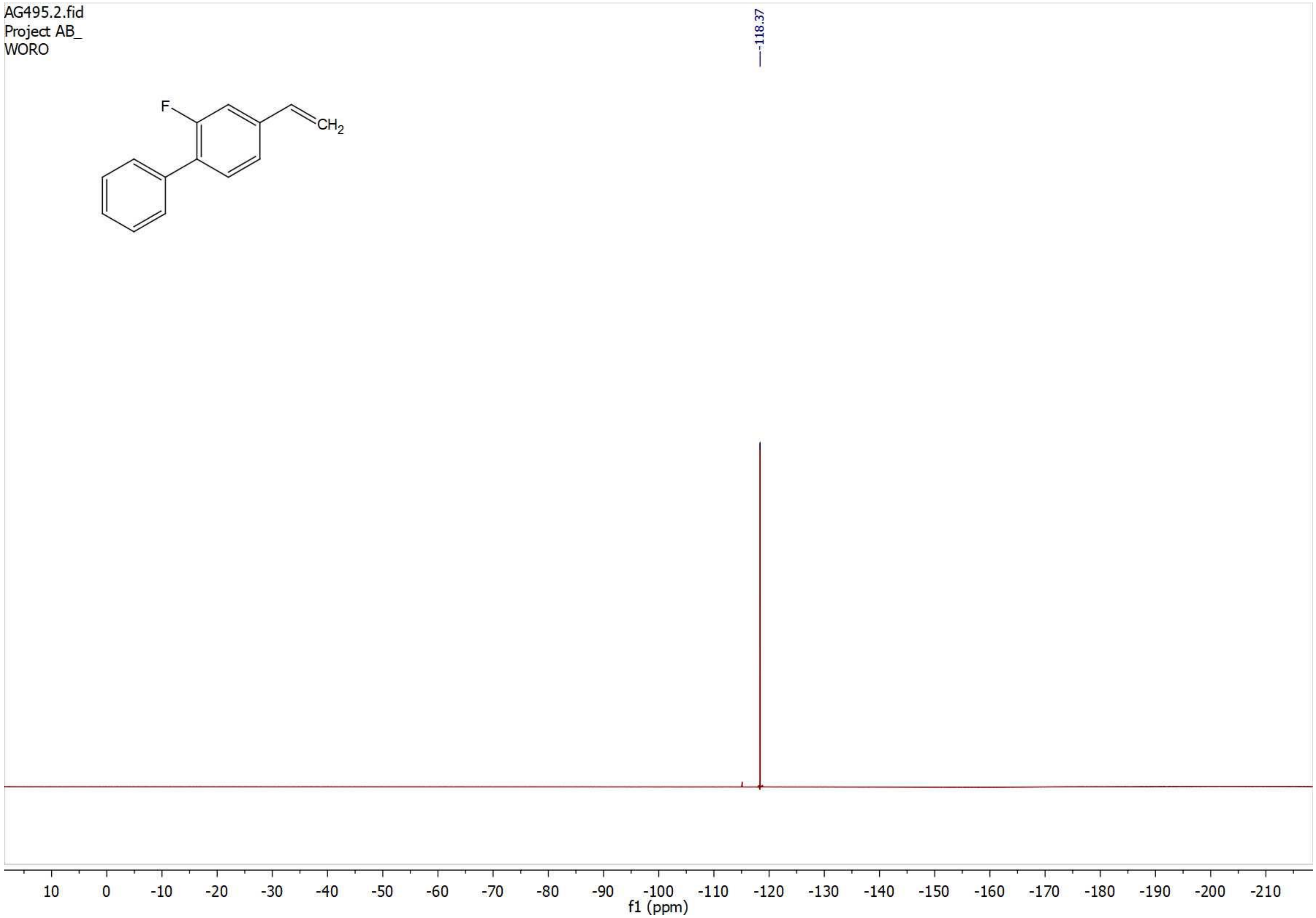

Compound 3sa

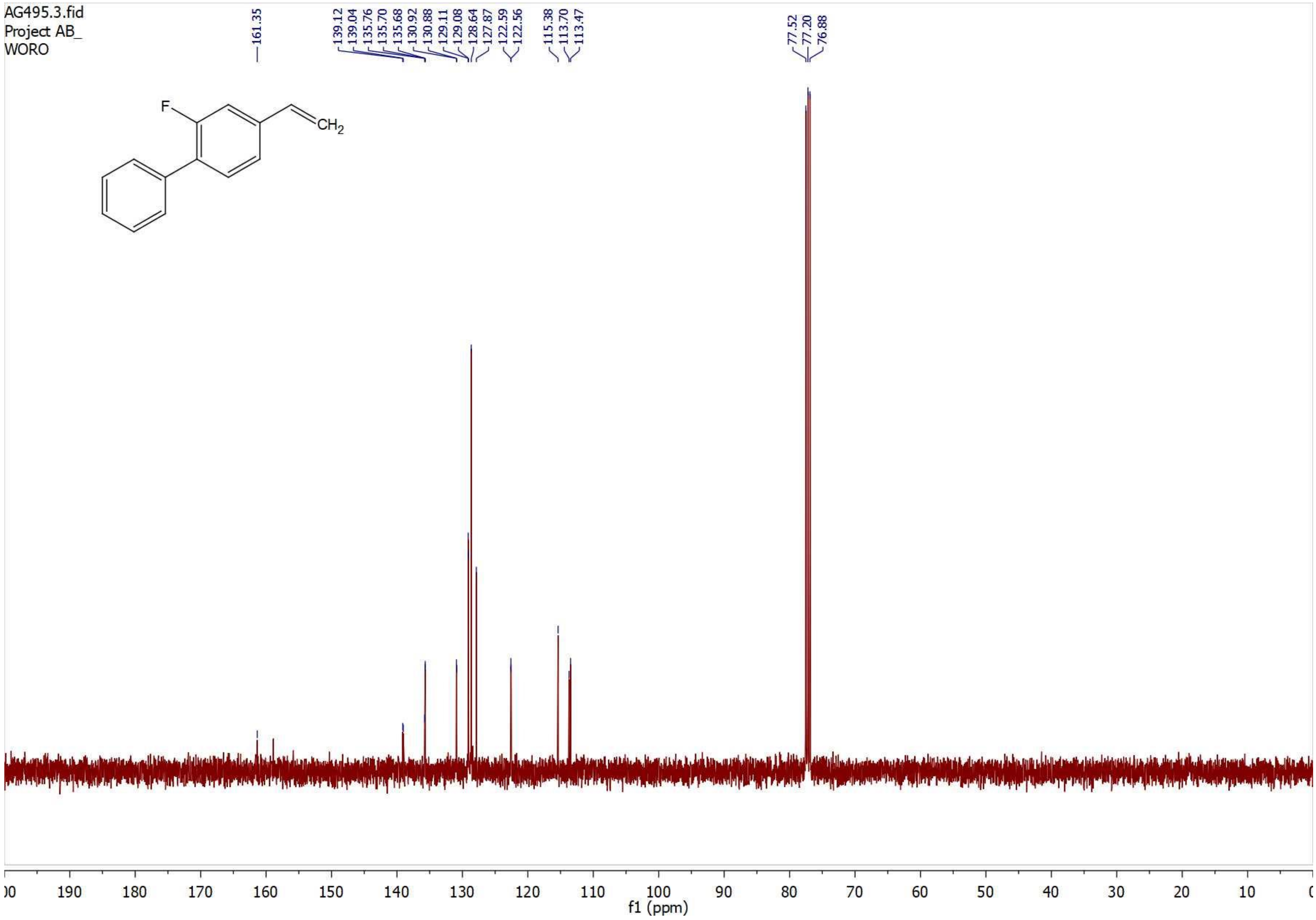

Compound 3rb

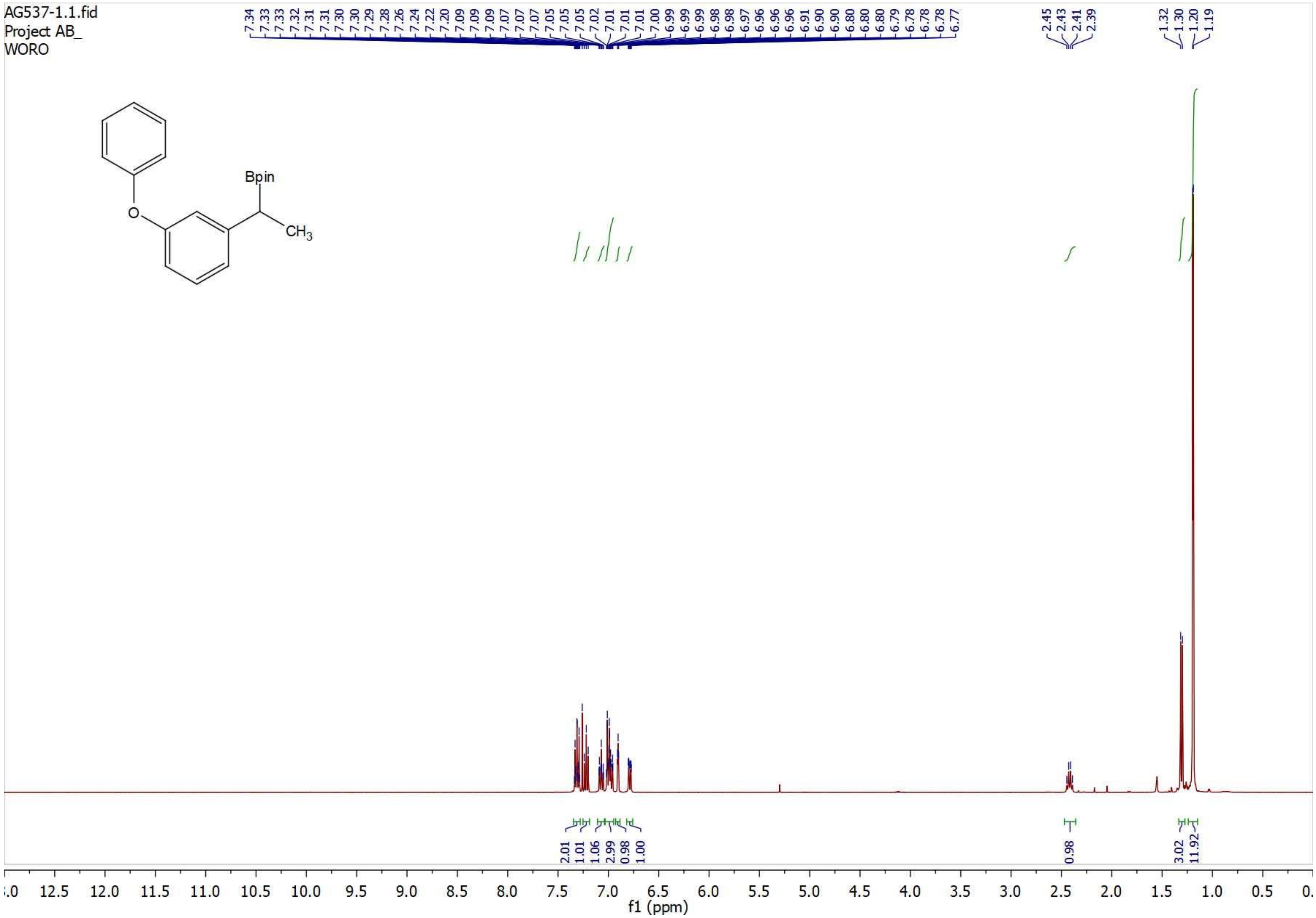

Compound 3rb

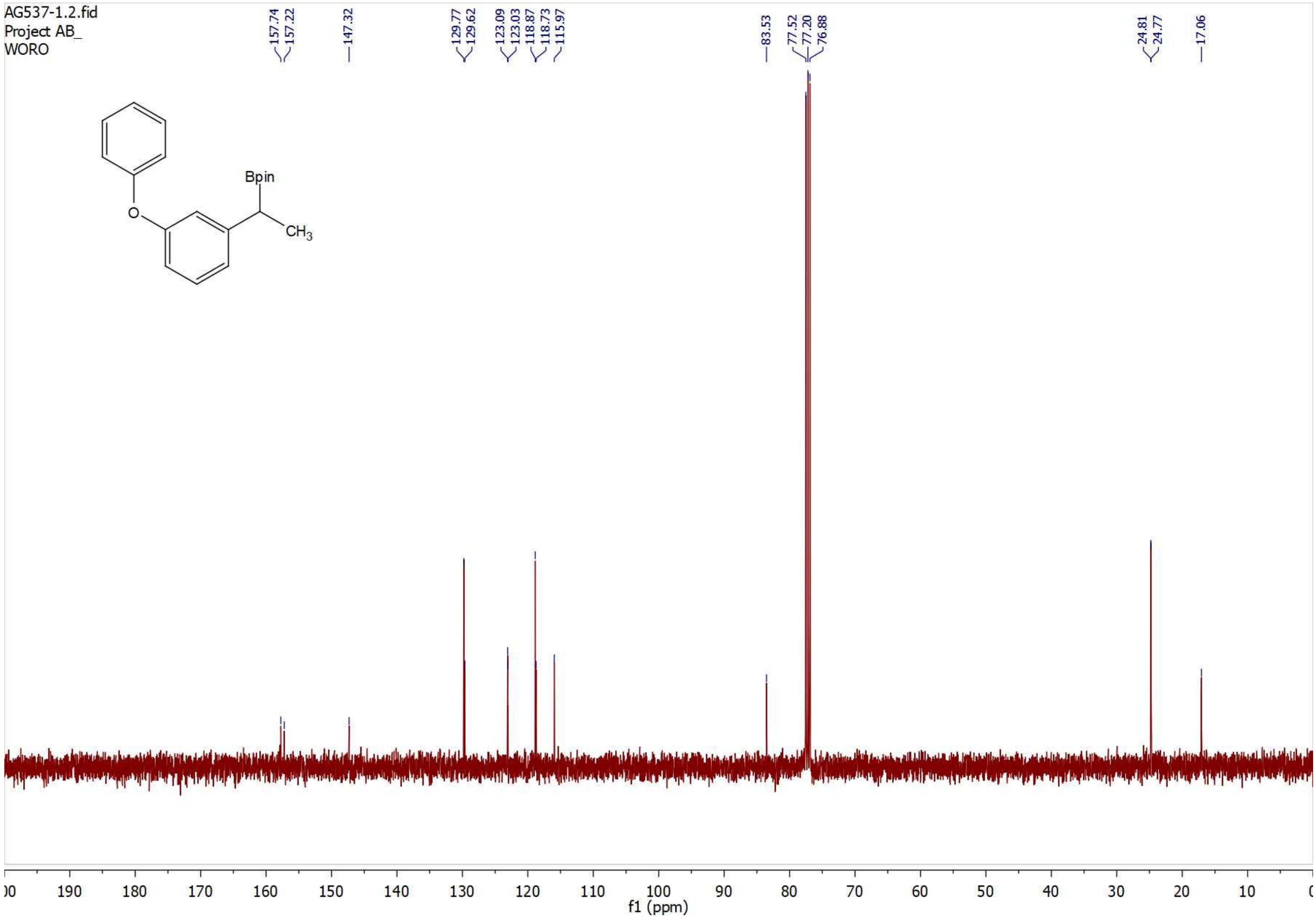

Compound 3sb

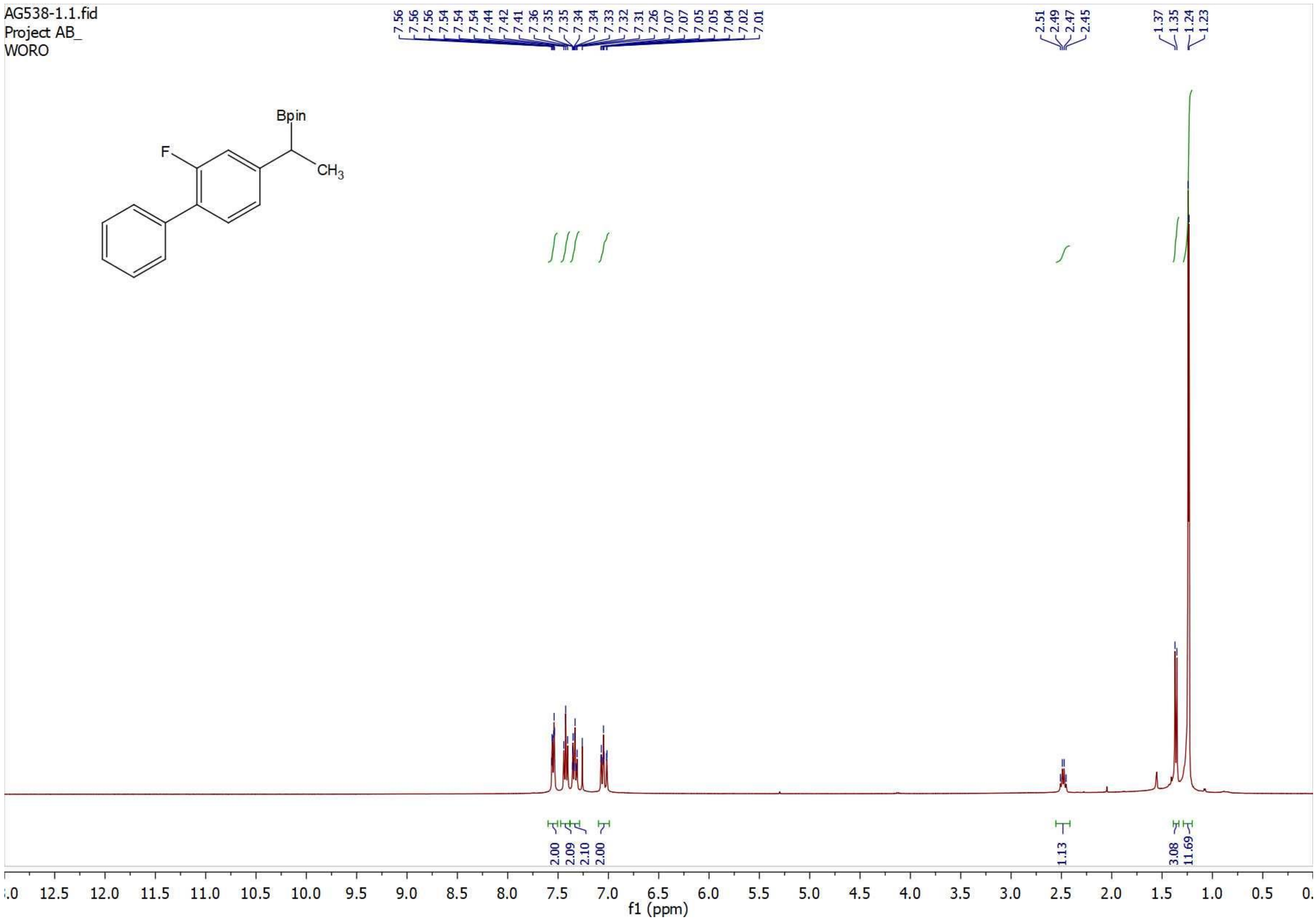

Compound 3sb

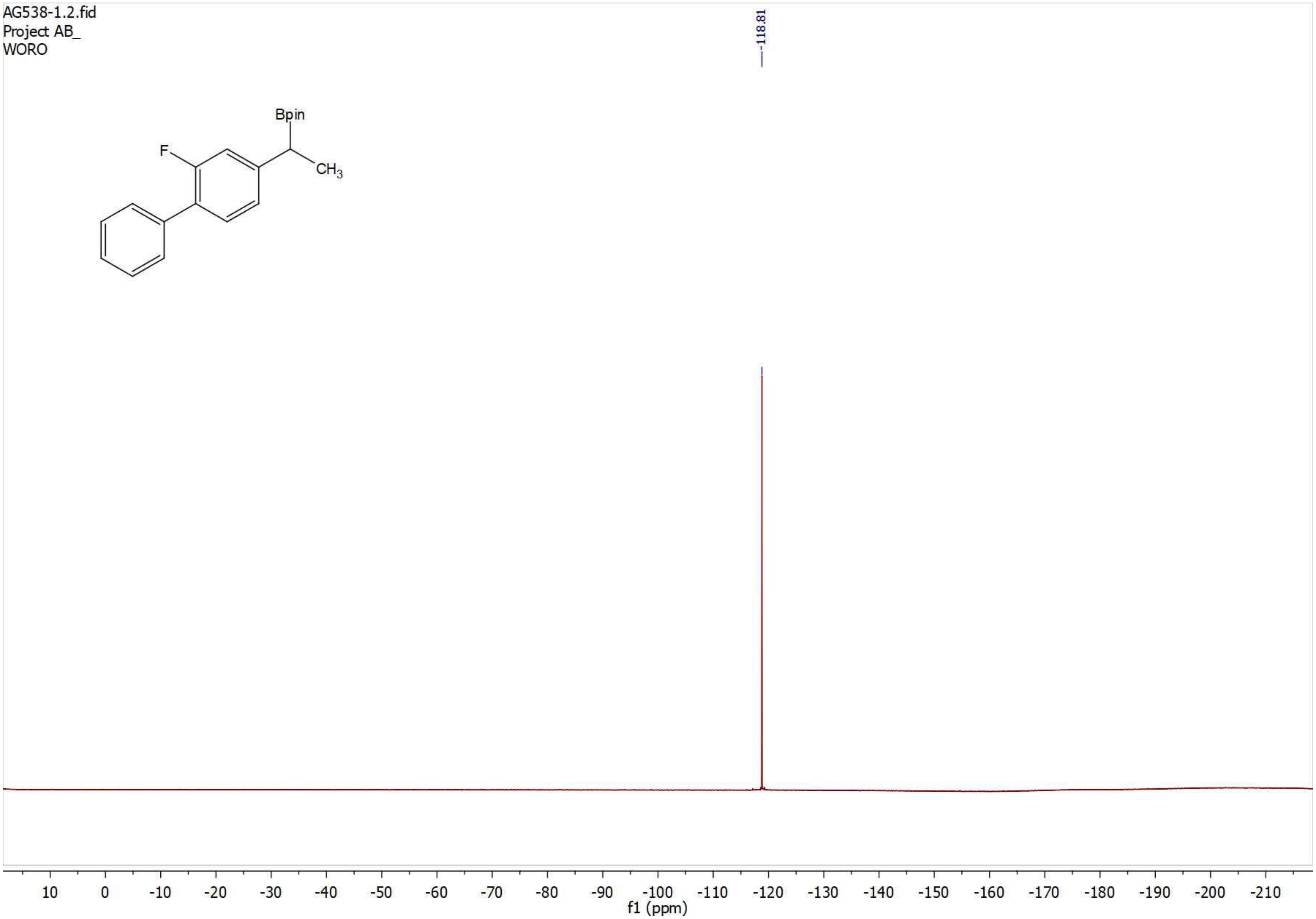

Compound 3sb

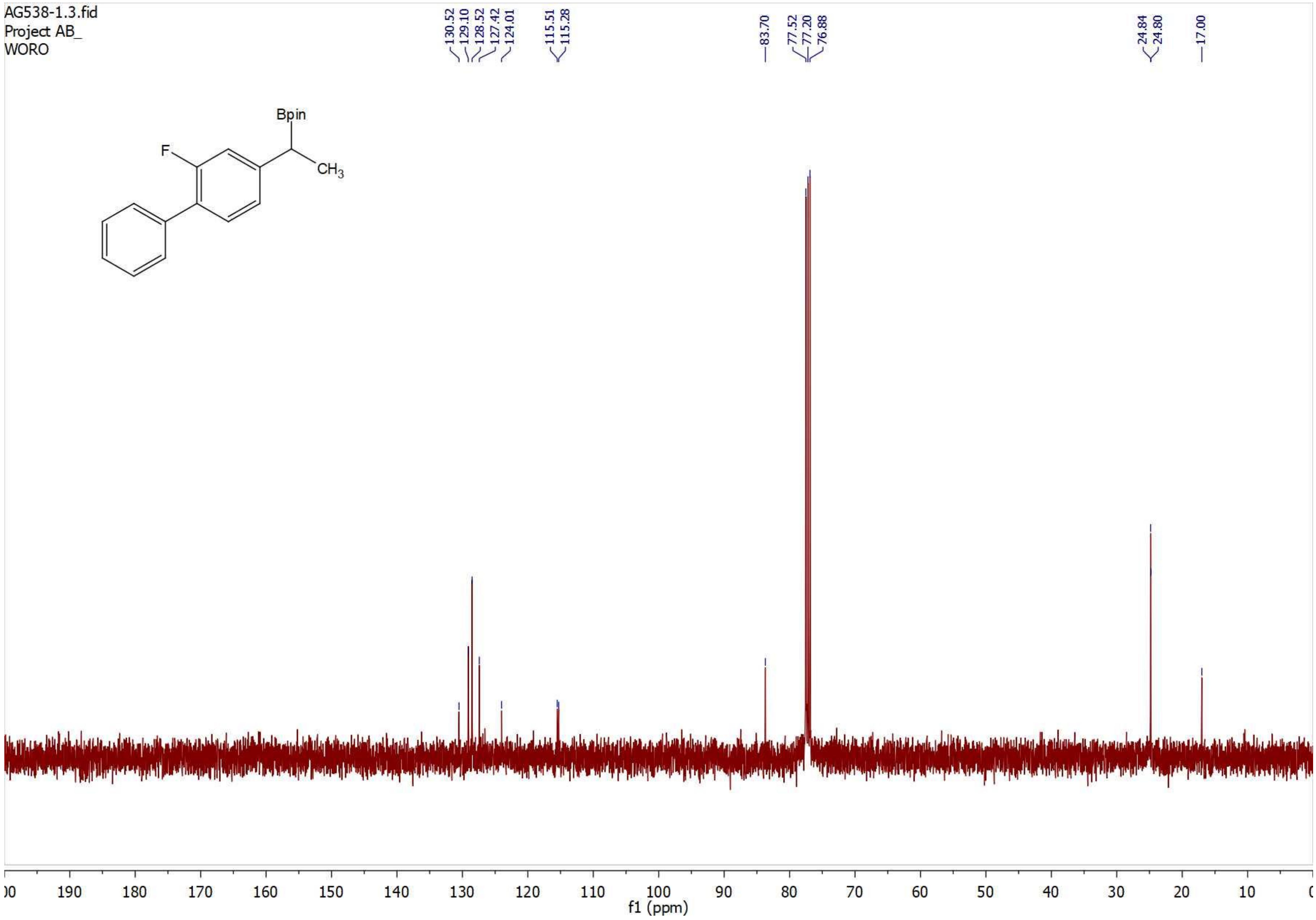

Compound 2a

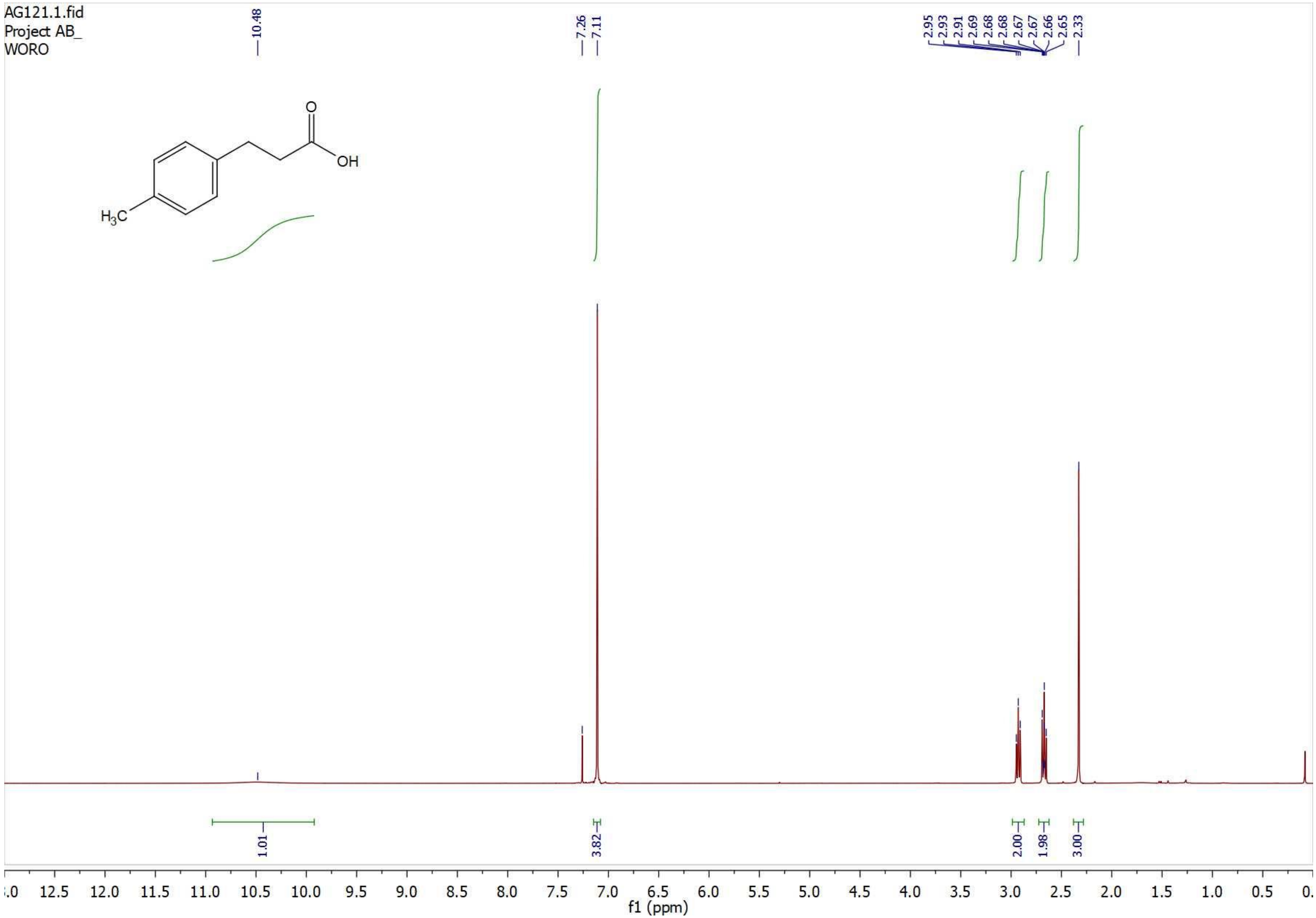

AG121.1.fid
Project AB_
WORO
10.48
7.26
7.11
2.95
2.93
2.91
2.69
2.68
2.68
2.67
2.67
2.66
2.65
2.33
O
OH
H_3C
1.01
3.82
2.00
1.98
3.00
f1 (ppm)

Compound 2a

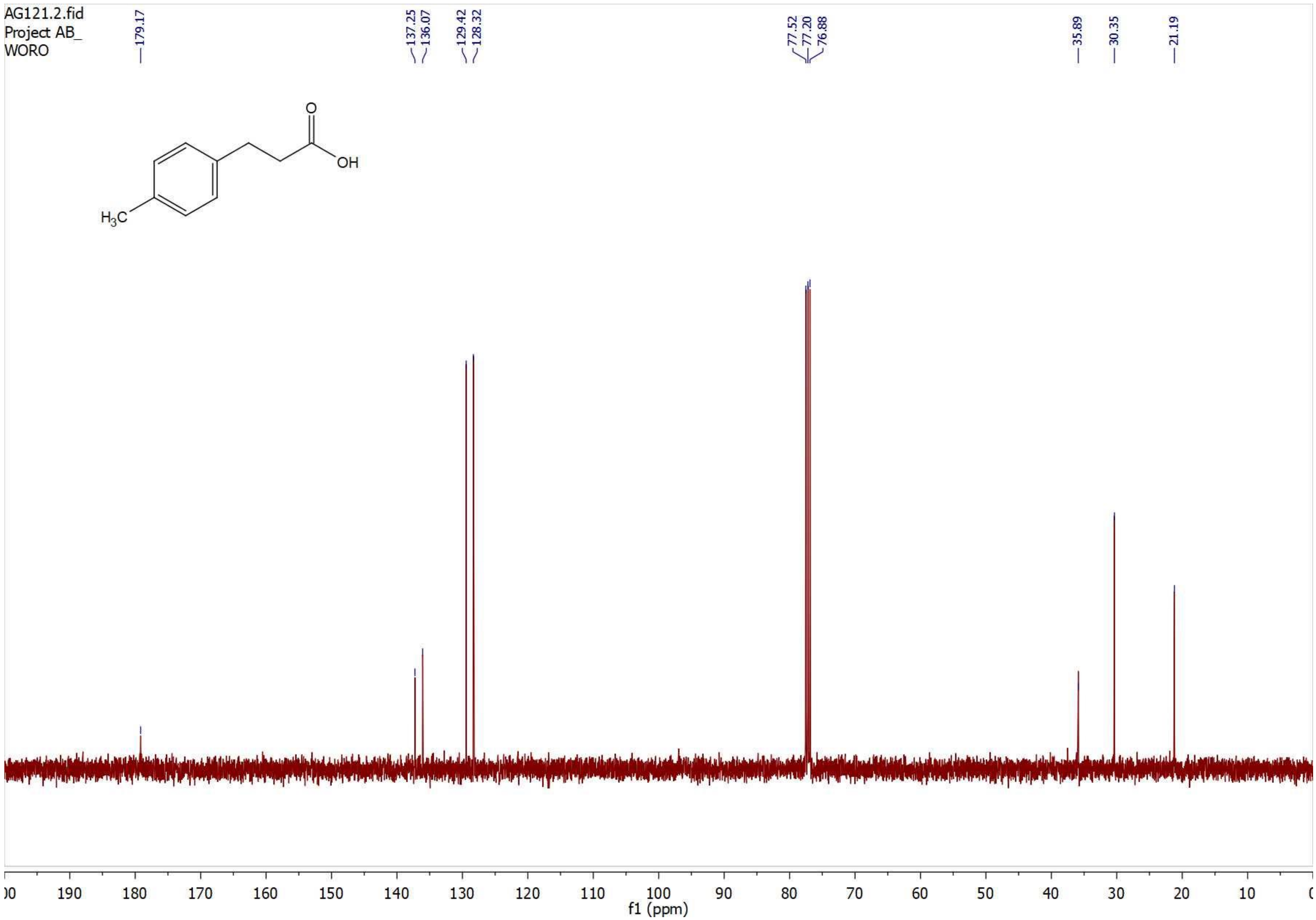
AG121.2.fid
Project AB_
WORO
179.17
137.25
136.07
129.42
128.32
77.52
77.20
76.88
35.89
30.35
21.19
O
OH
H_3C
190
180
170
160
150
140
130
120
110
100
90
80
70
60
50
40
30
20
10
f1 (ppm)

Compound 2b

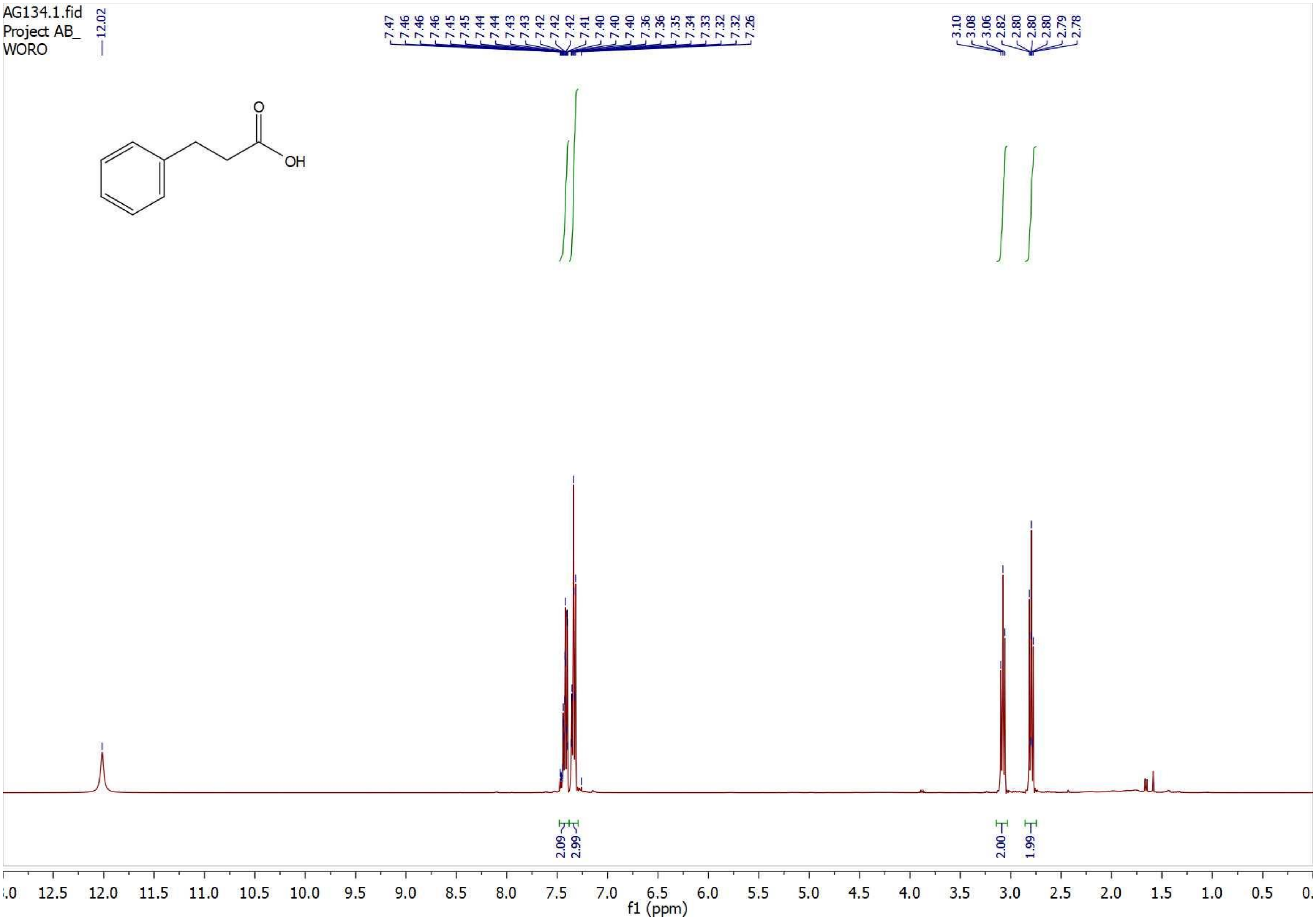
AG134.1.fid
Project AB_
WORO
12.02
7.47
7.46
7.46
7.45
7.45
7.44
7.44
7.43
7.43
7.42
7.42
7.42
7.41
7.40
7.40
7.40
7.36
7.36
7.35
7.34
7.33
7.32
7.32
7.26
3.10
3.08
3.06
2.82
2.80
2.80
2.80
2.79
2.78
OH
O
2.09
2.99
2.00
1.99
f1 (ppm)

Compound 2b

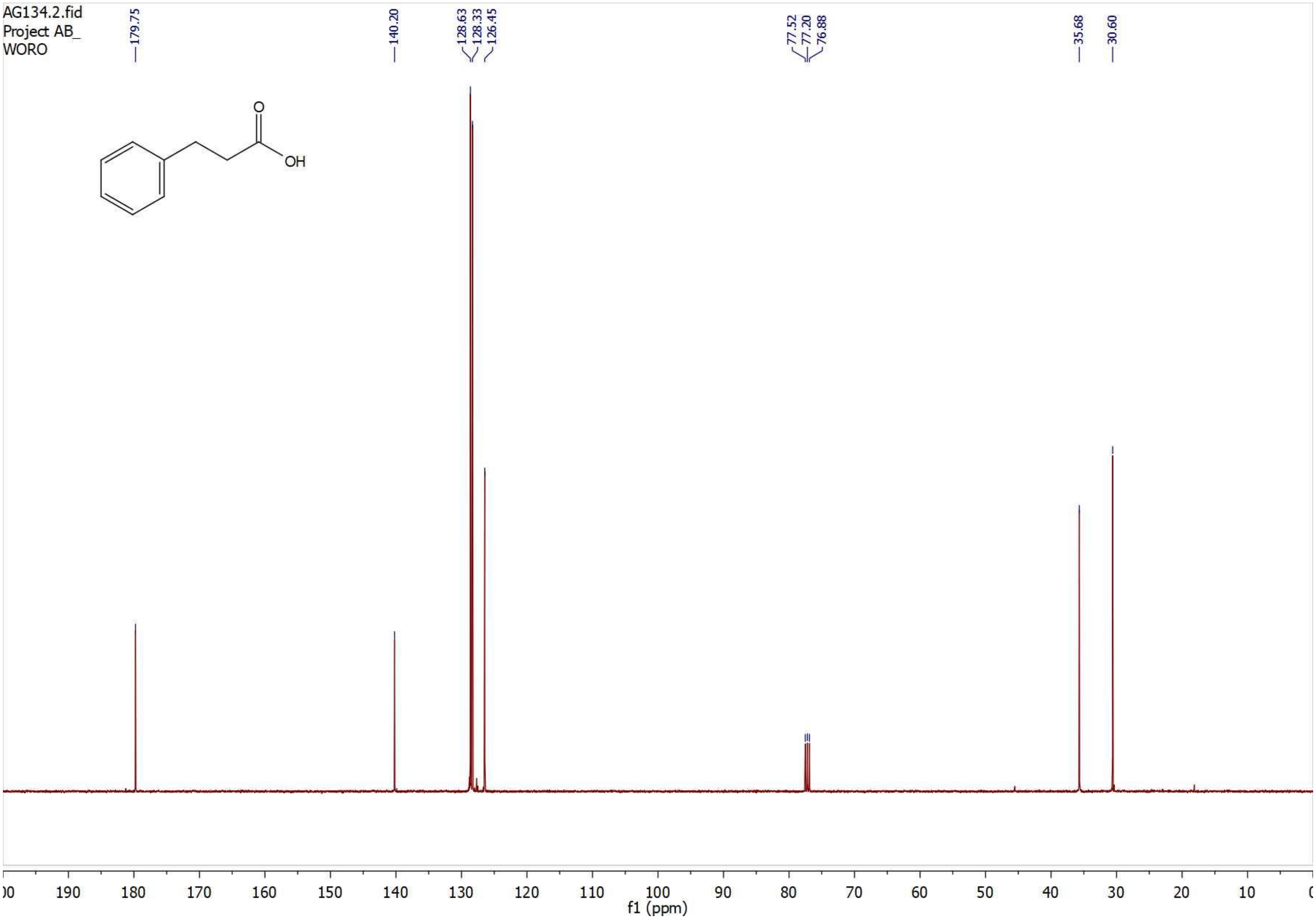
AG134.2.fid
Project AB_
WORO
179.75
140.20
128.63
128.33
126.45
77.52
77.20
76.88
35.68
30.60
O
OH
190
180
170
160
150
140
130
120
110
100
90
80
70
60
50
40
30
20
10
f1 (ppm)

Compound 2c

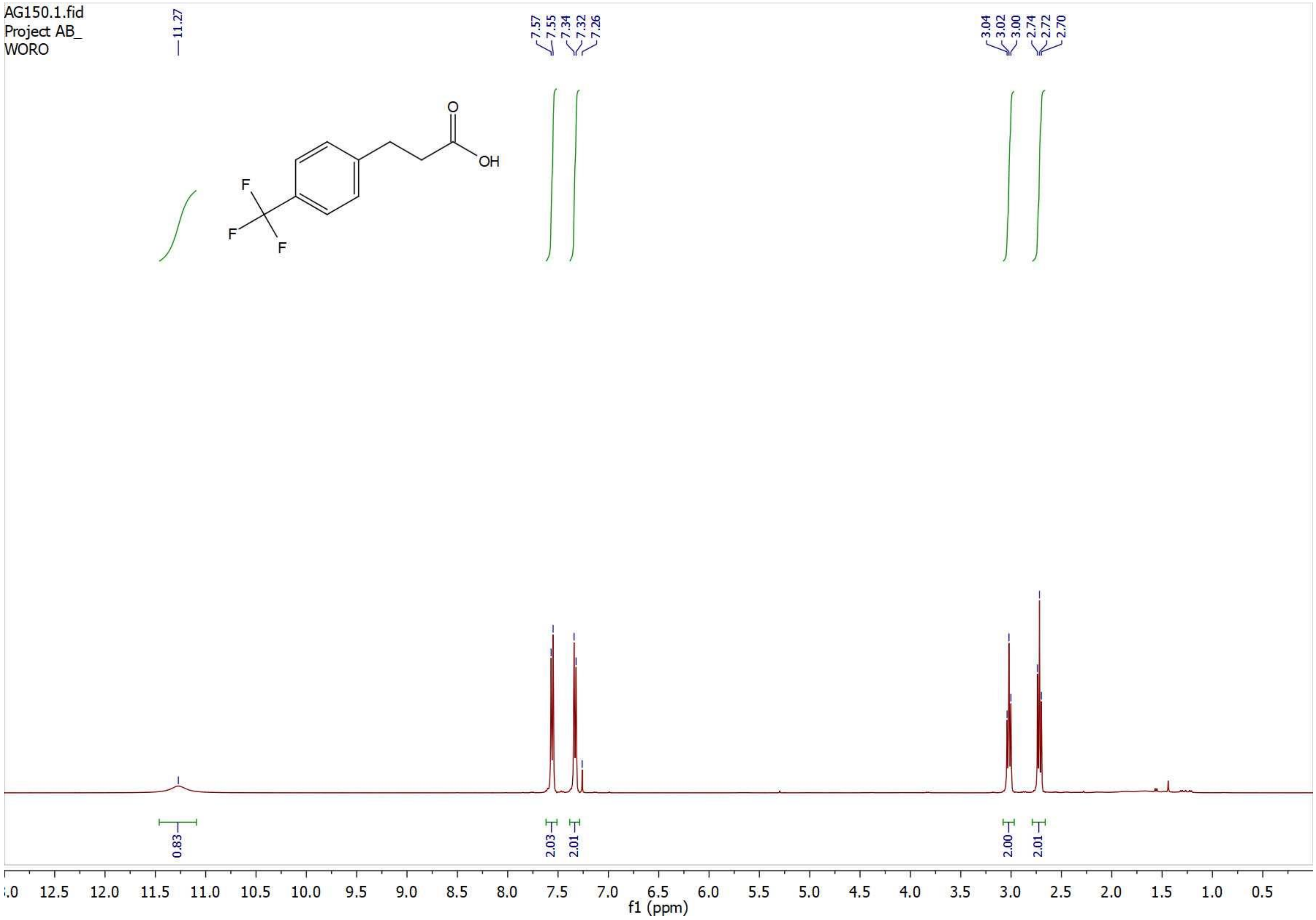
AG150.1.fid
Project AB_
WORO
11.27
7.57
7.55
7.34
7.32
7.26
3.04
3.02
3.00
2.74
2.72
2.70
F
F
F
O
OH
0.83
2.03
2.01
2.00
2.01
12.5
12.0
11.5
11.0
10.5
10.0
9.5
9.0
8.5
8.0
7.5
7.0
6.5
6.0
5.5
5.0
4.5
4.0
3.5
3.0
2.5
2.0
1.5
1.0
0.5
f1 (ppm)

Compound 2c

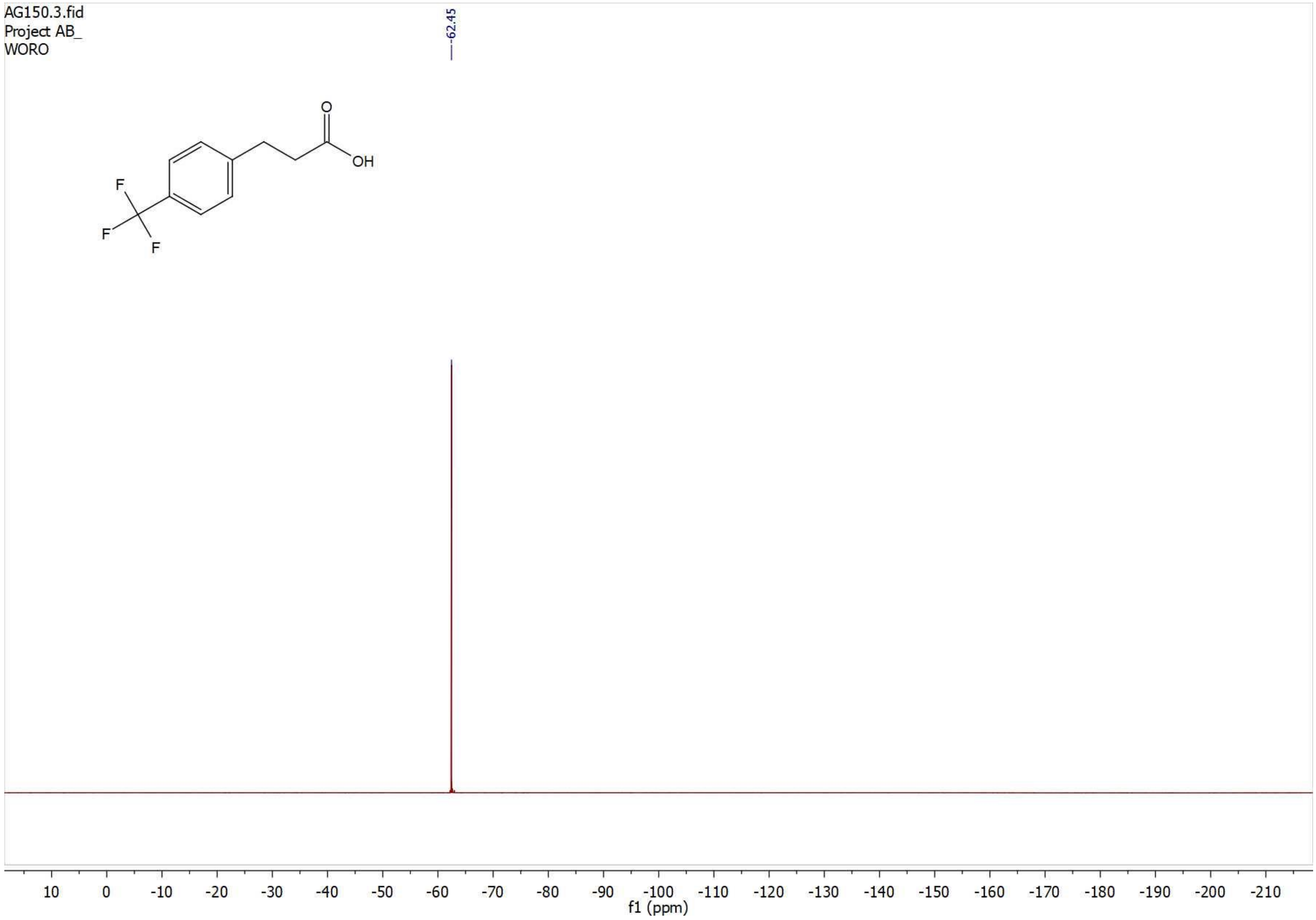
AG150.3.fid
Project AB_
WORO
-62.45
O
OH
F
F
F
10 0 -10 -20 -30 -40 -50 -60 -70 -80 -90 -100 -110 -120 -130 -140 -150 -160 -170 -180 -190 -200 -210
f1 (ppm)

Compound 2c

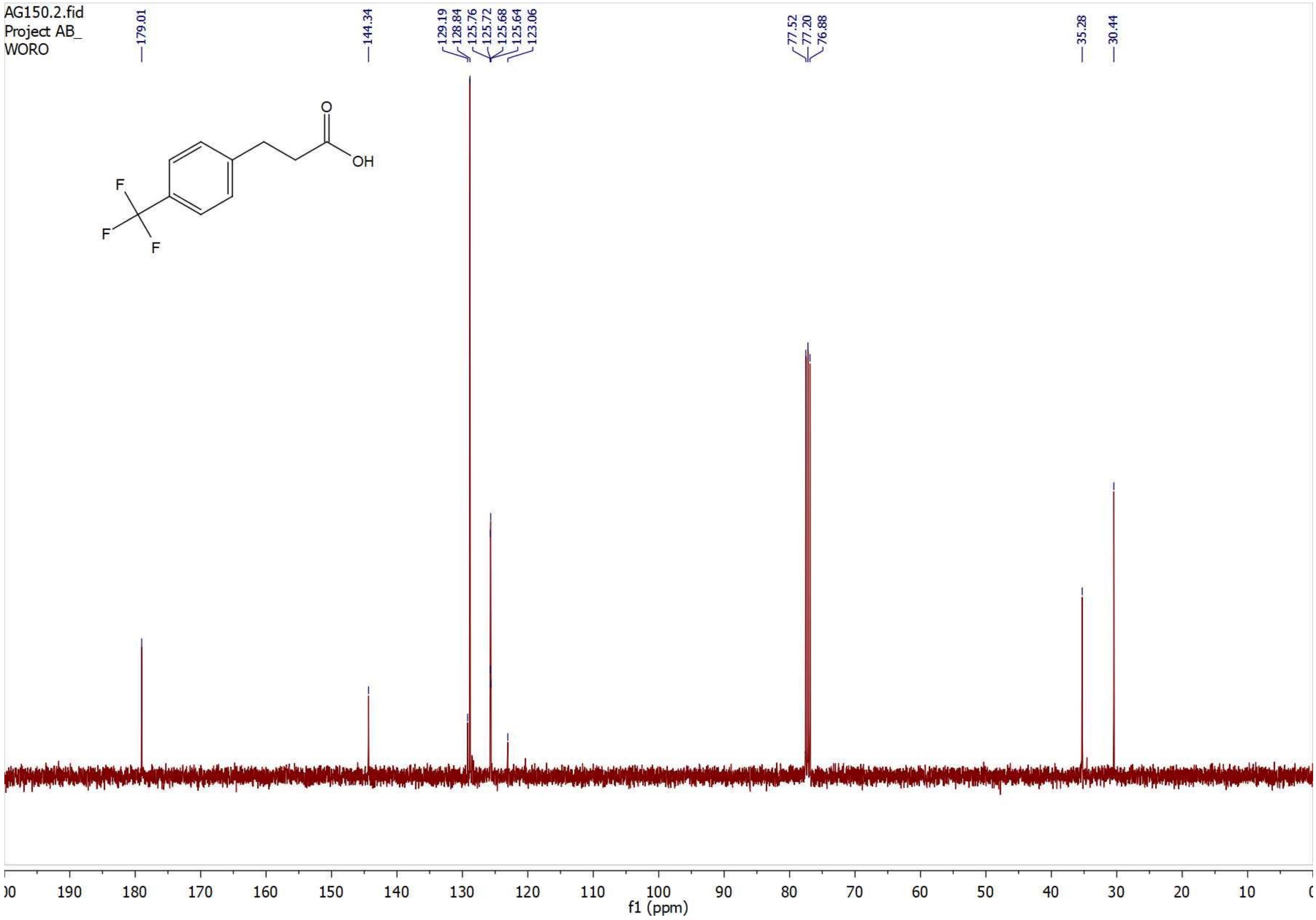
AG150.2.fid
Project AB_
WORO
179.01
144.34
129.19
128.84
125.76
125.72
125.68
125.64
123.06
77.52
77.20
76.88
35.28
30.44
O
OH
F
F
F
190
180
170
160
150
140
130
120
110
100
90
80
70
60
50
40
30
20
10
f1 (ppm)

Compound 2d

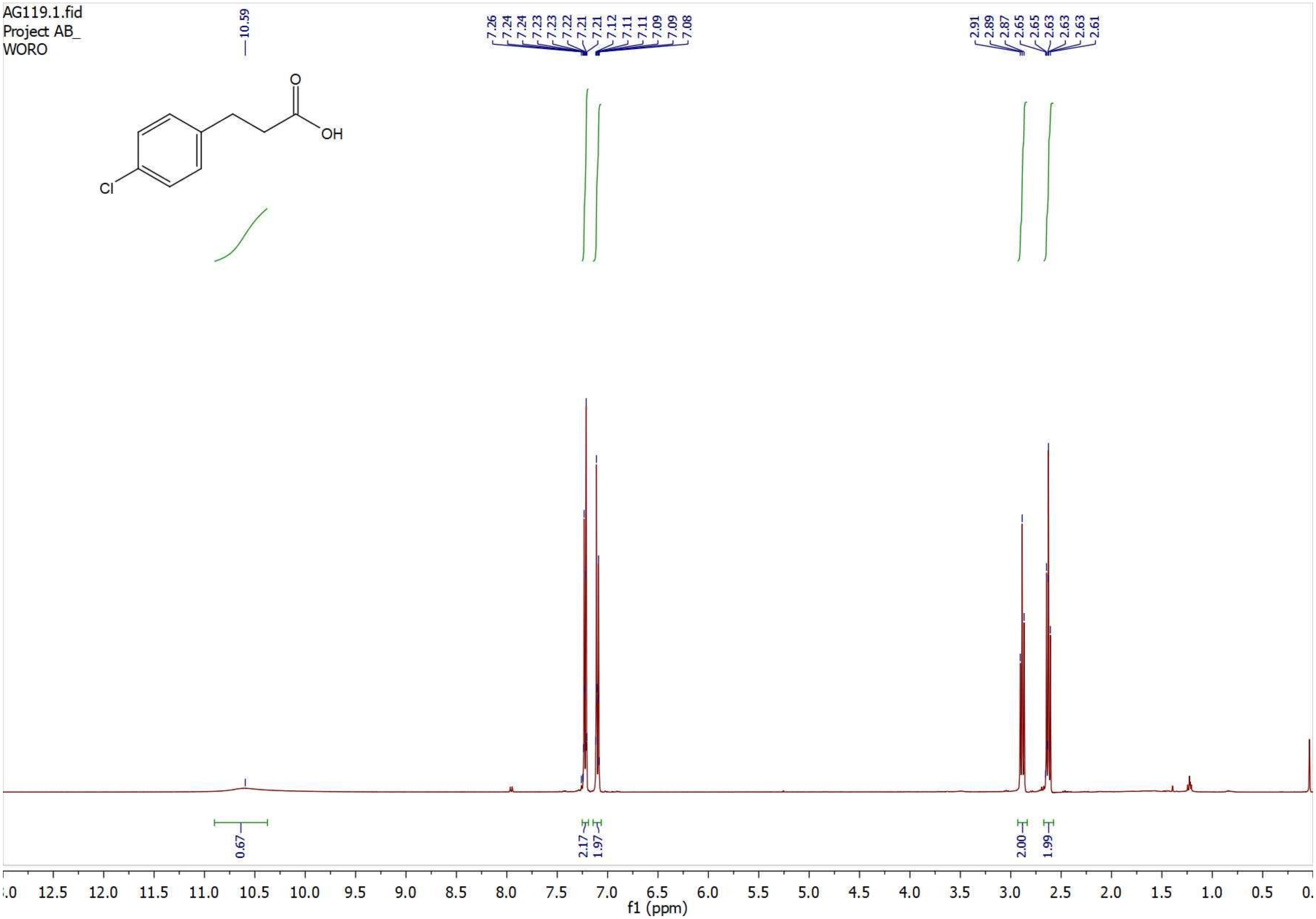
AG119.1.fid
Project AB_
WORO
10.59
7.26
7.24
7.24
7.23
7.23
7.22
7.21
7.21
7.12
7.11
7.11
7.09
7.09
7.08
2.91
2.89
2.87
2.65
2.65
2.63
2.63
2.63
2.61
O
OH
Cl
0.67
2.17
1.97
2.00
1.99
12.5
12.0
11.5
11.0
10.5
10.0
9.5
9.0
8.5
8.0
7.5
7.0
6.5
6.0
5.5
5.0
4.5
4.0
3.5
3.0
2.5
2.0
1.5
1.0
0.5
f1 (ppm)

Compound 2d

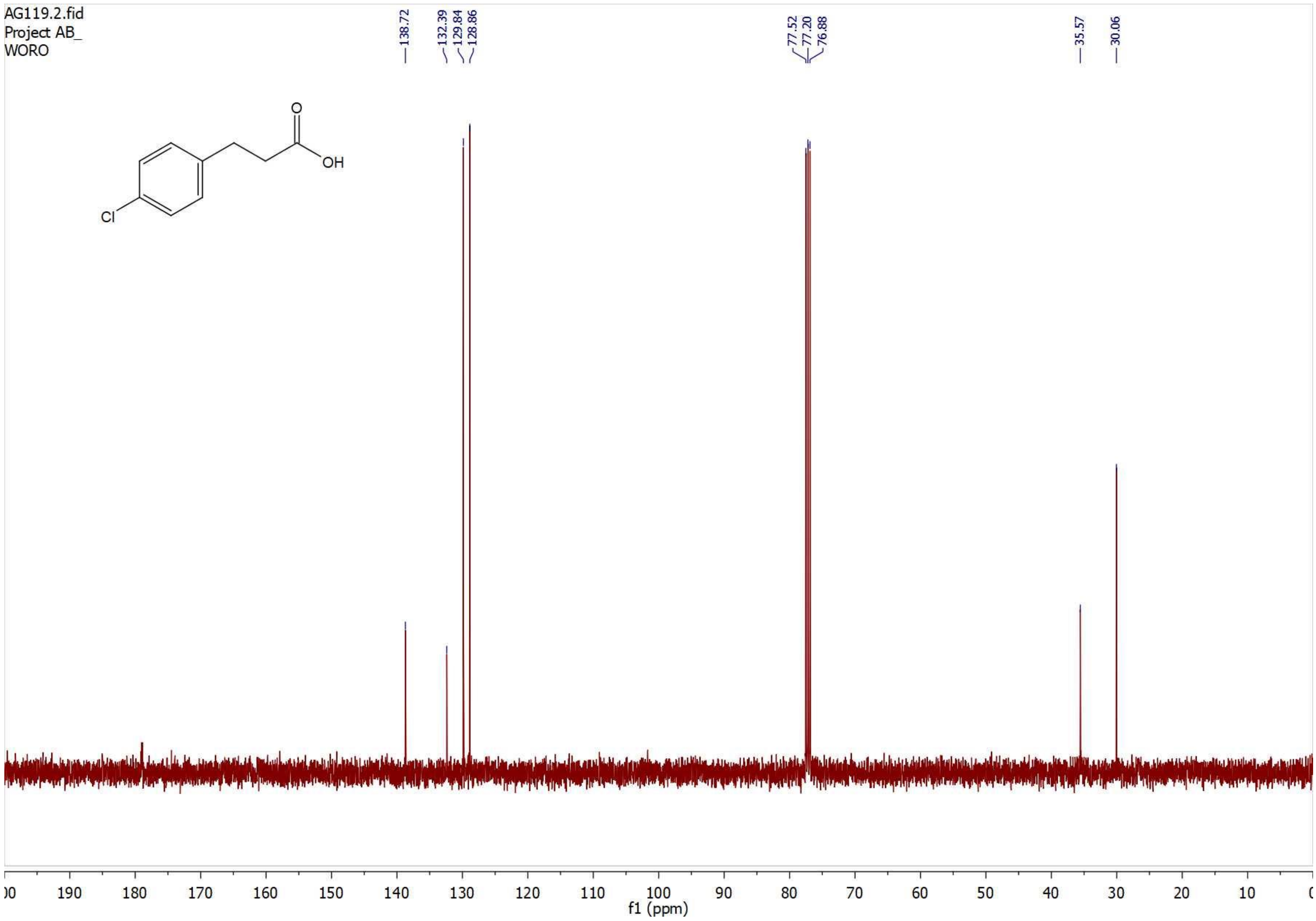
AG119.2.fid
Project AB_
WORO
138.72
132.39
129.84
128.86
77.52
77.20
76.88
35.57
30.06
O
OH
Cl
190
180
170
160
150
140
130
120
110
100
90
80
70
60
50
40
30
20
10
f1 (ppm)

Compound 2e

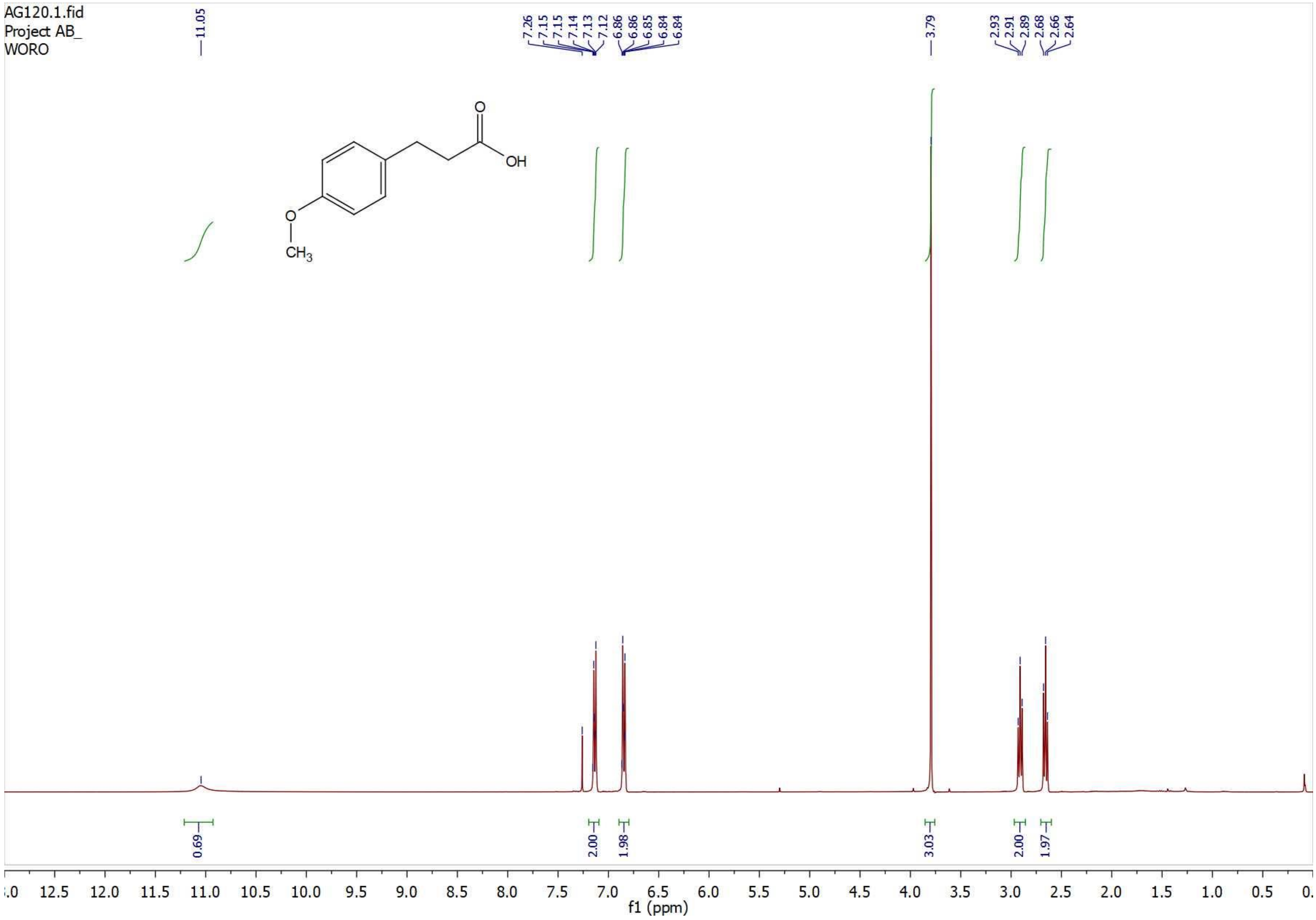
AG120.1.fid
Project AB_
WORO
11.05
7.26
7.15
7.15
7.14
7.13
7.12
6.86
6.86
6.85
6.84
6.84
3.79
2.93
2.91
2.89
2.68
2.66
2.64
O
OH
O
CH3
0.69
2.00
1.98
3.03
2.00
1.97
f1 (ppm)

Compound 2e

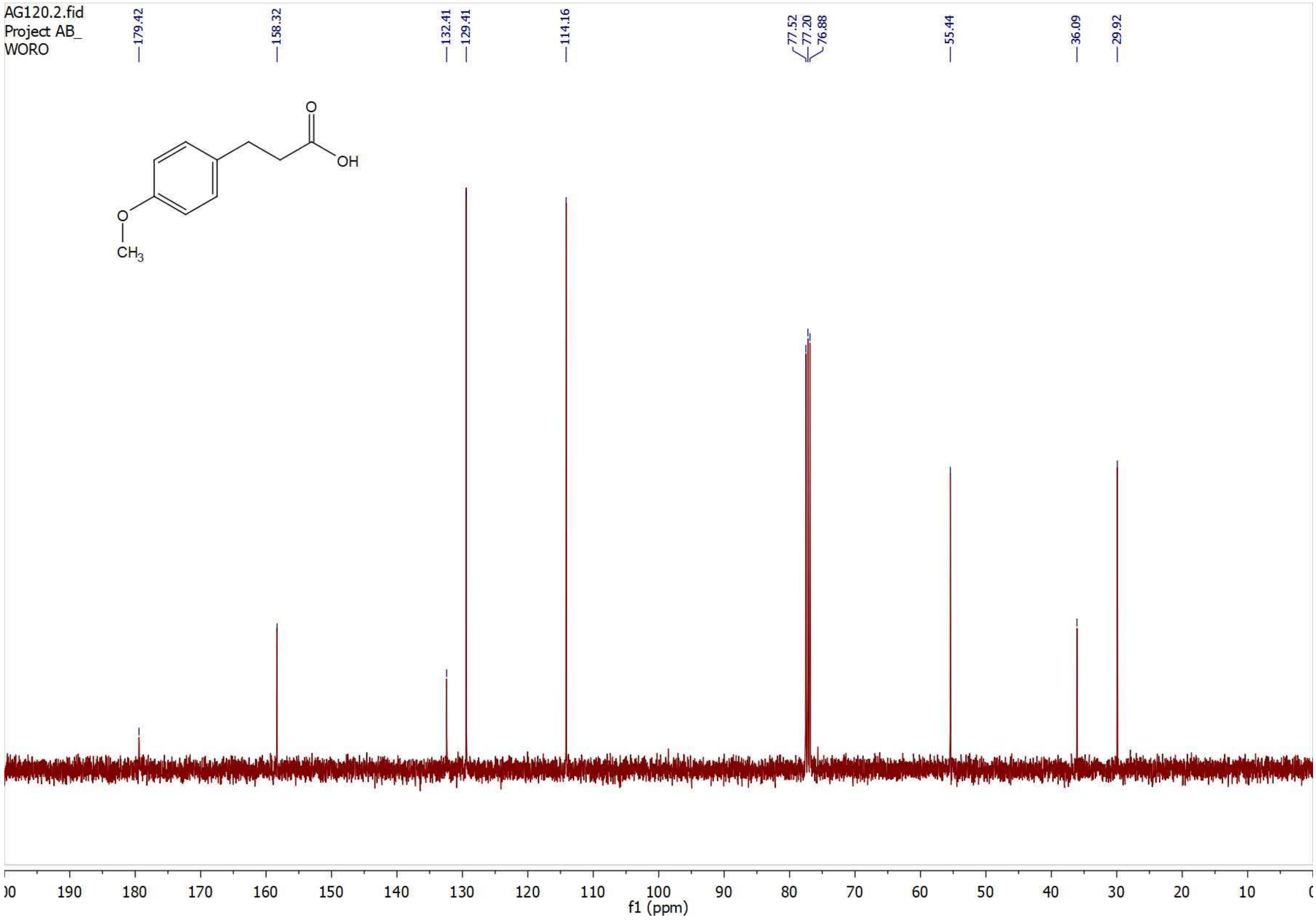
AG120.2.fid
Project AB_
WORO
179.42
158.32
132.41
129.41
114.16
77.52
77.20
76.88
55.44
36.09
29.92
O
OH
O
CH3
190
180
170
160
150
140
130
120
110
100
90
80
70
60
50
40
30
20
10
f1 (ppm)

Compound 2f

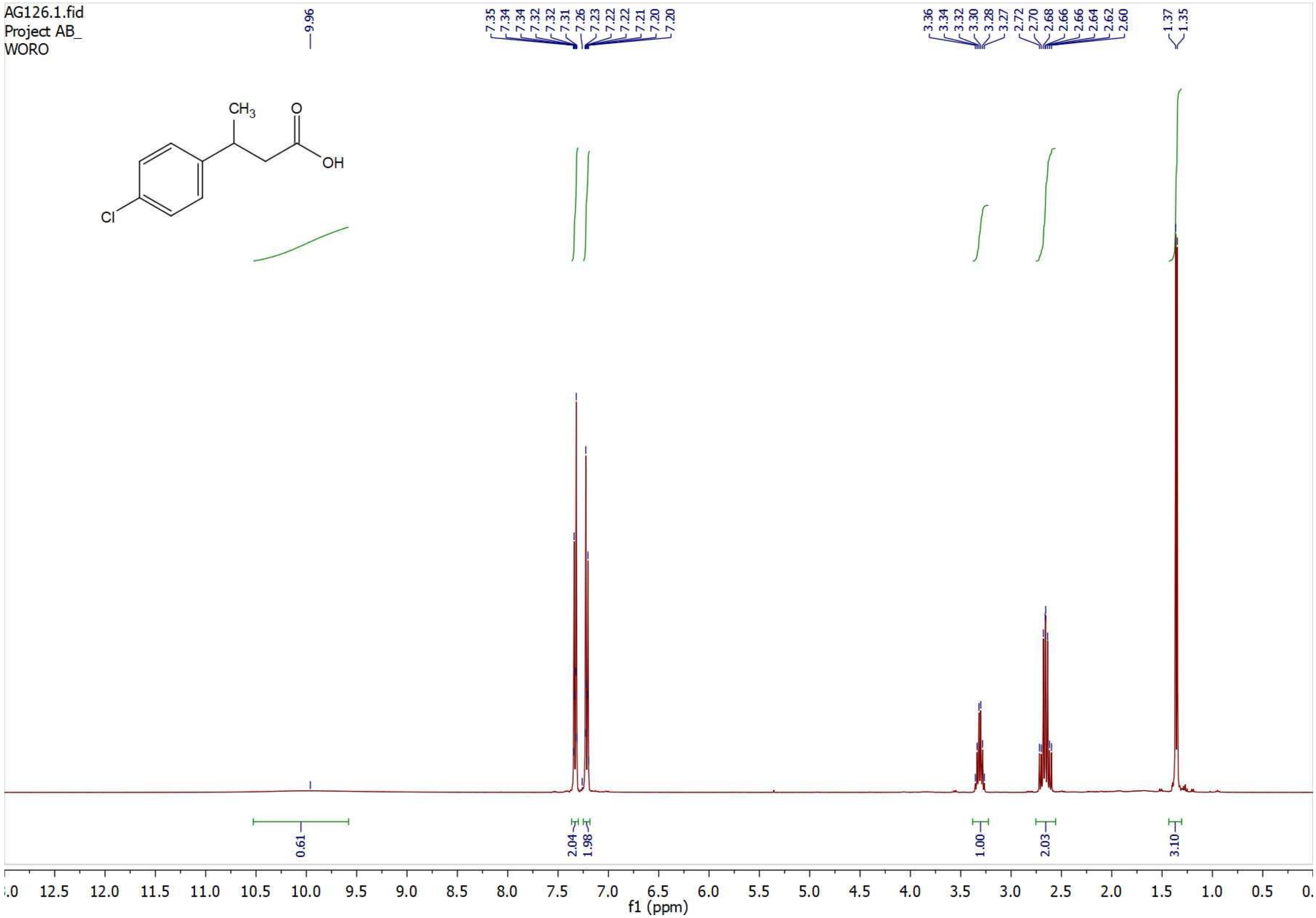
AG126.1.fid
Project AB_
WORO
CH3
O
OH
Cl
9.96
7.35
7.34
7.34
7.32
7.32
7.31
7.26
7.23
7.22
7.22
7.21
7.20
7.20
3.36
3.34
3.32
3.30
3.28
3.27
2.72
2.70
2.68
2.66
2.66
2.64
2.62
2.60
1.37
1.35
0.61
2.04
1.98
1.00
2.03
3.10
f1 (ppm)

Compound 2f

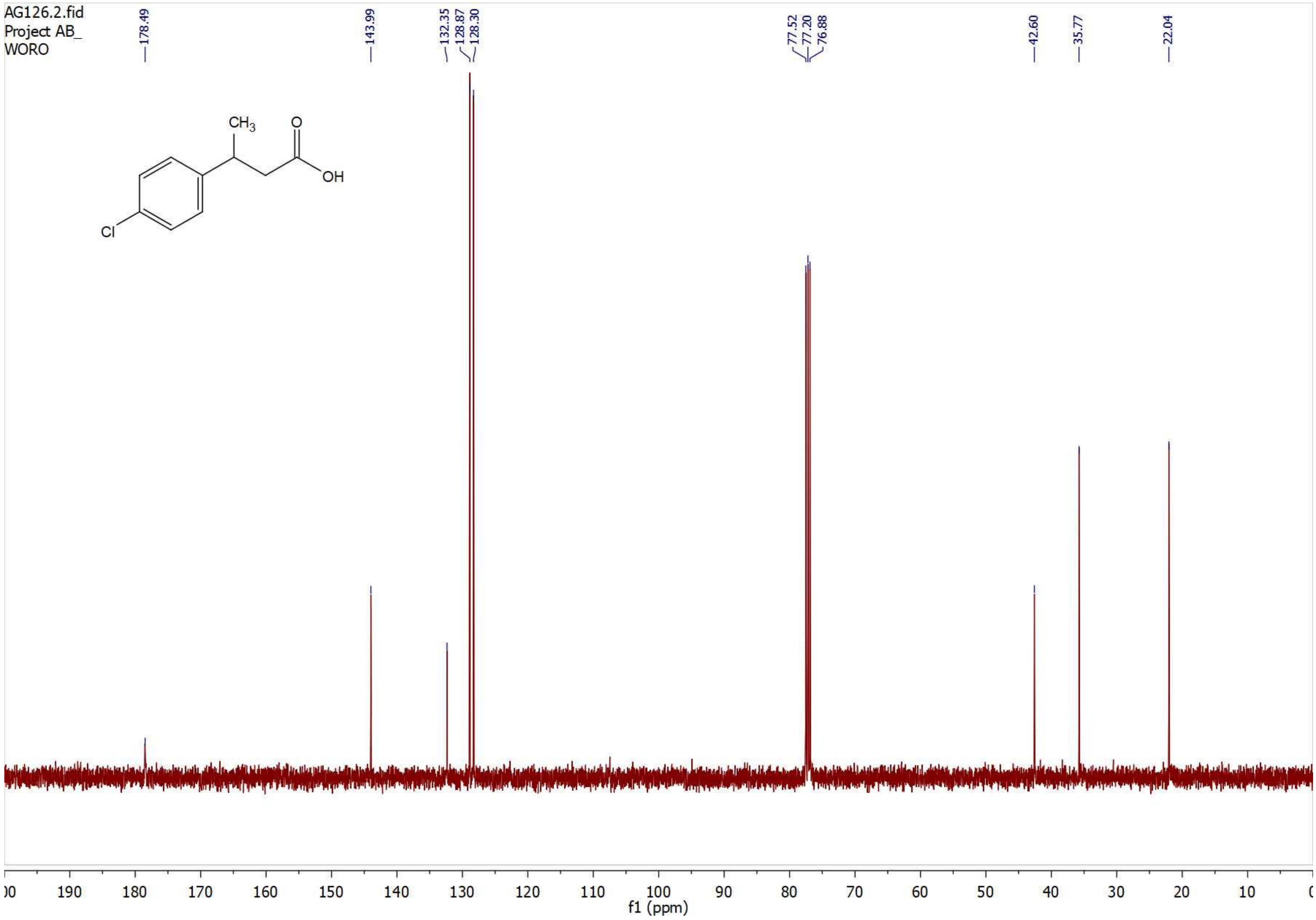
AG126.2.fid
Project AB_
WORO
178.49
143.99
132.35
128.87
128.30
77.52
77.20
76.88
42.60
35.77
22.04
CH3
O
OH
Cl
00
190
180
170
160
150
140
130
120
110
100
90
80
70
60
50
40
30
20
10
f1 (ppm)

Compound 2g

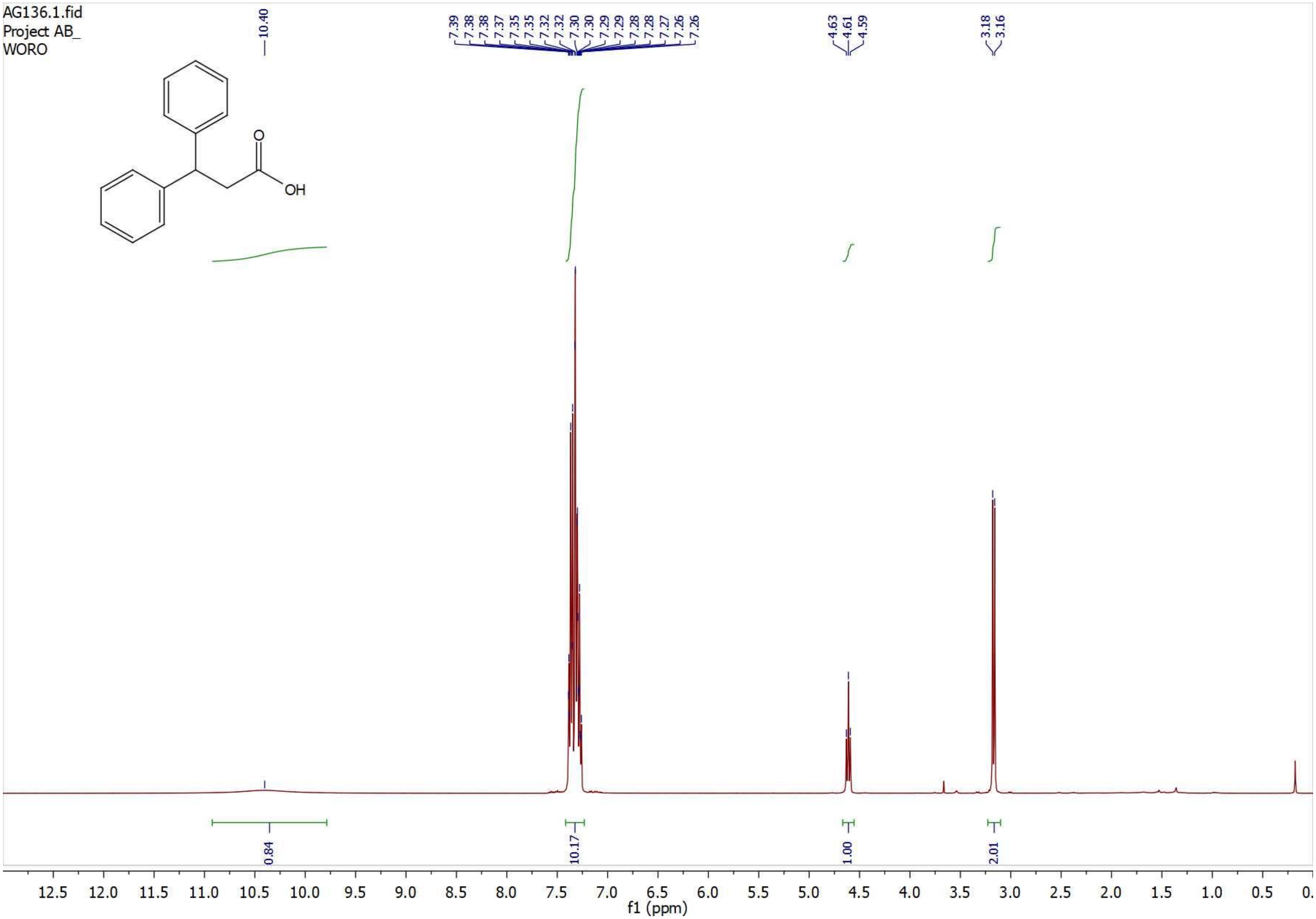

AG136.1.fid
Project AB_
WORO
OH
O
10.40
7.39
7.38
7.38
7.37
7.35
7.35
7.32
7.32
7.30
7.30
7.29
7.29
7.28
7.28
7.27
7.26
7.26
4.63
4.61
4.59
3.18
3.16
0.84
10.17
1.00
2.01
12.5 12.0 11.5 11.0 10.5 10.0 9.5 9.0 8.5 8.0 7.5 7.0 6.5 6.0 5.5 5.0 4.5 4.0 3.5 3.0 2.5 2.0 1.5 1.0 0.5 0.
f1 (ppm)

Compound 2g

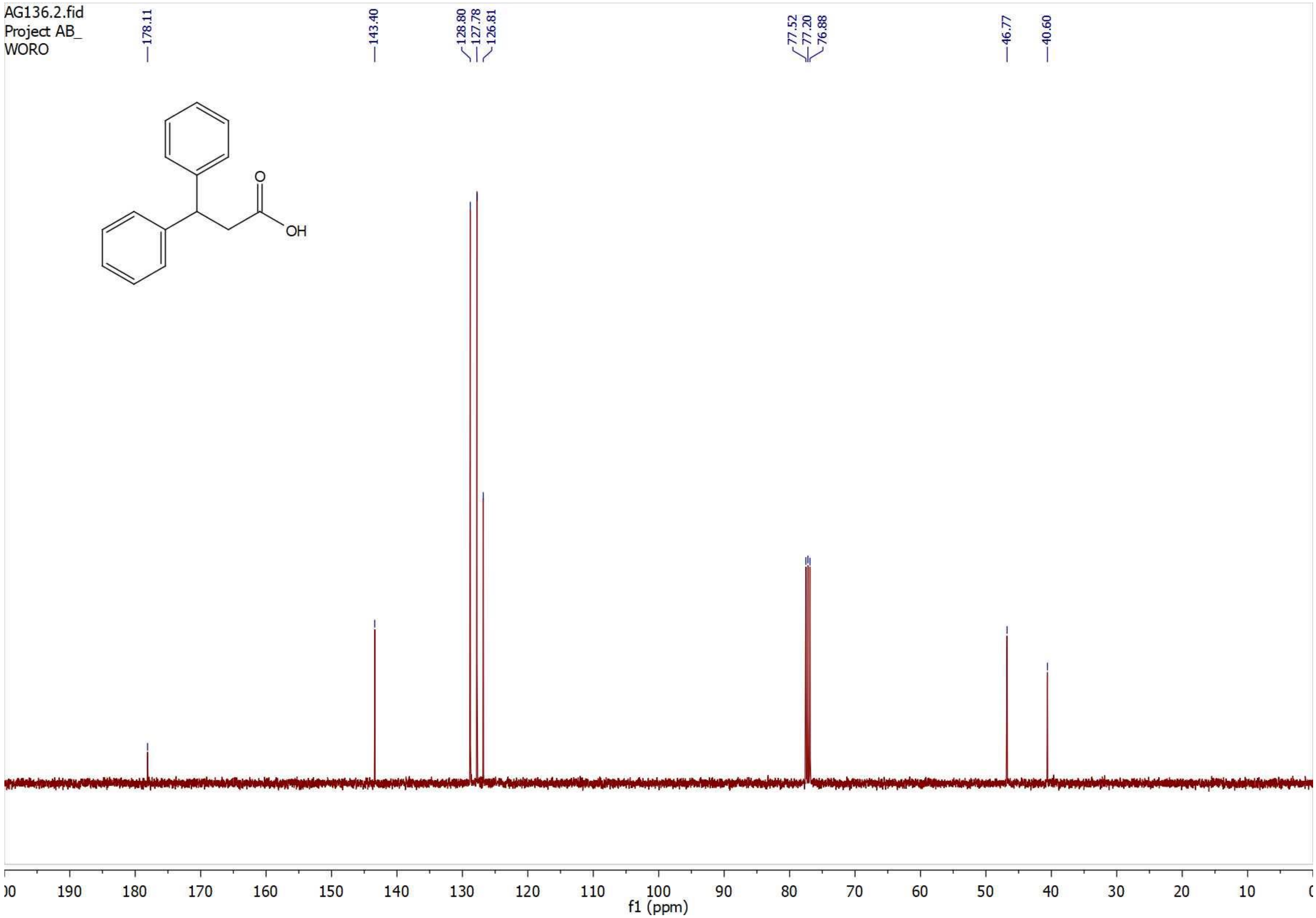
AG136.2.fid
Project AB_
WORO
178.11
143.40
128.80
127.78
126.81
77.52
77.20
76.88
46.77
40.60
O
OH
190
180
170
160
150
140
130
120
110
100
90
80
70
60
50
40
30
20
10
f1 (ppm)

Compound 2h

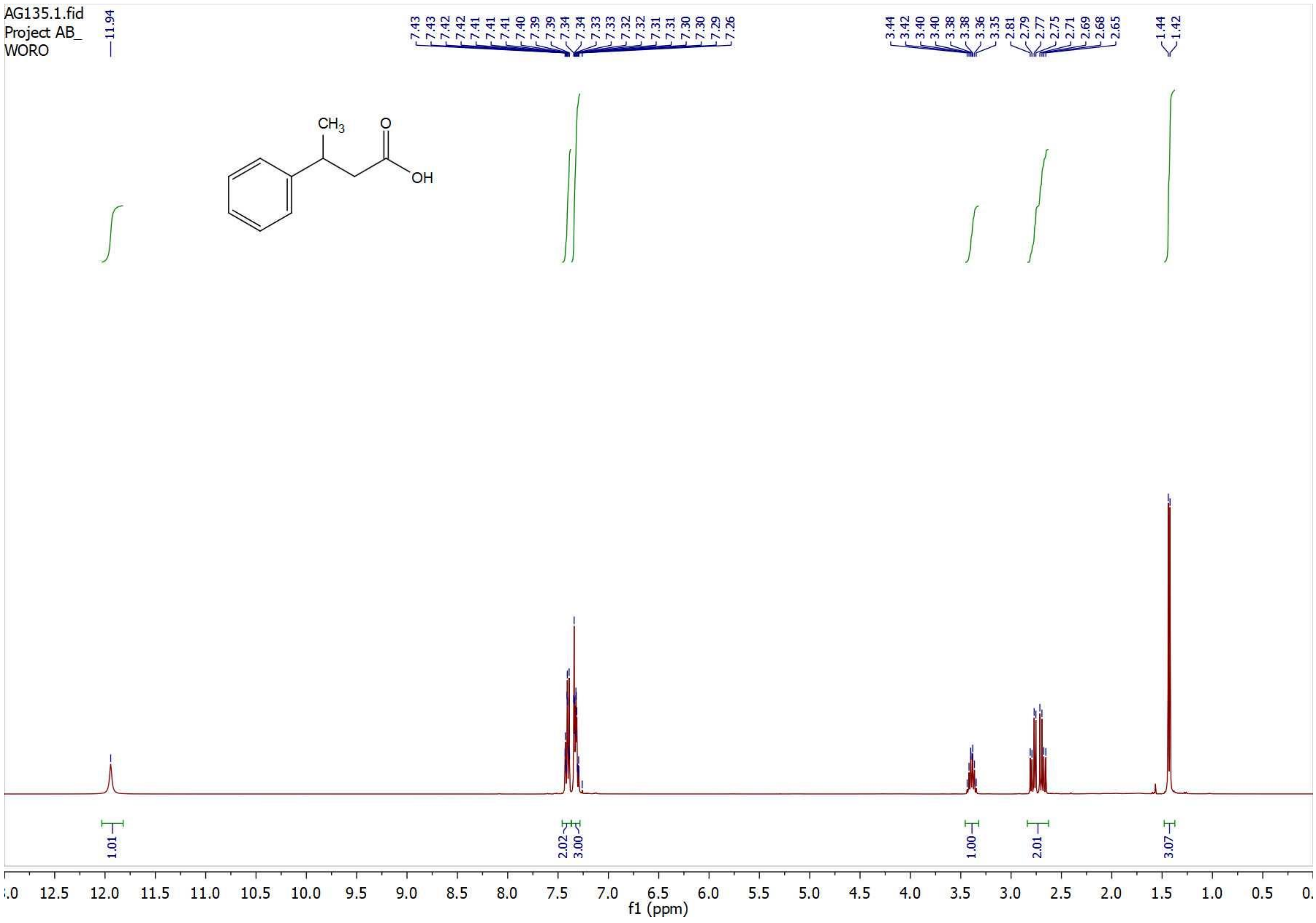
AG135.1.fid
Project AB_
WORO
11.94
7.43
7.43
7.42
7.42
7.41
7.41
7.41
7.40
7.39
7.39
7.34
7.34
7.33
7.32
7.32
7.31
7.31
7.30
7.30
7.29
7.26
3.44
3.42
3.40
3.40
3.38
3.38
3.36
3.35
2.81
2.79
2.77
2.75
2.71
2.69
2.68
2.65
1.44
1.42
CH3
O
OH
1.01
2.02
3.00
1.00
2.01
3.07
12.5
12.0
11.5
11.0
10.5
10.0
9.5
9.0
8.5
8.0
7.5
7.0
6.5
6.0
5.5
5.0
4.5
4.0
3.5
3.0
2.5
2.0
1.5
1.0
0.5
f1 (ppm)

Compound 2h

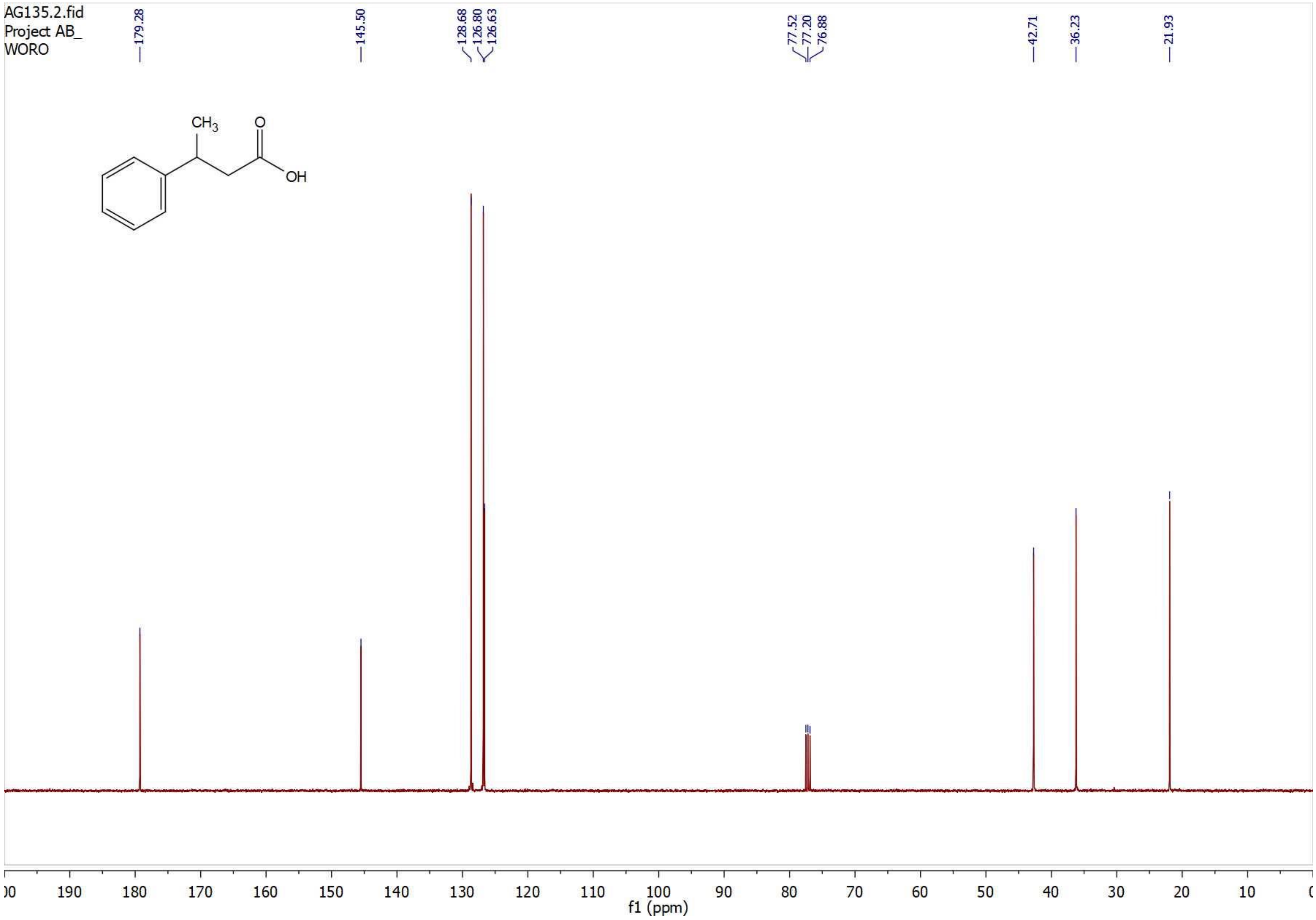
AG135.2.fid
Project AB_
WORO
179.28
145.50
128.68
126.80
126.63
77.52
77.20
76.88
42.71
36.23
21.93
CH3
O
OH
190
180
170
160
150
140
130
120
110
100
90
80
70
60
50
40
30
20
10
f1 (ppm)

Compound 2i

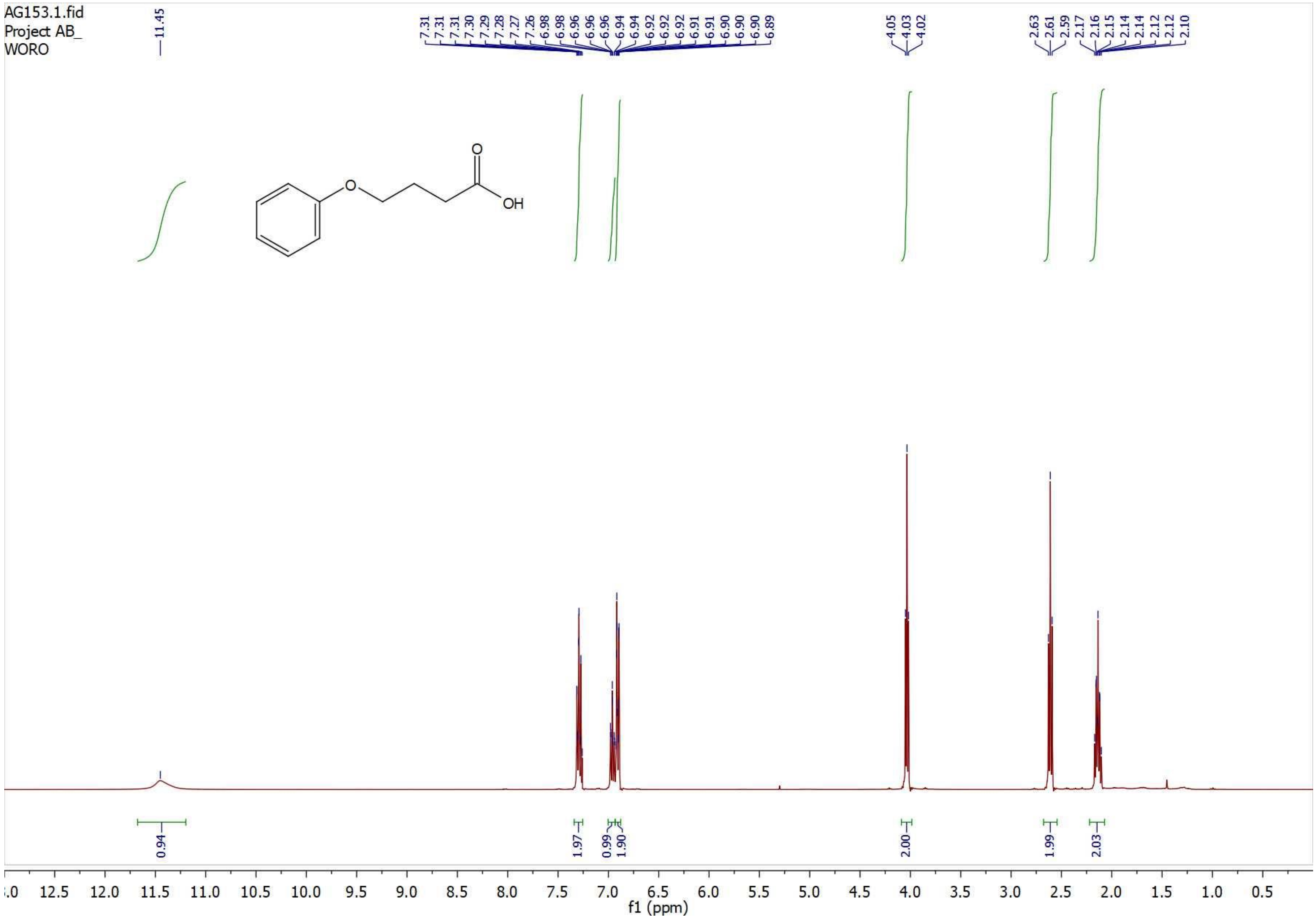

Compound 2i

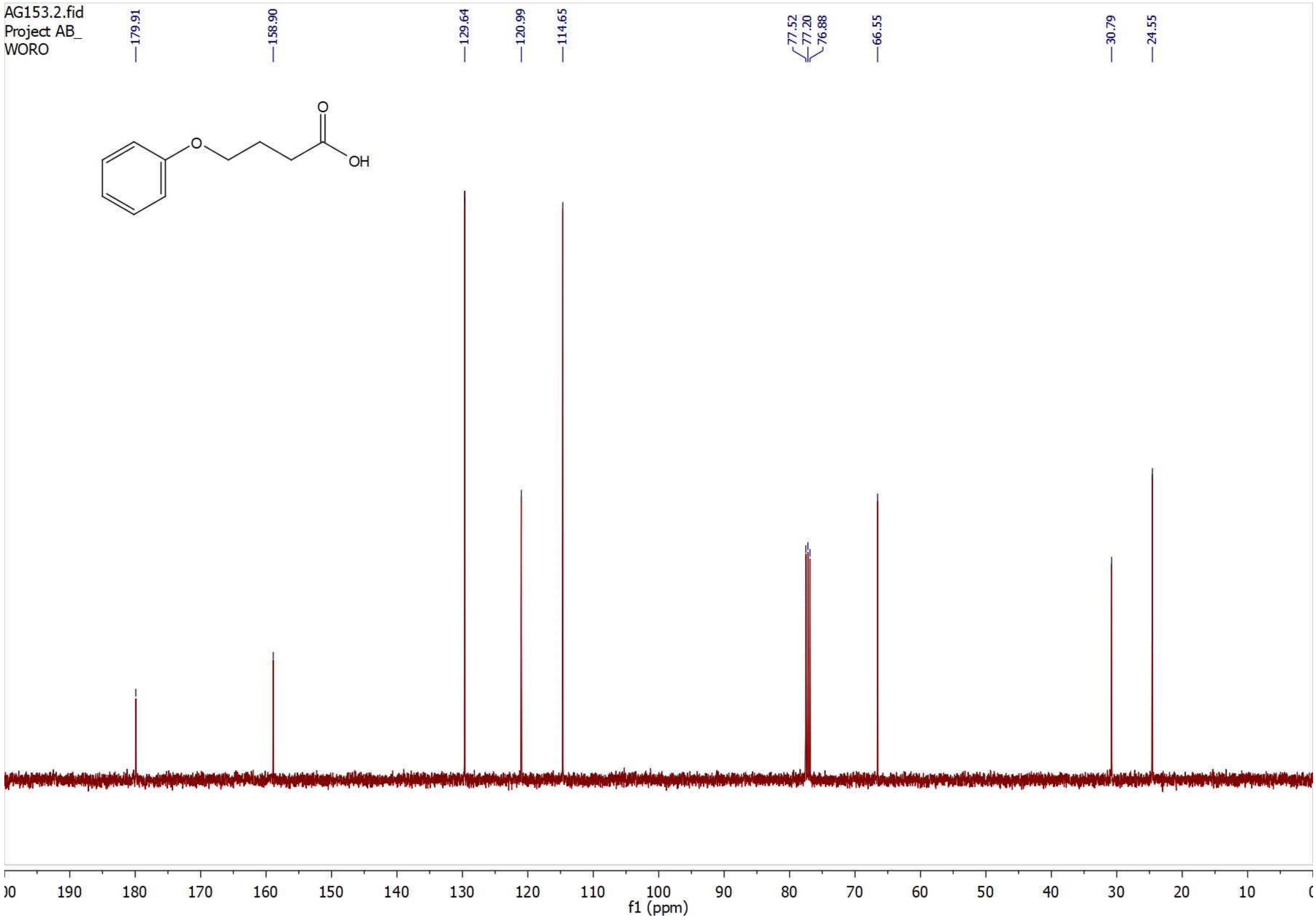
AG153.2.fid
Project AB_
WORO
179.91
158.90
129.64
120.99
114.65
77.52
77.20
76.88
66.55
30.79
24.55
O
OH
190
180
170
160
150
140
130
120
110
100
90
80
70
60
50
40
30
20
10
f1 (ppm)

Compound 2j

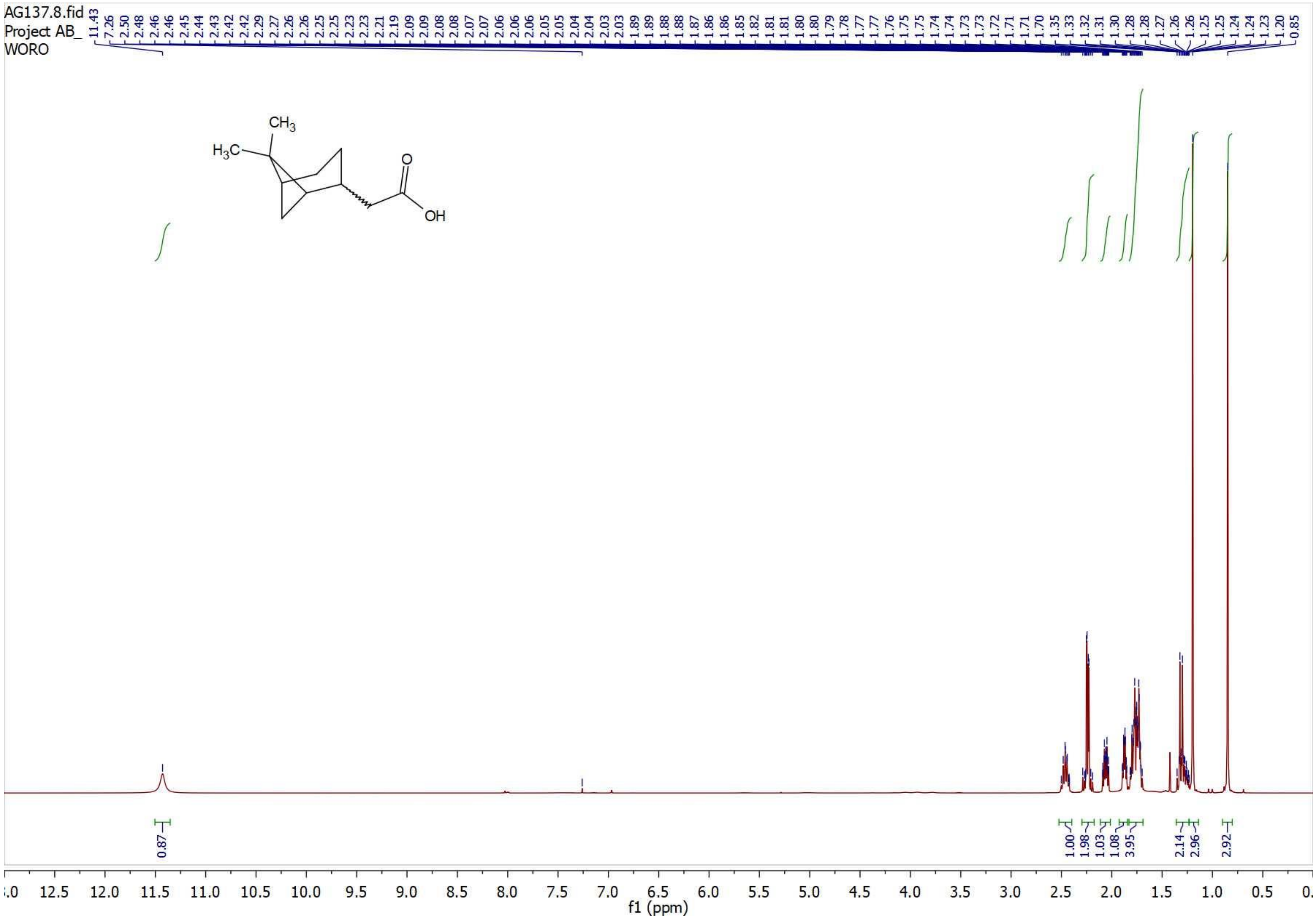

Compound 2j

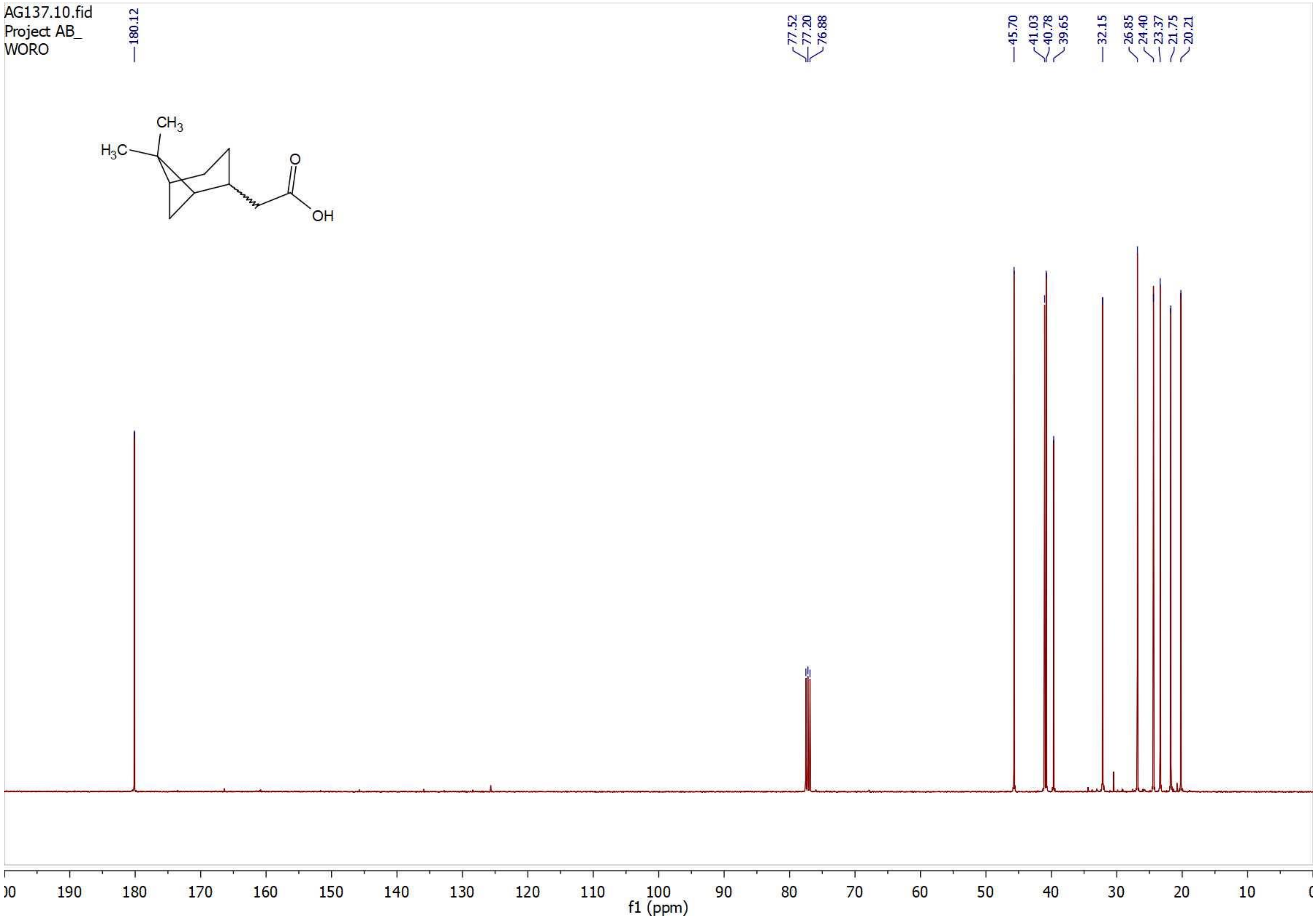
AG137.10.fid
Project AB_
WORO
180.12
77.52
77.20
76.88
45.70
41.03
40.78
39.65
32.15
26.85
24.40
23.37
21.75
20.21
CH3
H3C
O
OH
f1 (ppm)

Compound 2k

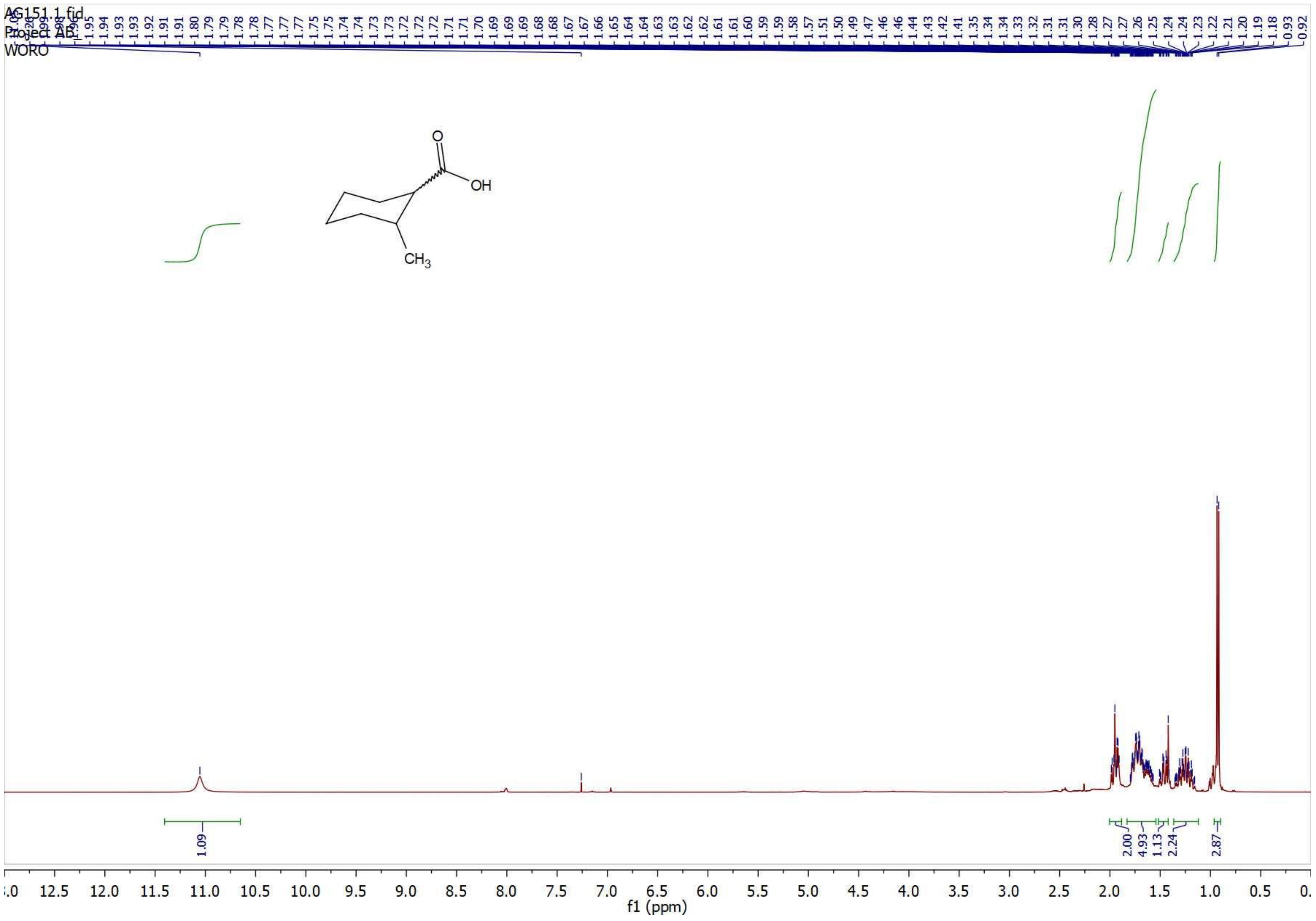

Compound 2k

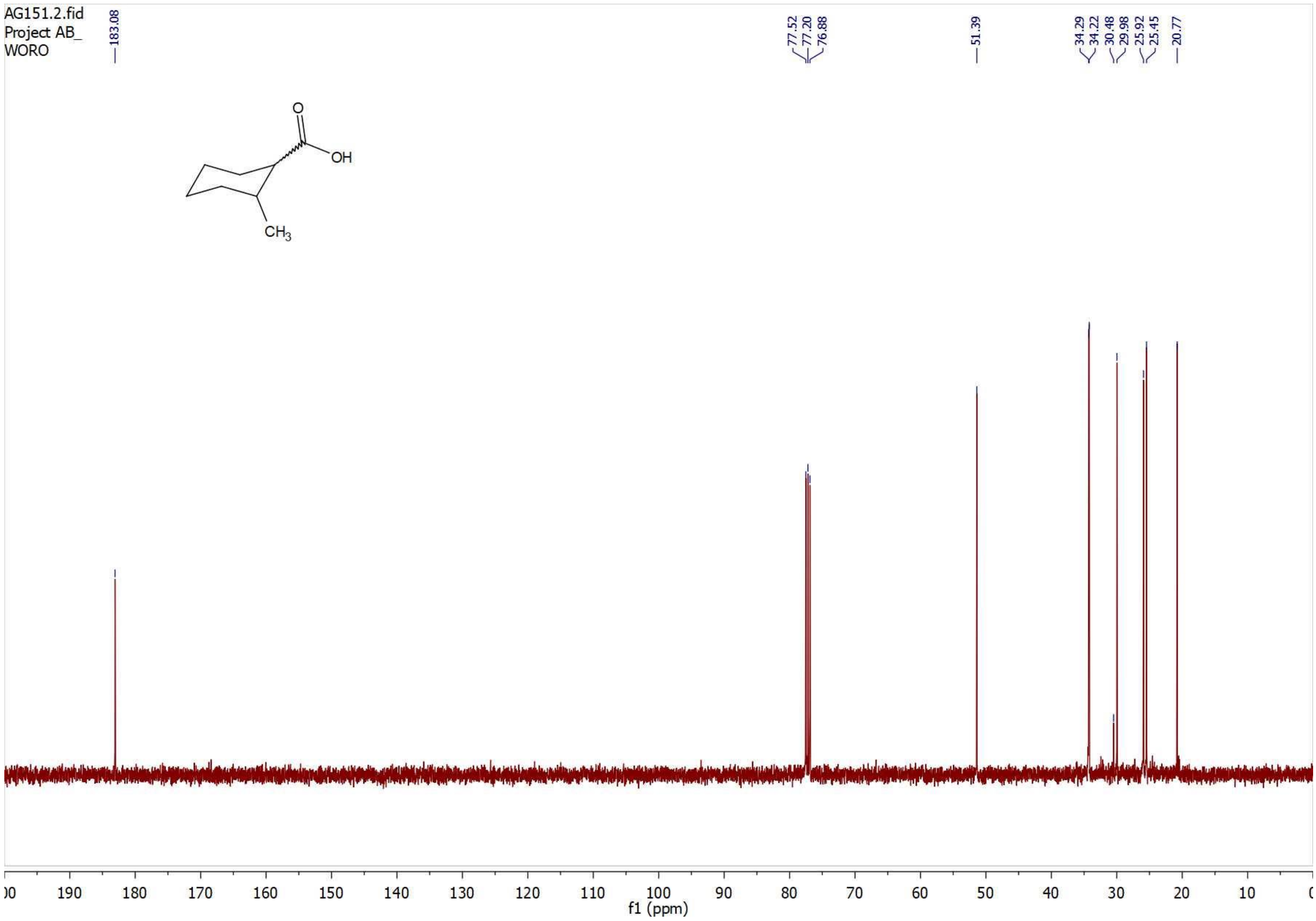
AG151.2.fid
Project AB_
WORO
183.08
77.52
77.20
76.88
51.39
34.29
34.22
30.48
29.98
25.92
25.45
20.77
O
OH
CH3
00
190
180
170
160
150
140
130
120
110
100
90
80
70
60
50
40
30
20
10
f1 (ppm)

Compound 2l

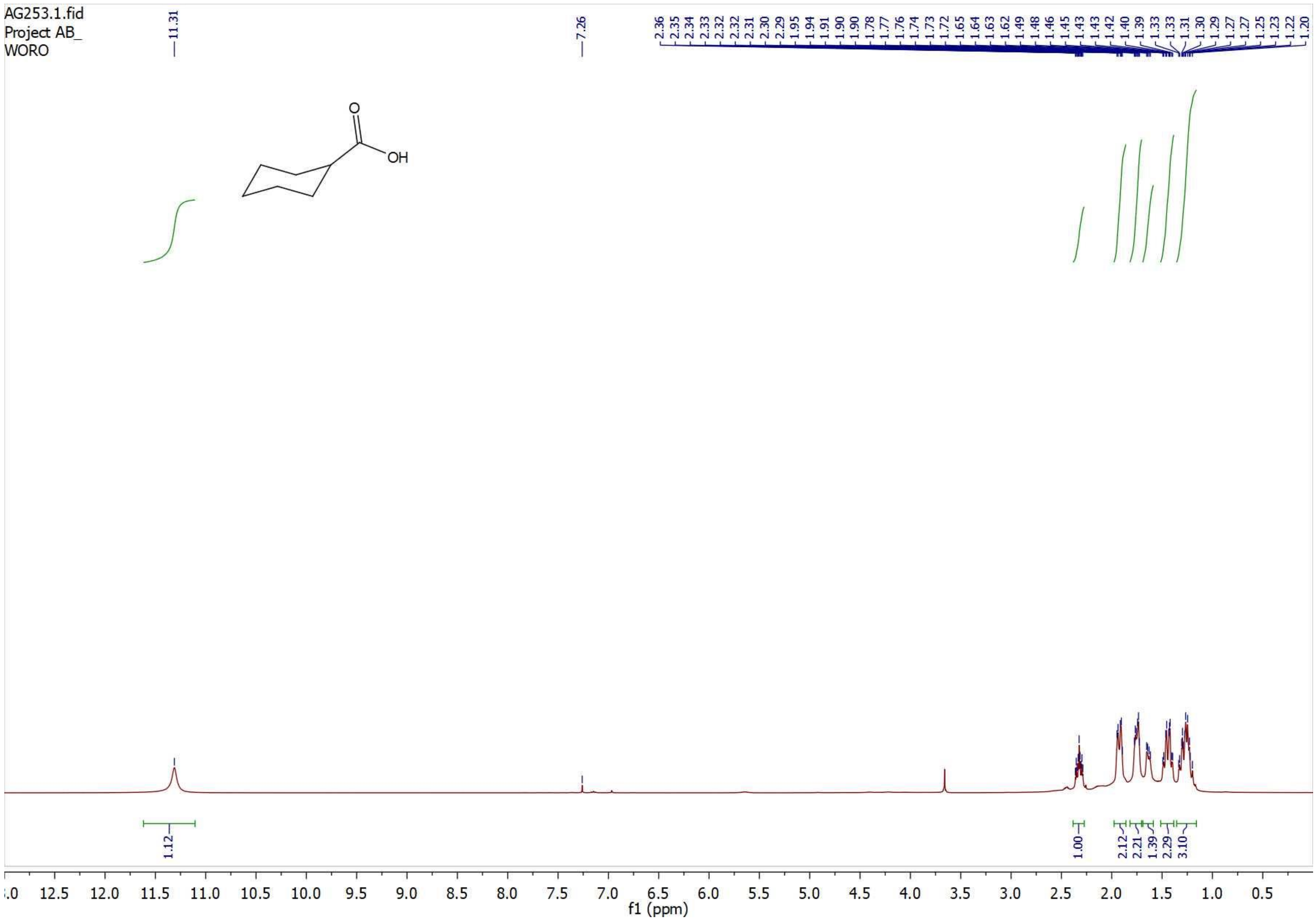

AG253.1.fid
Project AB_
WORO
11.31
7.26
2.36 2.35 2.34 2.33 2.32 2.32 2.31 2.30 2.29 1.95 1.94 1.91 1.90 1.90 1.78 1.77 1.76 1.74 1.73 1.72 1.65 1.64 1.63 1.62 1.49 1.48 1.46 1.45 1.43 1.43 1.42 1.40 1.39 1.33 1.33 1.31 1.30 1.29 1.27 1.27 1.25 1.23 1.22 1.20
OH
O
1.12
1.00
2.12
2.21
1.39
2.29
3.10
f1 (ppm)

Compound 2l

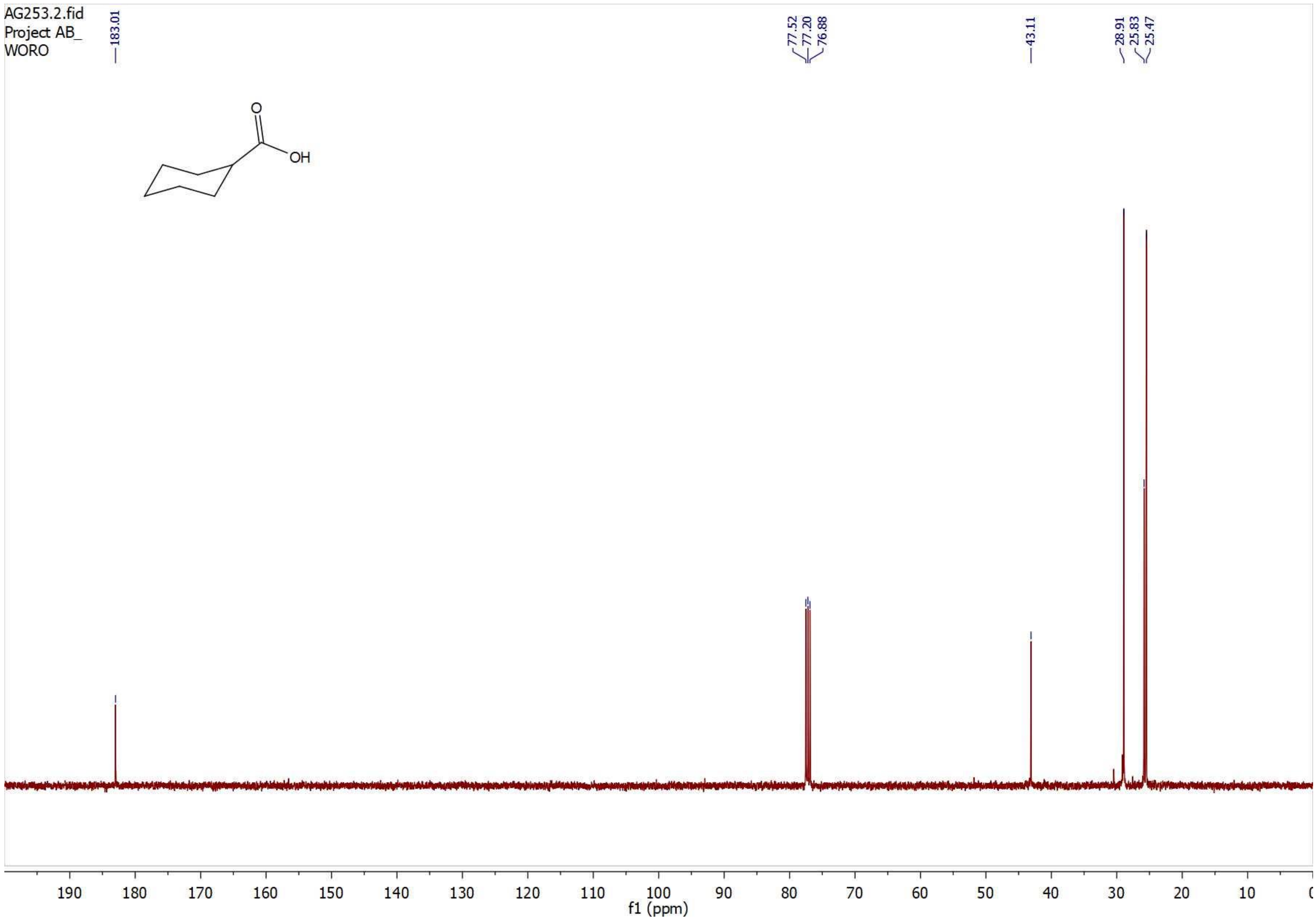
AG253.2.fid
Project AB_
WORO
183.01
77.52
77.20
76.88
43.11
28.91
25.83
25.47
OH
O
190
180
170
160
150
140
130
120
110
100
90
80
70
60
50
40
30
20
10
f1 (ppm)

Compound 2m

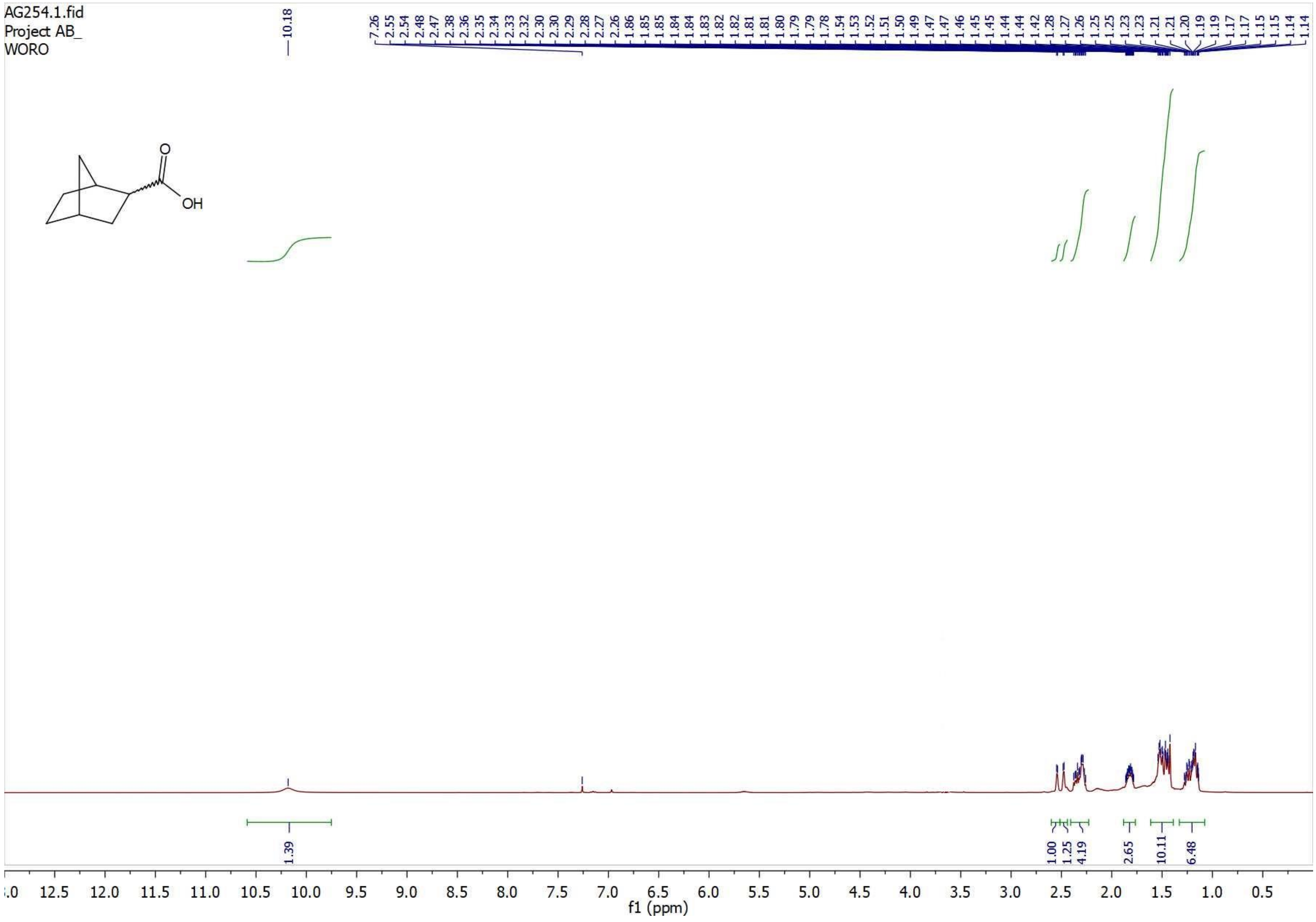
AG254.1.fid
Project AB_
WORO
OH
10.18
7.26
1.39
1.00
1.25
4.19
2.65
10.11
6.48
f1 (ppm)

Compound 2m

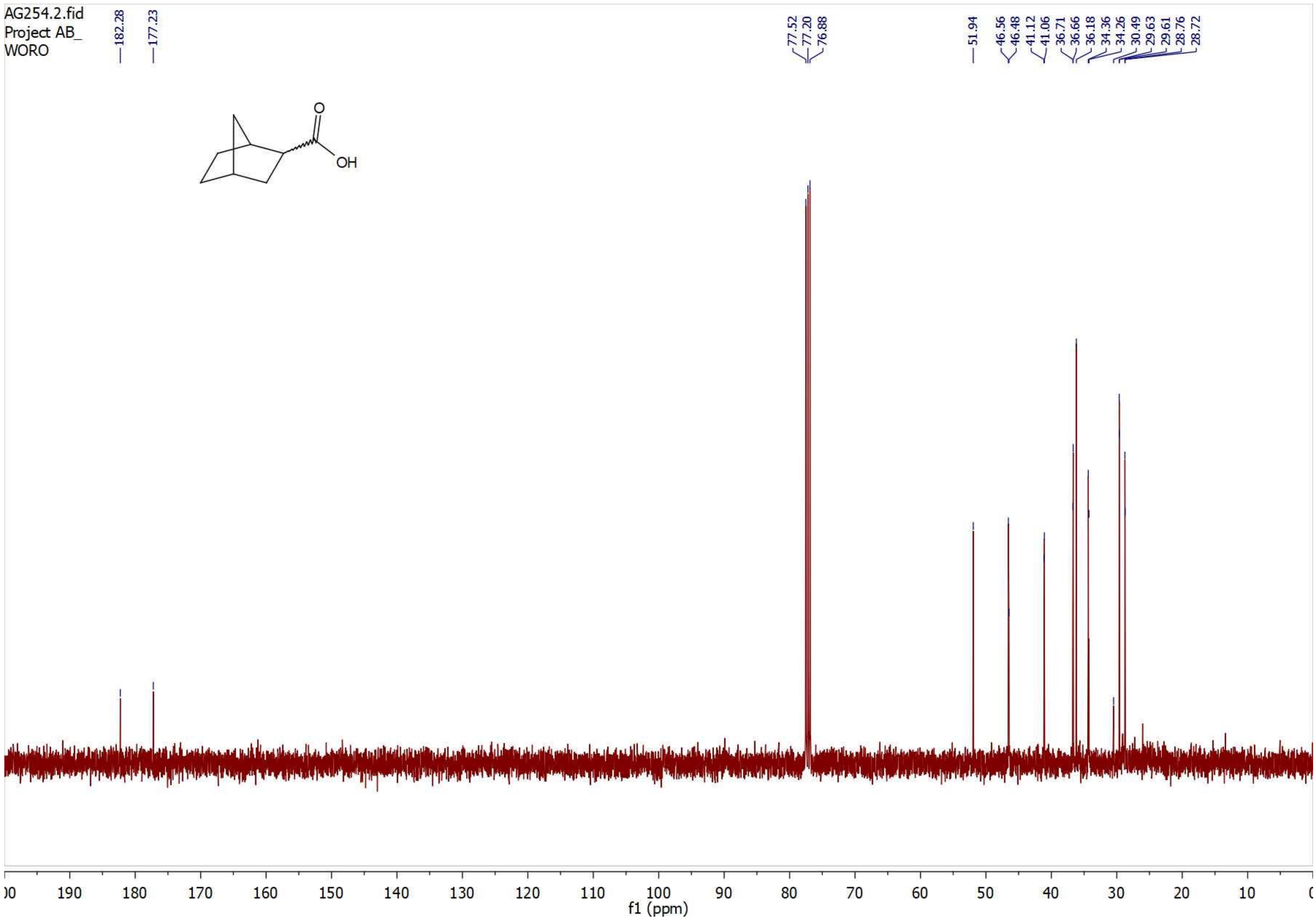
AG254.2.fid
Project AB_
WORO
182.28
177.23
77.52
77.20
76.88
51.94
46.56
46.48
41.12
41.06
36.71
36.66
36.18
34.36
34.26
30.49
29.63
29.61
28.76
28.72
O
OH
190
180
170
160
150
140
130
120
110
100
90
80
70
60
50
40
30
20
10
f1 (ppm)

Compound 2n

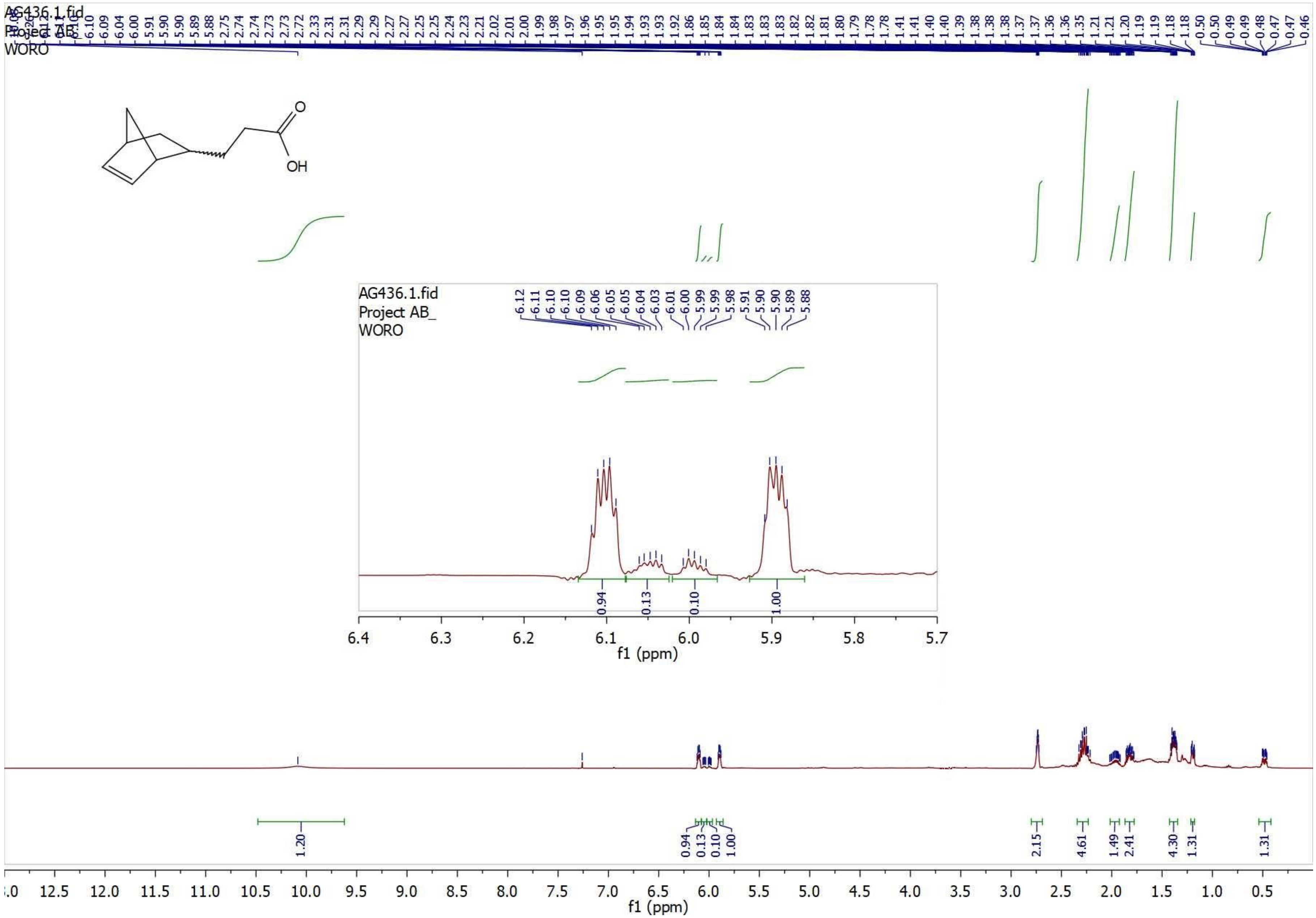

Compound 2n

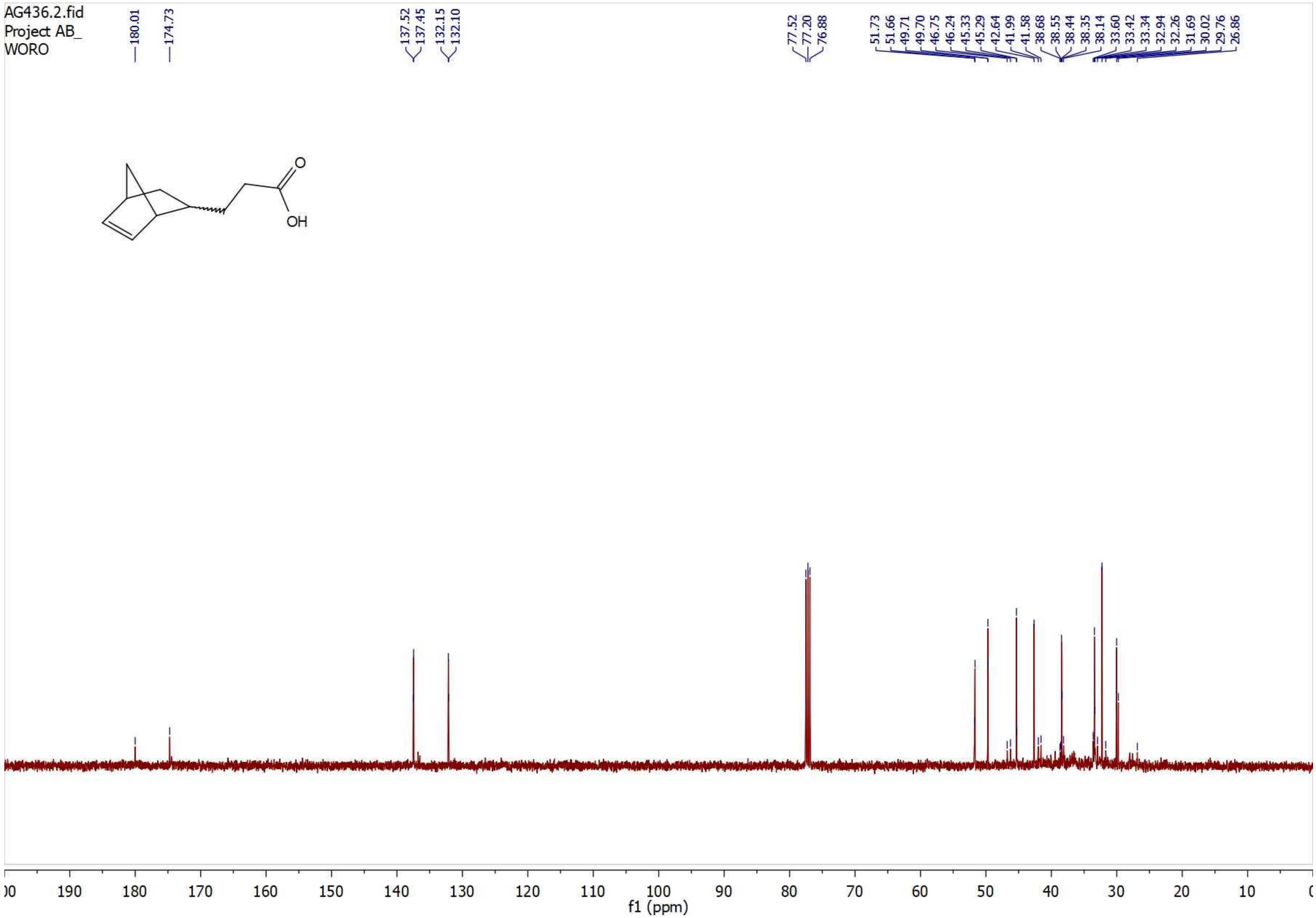
AG436.2.fid
Project AB_
WORO
180.01
174.73
137.52
137.45
132.15
132.10
77.52
77.20
76.88
51.73
51.66
49.71
49.70
46.75
46.24
45.33
45.29
42.64
41.99
41.58
38.68
38.55
38.44
38.35
38.14
33.60
33.42
33.34
32.94
32.26
31.69
30.02
29.76
26.86
O
OH
190
180
170
160
150
140
130
120
110
100
90
80
70
60
50
40
30
20
10
f1 (ppm)

Compound 2o

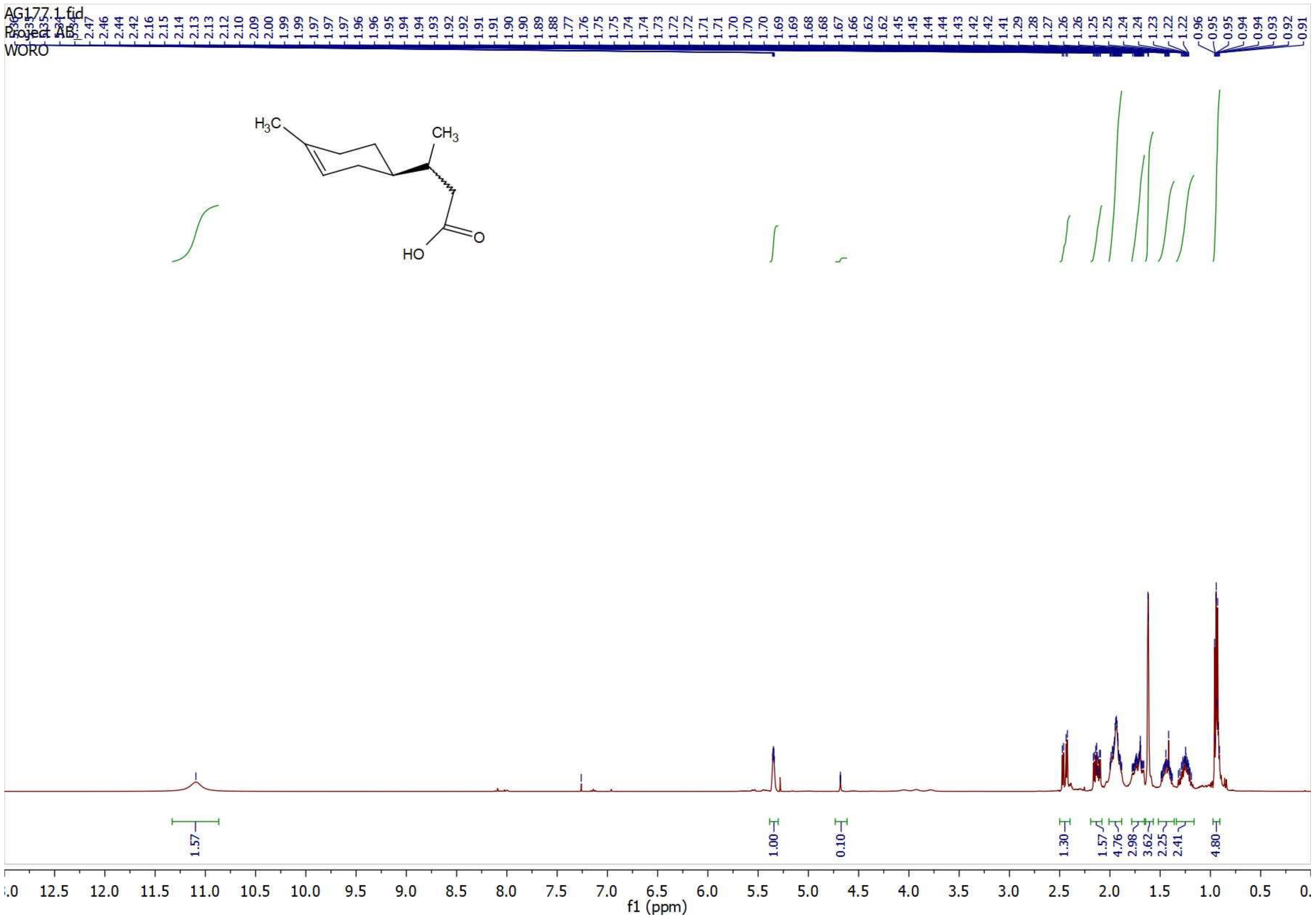

Compound 2o

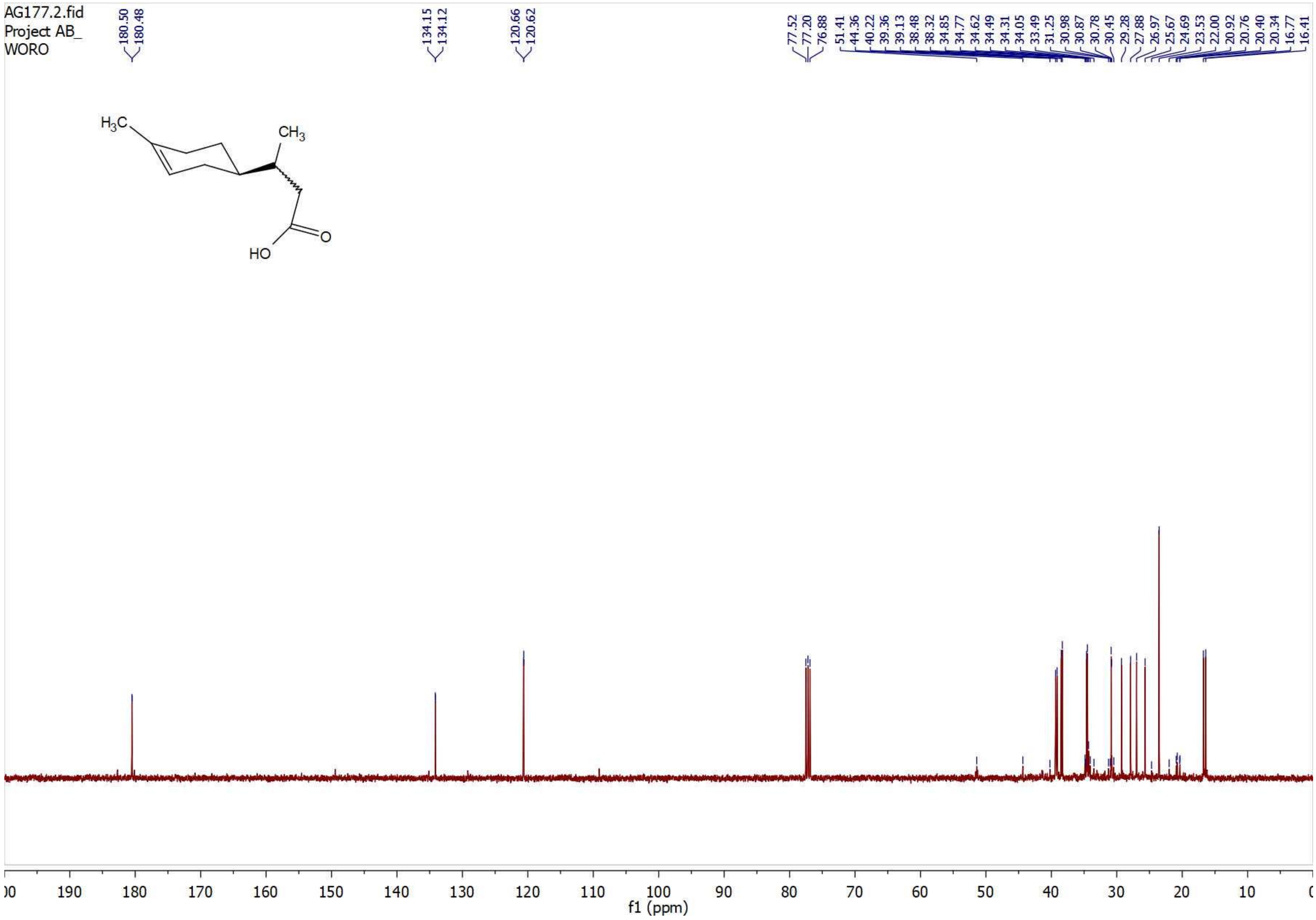

AG177.2.fid
Project AB_
WORO
180.50
180.48
134.15
134.12
120.66
120.62
77.52
77.20
76.88
51.41
44.36
40.22
39.36
39.13
38.48
38.32
34.85
34.77
34.62
34.49
34.31
34.05
33.49
31.25
30.98
30.87
30.78
30.45
29.28
27.88
26.97
25.67
24.69
23.53
22.00
20.92
20.76
20.40
20.34
16.77
16.41
H_3C
CH_3
HO
O
f1 (ppm)

Compound 2p

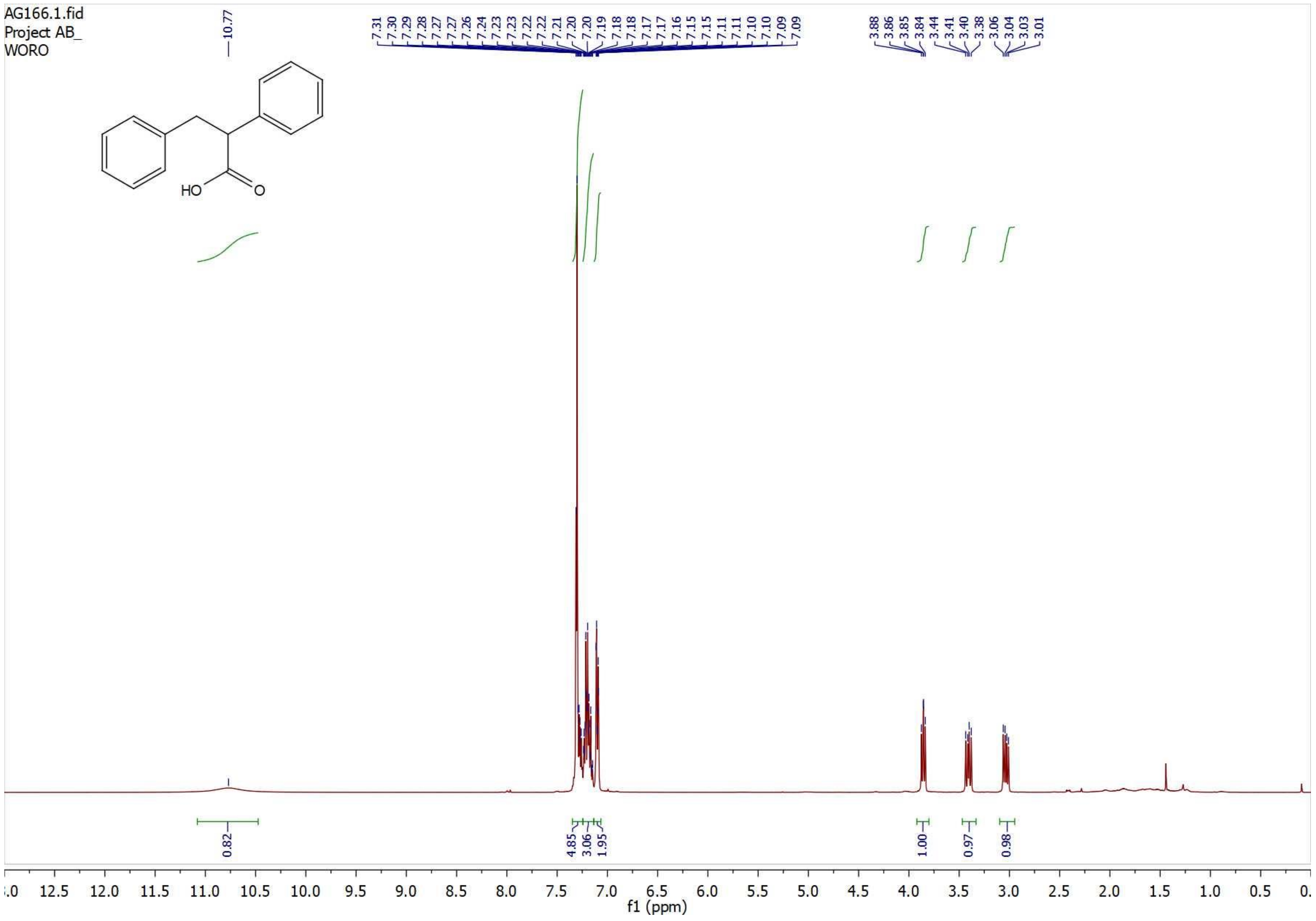
AG166.1.fid
Project AB_
WORO
10.77
7.31
7.30
7.29
7.28
7.27
7.27
7.26
7.24
7.23
7.23
7.22
7.22
7.21
7.20
7.20
7.19
7.18
7.18
7.17
7.17
7.16
7.15
7.15
7.11
7.11
7.10
7.10
7.09
7.09
3.88
3.86
3.85
3.84
3.44
3.41
3.40
3.38
3.06
3.04
3.03
3.01
HO
O
0.82
4.85
3.06
1.95
1.00
0.97
0.98
f1 (ppm)

Compound 2p

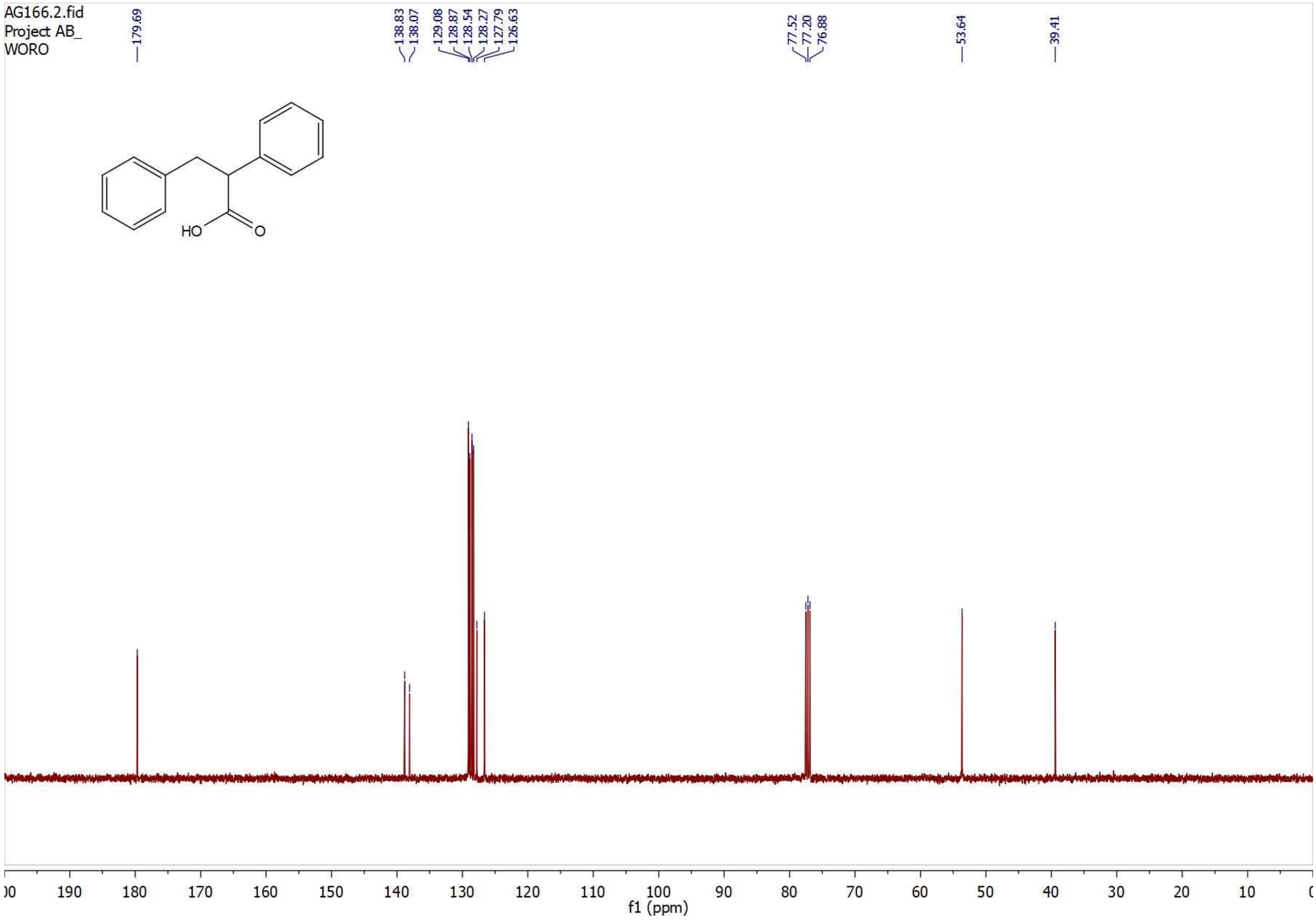
AG166.2.fid
Project AB_
WORO
179.69
138.83
138.07
129.08
128.87
128.54
128.27
127.79
126.63
77.52
77.20
76.88
53.64
39.41
HO
O
190
180
170
160
150
140
130
120
110
100
90
80
70
60
50
40
30
20
10
f1 (ppm)

Compound 2q

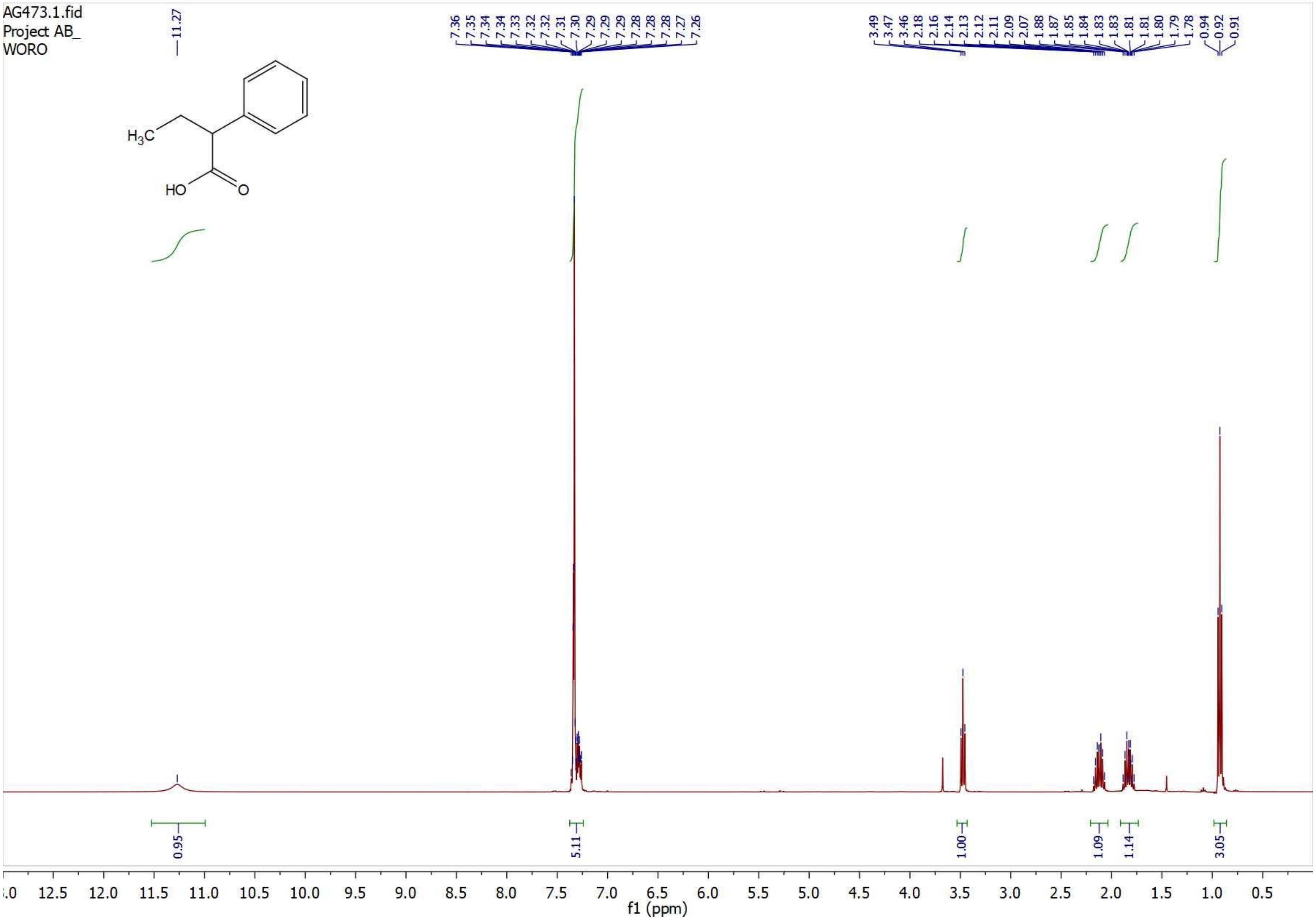
AG473.1.fid
Project AB_
WORO
11.27
7.36
7.35
7.34
7.34
7.33
7.32
7.32
7.31
7.30
7.29
7.29
7.29
7.28
7.28
7.28
7.27
7.26
3.49
3.47
3.46
2.18
2.16
2.14
2.13
2.12
2.11
2.09
2.07
1.88
1.87
1.85
1.84
1.83
1.83
1.81
1.81
1.80
1.79
1.78
0.94
0.92
0.91
H3C
HO
O
0.95
5.11
1.00
1.09
1.14
3.05
f1 (ppm)

Compound 2q

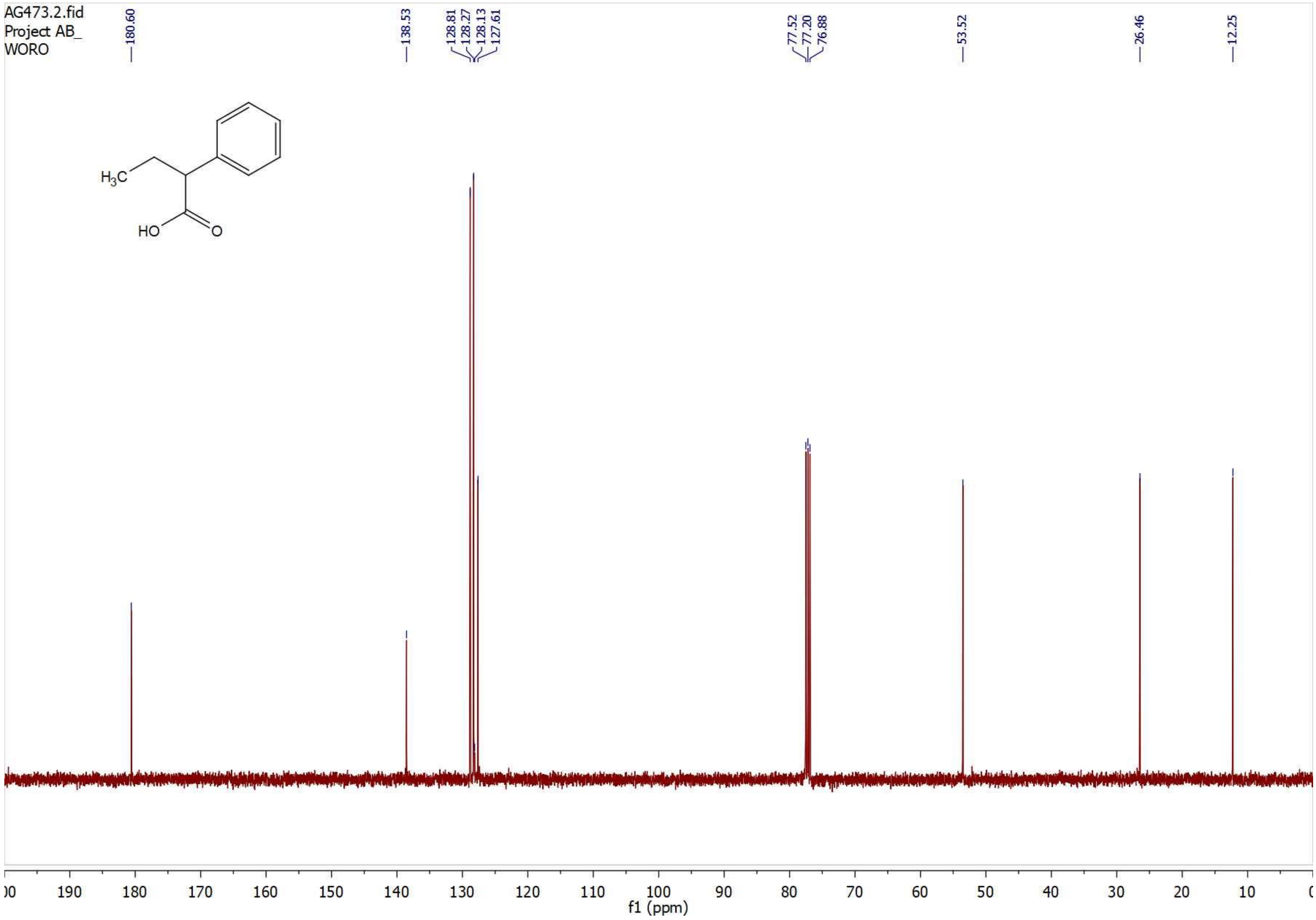
AG473.2.fid
Project AB_
WORO
180.60
138.53
128.81
128.27
128.13
127.61
77.52
77.20
76.88
53.52
26.46
12.25
H3C
HO
O
f1 (ppm)

Compound 2r

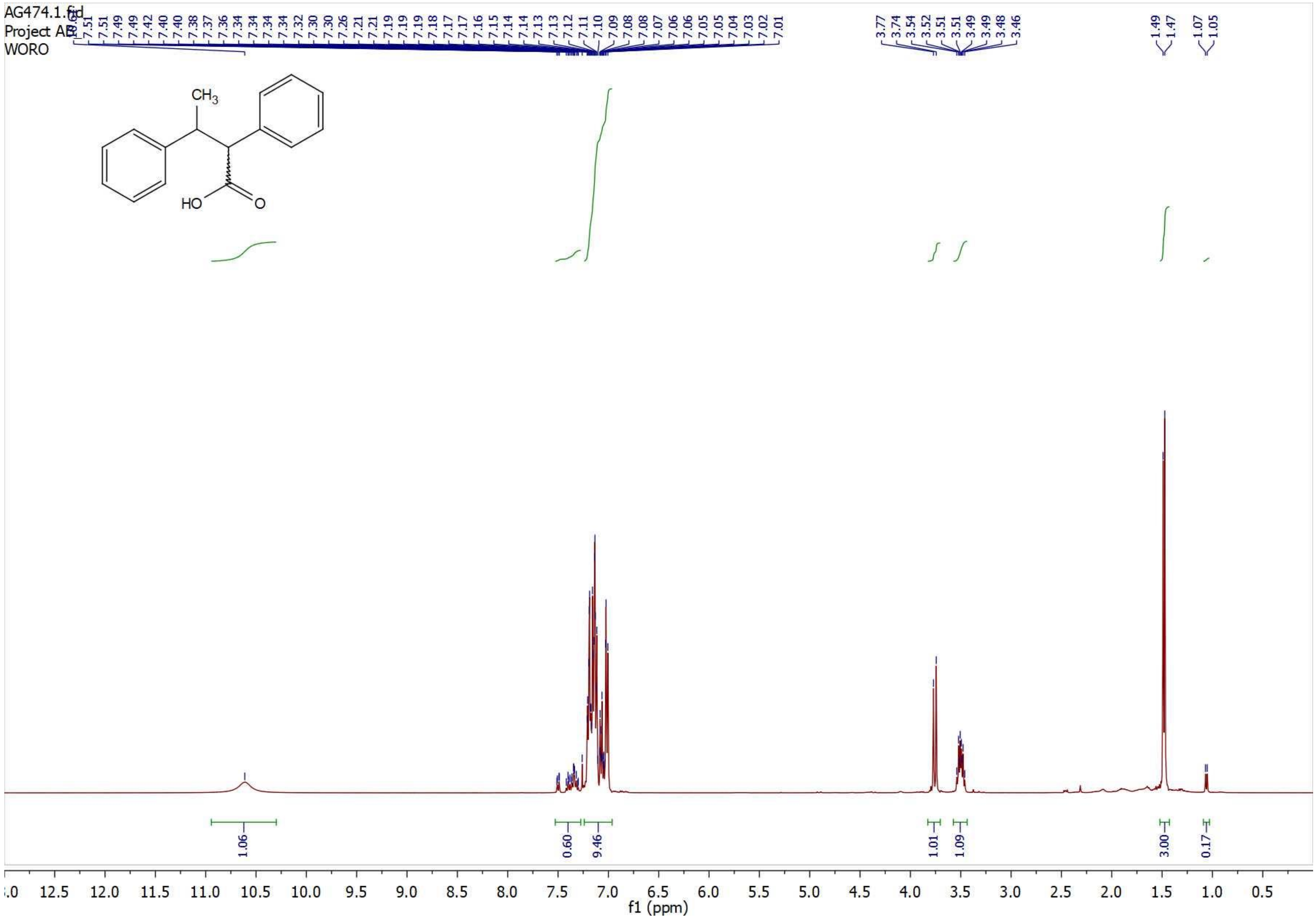

Compound 2r

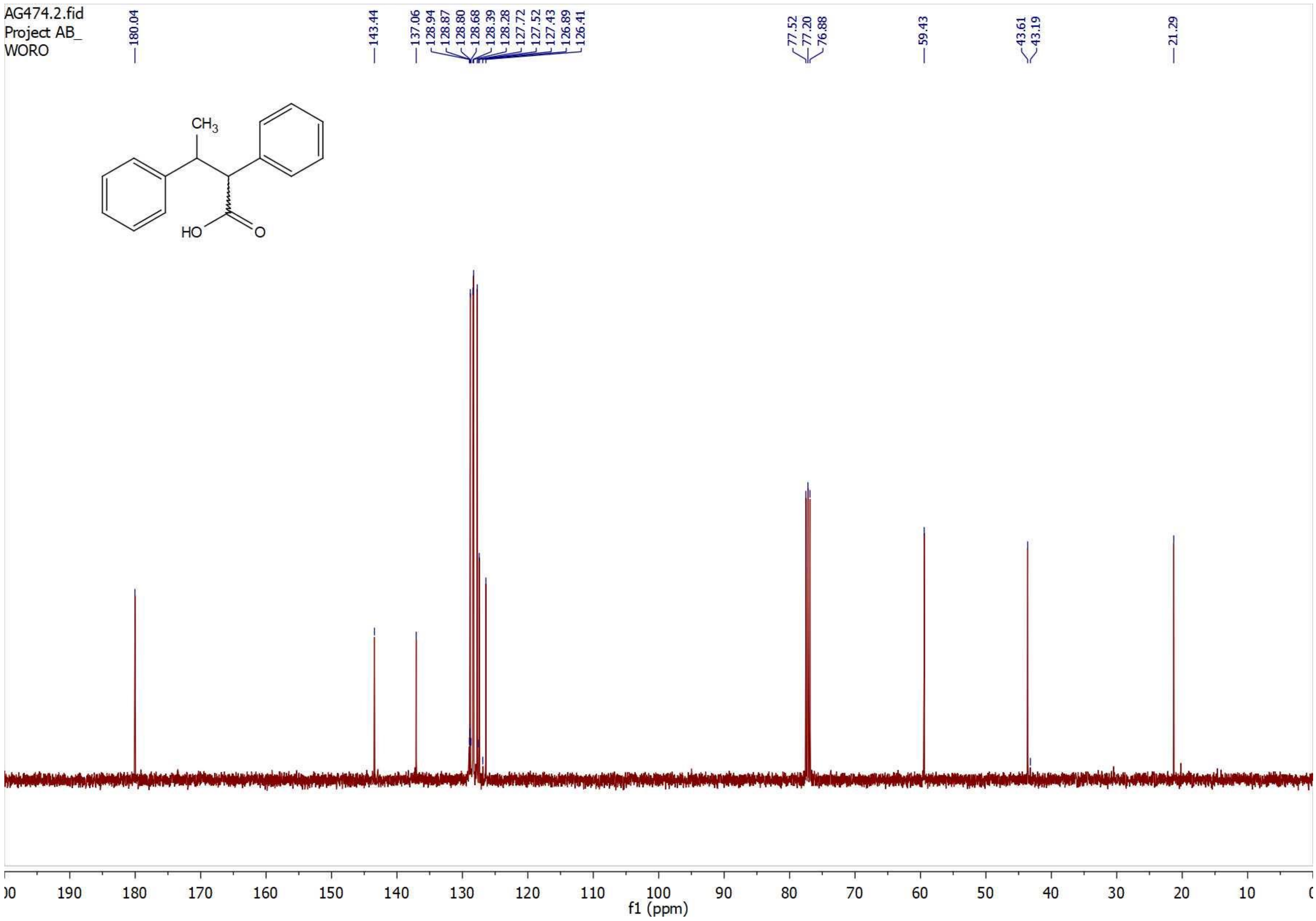

AG474.2.fid
Project AB_
WORO
180.04
143.44
137.06
128.94
128.87
128.80
128.68
128.39
128.28
127.72
127.52
127.43
126.89
126.41
77.52
77.20
76.88
59.43
43.61
43.19
21.29
CH3
HO
O
190
180
170
160
150
140
130
120
110
100
90
80
70
60
50
40
30
20
10
f1 (ppm)

Compound 2s

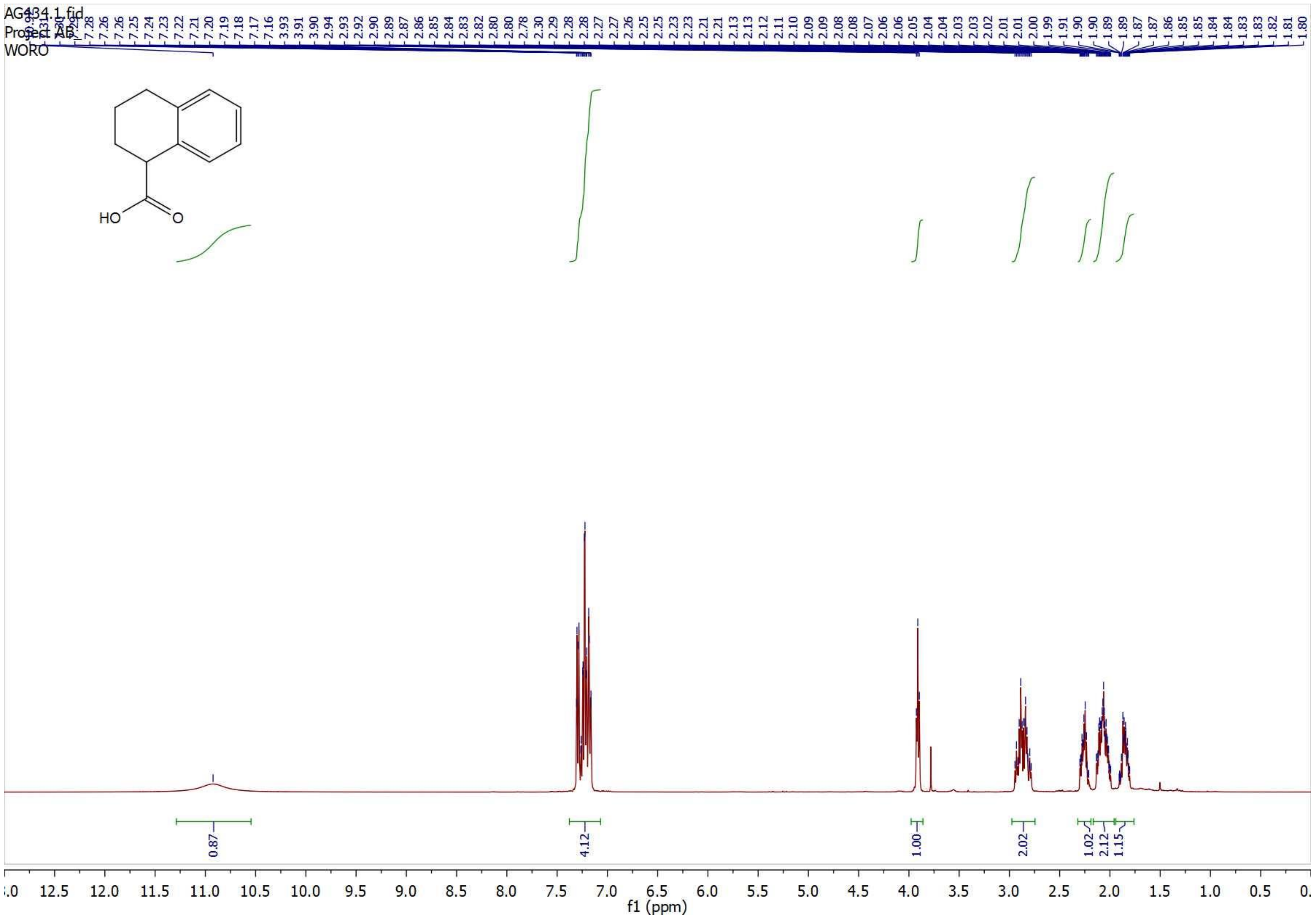
AG434.1.fid
Project AB
WORO
HO
O
0.87
4.12
1.00
2.02
1.02
2.12
1.15
f1 (ppm)

Compound 2s

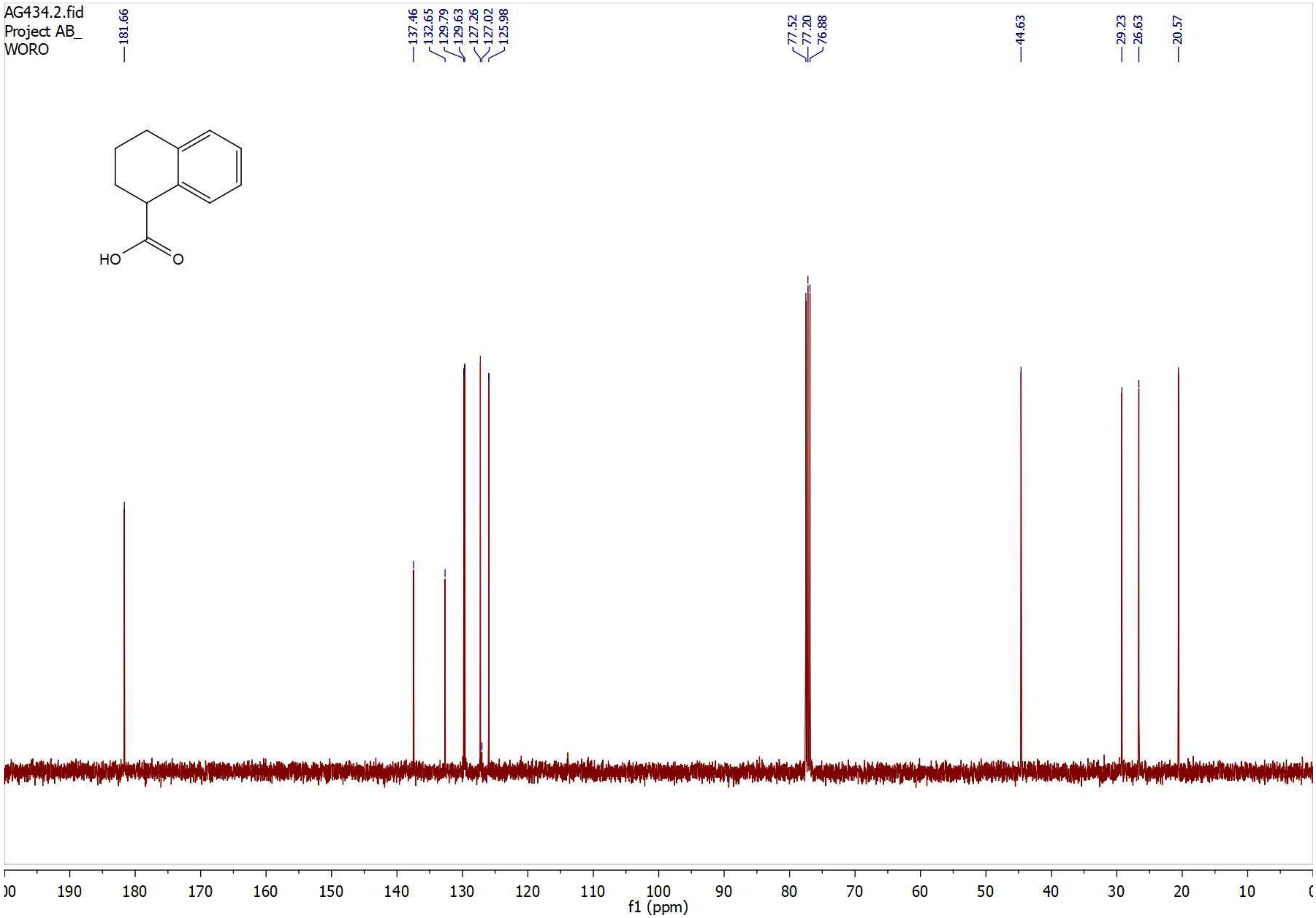

AG434.2.fid
Project AB_
WORO
181.66
137.46
132.65
129.79
129.63
127.26
127.02
125.98
77.52
77.20
76.88
44.63
29.23
26.63
20.57
HO
O
190
180
170
160
150
140
130
120
110
100
90
80
70
60
50
40
30
20
10
f1 (ppm)

Compound 4a

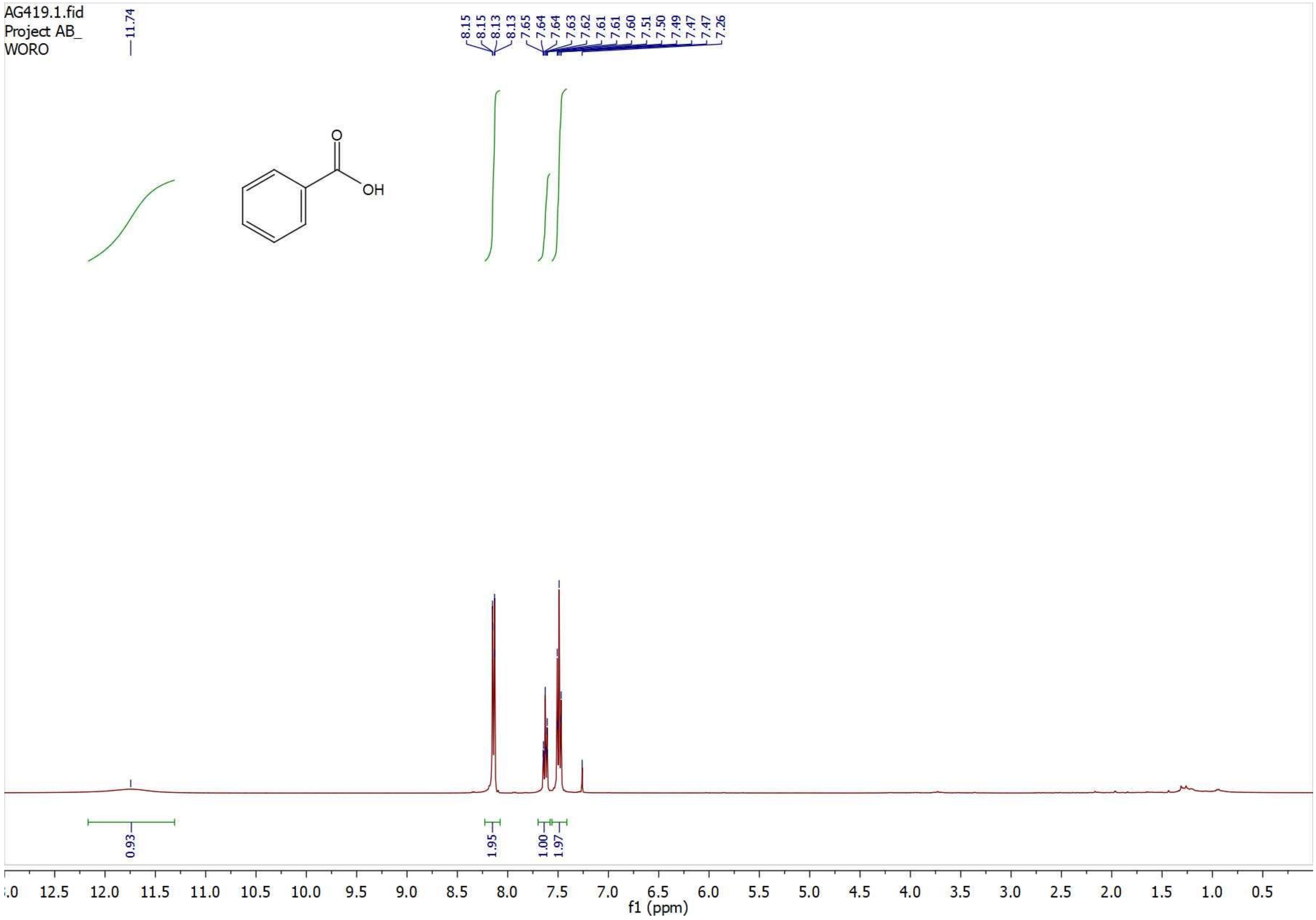

AG419.1.fid
Project AB_
WORO
11.74
8.15 8.15 8.13 8.13 7.65 7.64 7.64 7.63 7.62 7.61 7.61 7.60 7.51 7.50 7.49 7.47 7.47 7.26
OH
O
0.93
1.95
1.00
1.97
f1 (ppm)

Compound 4a

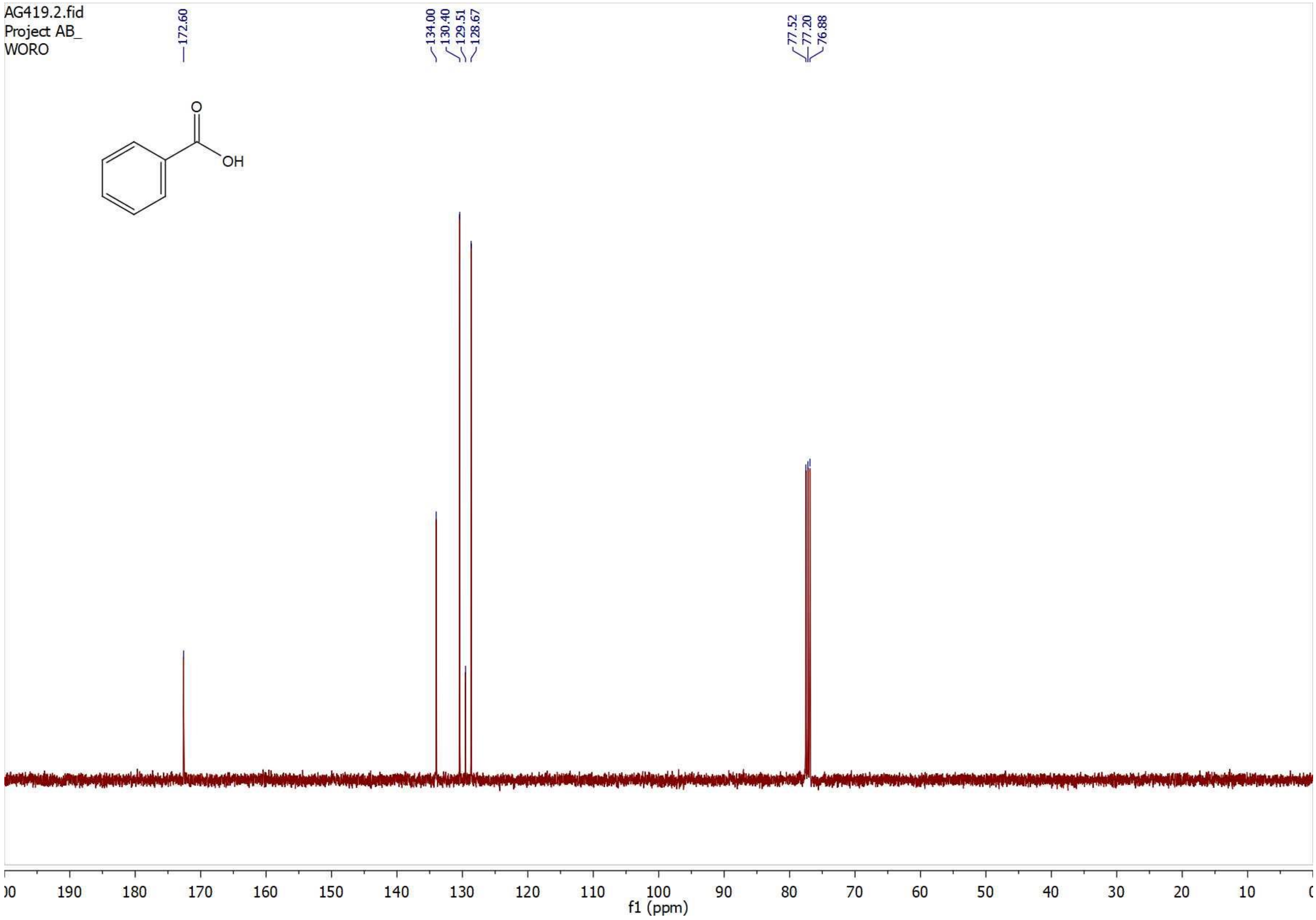
AG419.2.fid
Project AB_
WORO
172.60
134.00
130.40
129.51
128.67
77.52
77.20
76.88
OH
O
190
180
170
160
150
140
130
120
110
100
90
80
70
60
50
40
30
20
10
f1 (ppm)

Compound 4b

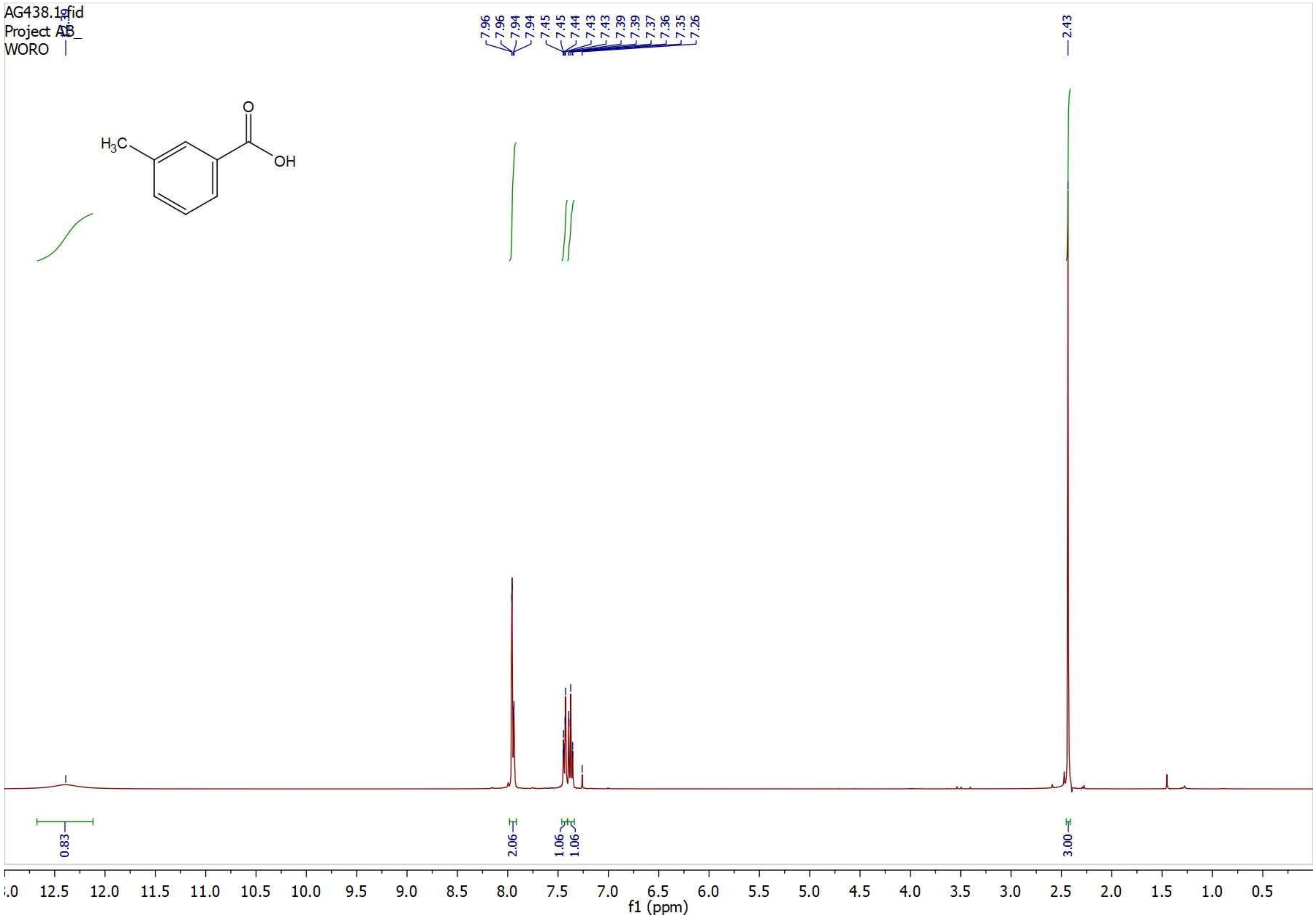

AG438.1.fid
Project AB_
WORO
7.96
7.96
7.94
7.94
7.45
7.45
7.44
7.43
7.43
7.39
7.39
7.37
7.36
7.35
7.26
2.43
H_3C
O
OH
0.83
2.06
1.06
1.06
3.00
12.5
12.0
11.5
11.0
10.5
10.0
9.5
9.0
8.5
8.0
7.5
7.0
6.5
6.0
5.5
5.0
4.5
4.0
3.5
3.0
2.5
2.0
1.5
1.0
0.5
f1 (ppm)

Compound 4b

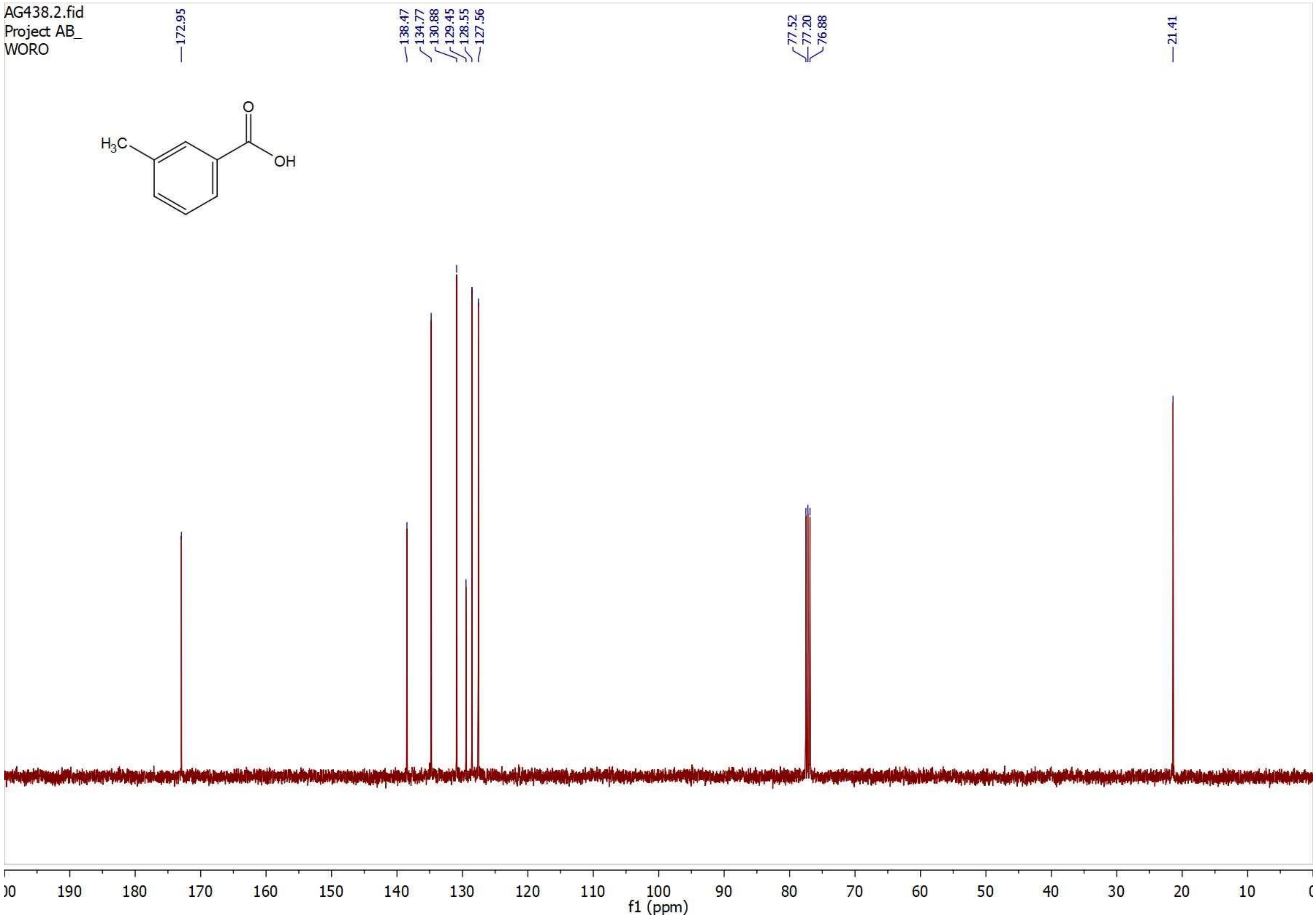
AG438.2.fid
Project AB_
WORO
172.95
138.47
134.77
130.88
129.45
128.55
127.56
77.52
77.20
76.88
21.41
O
H_3C
OH
190
180
170
160
150
140
130
120
110
100
90
80
70
60
50
40
30
20
10
f1 (ppm)

Compound 4c

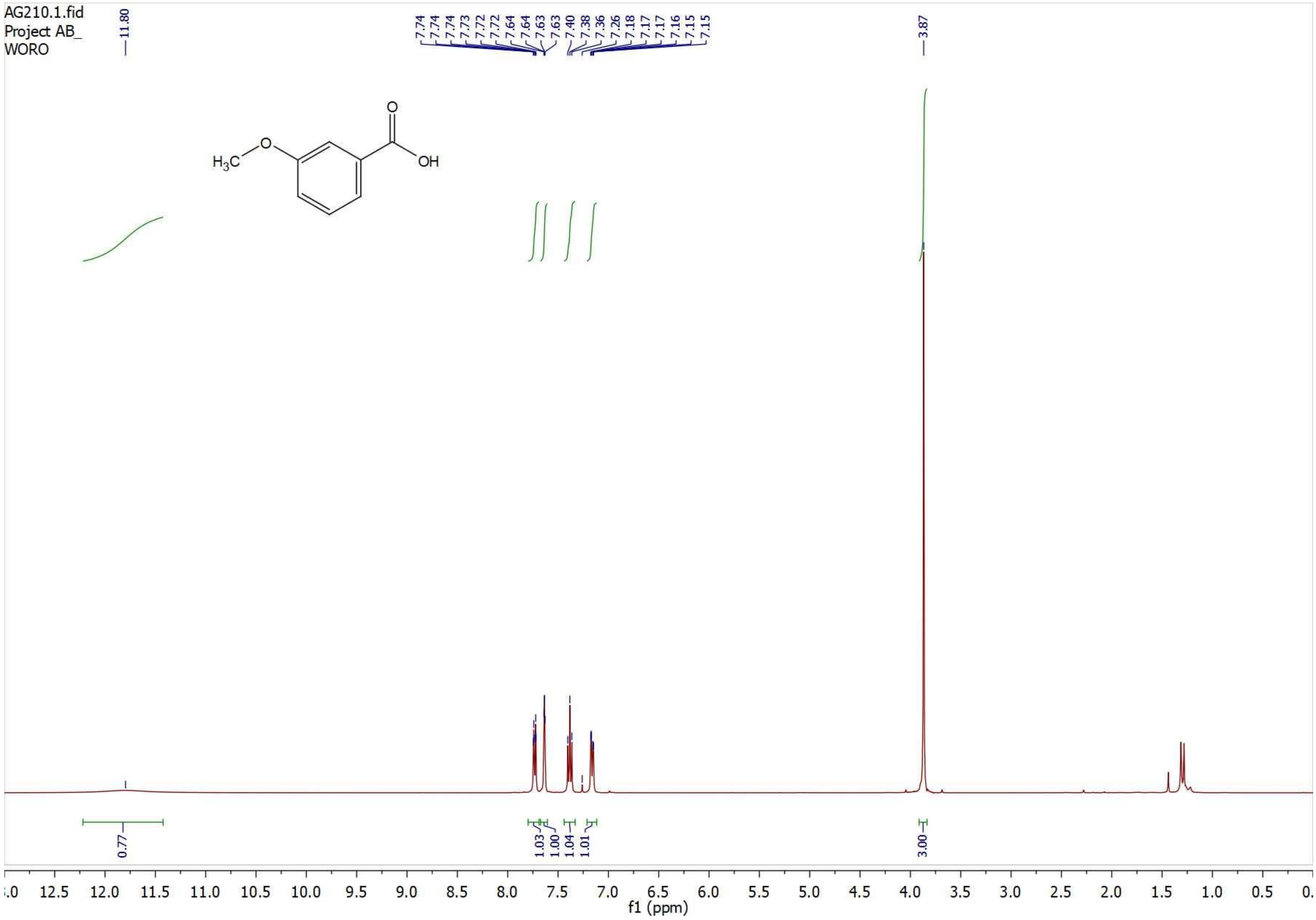
AG210.1.fid
Project AB_
WORO
11.80
7.74
7.74
7.74
7.73
7.72
7.72
7.64
7.64
7.63
7.63
7.40
7.38
7.36
7.26
7.18
7.17
7.17
7.16
7.15
7.15
3.87
O
OH
H3C
O
0.77
1.03
1.00
1.04
1.01
3.00
12.5
12.0
11.5
11.0
10.5
10.0
9.5
9.0
8.5
8.0
7.5
7.0
6.5
6.0
5.5
5.0
4.5
4.0
3.5
3.0
2.5
2.0
1.5
1.0
0.5
f1 (ppm)

Compound 4c

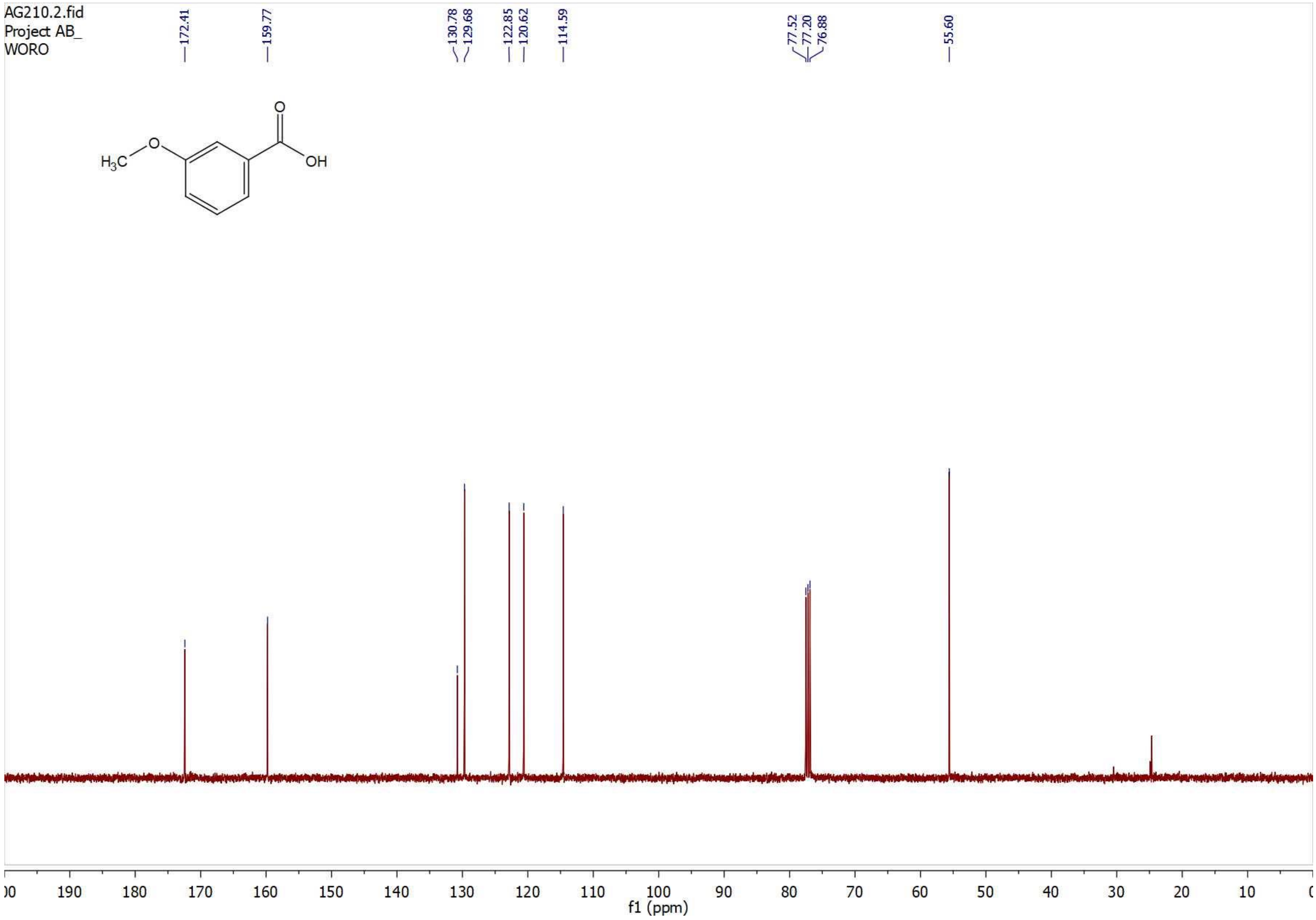
AG210.2.fid
Project AB_
WORO
172.41
159.77
130.78
129.68
122.85
120.62
114.59
77.52
77.20
76.88
55.60
O
OH
H3C
O
190
180
170
160
150
140
130
120
110
100
90
80
70
60
50
40
30
20
10
f1 (ppm)

Compound 4d

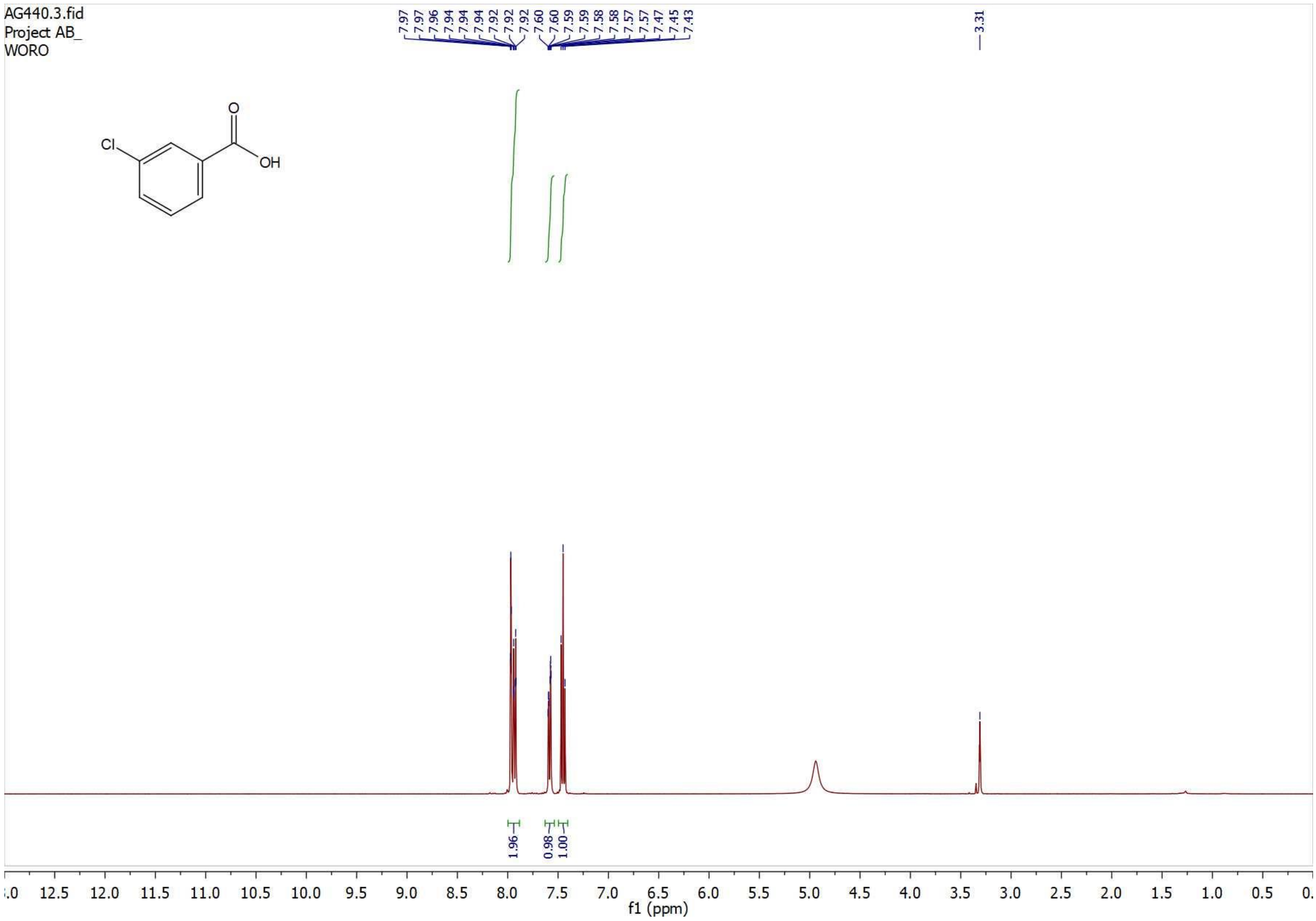
AG440.3.fid
Project AB_
WORO
Cl
O
OH
7.97
7.97
7.96
7.94
7.94
7.94
7.92
7.92
7.92
7.60
7.60
7.59
7.59
7.58
7.58
7.57
7.57
7.47
7.45
7.43
3.31
1.96
0.98
1.00
f1 (ppm)

Compound 4d

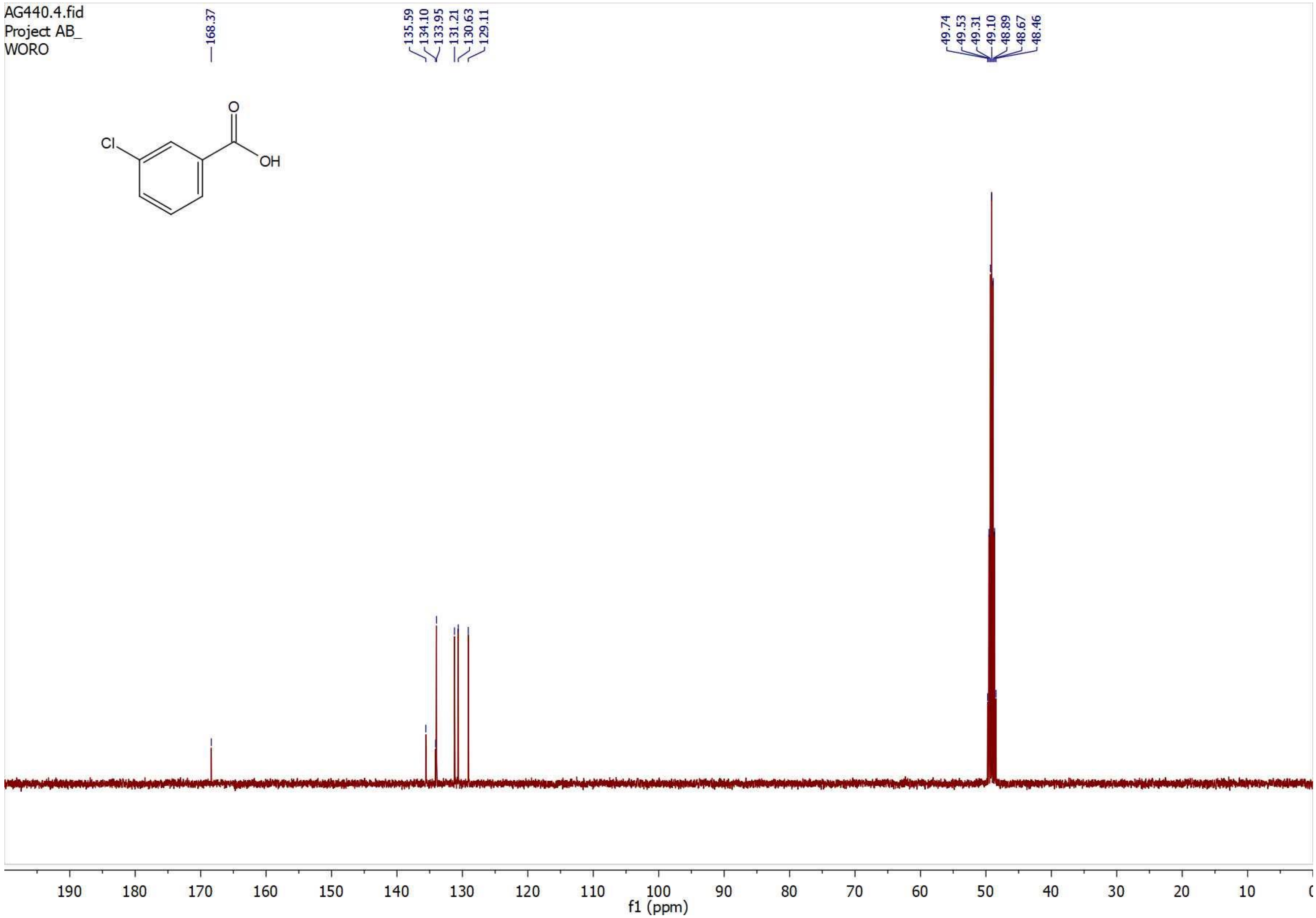
AG440.4.fid
Project AB_
WORO
168.37
135.59
134.10
133.95
131.21
130.63
129.11
49.74
49.53
49.31
49.10
48.89
48.67
48.46
Cl
O
OH
190 180 170 160 150 140 130 120 110 100 90 80 70 60 50 40 30 20 10
f1 (ppm)

Compound 4e

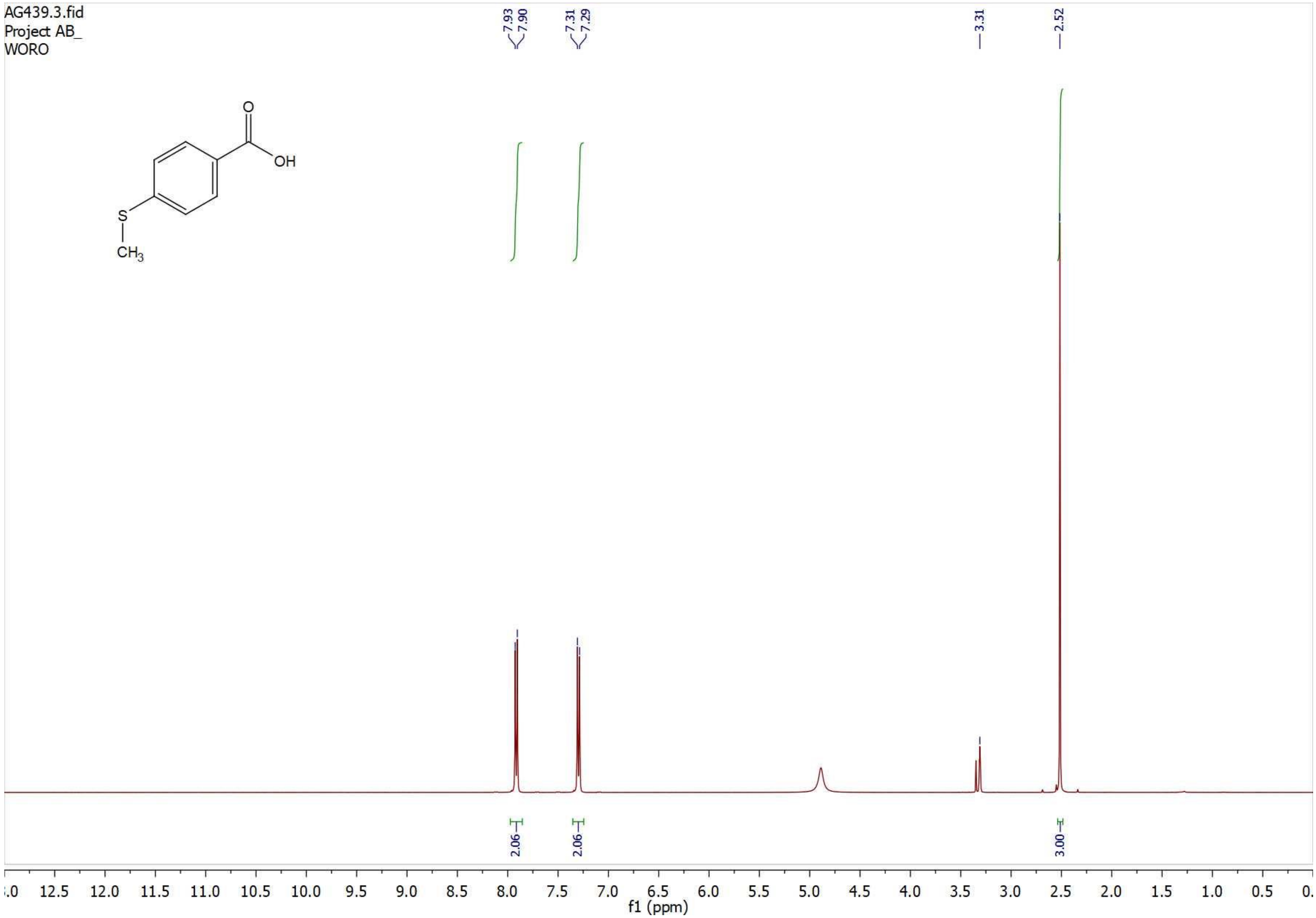
AG439.3.fid
Project AB_
WORO
O
OH
S
CH3
7.93
7.90
7.31
7.29
3.31
2.52
2.06
2.06
3.00
12.5 12.0 11.5 11.0 10.5 10.0 9.5 9.0 8.5 8.0 7.5 7.0 6.5 6.0 5.5 5.0 4.5 4.0 3.5 3.0 2.5 2.0 1.5 1.0 0.5
f1 (ppm)

Compound 4e

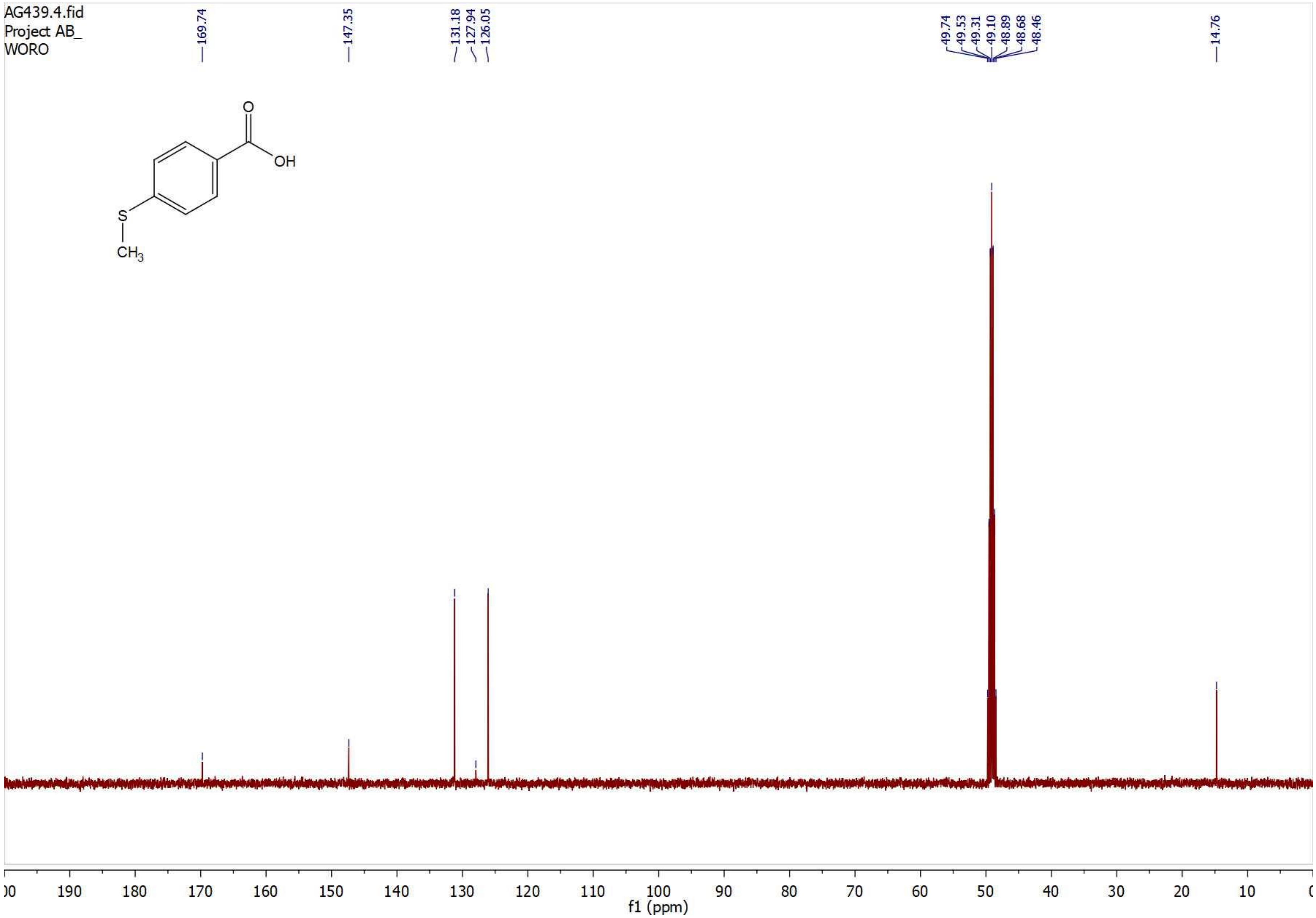
AG439.4.fid
Project AB_
WORO
169.74
147.35
131.18
127.94
126.05
49.74
49.53
49.31
49.10
48.89
48.68
48.46
14.76
O
OH
S
CH3
f1 (ppm)

Compound 4f

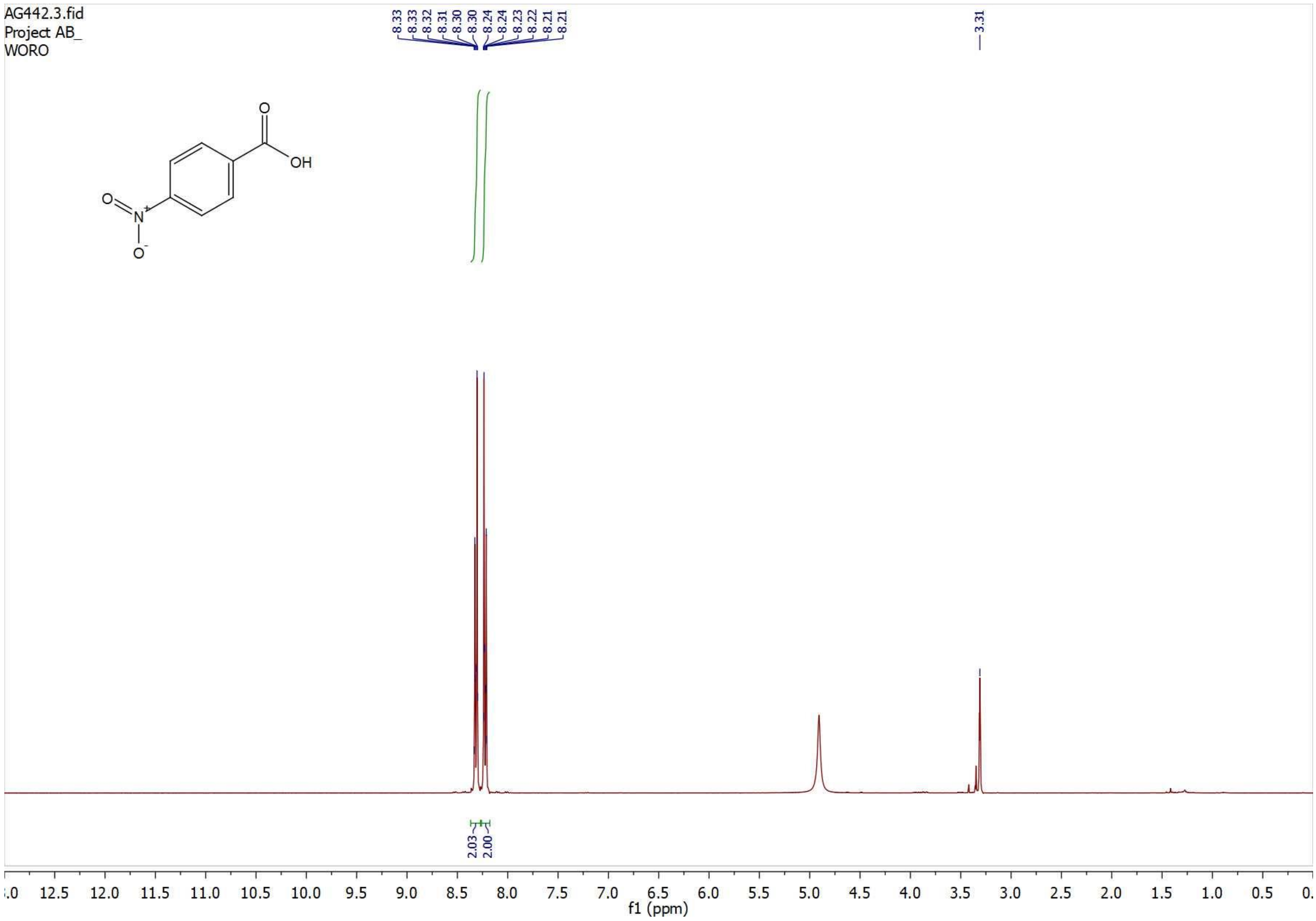

AG442.3.fid
Project AB_
WORO
8.33
8.33
8.32
8.31
8.30
8.24
8.24
8.23
8.22
8.21
8.21
3.31
OH
O
N
2.03
2.00
12.5 12.0 11.5 11.0 10.5 10.0 9.5 9.0 8.5 8.0 7.5 7.0 6.5 6.0 5.5 5.0 4.5 4.0 3.5 3.0 2.5 2.0 1.5 1.0 0.5
f1 (ppm)

Compound 4f

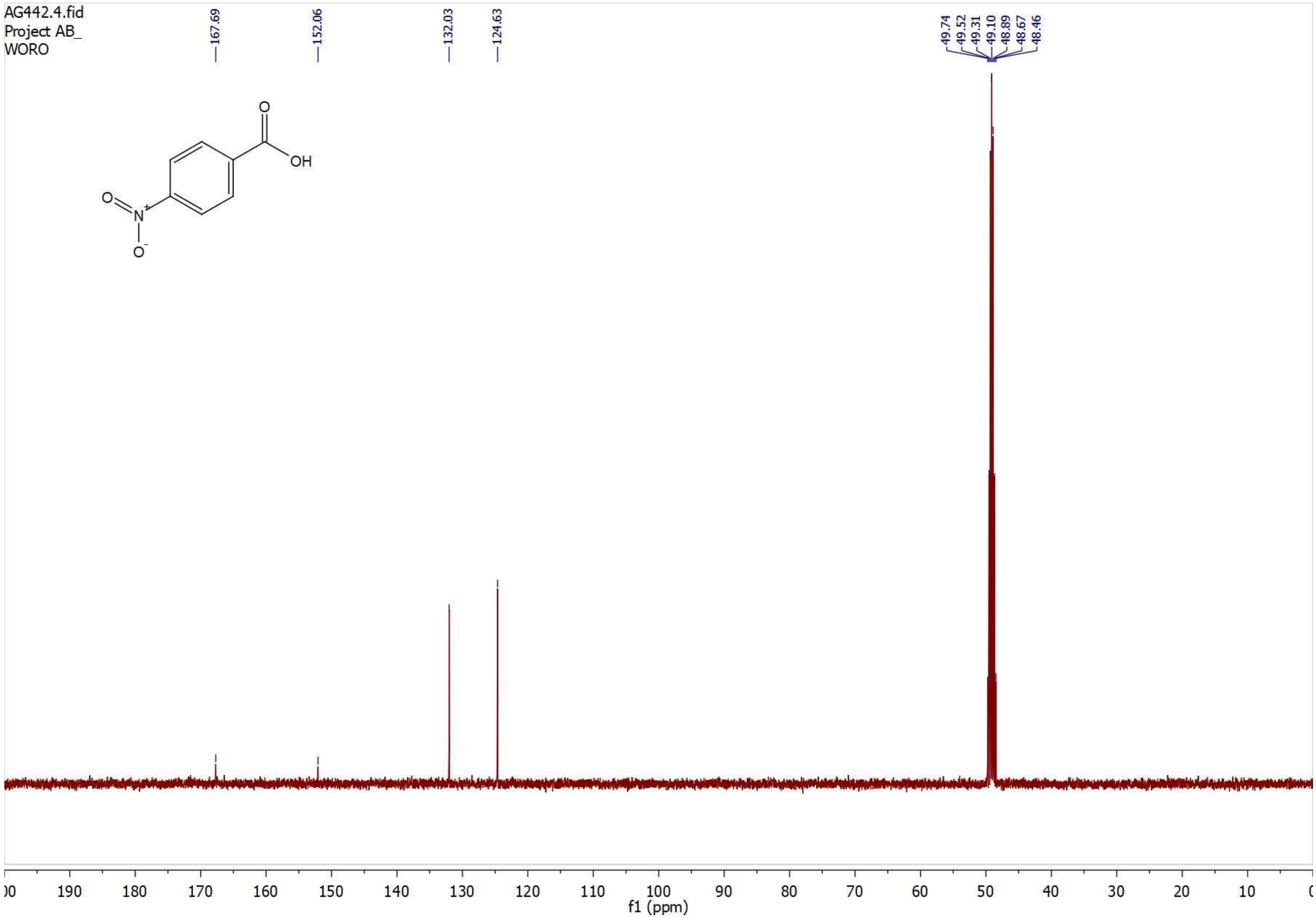

AG442.4.fid
Project AB_
WORO
167.69
152.06
132.03
124.63
49.74
49.52
49.31
49.10
48.89
48.67
48.46
O
OH
O
N
O
f1 (ppm)
190
180
170
160
150
140
130
120
110
100
90
80
70
60
50
40
30
20
10

Compound 4g

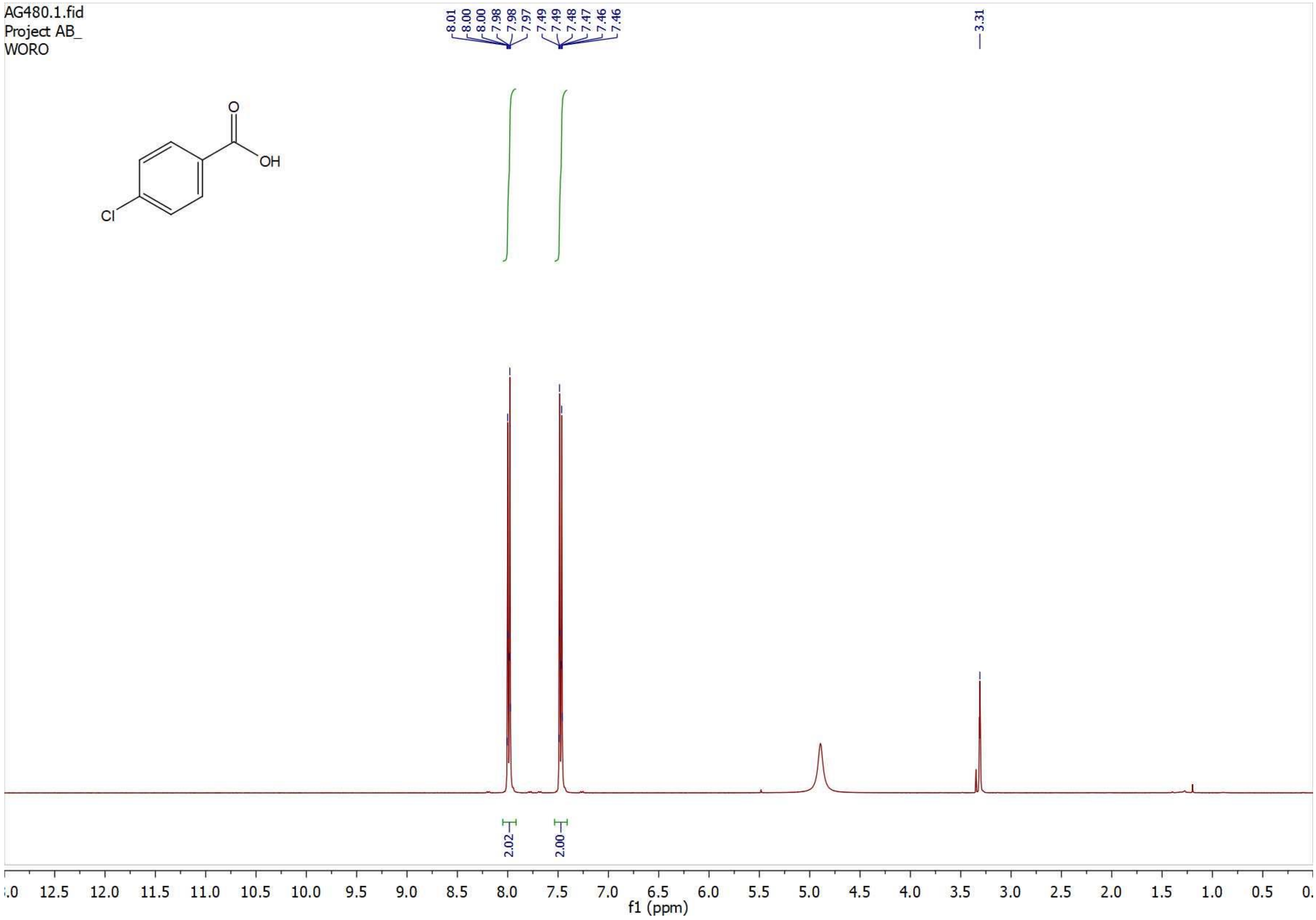
AG480.1.fid
Project AB_
WORO
8.01
8.00
8.00
7.98
7.98
7.97
7.49
7.49
7.48
7.47
7.46
7.46
3.31
O
OH
Cl
2.02
2.00
f1 (ppm)

Compound 4g

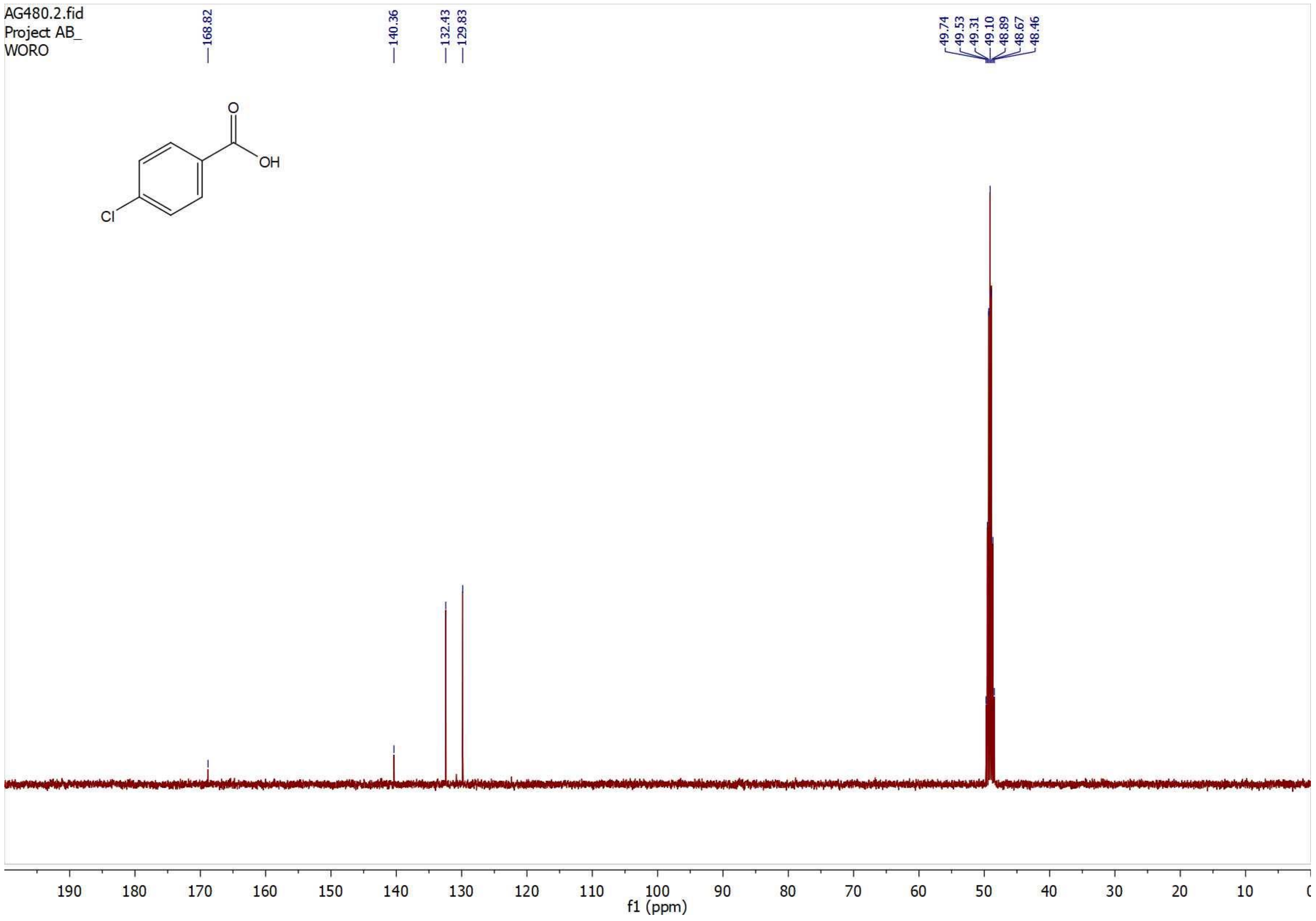

AG480.2.fid
Project AB_
WORO
168.82
140.36
132.43
129.83
49.74
49.53
49.31
49.10
48.89
48.67
48.46
O
OH
Cl
190
180
170
160
150
140
130
120
110
100
90
80
70
60
50
40
30
20
10
f1 (ppm)

Compound 4h

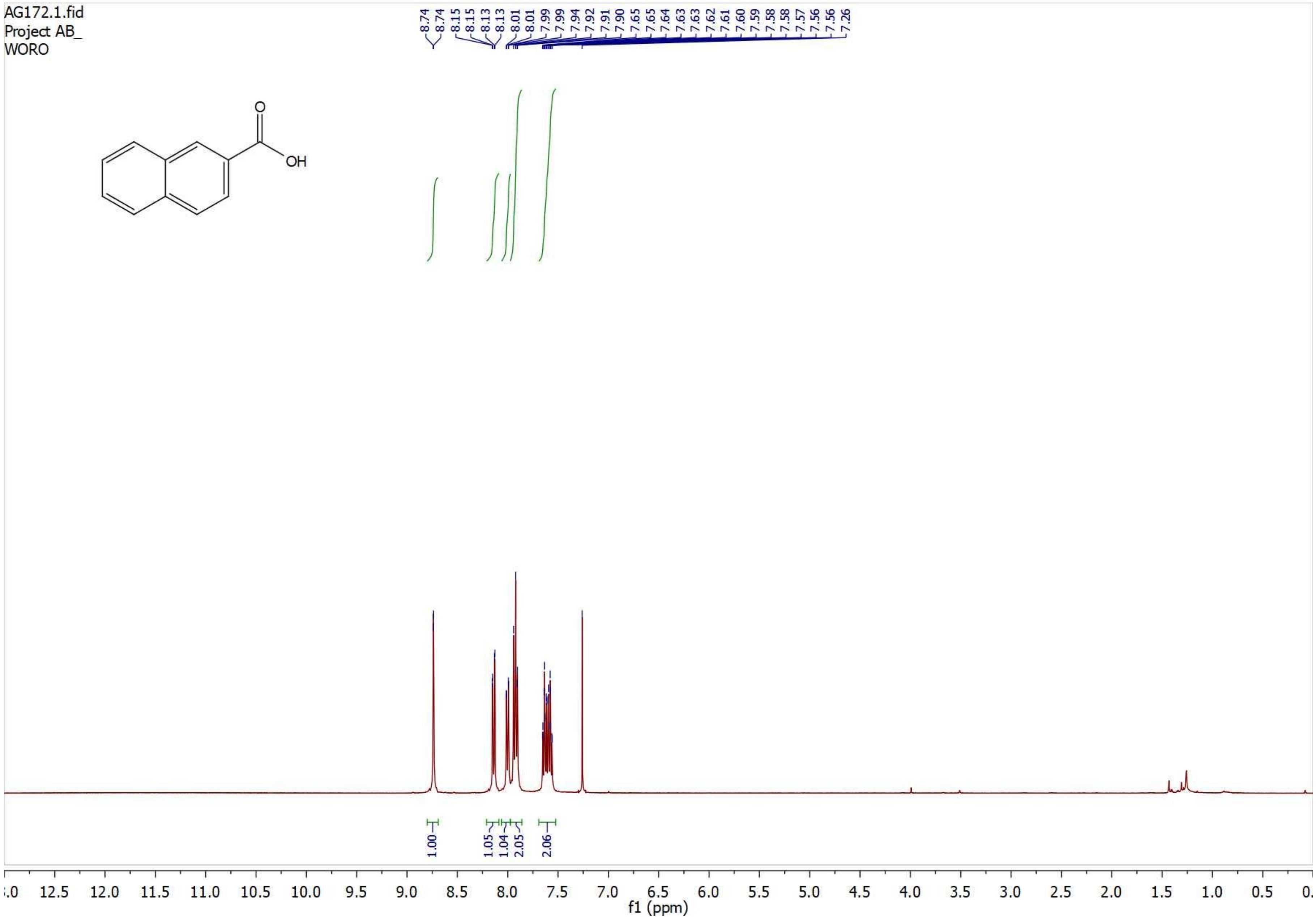
AG172.1.fid
Project AB_
WORO
OH
O
8.74
8.74
8.15
8.15
8.13
8.13
8.01
8.01
7.99
7.99
7.94
7.92
7.91
7.90
7.65
7.65
7.64
7.63
7.63
7.62
7.61
7.60
7.59
7.58
7.58
7.57
7.56
7.56
7.26
1.00
1.05
1.04
2.05
2.06
f1 (ppm)

Compound 4h

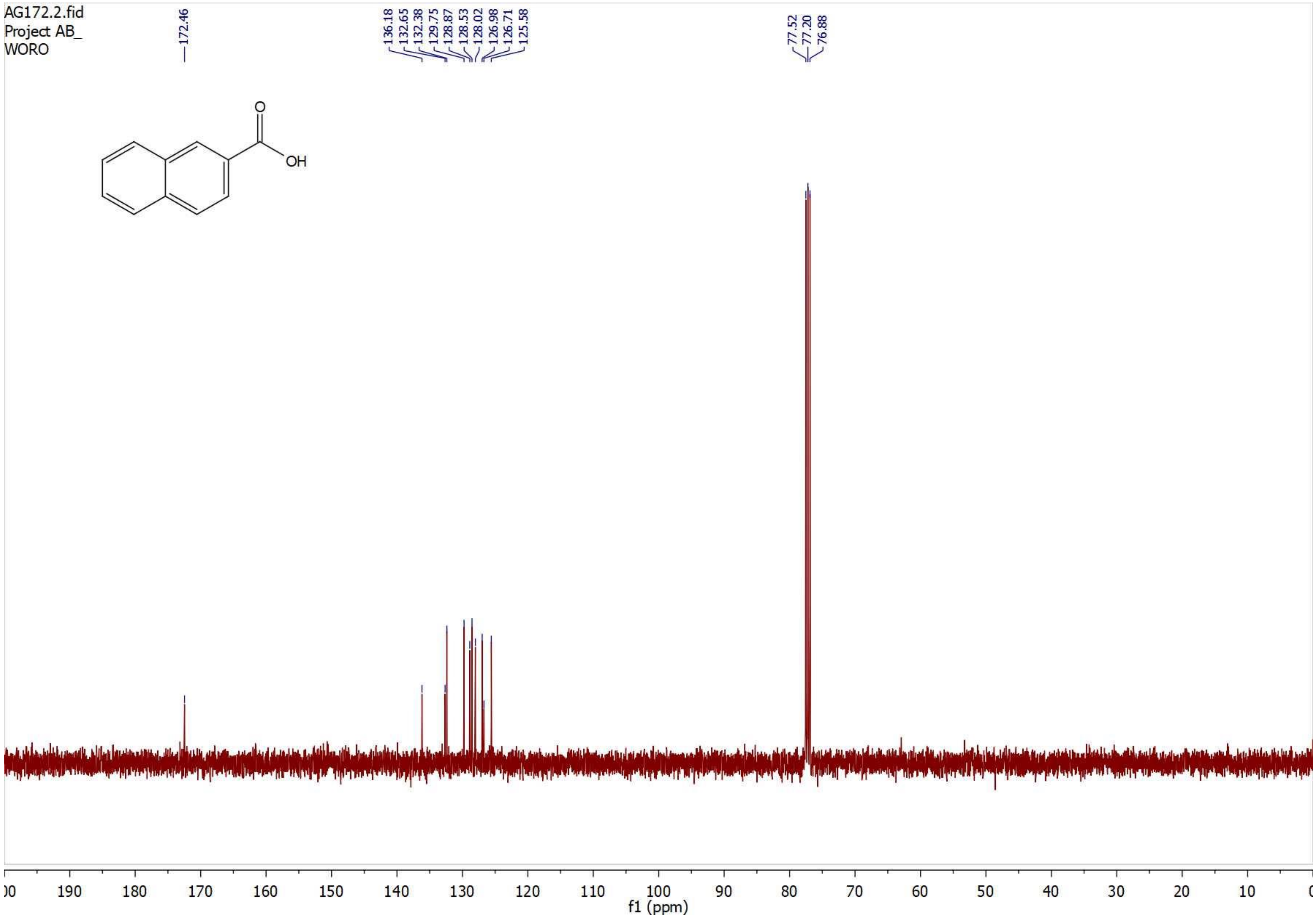
AG172.2.fid
Project AB_
WORO
172.46
136.18
132.65
132.38
129.75
128.87
128.53
128.02
126.98
126.71
125.58
77.52
77.20
76.88
OH
O
190
180
170
160
150
140
130
120
110
100
90
80
70
60
50
40
30
20
10
f1 (ppm)

Compound 4i

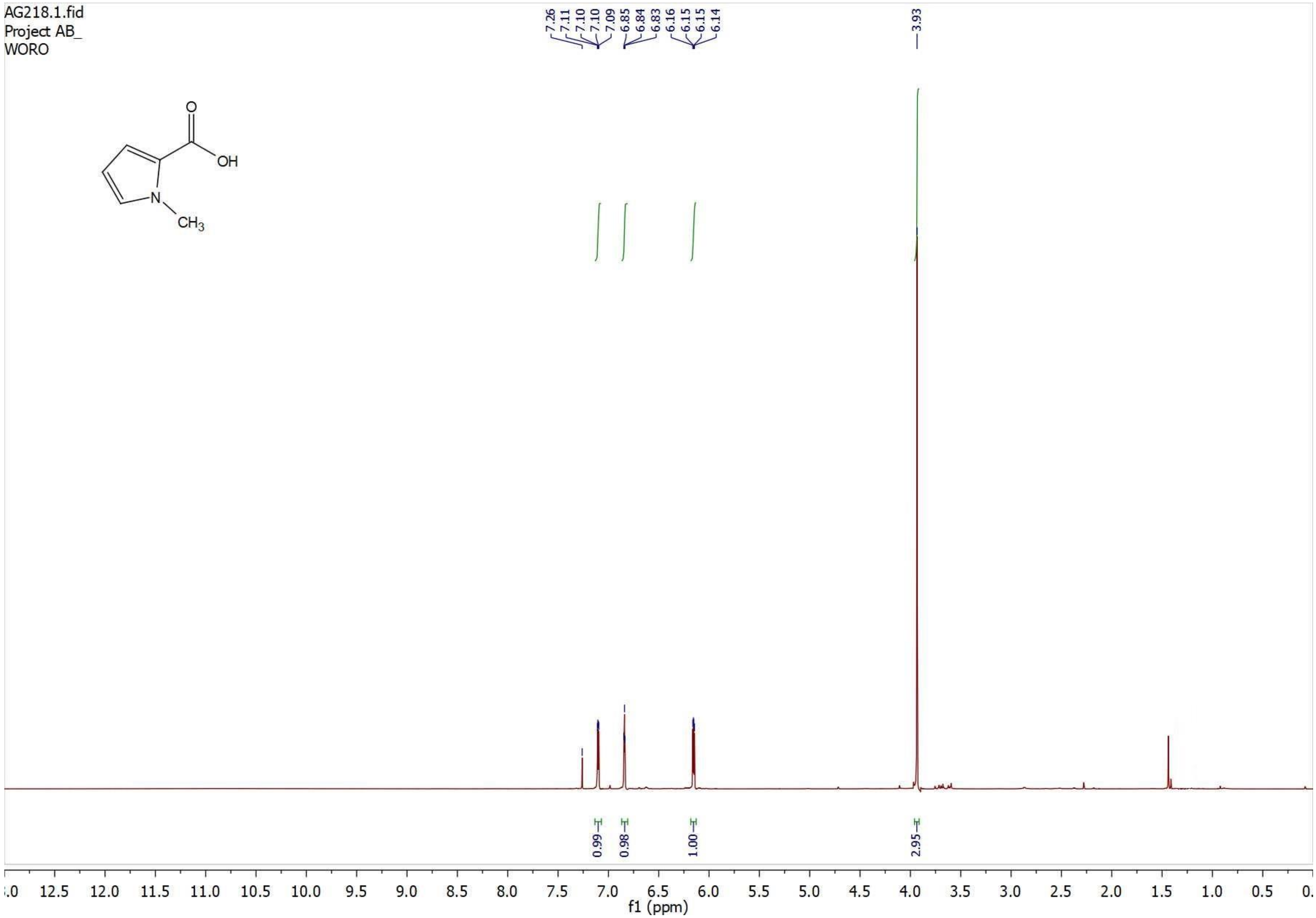
AG218.1.fid
Project AB_
WORO
7.26
7.11
7.10
7.10
7.09
6.85
6.84
6.83
6.16
6.15
6.15
6.14
3.93
O
OH
N
CH3
0.99
0.98
1.00
2.95
12.5
12.0
11.5
11.0
10.5
10.0
9.5
9.0
8.5
8.0
7.5
7.0
6.5
6.0
5.5
5.0
4.5
4.0
3.5
3.0
2.5
2.0
1.5
1.0
0.5
f1 (ppm)

Compound 4i

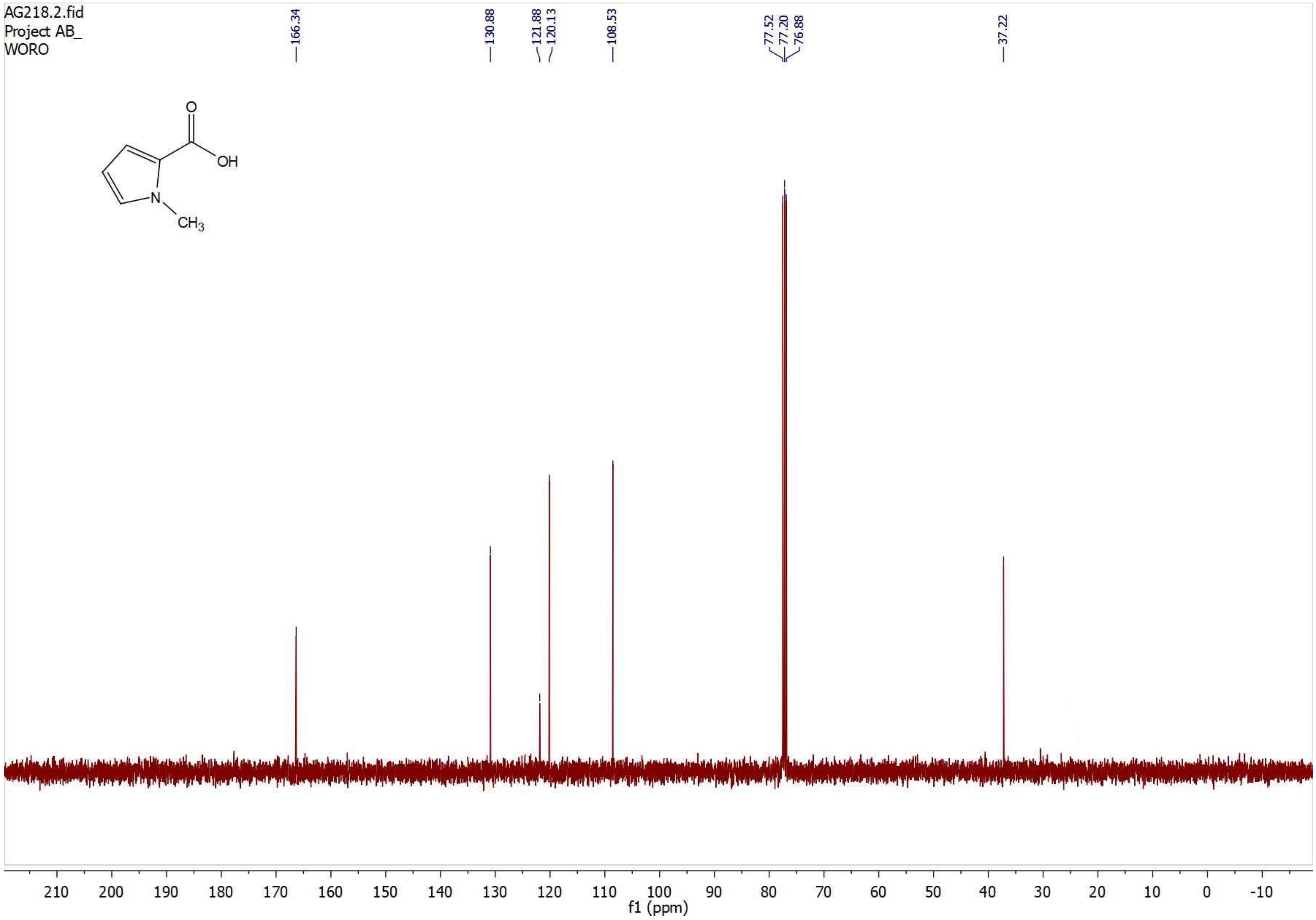
AG218.2.fid
Project AB_
WORO
166.34
130.88
121.88
120.13
108.53
77.52
77.20
76.88
37.22
O
OH
N
CH3
210
200
190
180
170
160
150
140
130
120
110
100
90
80
70
60
50
40
30
20
10
0
-10
f1 (ppm)

Compound 4j

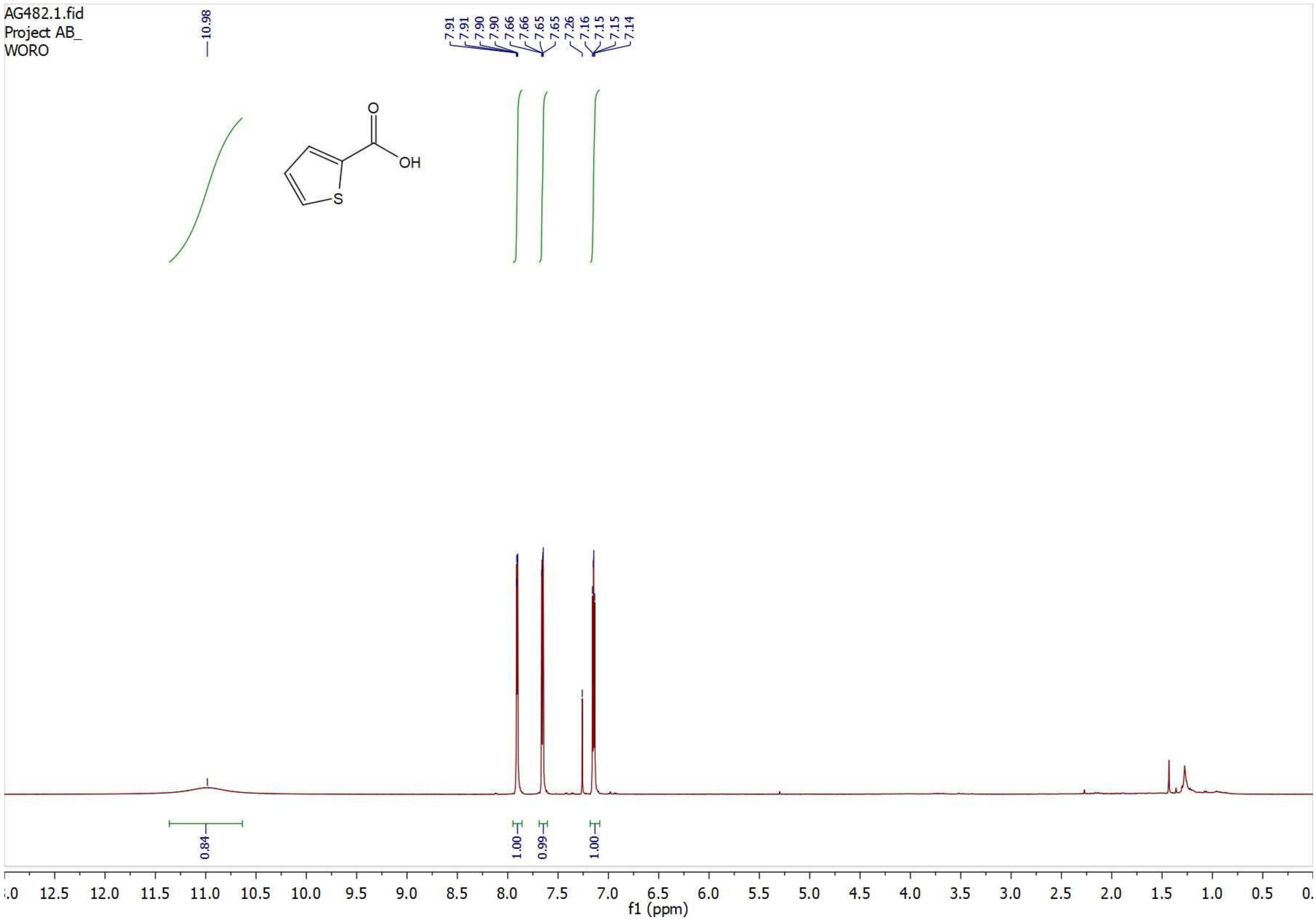
AG482.1.fid
Project AB_
WORO
10.98
7.91
7.91
7.90
7.90
7.66
7.66
7.65
7.65
7.26
7.16
7.15
7.15
7.14
OH
S
O
0.84
1.00
0.99
1.00
f1 (ppm)

Compound 4j

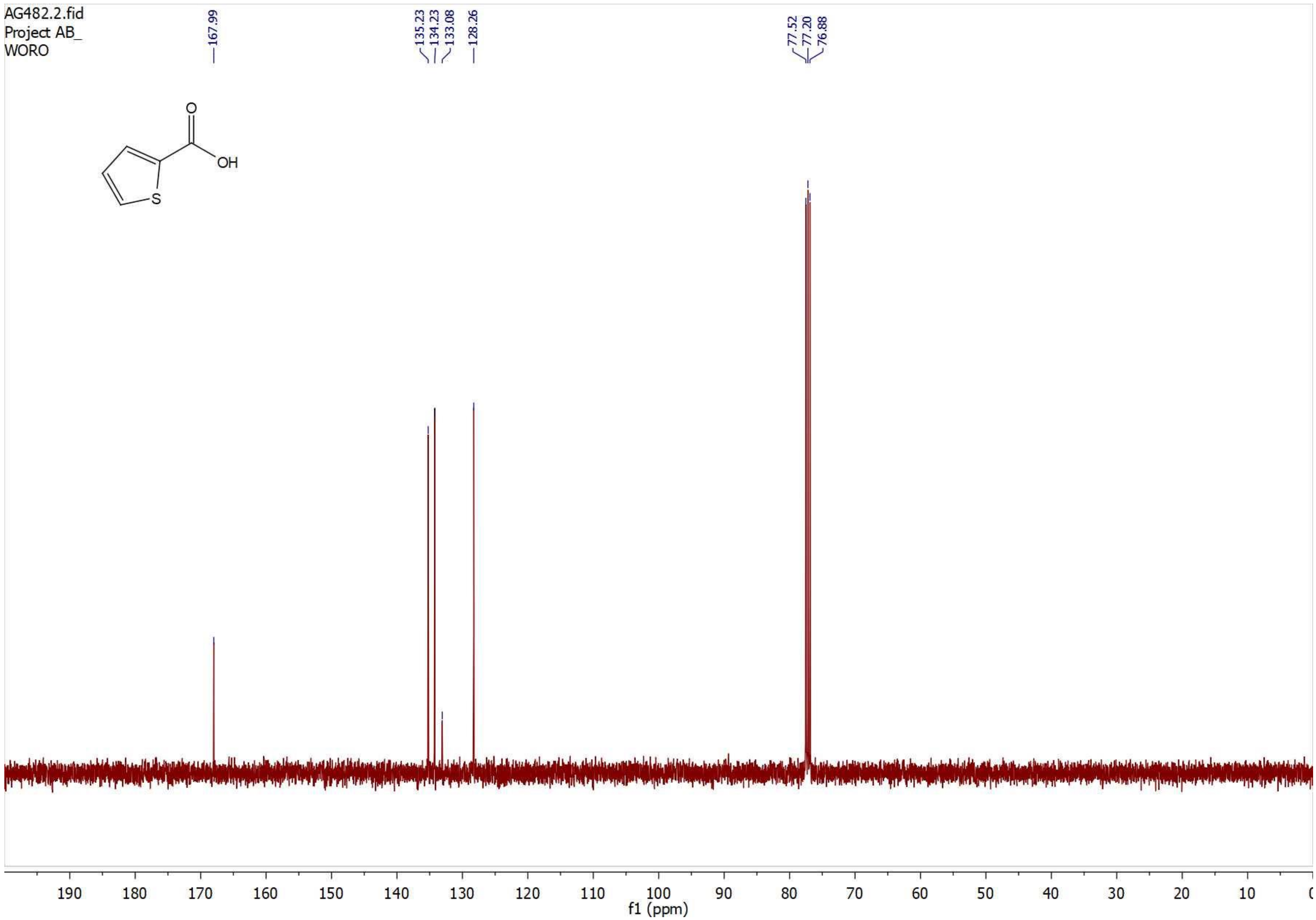

AG482.2.fid
Project AB_
WORO
167.99
135.23
134.23
133.08
128.26
77.52
77.20
76.88
O
OH
S
190
180
170
160
150
140
130
120
110
100
90
80
70
60
50
40
30
20
10
f1 (ppm)

Compound 4k

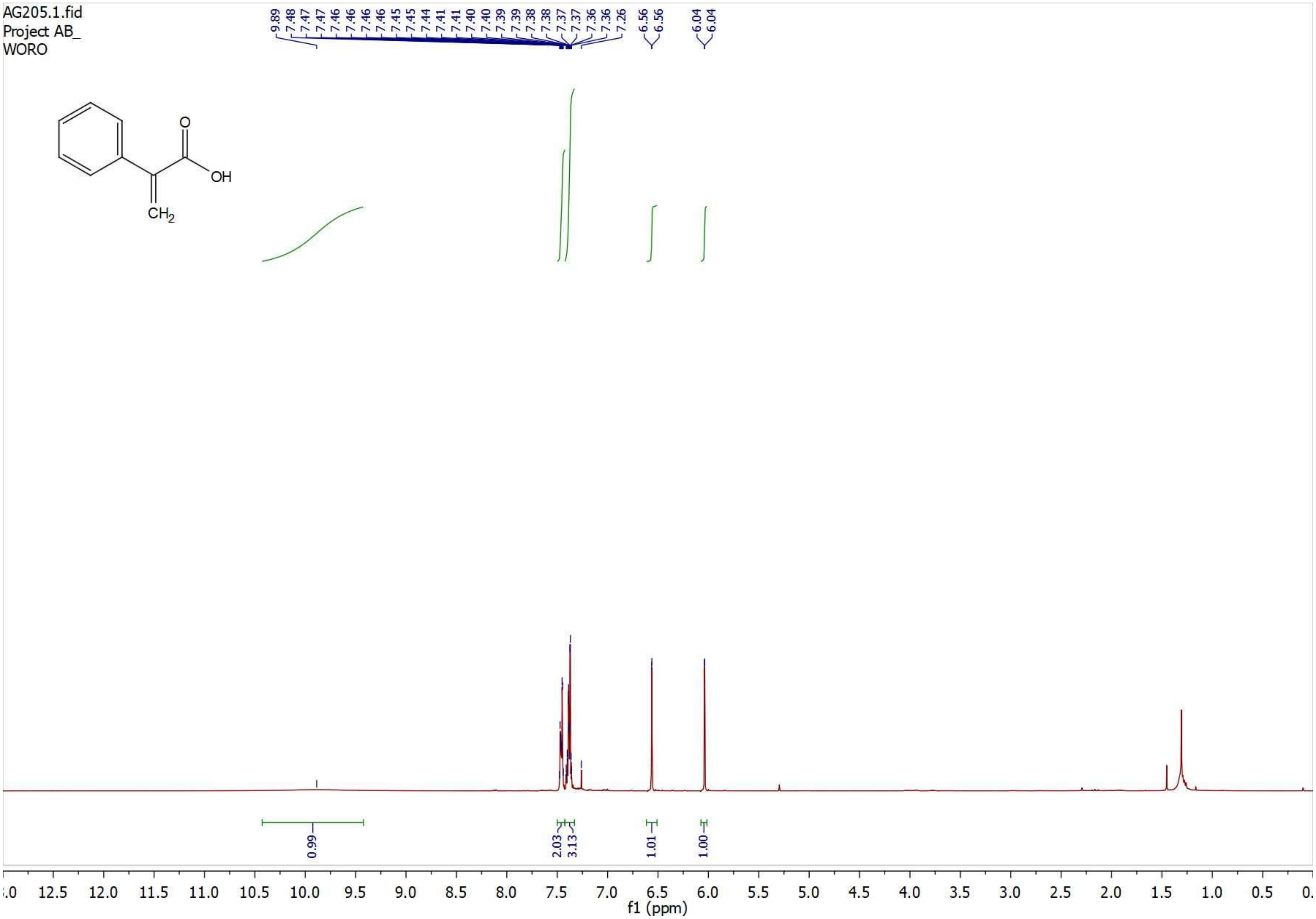

AG205.1.fid
Project AB_
WORO
9.89 7.48 7.47 7.47 7.46 7.46 7.46 7.45 7.45 7.44 7.41 7.40 7.40 7.39 7.39 7.38 7.38 7.37 7.37 7.36 7.36 7.26 6.56 6.56 6.04 6.04
0.99 2.03 3.13 1.01 1.00
12.5 12.0 11.5 11.0 10.5 10.0 9.5 9.0 8.5 8.0 7.5 7.0 6.5 6.0 5.5 5.0 4.5 4.0 3.5 3.0 2.5 2.0 1.5 1.0 0.5
f1 (ppm)

Compound 4k

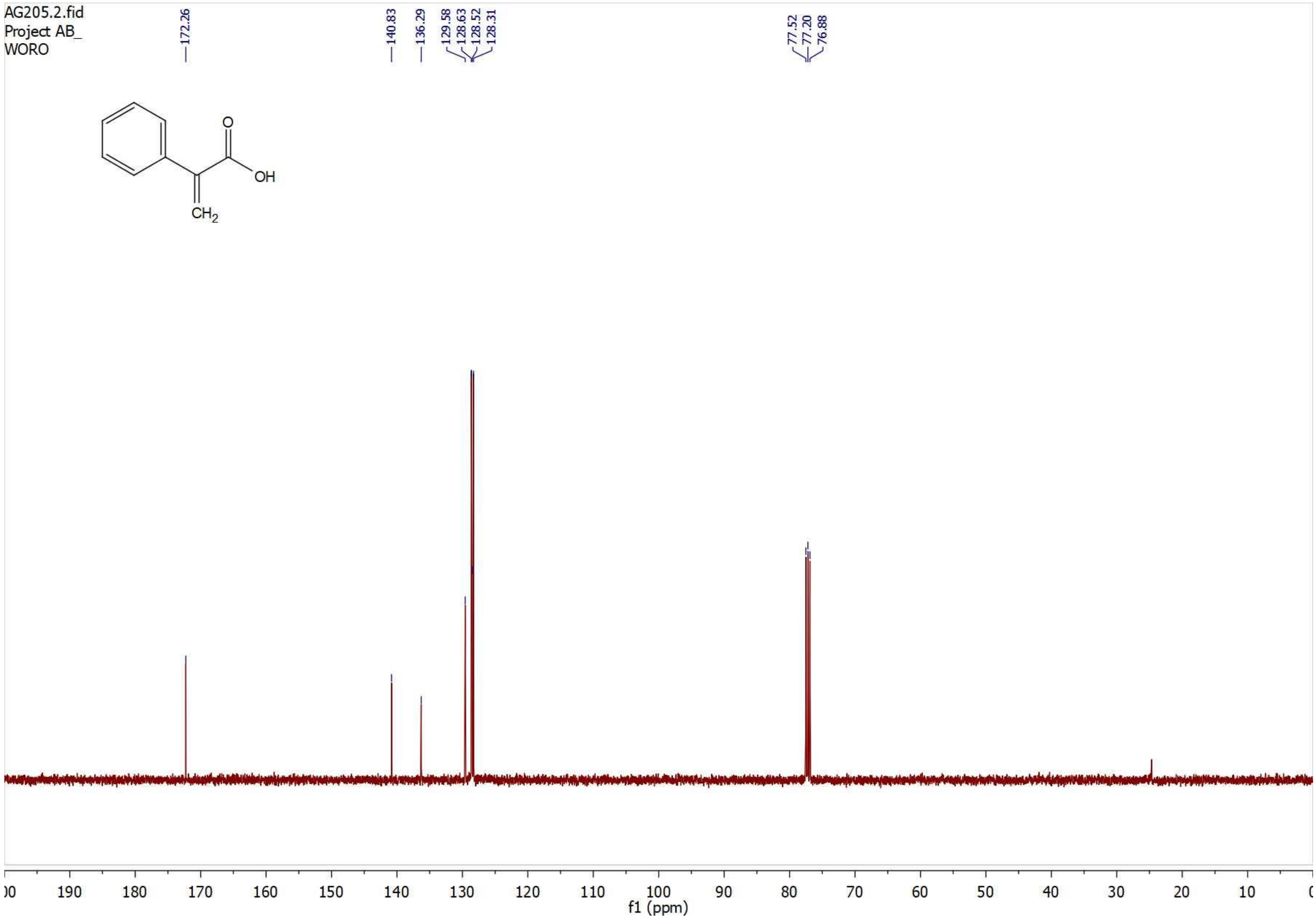
AG205.2.fid
Project AB_
WORO
172.26
140.83
136.29
129.58
128.63
128.52
128.31
77.52
77.20
76.88
O
OH
CH_2
190 180 170 160 150 140 130 120 110 100 90 80 70 60 50 40 30 20 10
f1 (ppm)

Compound 4l

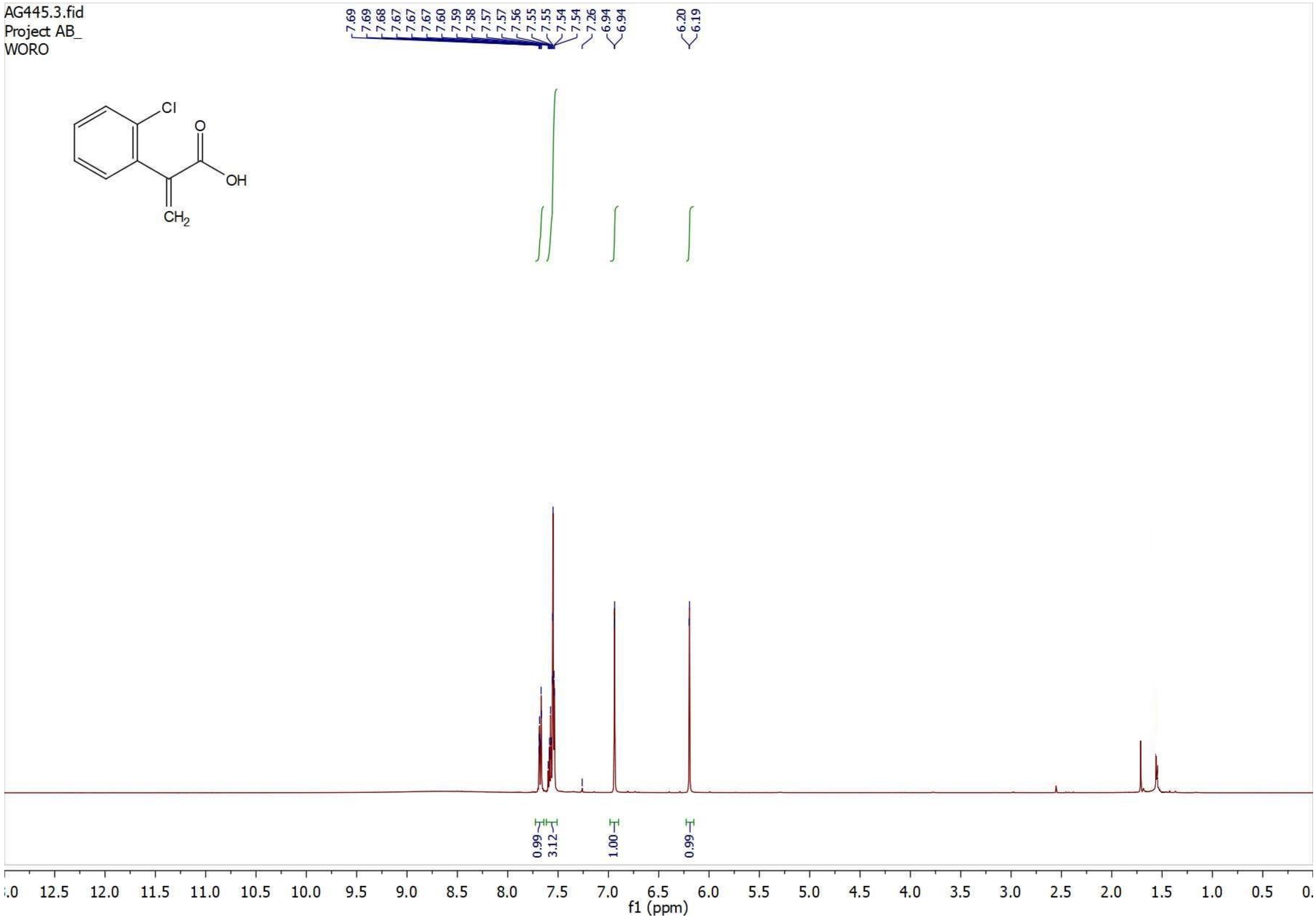
AG445.3.fid
Project AB_
WORO
Cl
O
OH
CH2
7.69
7.69
7.68
7.67
7.67
7.67
7.60
7.59
7.58
7.57
7.57
7.56
7.55
7.55
7.54
7.54
7.26
6.94
6.94
6.20
6.19
0.99
3.12
1.00
0.99
f1 (ppm)

Compound 4l

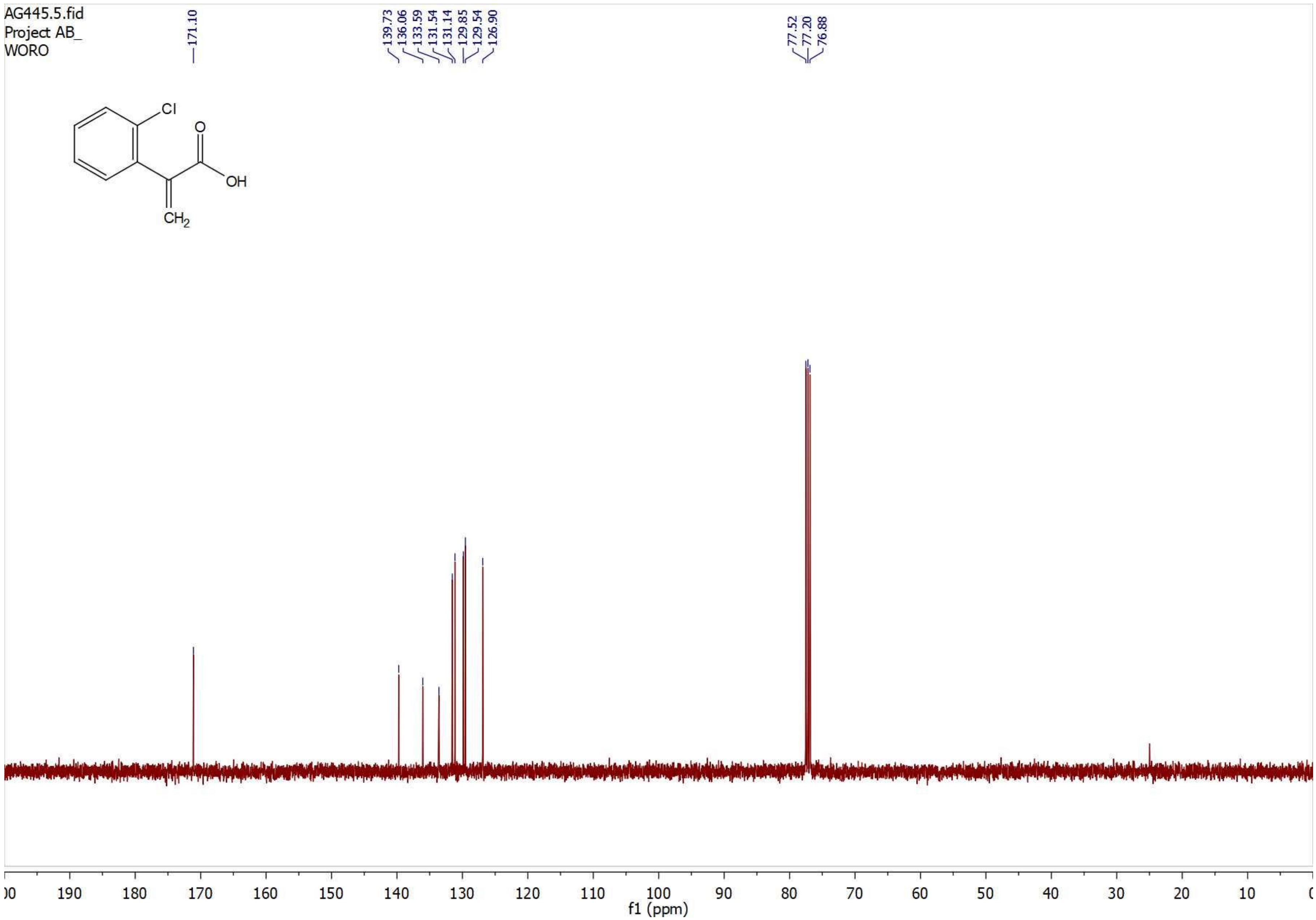

AG445.5.fid
Project AB_
WORO
171.10
139.73
136.06
133.59
131.54
131.14
129.85
129.54
126.90
77.52
77.20
76.88
Cl
O
OH
CH2
190
180
170
160
150
140
130
120
110
100
90
80
70
60
50
40
30
20
10
f1 (ppm)

Compound 4m

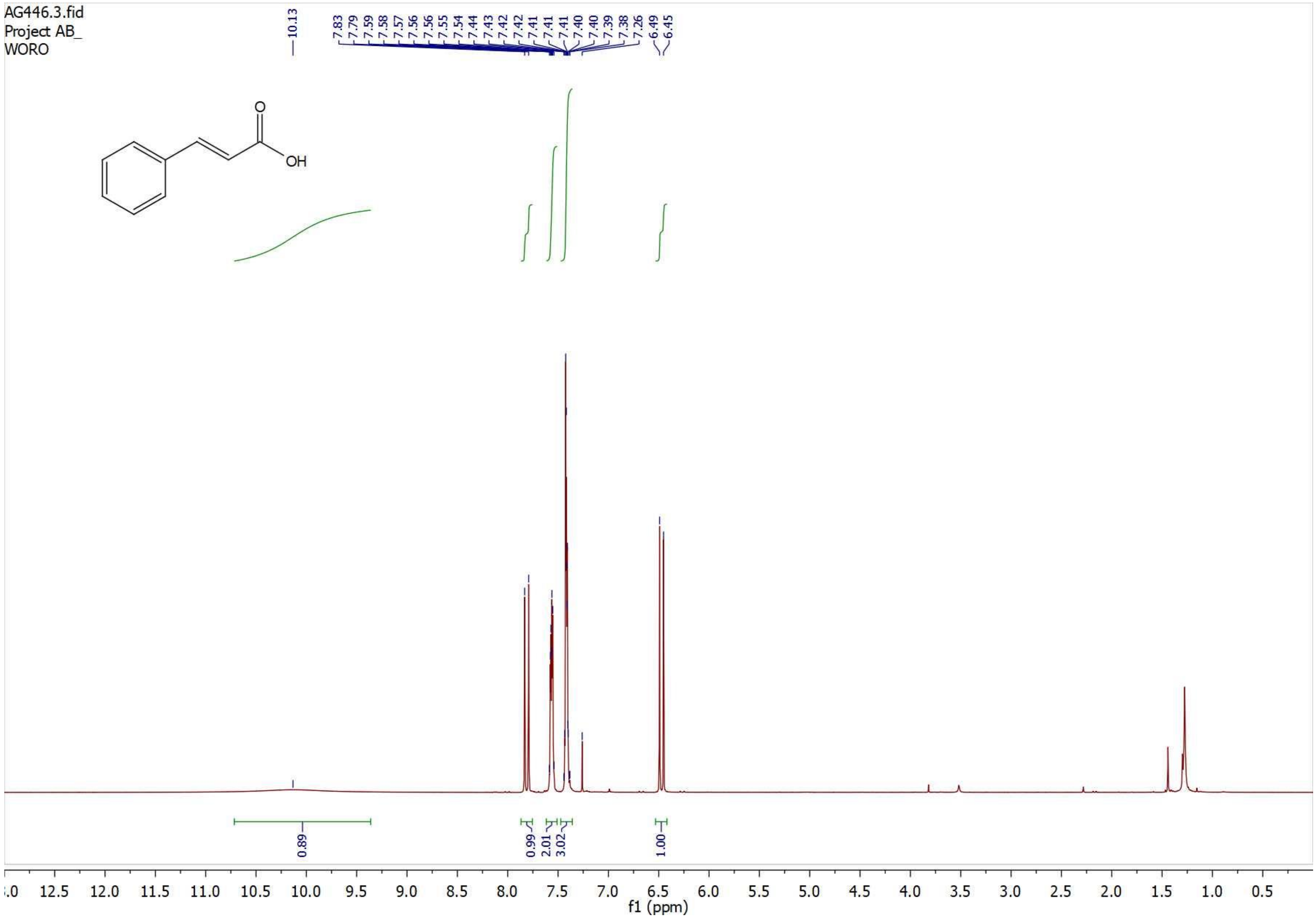
AG446.3.fid
Project AB_
WORO
10.13
7.83
7.79
7.59
7.58
7.57
7.56
7.56
7.55
7.54
7.44
7.43
7.42
7.42
7.41
7.41
7.41
7.40
7.39
7.38
7.26
6.49
6.45
OH
O
0.89
0.99
2.01
3.02
1.00
f1 (ppm)

Compound 4m

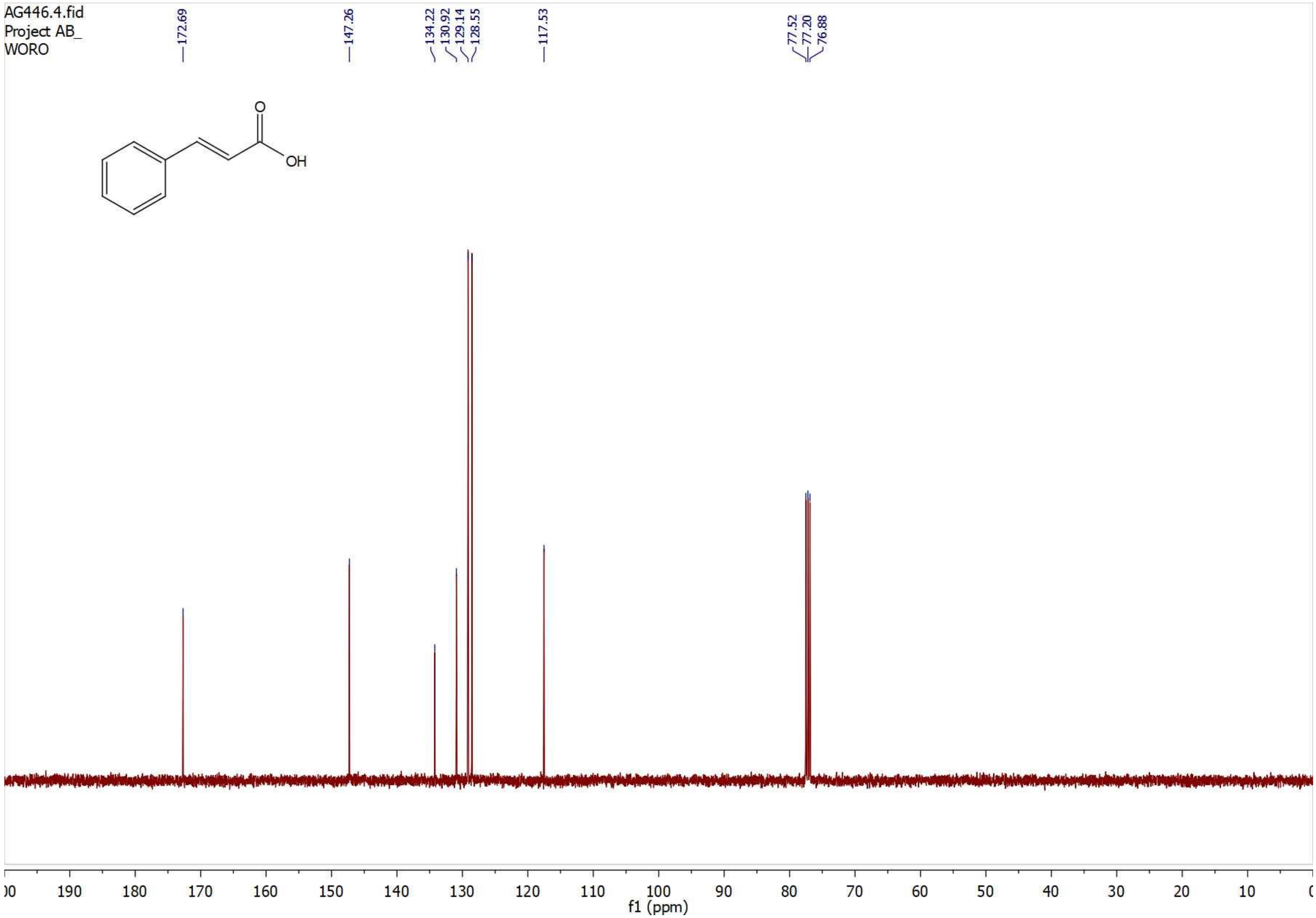
AG446.4.fid
Project AB_
WORO
172.69
147.26
134.22
130.92
129.14
128.55
117.53
77.52
77.20
76.88
O
OH
190
180
170
160
150
140
130
120
110
100
90
80
70
60
50
40
30
20
10
f1 (ppm)

Compound 4n

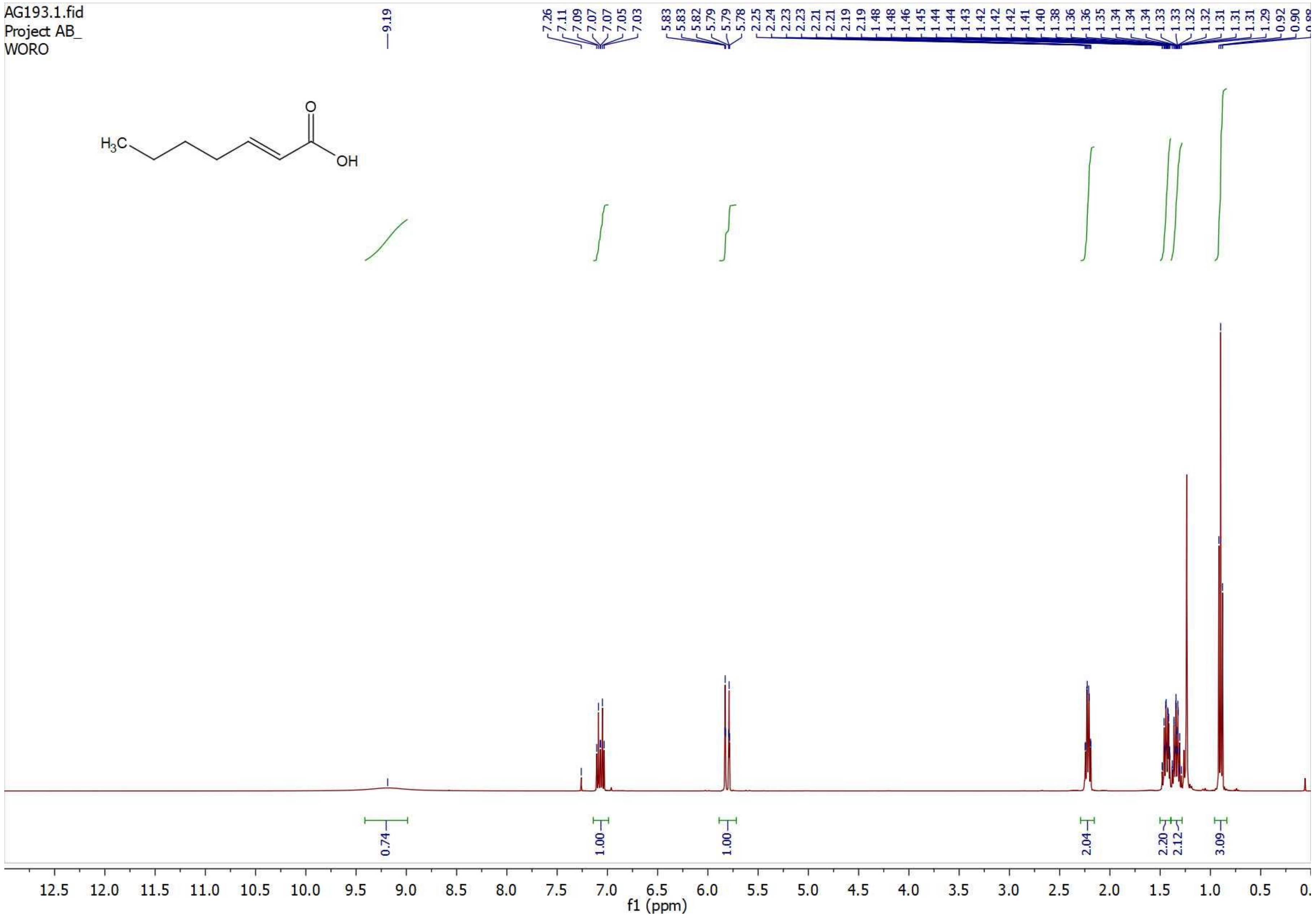

Compound 4n

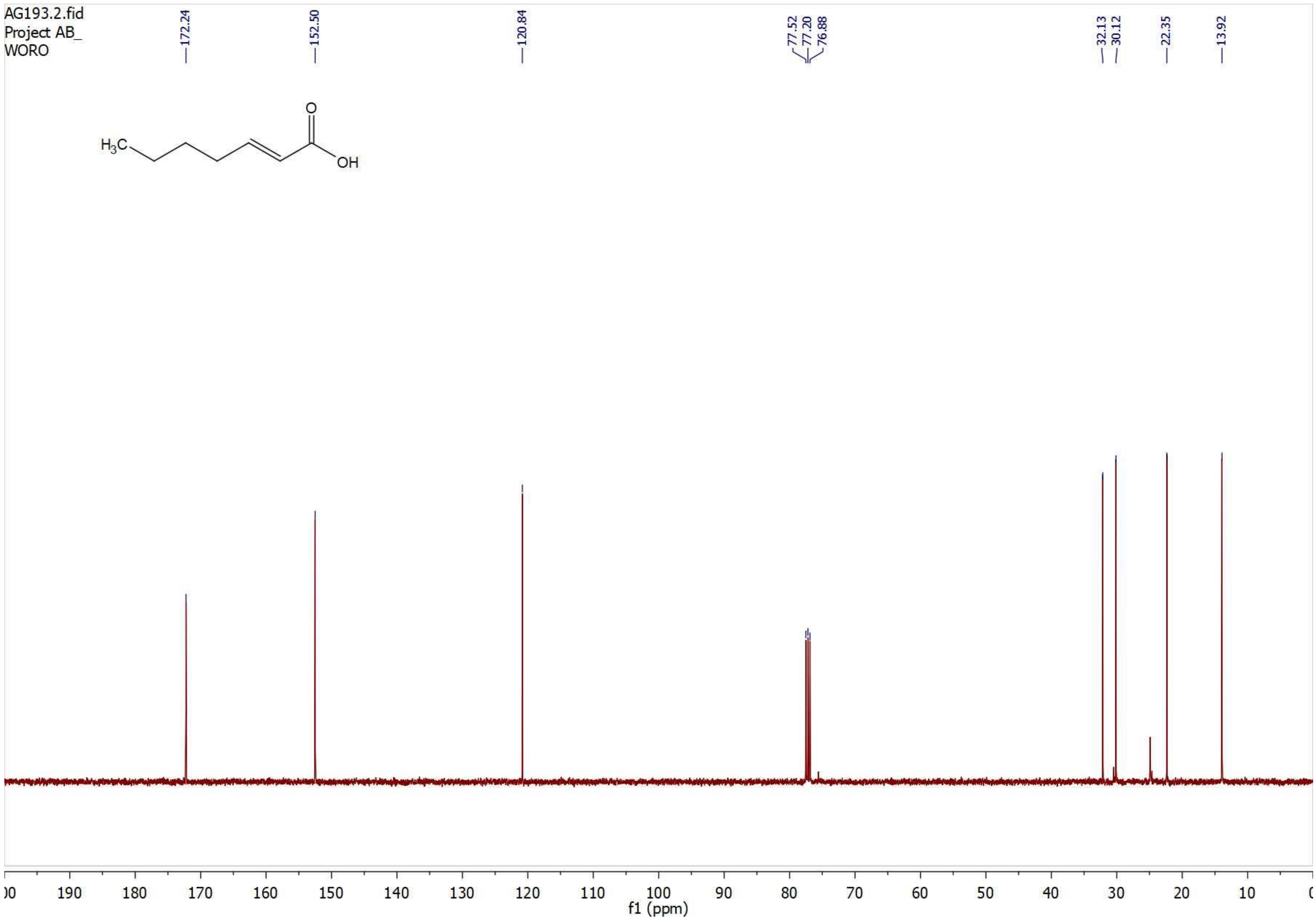

AG193.2.fid
Project AB_
WORO
172.24
152.50
120.84
77.52
77.20
76.88
32.13
30.12
22.35
13.92
H3C
O
OH
f1 (ppm)

Compound 4o

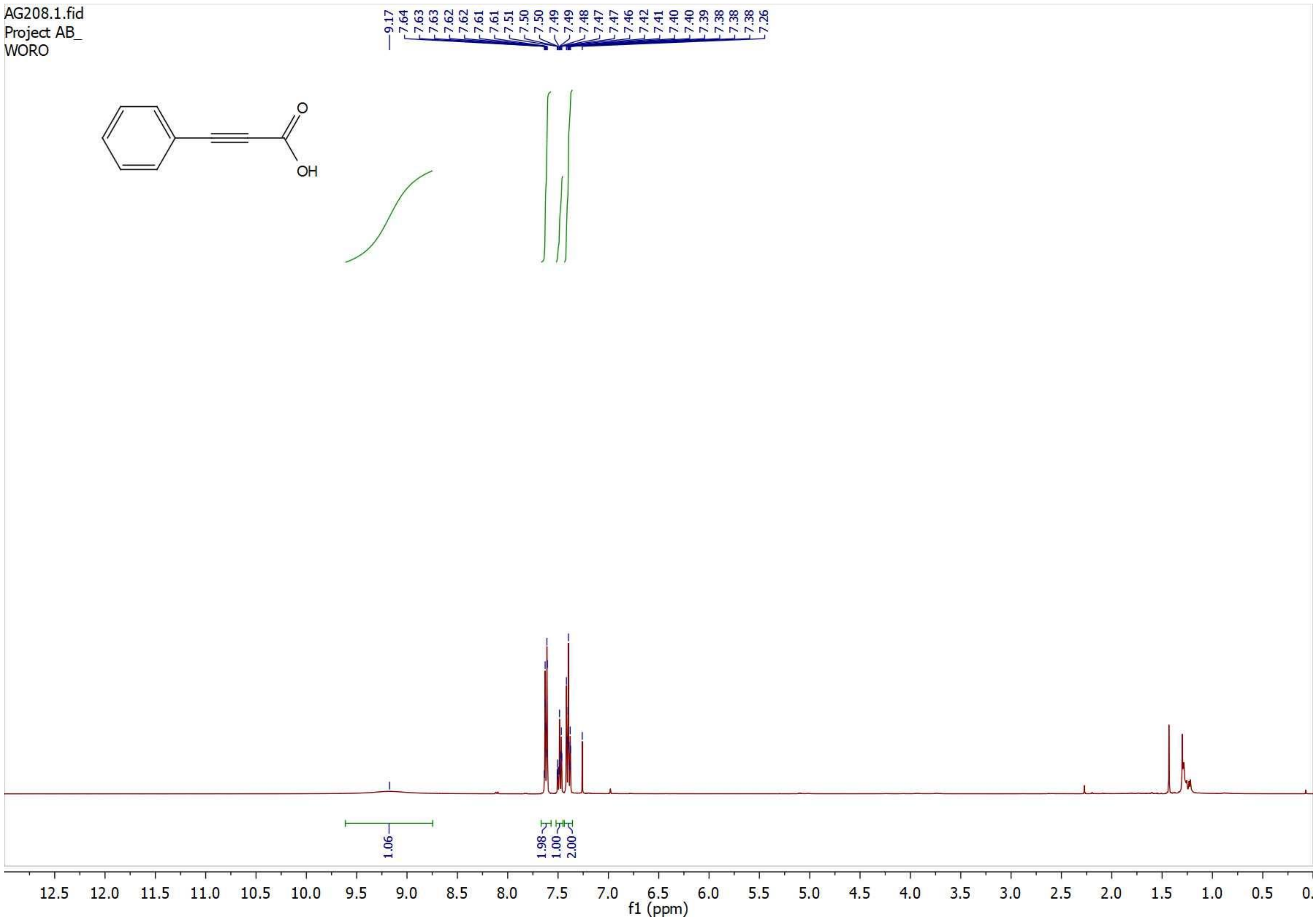

AG208.1.fid
Project AB_
WORO
OH
O
9.17
7.64
7.63
7.63
7.62
7.62
7.61
7.61
7.51
7.50
7.50
7.49
7.49
7.48
7.47
7.47
7.46
7.42
7.41
7.40
7.40
7.39
7.38
7.38
7.38
7.26
1.06
1.98
1.00
2.00
12.5 12.0 11.5 11.0 10.5 10.0 9.5 9.0 8.5 8.0 7.5 7.0 6.5 6.0 5.5 5.0 4.5 4.0 3.5 3.0 2.5 2.0 1.5 1.0 0.5 0.
f1 (ppm)

Compound 4o

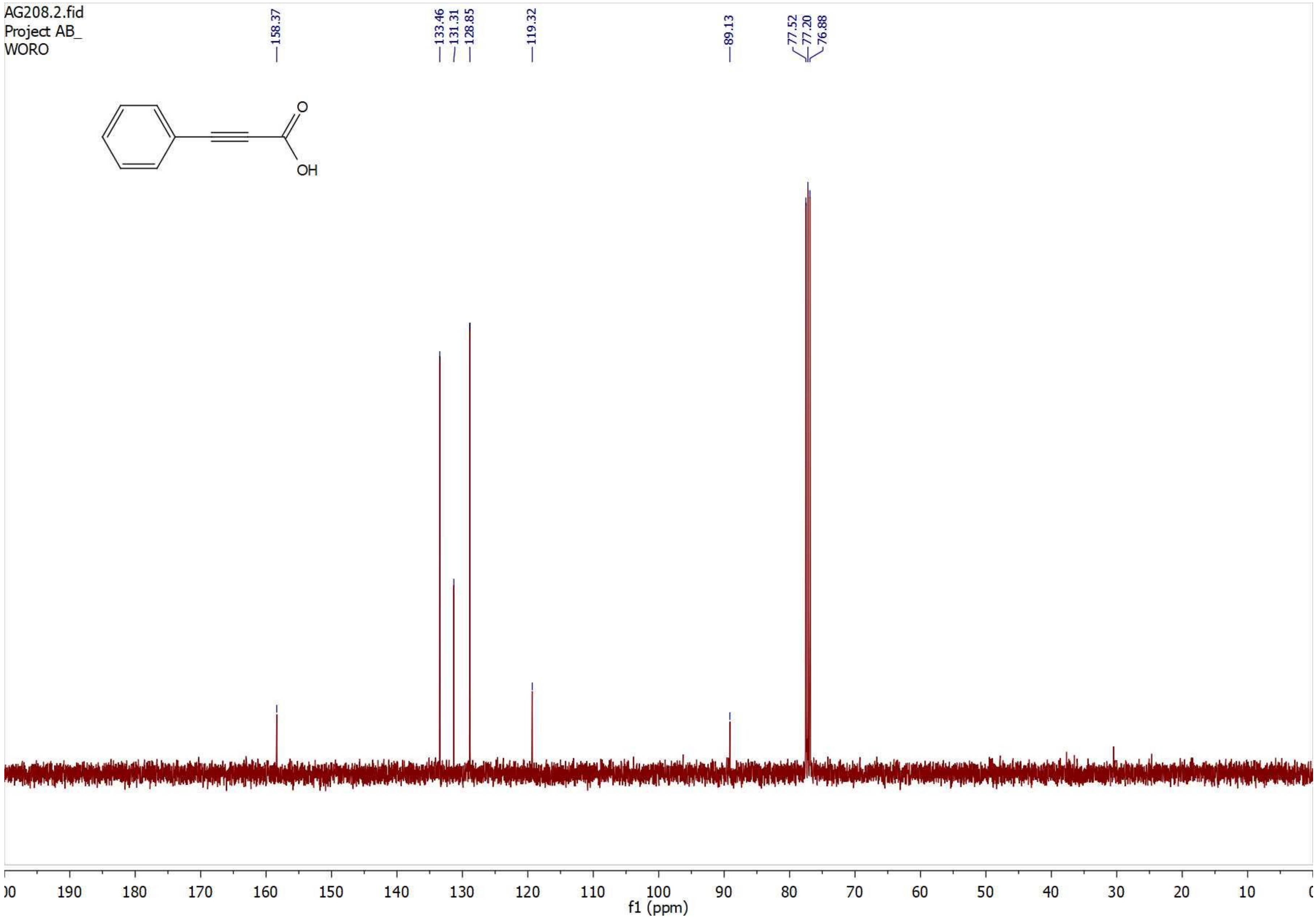

AG208.2.fid
Project AB_
WORO
158.37
133.46
131.31
128.85
119.32
89.13
77.52
77.20
76.88
OH
O
190
180
170
160
150
140
130
120
110
100
90
80
70
60
50
40
30
20
10
f1 (ppm)

Compound 4p

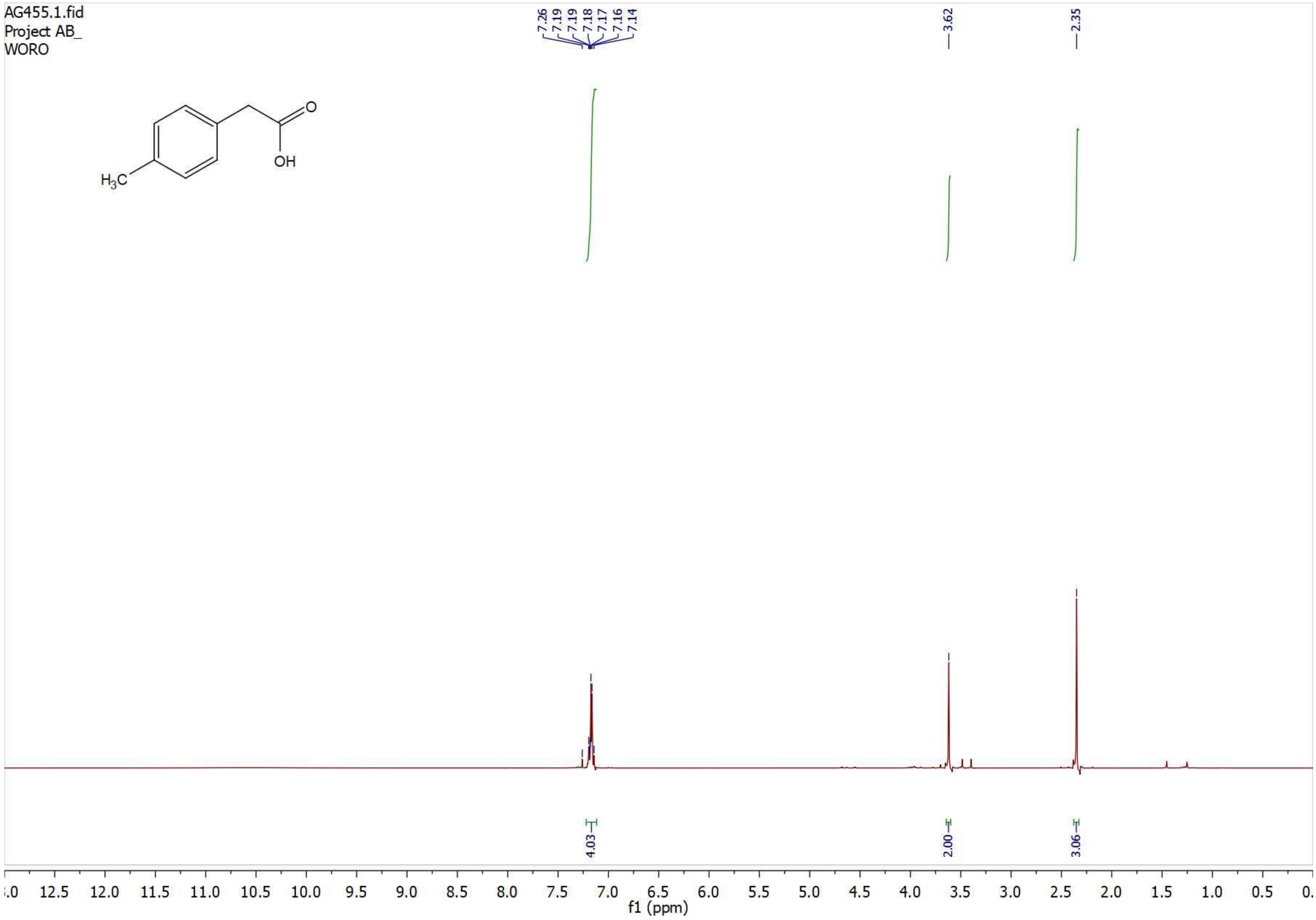
AG455.1.fid
Project AB_
WORO
7.26
7.19
7.19
7.18
7.17
7.16
7.14
3.62
2.35
H3C
O
OH
4.03
2.00
3.06
13.0 12.5 12.0 11.5 11.0 10.5 10.0 9.5 9.0 8.5 8.0 7.5 7.0 6.5 6.0 5.5 5.0 4.5 4.0 3.5 3.0 2.5 2.0 1.5 1.0 0.5 0.
f1 (ppm)

Compound 4p

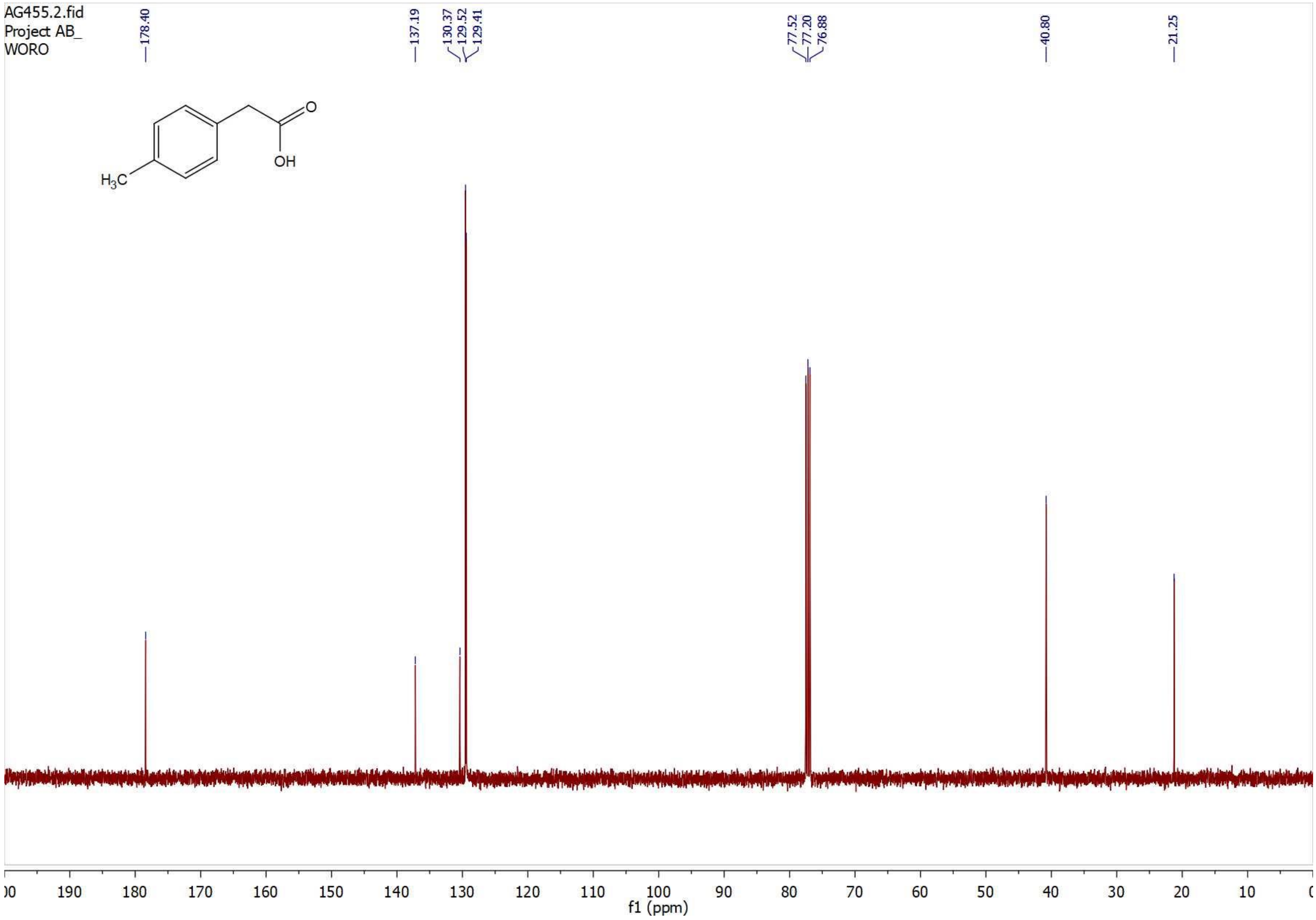

AG455.2.fid
Project AB_
WORO
178.40
137.19
130.37
129.52
129.41
77.52
77.20
76.88
40.80
21.25
H3C
OH
O
f1 (ppm)

Compound 4q

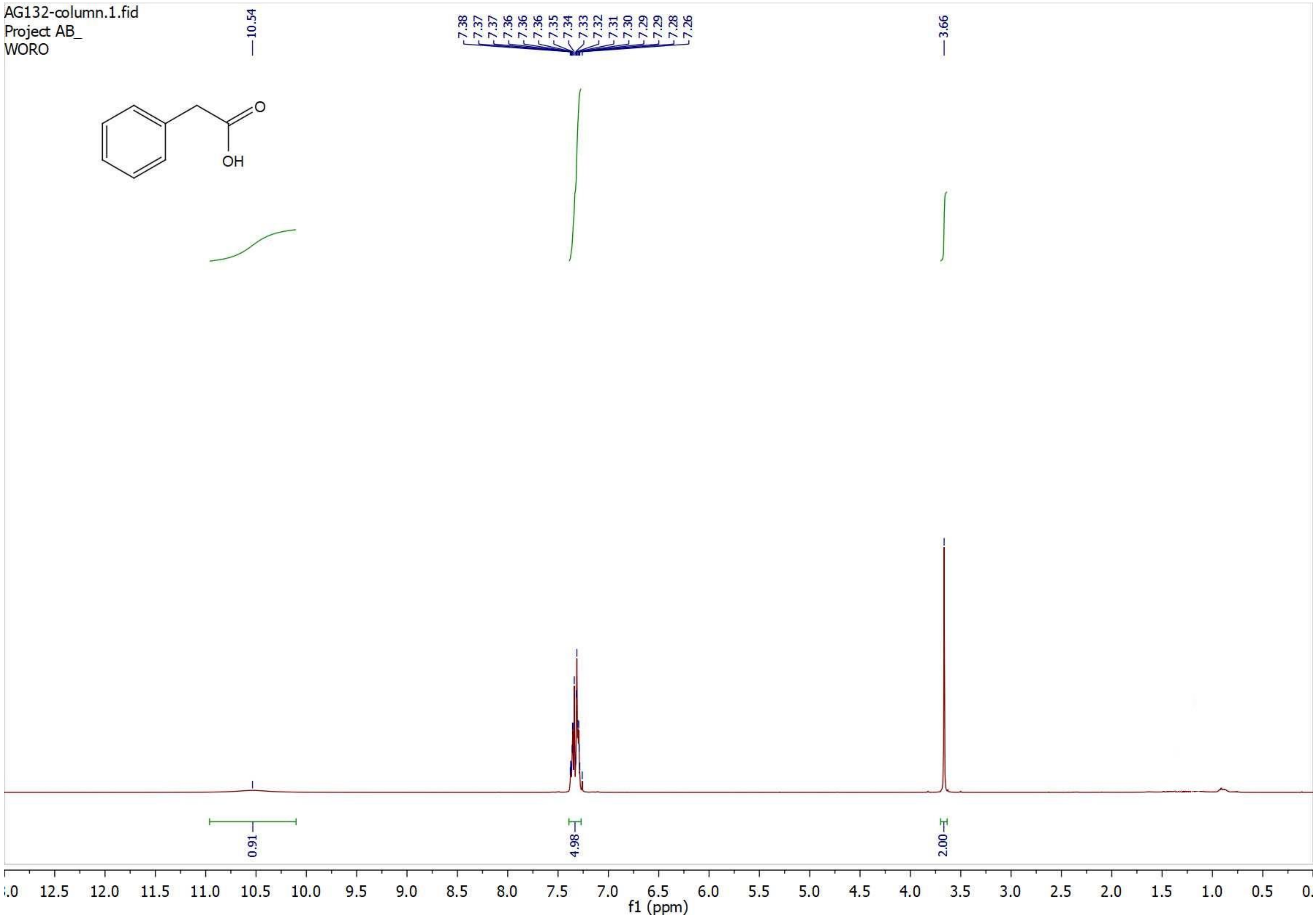
AG132-column.1.fid
Project AB_
WORO
10.54
7.38
7.37
7.37
7.36
7.36
7.35
7.34
7.33
7.32
7.31
7.30
7.29
7.29
7.28
7.26
3.66
OH
O
0.91
4.98
2.00
f1 (ppm)

Compound 4q

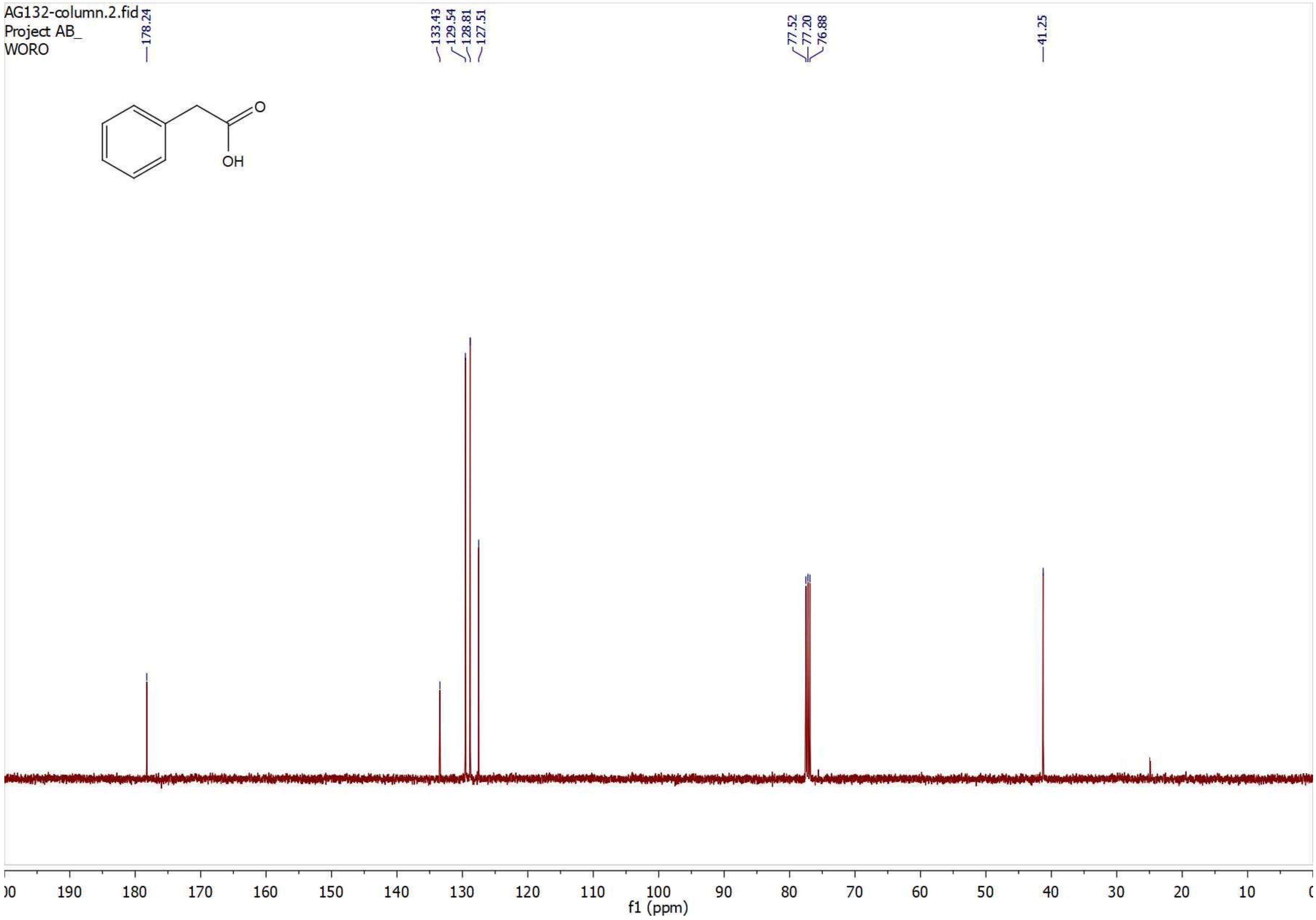
AG132-column.2.fid
Project AB_
WORO
178.24
133.43
129.54
128.81
127.51
77.52
77.20
76.88
41.25
OH
O
190
180
170
160
150
140
130
120
110
100
90
80
70
60
50
40
30
20
10
f1 (ppm)

Compound 4r, Fenoprofen

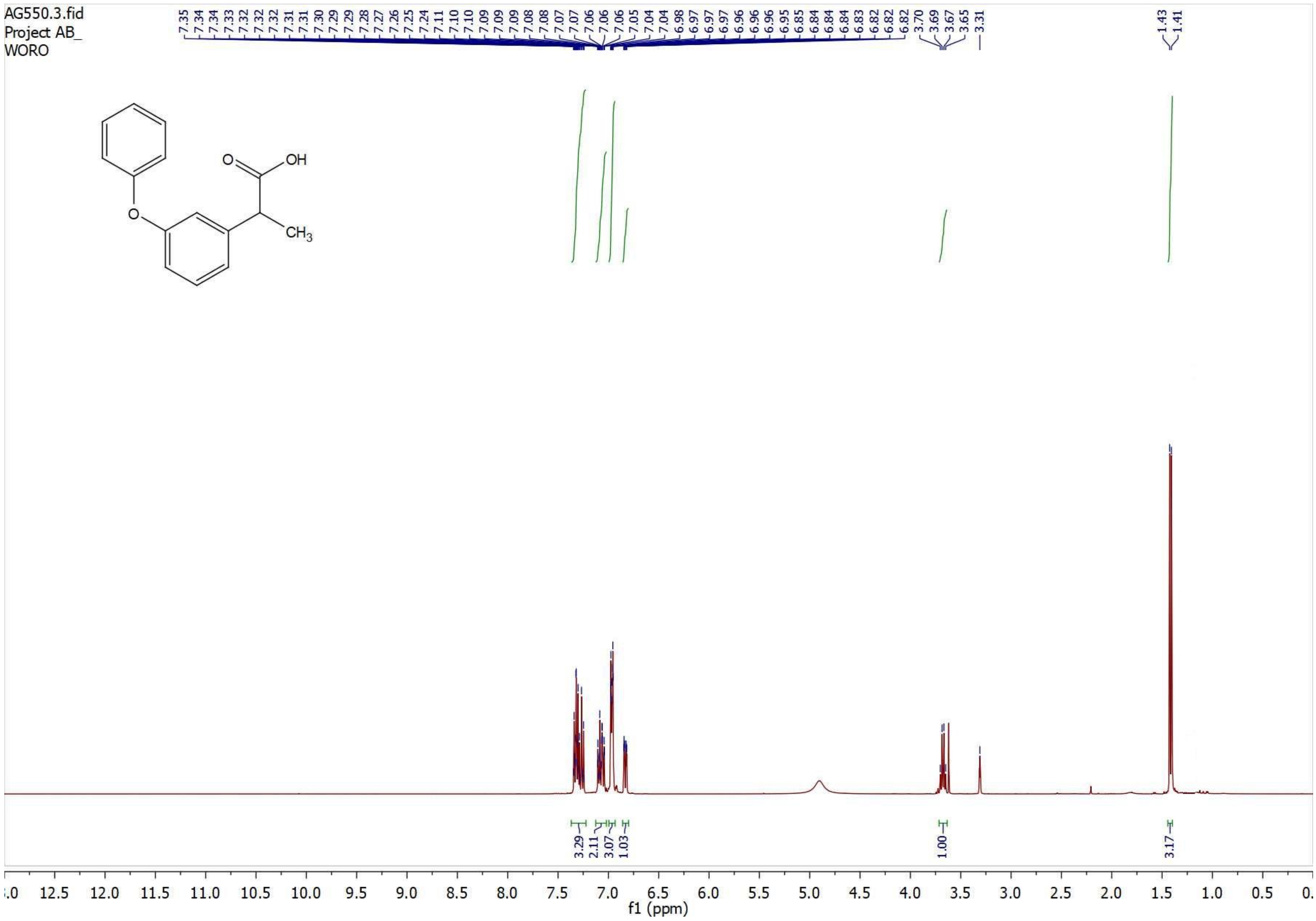

Compound 4r, Fenoprofen

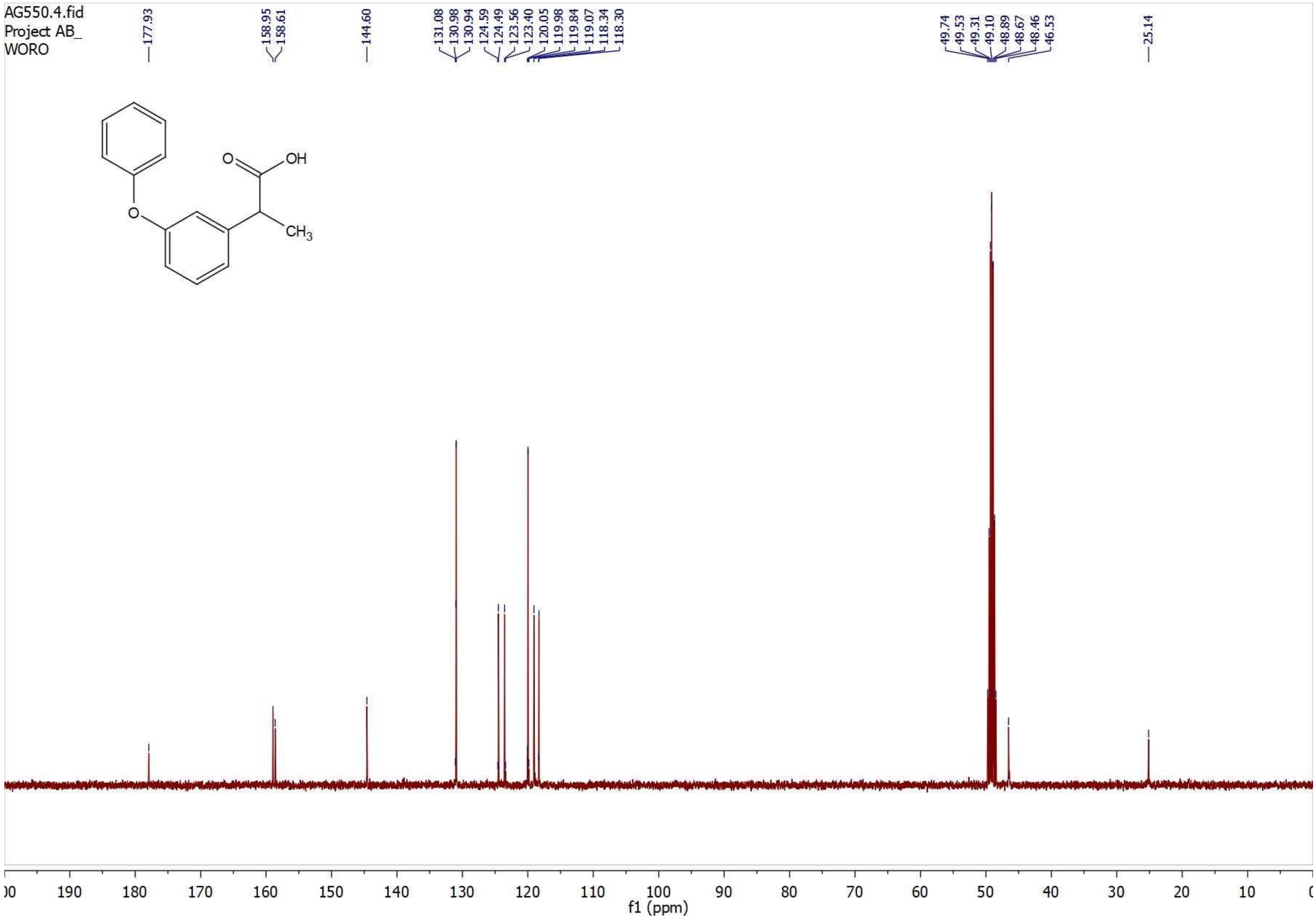

Compound 4s, Flurbiprofen

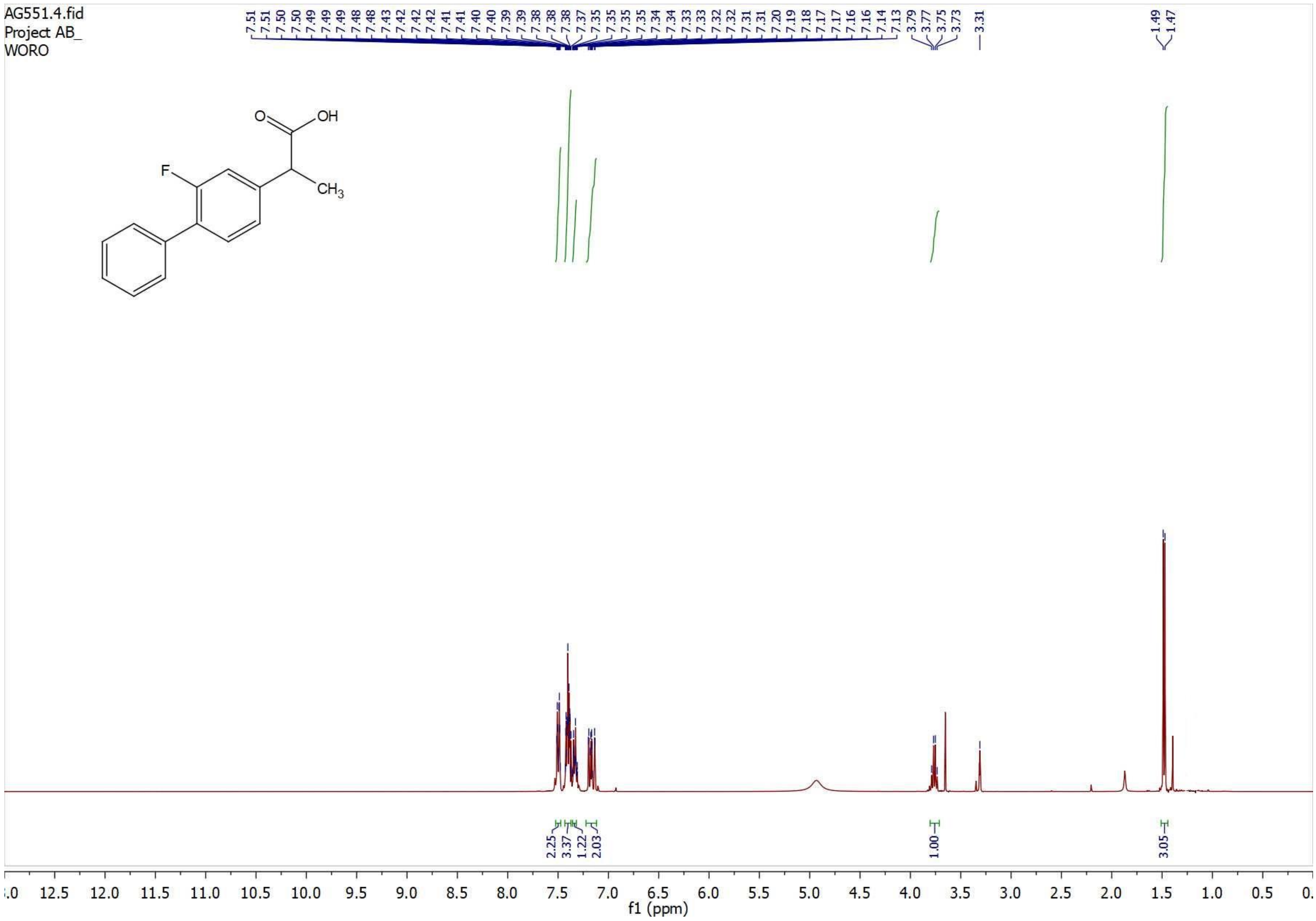

Compound 4s, Flurbiprofen

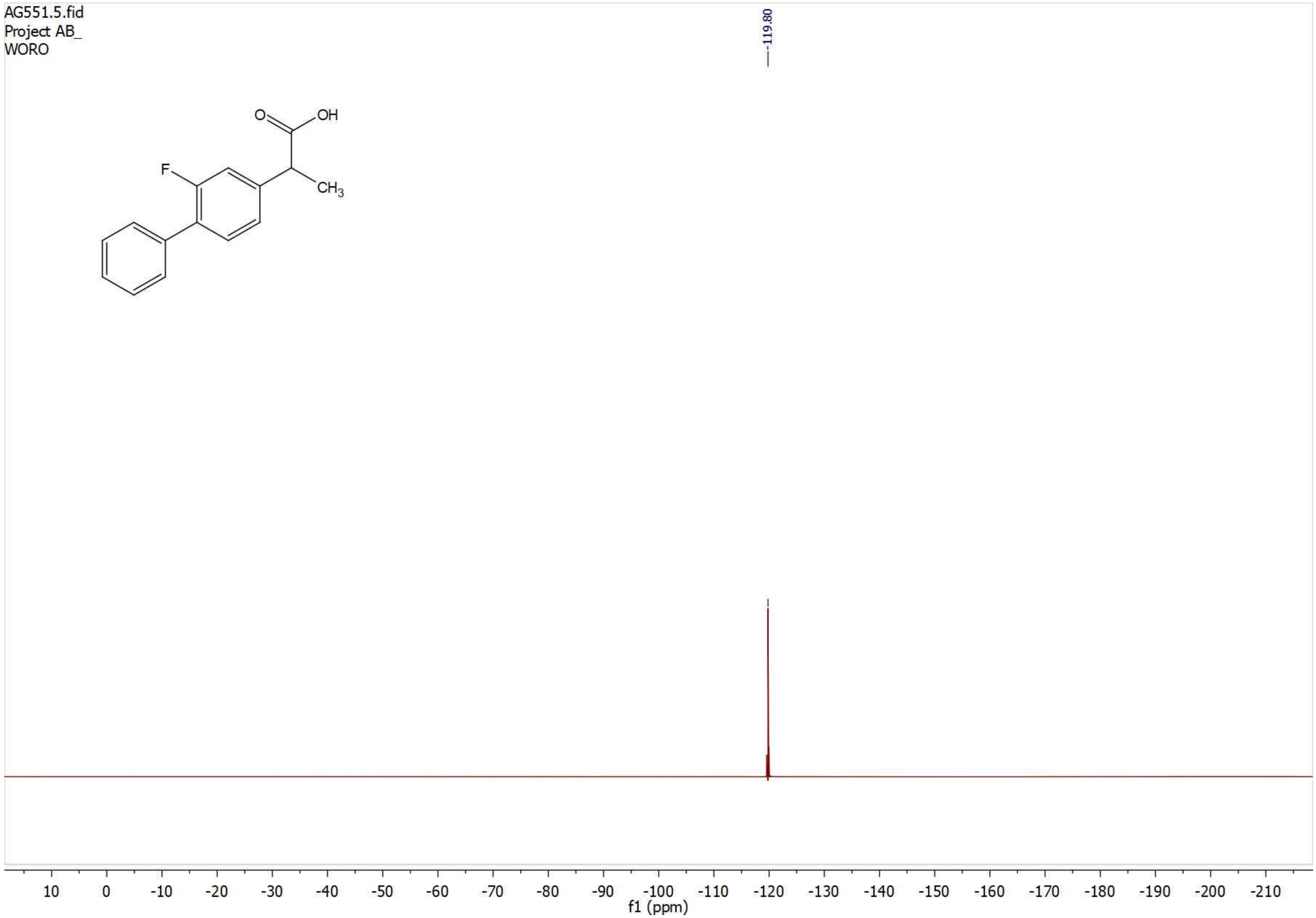

Compound 4s, Flurbiprofen

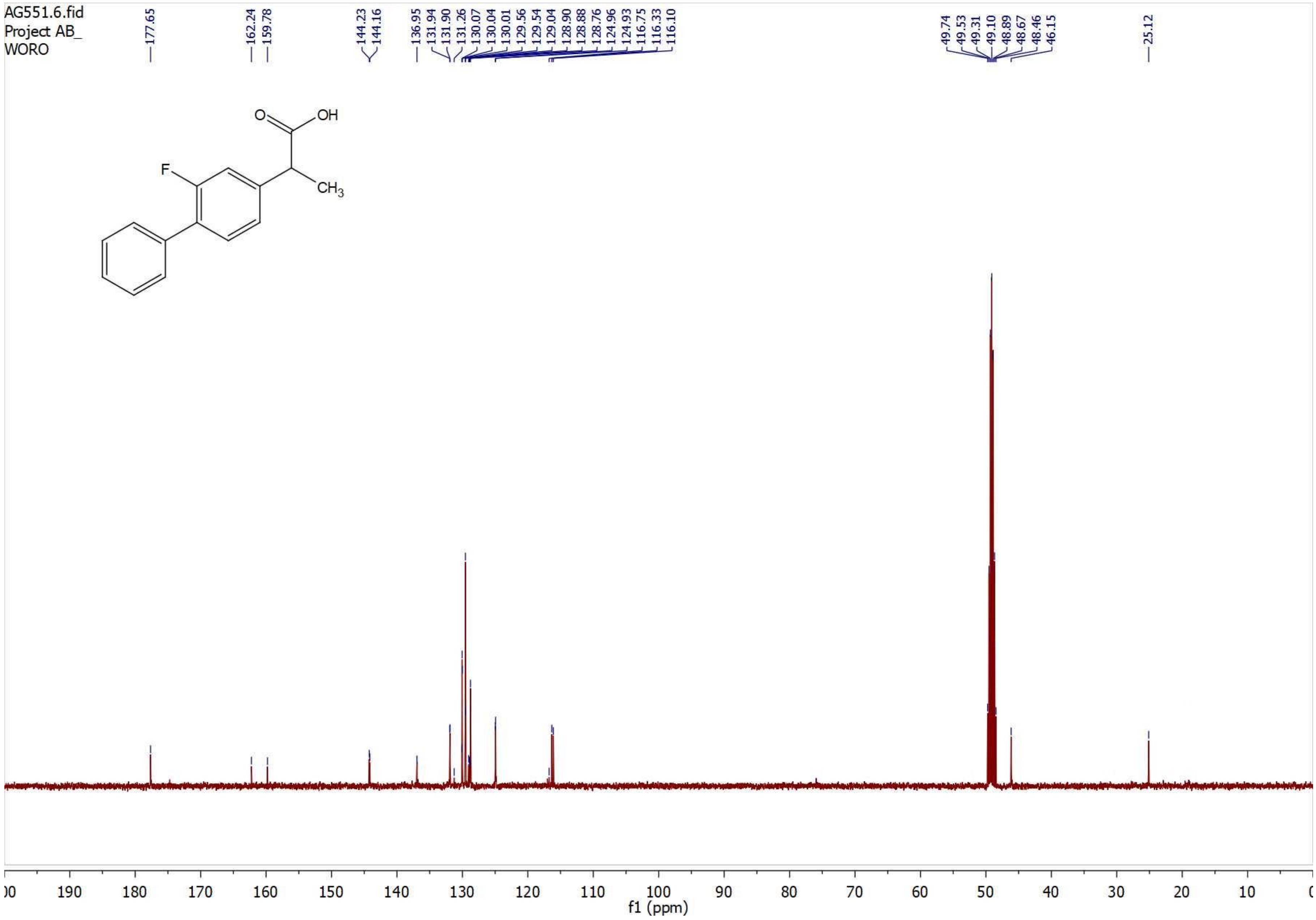

Compound 2b:6a

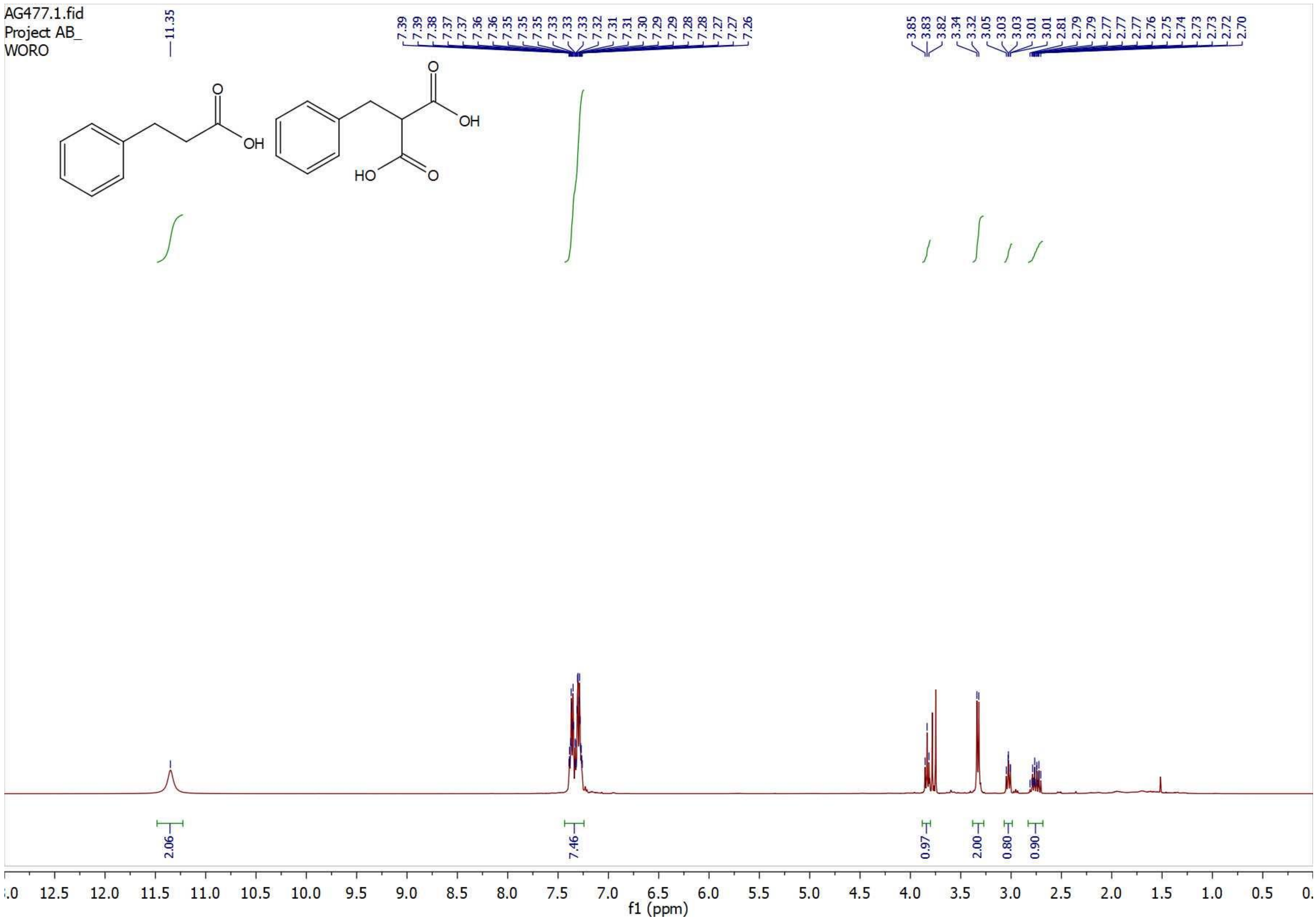

Compound 2b:6a

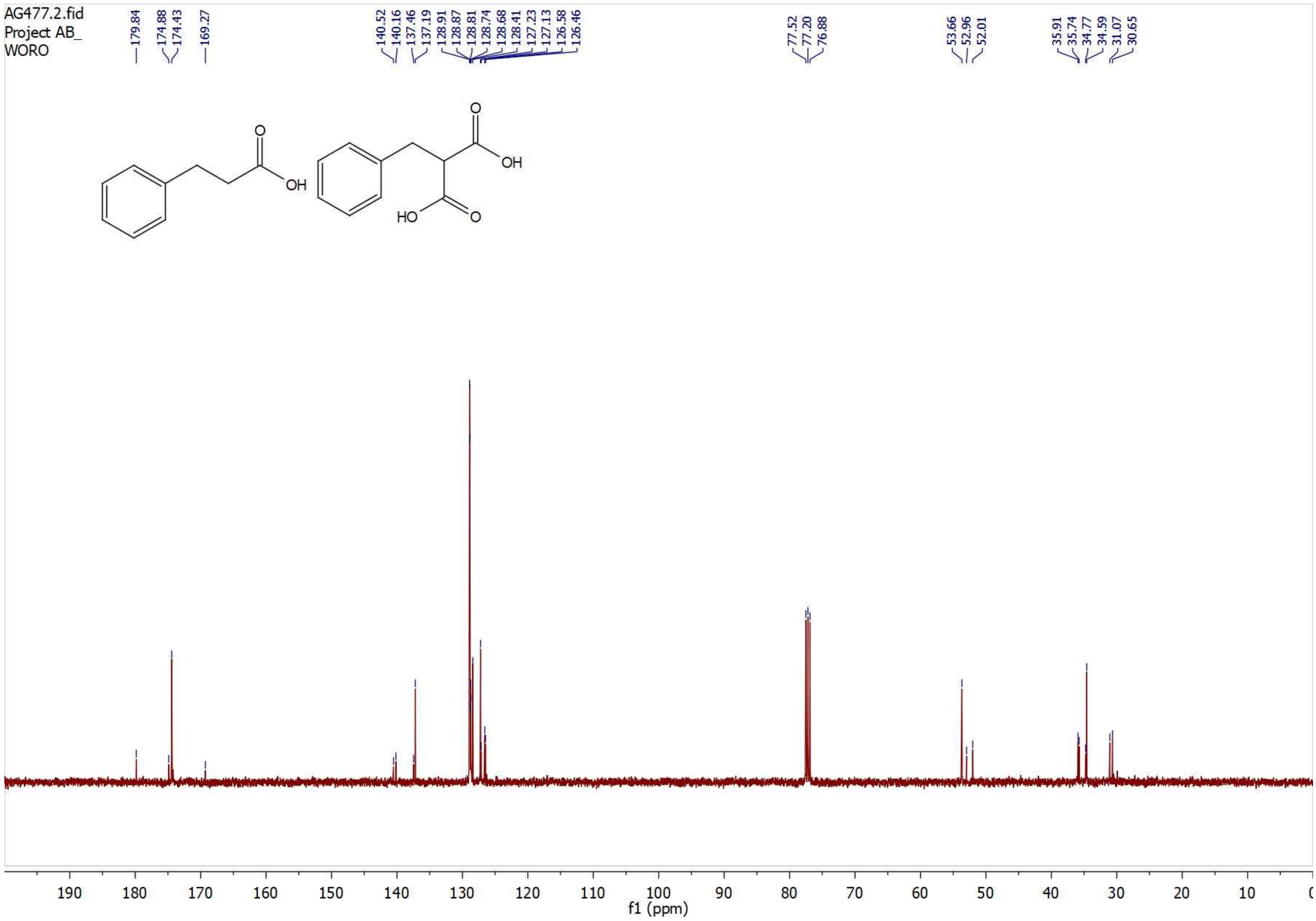

Compound 7a

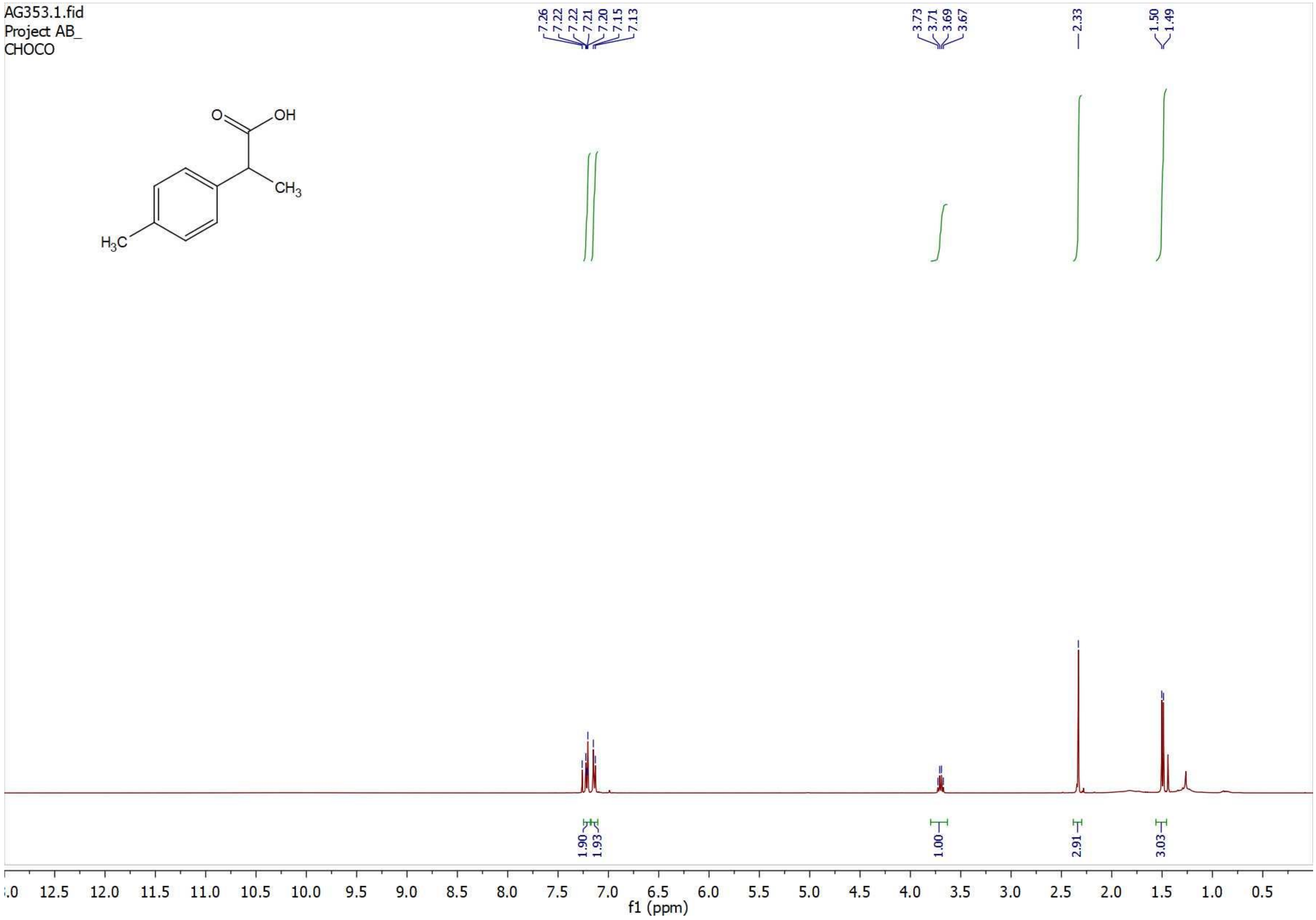
AG353.1.fid
Project AB_
CHOCO
7.26
7.22
7.22
7.21
7.20
7.15
7.13
3.73
3.71
3.69
3.67
2.33
1.50
1.49
O
OH
CH3
H3C
1.90
1.93
1.00
2.91
3.03
12.5
12.0
11.5
11.0
10.5
10.0
9.5
9.0
8.5
8.0
7.5
7.0
6.5
6.0
5.5
5.0
4.5
4.0
3.5
3.0
2.5
2.0
1.5
1.0
0.5
f1 (ppm)

Compound 7a

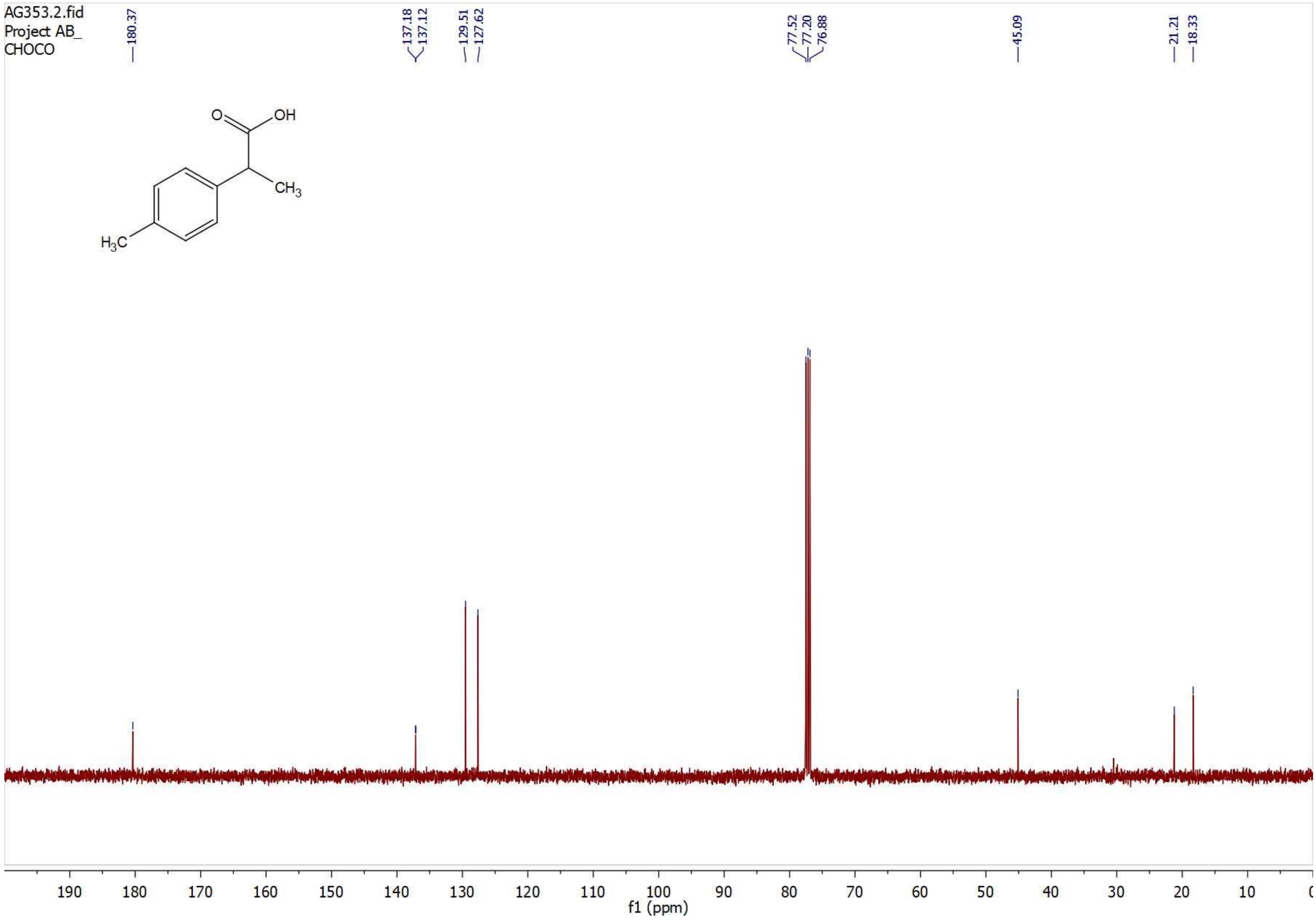

AG353.2.fid
Project AB_
CHOCO
180.37
137.18
137.12
129.51
127.62
77.52
77.20
76.88
45.09
21.21
18.33
OH
O
CH3
H3C
190
180
170
160
150
140
130
120
110
100
90
80
70
60
50
40
30
20
10
f1 (ppm)

Compound 9a

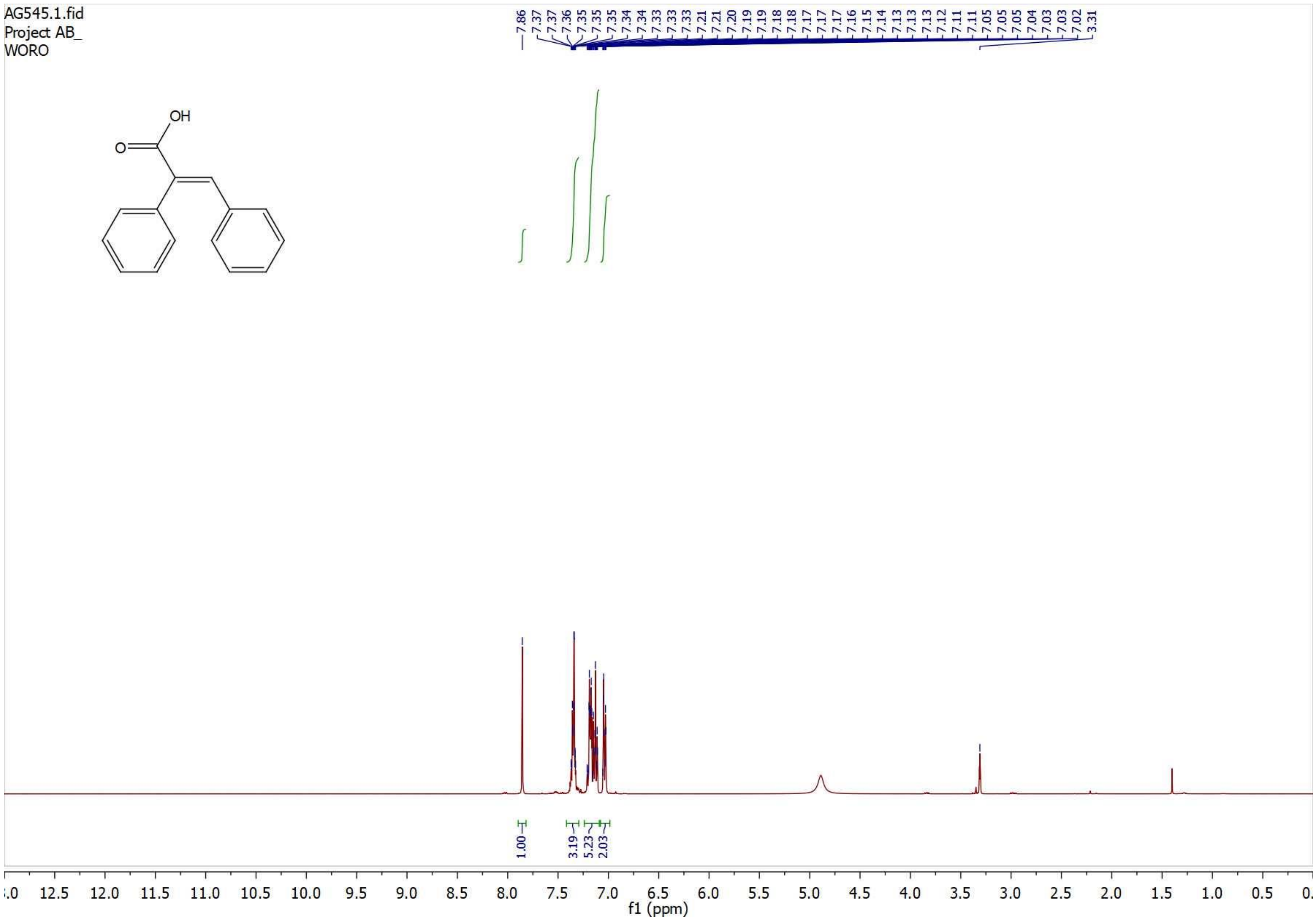
AG545.1.fid
Project AB_
WORO
OH
O
7.86
7.37
7.37
7.36
7.35
7.35
7.35
7.34
7.34
7.33
7.33
7.33
7.33
7.21
7.21
7.20
7.19
7.19
7.18
7.17
7.17
7.17
7.16
7.15
7.14
7.13
7.13
7.13
7.12
7.11
7.11
7.05
7.05
7.05
7.04
7.03
7.03
7.02
3.31
1.00
3.19
5.23
2.03
12.5 12.0 11.5 11.0 10.5 10.0 9.5 9.0 8.5 8.0 7.5 7.0 6.5 6.0 5.5 5.0 4.5 4.0 3.5 3.0 2.5 2.0 1.5 1.0 0.5
f1 (ppm)

Compound 9a

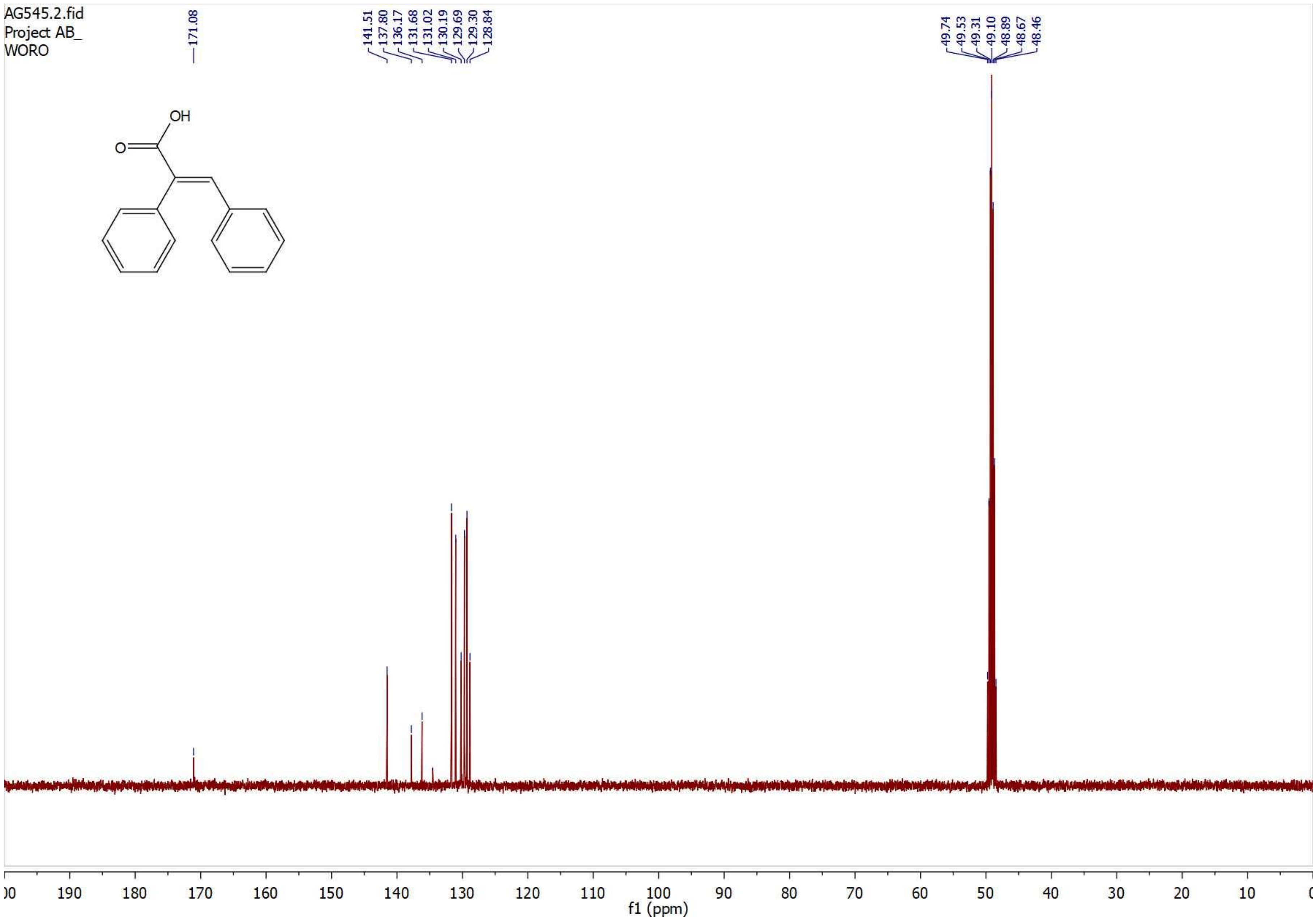
AG545.2.fid
Project AB_
WORO
171.08
141.51
137.80
136.17
131.68
131.02
130.19
129.69
129.30
128.84
49.74
49.53
49.31
49.10
48.89
48.67
48.46
OH
O
f1 (ppm)

Compound 10a

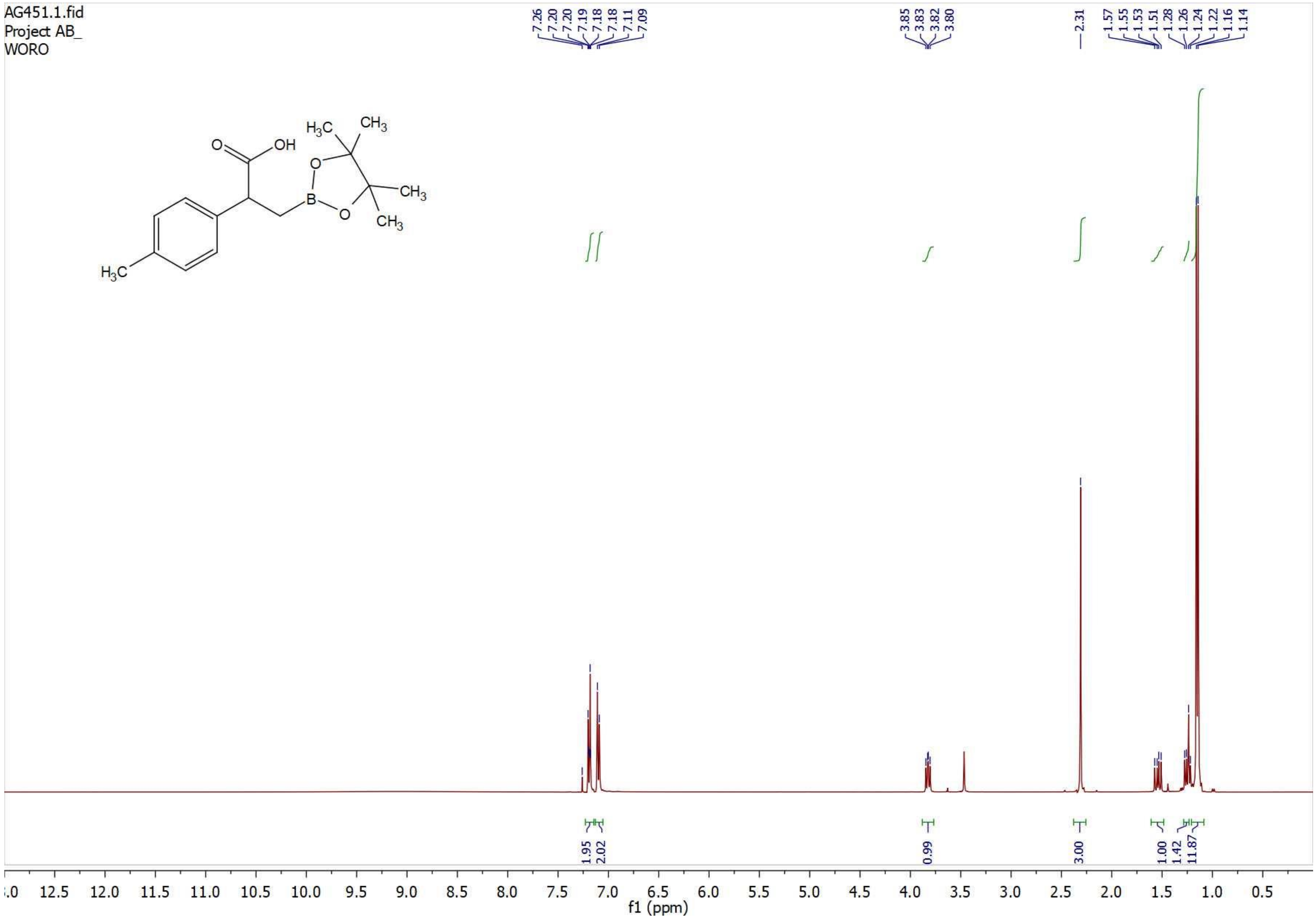

Compound 10a

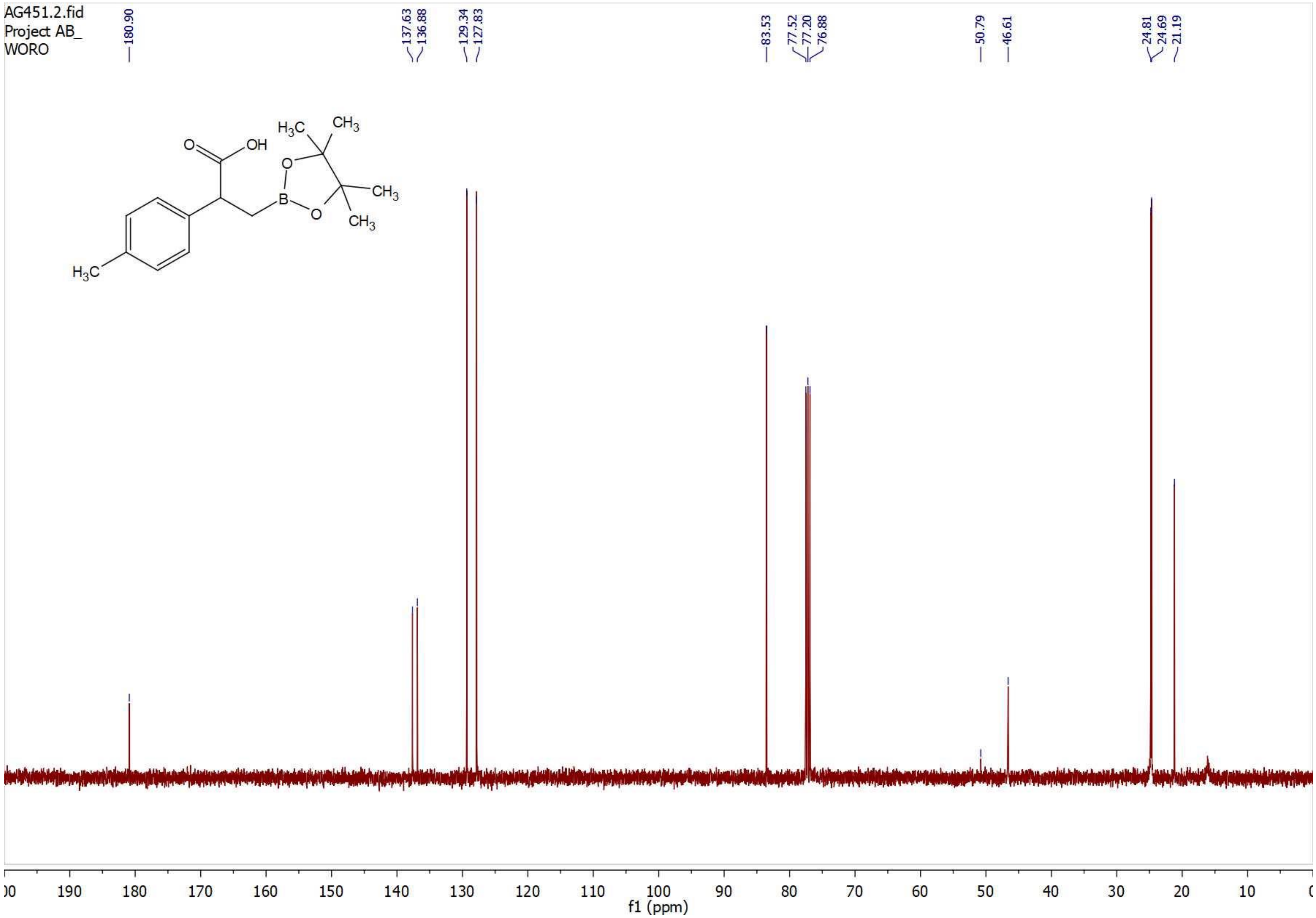

Compound 11a

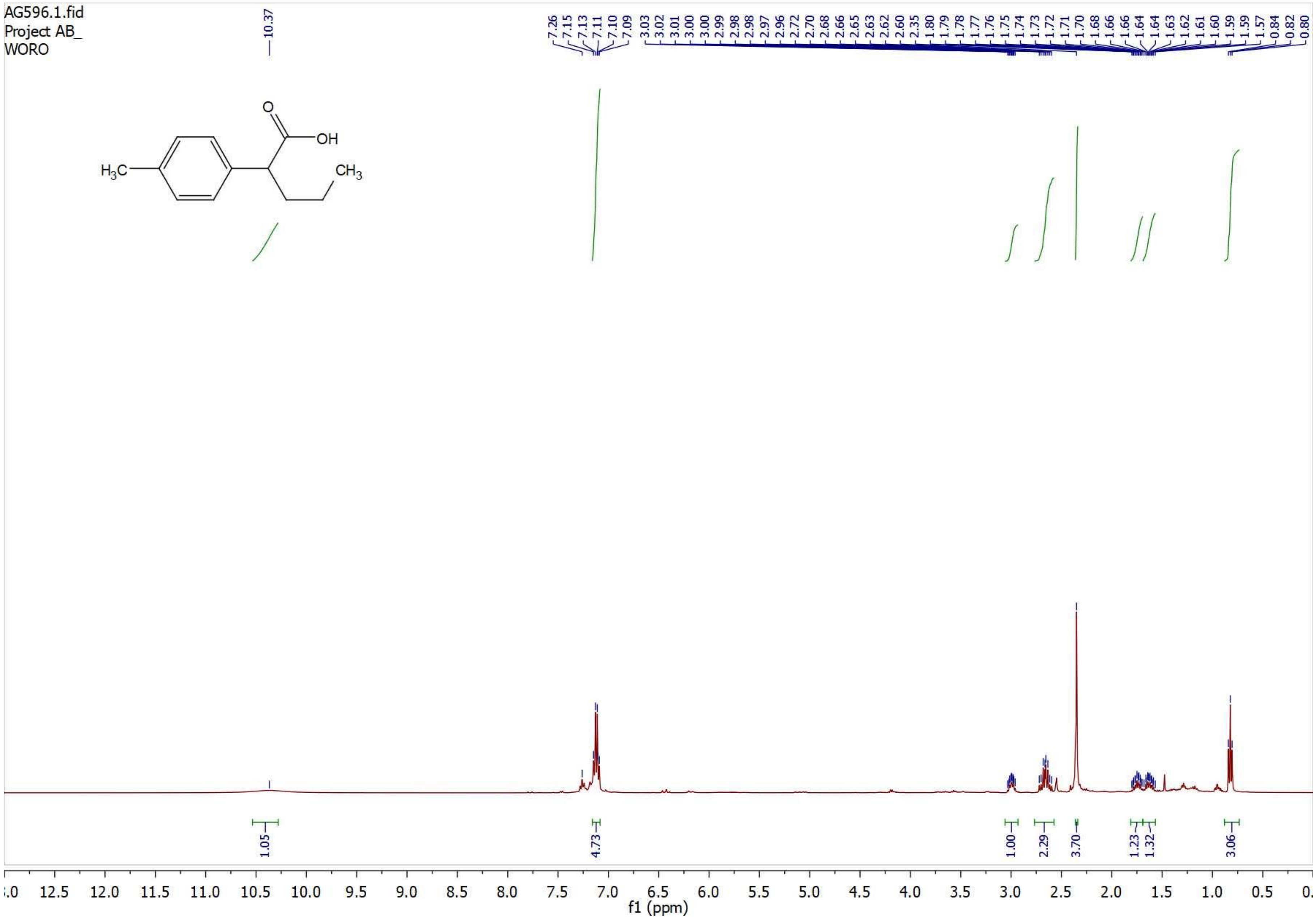

Compound 11a

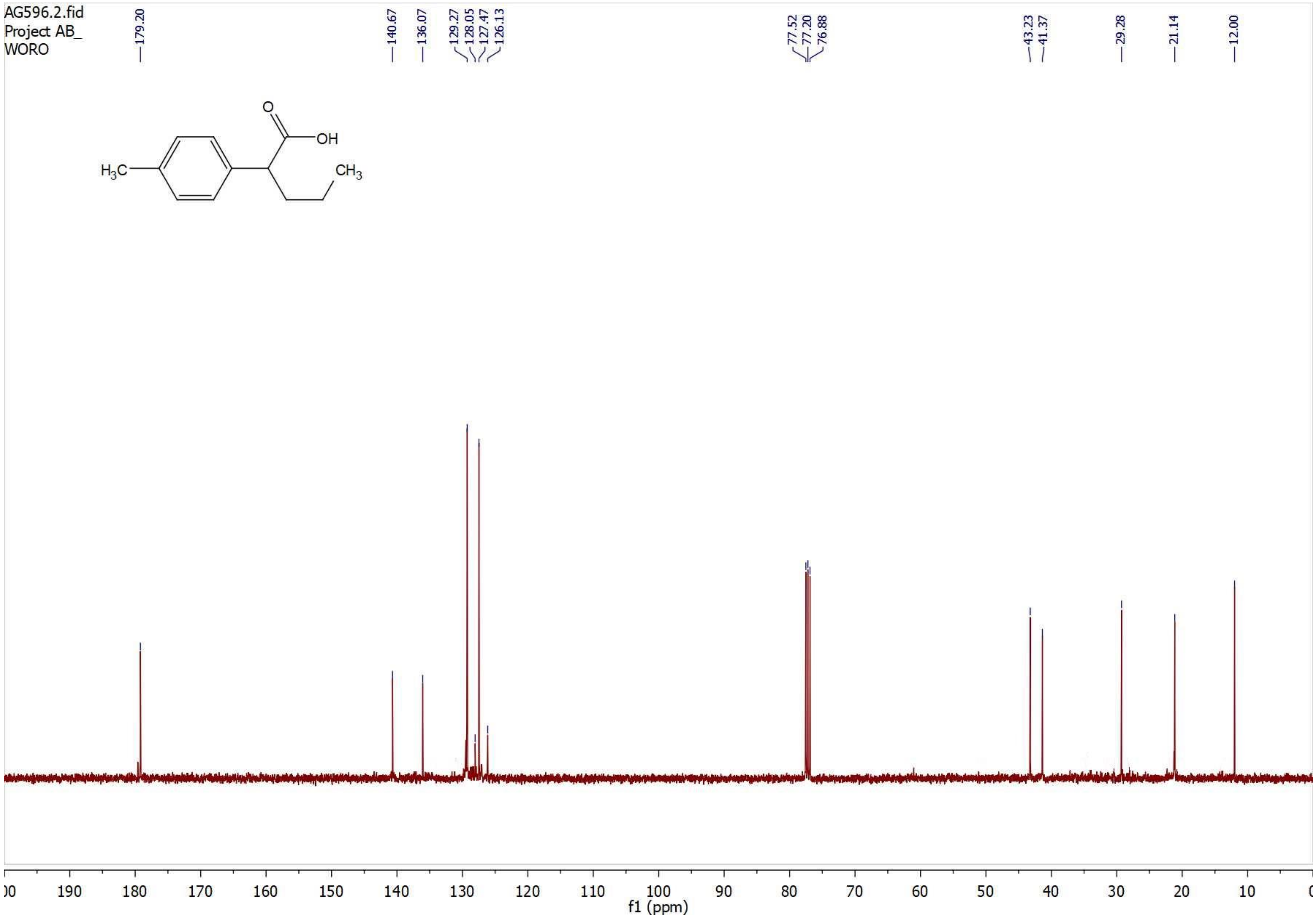

Supplement: Supplementary file 1 — Supplementary [file CSSC-13-2080-s001.pdf]
